# Enhanced solid-state phosphorescence of organoplatinum π-systems by ion-pairing assembly†

**DOI:** 10.1039/d3sc04564a

**Published:** 2023-12-05

**Authors:** Yohei Haketa, Kaifu Komatsu, Hiroi Sei, Hiroki Imoba, Wataru Ota, Tohru Sato, Yu Murakami, Hiroki Tanaka, Nobuhiro Yasuda, Norimitsu Tohnai, Hiromitsu Maeda

**Affiliations:** a Department of Applied Chemistry, College of Life Sciences, Ritsumeikan University Kusatsu 525-8577 Japan maedahir@ph.ritsumei.ac.jp; b Department of Applied Chemistry, Graduate School of Engineering, Osaka University Suita 565-0871 Japan; c MOLFEX, Inc. Kyoto 606-8103 Japan; d Fukui Institute for Fundamental Chemistry, Kyoto University Kyoto 606-8103 Japan; e Department of Molecular Engineering, Graduate School of Engineering, Kyoto University Kyoto 615-8510 Japan; f Beamline Division, Japan Synchrotron Radiation Research Institute Sayo 679-5198 Japan

## Abstract

Anion binding and ion pairing of dipyrrolyldiketone Pt^II^ complexes as anion-responsive π-electronic molecules resulted in photophysical modulations, as observed in solid-state phosphorescence properties. Modifications to arylpyridine ligands in the Pt^II^ complexes significantly impacted the assembling behaviour and photophysical properties of anion-free and anion-binding (ion-pairing) forms. The Pt^II^ complexes, in the presence of guest anions and their countercations, formed various anion-binding modes and ion-pairing assembled structures depending on constituents and forms (solutions and crystals). The Pt^II^ complexes emitted strong phosphorescence in deoxygenated solutions but showed extremely weak phosphorescence in the solid state owing to self-association. In contrast, the solid-state ion-pairing assemblies with tetraalkylammonium cations exhibited enhanced phosphorescence owing to the formation of hydrogen-bonding 1D-chain Pt^II^ complexes dispersed by stacking with aliphatic cations. Theoretical studies revealed that the enhanced phosphorescence in the solid-state ion-pairing assembly was attributed to preventing the delocalisation of the electron wavefunction over Pt^II^ complexes.

## Abstract 2

The solid-state ion-pairing assemblies in the form of anion complexes with tetraalkylammonium cations exhibited enhanced phosphorescence owing to the dispersion of emissive hydrogen-bonding 1D-chain Pt^II^ complexes by aliphatic cations.

## Graphical abstract

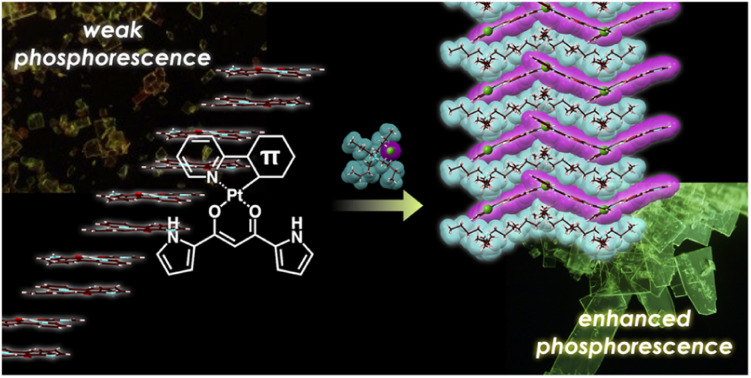

## Introduction

**Fig. 1:**
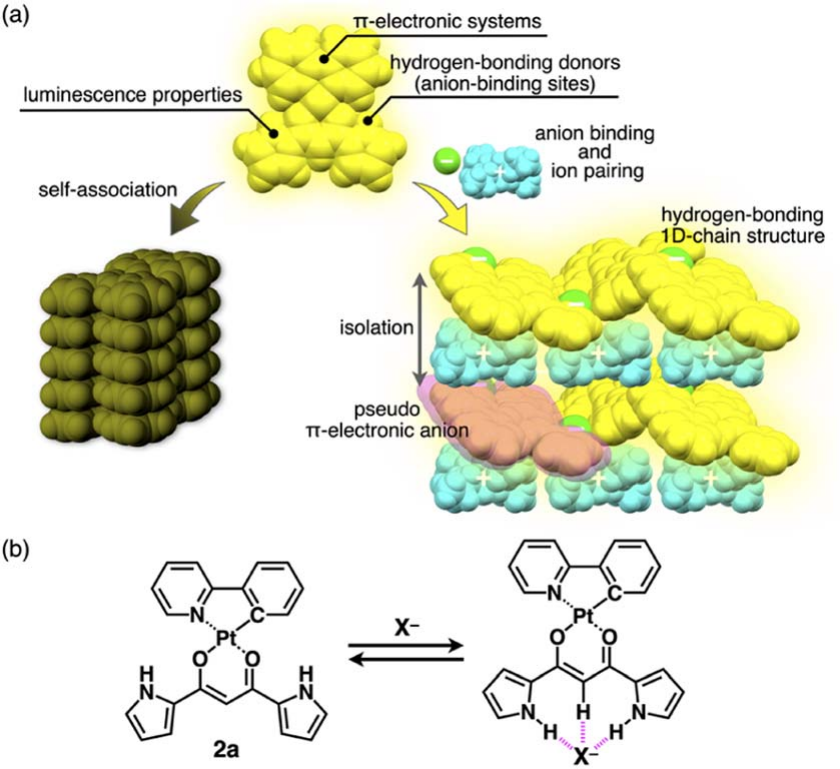
(a) Conceptual diagram of anion binding and ion pairing for emission enhancement: stacking structure of anion-responsive luminescent molecules (left) and charge-by-charge assembly with hydrogen-bonding 1D-chain structures of receptor–anion complexes as pseudo π-electronic anions (right) and (b) anion complexation of dipyrrolyldiketone Pt^II^ complex 2a.

π-Electronic molecules in ordered arrangements demonstrate fascinating electronic and electrooptical properties that are not observed in single molecules.^1^ Introducing multiple building units in assemblies induces properties that can be modulated by constituent species. Among organic materials, solid-state luminescent materials have received significant attention owing to their applications in light-emitting diodes, sensors and photonics.^2^ In particular, crystals of square-planar organoplatinum(ii) complexes exhibit fascinating photoluminescence properties,^3,4^ such as triplet energy transfer and phosphorescence anisotropy amplification.^4*h*,*j*^ The solid-state luminescence of π-electronic species is frequently quenched or weakened by self-association (Fig. 1a left), primarily because of exciton coupling between neighbouring molecules.^5,6^ To prevent this, introducing bulky groups to interfere with the molecular contact is an effective strategy for enhancing solid-state luminescence by electronic decoupling of organoplatinum π-systems.^4*c*,*e*,*j*,7^ Therefore, appropriate isolation arrangements for emissive species have been highly demanded. Furthermore, it is important to maintain rigid structures to decrease the rates of non-radiative decay processes that interfere with phosphorescent emission.^3*c*,8^ Thus, both isolation and robustness in packing structures are required for effective luminescence.

Ion pairs, which comprise complementarily associated positively and negatively charged species, can be employed for an isolation strategy.^9,10^ Isolation and electronic decoupling of luminescent cations have been achieved by introducing bulky counteranions.^11^ Furthermore, ion complexation by ion-responsive π-electronic molecules (receptors) followed by pairing with counterions is appropriate for preparing π-electronic ion pairs. This method can be used to create pseudo-π-electronic anions in the form of receptor–anion complexes. Spatially and electronically isolated charged fluorophores (cationic dyes) exhibit fluorescence emission in the solid state when combined with well-designed counter species (anion complexes).^12^ In contrast, including photo-functional anion-responsive π-electronic systems would also provide luminescent crystalline states through anion complexation with hydrogen bonding and alternate stacking with countercations (Fig. 1a right). The anion complexation would give rise to rigid structures by hydrogen bonds tightly connecting building units (pseudo-π-electronic anions comprising luminescent receptors) with the support of the charge-by-charge arrangement by electrostatic and dispersion forces. Designing luminescent anion receptors and combining them with countercations for isolation enable the control of the photophysical properties.

As luminescent π-electronic systems, dipyrrolyldiketone Pt^II^ complexes (*e.g.*, 2a, Fig. 1b), with phenylpyridine (ppy) as a C^N ligand, have been synthesized as phosphorescent anion sensors, exhibiting absorption and emission maxima (*λ*_max_ and *λ*_em_, respectively) at 410 and 510 nm, respectively, with an emission quantum yield (*Φ*_em_) of 42% for 2a in CH_2_Cl_2_.^13^ Planar geometries around Pt^II^ with a ppy ligand are suitable for stacking structures by themselves and in the form of ion-pairing assemblies.^14^ The Pt^II^ complexes would exhibit luminescent properties according to the introduced C^N ligands in solution and ion-pairing assemblies. Furthermore, the solid-state luminescent properties, whose control by countercations is also challenging, have not been elucidated. In this study, enhanced solid-state phosphorescence was achieved by ion pairing and the isolation of emissive anion complex moieties by countercations, for diverse Pt^II^ complexes.

## Results and discussion

### Synthesis and characterization of Pt^II^ complexes

**Fig. 2:**
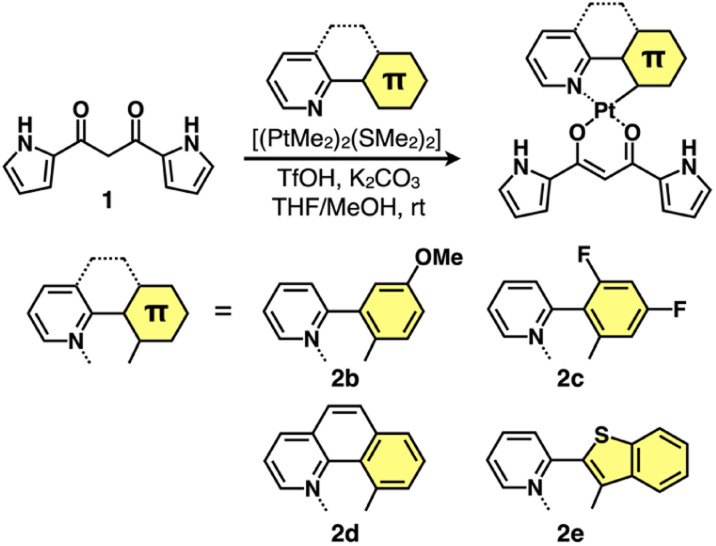
Synthesis of dipyrrolyldiketone Pt^II^ complexes 2b–e.

Four different C^N ligands were used to produce Pt^II^ complexes 2b–e in 7.0–27% yields by treating dipyrrolyldiketone 1 with the mixture of the ligands and [(PtMe_2_)_2_(SMe_2_)_2_]^15^ at r.t. in the presence of trifluoromethanesulfonic acid (TfOH) (1 equiv.) and K_2_CO_3_ (1.5 equiv.) (Fig. 2). The obtained Pt^II^ complexes were characterized by ^1^H and ^13^C NMR and ESI-TOF-MS. Conformations without pyrrole inversions (*py0i* conformations) were suggested by the theoretically optimized structures: doubly pyrrole-inverted (*py2i*) conformations of 2b–e, suitable for anion binding, were less stable by 6.84, 6.79, 7.10 and 6.15 kcal mol^−1^, respectively, due to the enhanced molecular electric dipoles.^16^

### Solution-state properties

**Fig. 3:**
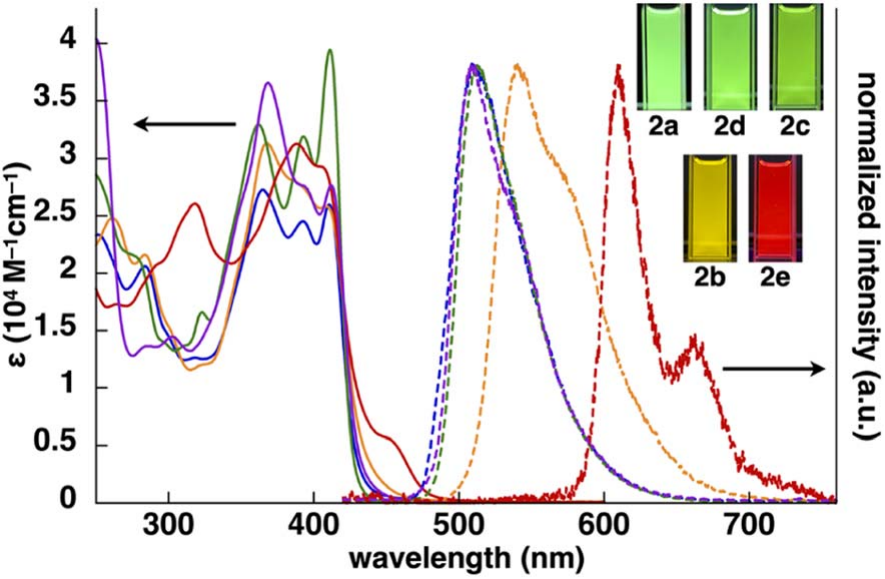
UV/vis absorption spectra (solid lines, CH_2_Cl_2_) and normalized emission spectra (dashed lines, deoxygenated CH_2_Cl_2_) with the excitation at 410, 368, 411, 368 and 388 nm of 2a (blue), 2b (orange), 2c (green), 2d (purple) and 2e (red) (inset: photographs of Pt^II^ complexes under UV_365_ (0.03 mM)).

The UV/vis absorption spectra of 2b–e in CH_2_Cl_2_ exhibited the *λ*_max_ at ∼310–450 nm, and the spectral features, *λ*_max_ values and absorbance intensities, were complicated depending on the introduced ligand moieties (Fig. 3 and Table 1). For example, 2b displayed the *λ*_max_ at 368, 390 and 412 nm, and similar spectral features were observed in 2c,d, whereas 2e exhibited a distinctive spectrum with the *λ*_max_ at 388 and 409 nm along with those at 318 and 455 nm. Similarly to 2a,^13^ the main absorption bands of 2b–e were assigned as the lowest-lying singlet states, originating primarily from the HOMO-to-LUMO transition with a significant contribution from ligand-to-ligand charge transfer (LLCT) from the dipyrrolyldiketone unit to the arylpyridine ligands and metal-to-ligand charge transfer (MLCT) from Pt^II^ to arylpyridine ligands; the theoretical study was conducted using time-dependent (TD)-DFT calculations at the CAM-B3LYP level using the 6-31+G(d,p) basis set with the LanL2DZ basis set and associated effective core potentials for Pt, which were used for the calculations in the following parts.^16^ Furthermore, characteristic absorption bands of 2e at 318 and 455 nm can be attributed to the π–π* transition of the benzothienyl-pyridine ligand and intraligand charge transfer from the π orbitals of the benzo unit to the π* orbitals of the pyridyl group, respectively.^17^

**Table 1:** Summary of the UV/vis absorption (*λ*_max_) and emission (*λ*_em_) maxima with quantum yields (*Φ*_em_) of 2b–e with 2a^a^ as a reference in CH_2_Cl_2_

	*λ*_max_ b [nm]	*λ*_em_ [nm]	*Φ*_em_ [%]	*τ* [μs]
2b	**368**/390/412	542	68	4.6
2c	361/393/**411**	512	50	9.6
2d	**368**/390/408	508	50	4.2
2e	**388**/409	610/662(sh)	16	6.8
2a^a^	364/393/**410**	510	42	5.1

a Ref. 13.

b Excitation wavelengths for emission spectra are in bold.

The phosphorescence spectra of 2b–e in deoxygenated CH_2_Cl_2_ exhibited broad emission bands with a *λ*_em_ of 542, 512, 508 and 610/662(sh) nm, respectively (Fig. 3 and Table 1), suggesting the red-shifted emissions for 2b,e compared to 2a. The *Φ*_em_ of 2b–d were 68%, 50% and 50%, respectively, which were greater than that of 2e (16%). The phosphorescence emissions of 2b–e originating from the triplet states were suggested by the emission lifetimes (*τ*) of 4.6, 9.6, 4.2 and 6.8 μs, respectively, similar to that of 2a (5.1 μs).^13^ TD-DFT calculations for the optimized T_1_ structures at the PCM-M06-2X level in CH_2_Cl_2_ showed theoretically estimated emission maxima at 499, 524, 495 and 714 nm for 2b–e, respectively, which are close to the observed values (Fig. S56†). The phosphorescence emissions of 2c,e were mainly derived from the LUMO-to-HOMO (84% and 96%, respectively) transitions, whereas those of 2b,d were ascribed to the LUMO-to-HOMO−1 (68%) and LUMO+1-to-HOMO (60%) transitions, respectively.

Anion-binding behaviours were revealed by ^1^H NMR spectral changes in CD_2_Cl_2_ (1.0 mM) (Fig. S74–S77†). Upon the addition of 3.3 equiv. of tetrabutylammonium chloride (TBACl) to, as an example, 2c, the signals of the pyrrole NH and bridging CH at 9.35/9.31 and 6.41 ppm, respectively, at −50 °C disappeared, whereas the corresponding new signals appeared at 12.55/12.49 and 7.42 ppm, respectively. The downfield shifts were caused by hydrogen bonding with Cl^−^, implying the formation of [1 + 1]-type Cl^−^ complexes, as demonstrated by theoretical studies (Fig. S39–S42†).^16^

The anion-binding constants (*K*_a_) of 2b–e in a [1 + 1]-binding mode were evaluated by the changes in UV/vis absorption spectra caused by the addition of anions (Cl^−^, Br^−^ and CH_3_CO_2_^−^) as TBA salts in CH_2_Cl_2_ (Table 2 and Fig. S70–S73†). In all the derivatives discussed in this study, the *K*_a_ values were in the order of CH_3_CO_2_^−^ > Cl^−^ > Br^−^ correlating with the basicity. Cl^−^ complexation produced phosphorescence emissions (*λ*_em_ at 542, 505, 511 and 610/660 nm for 2b–e, respectively) with *Φ*_em_ (54%, 32%, 41% and 14%, respectively) and *τ* (4.3–14.2 μs), which were comparable to those of 2a·Cl^−^ (*λ*_em_: 490/520 nm, *Φ*_em_: 48%, τ: 2.4 μs) (Table 3). It should be noted that anion complexation of the Pt^II^ complexes in solution did not significantly affect the emission properties. Pt^II^ complexes with such emission properties can be used as building blocks for solid-state materials whose packing structures and resulting luminescent properties are modulated by anion complexation and ion pairing with countercations.

**Table 2:** Anion-binding constants (*K*_a_, M^−1^) of 2b–e with 2a^a^ as a reference in CH_2_Cl_2_

	2b	2c	2d	2e	2a^a^
Cl^−^	2000	2200	1200	1900	1300
Br^−^	320	240	50	310	61
CH_3_CO_2_^−^	7400	22 000	5400	15 000	15 000

a Ref. 13.

**Table 3:** Summary of the UV/vis absorption (*λ*_max_) and emission (*λ*_em_) maxima with quantum yields (*Φ*_em_) of 2b–e with 2a^a^ as a reference upon the addition of TBACl (2000 equiv. for 2b–e and 3000 equiv. for 2d) in CH_2_Cl_2_

	*λ*_max_ b [nm]	*λ*_em_ [nm]	*Φ*_em_ [%]	*τ* [μs]
2b	**372**/387/410	542	54	4.3
2c	367/390/**409**	505	32	6.4
2d	**372**/411	511	41	14.2
2e	**388**/409	610/660(sh)	14	6.8
2a^a^	369/389/**410**	490/520(sh)	48	2.4

a Ref. 13.

b Excitation wavelengths for emission spectra with bold.

### Solid-state luminescence and packing structures

**Fig. 4:**
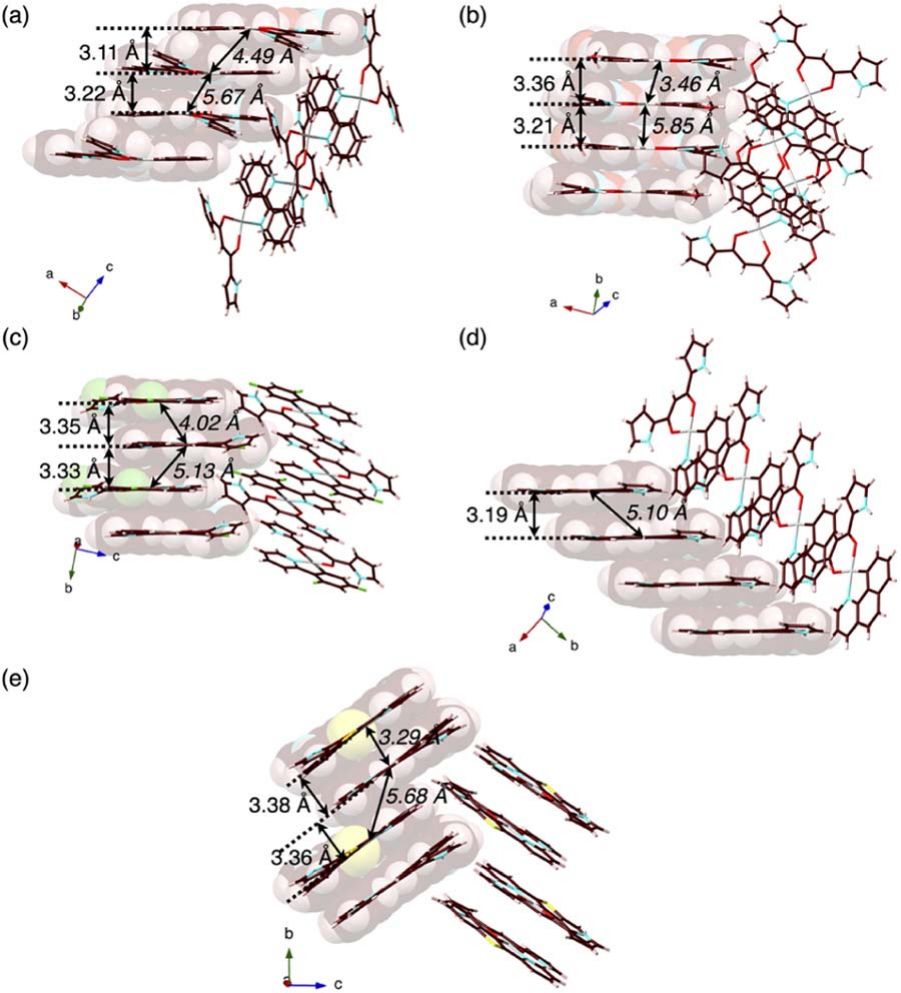
Single-crystal X-ray structures, with selected stacking and Pt⋯Pt (italic) distances, of (a) 2a as a reference,^13^ and (b) 2b, (c) 2c, (d) 2d and (e) 2e as packing diagrams. Atom colour codes in Fig. 4 and the following figures: brown, pink, blue, red, yellow green, yellow and grey refer to carbon, hydrogen, nitrogen, oxygen, fluorine, sulfur and platinum, respectively.

In contrast to the phosphorescence emission in the dispersed solution state, the solid-state Pt^II^ complexes obtained from the single crystals (*vide infra*) exhibited extremely weak luminescence: 2a–e exhibited *λ*_em_ at 523/682, 591, 673, 652 and 627 nm with *Φ*_em_ of 0.7%, ∼0.1%, 1.8%, ∼0.1% and ∼0.2%, respectively (Fig. S86–S90†).^18^ Such red-shifted emission with emission quenching can be caused by the excimer formation of excited Pt^II^ complexes,^19^ as theoretically discussed in the following section. Notably, the *Φ*_em_ of solid-state 2a–e are lower than those of 1,3-diphenyl-1,3-propanedione Pt^II^ complexes.^4*h*^ The details of the assembled structures were revealed by X-ray analysis for the single crystals of 2b–e obtained by vapour diffusion of *n*-hexane into CH_2_Cl_2_ solutions (Fig. 4 and S19–S23†).^20^2b–e exhibited planar pyrrole-non-inverted (*py0i*) conformations in stacking structures, similar to 2a,^13^ with mean-plane deviations (defined by the core atoms without hydrogen atoms) of 0.103–0.174 Å. The Pt^II^ complexes 2b–e formed columnar π–π stacking structures with stacking distances of 3.21–3.38 Å. Among them, 2b,e showed stacked-dimer alignment with Pt⋯Pt distances of 3.46 and 3.29 Å, respectively. The π–π stacking structures were identified by Hirshfeld surface analysis (Fig. S33–S37†).^20^ There are no notable intermolecular interactions on the lateral side of the columnar π–π stacking structures except for 2c, which showed N–H⋯π interactions between pyrrole rings.

**Fig. 5:**
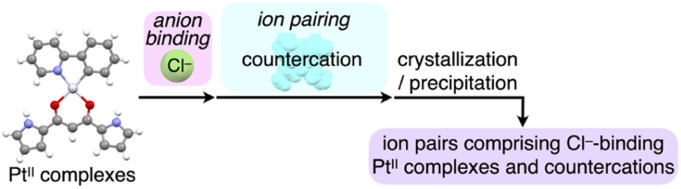
Preparation procedures of ion-pairing assemblies comprising Cl^−^-binding Pt^II^ complexes and countercations.

**Fig. 6:**
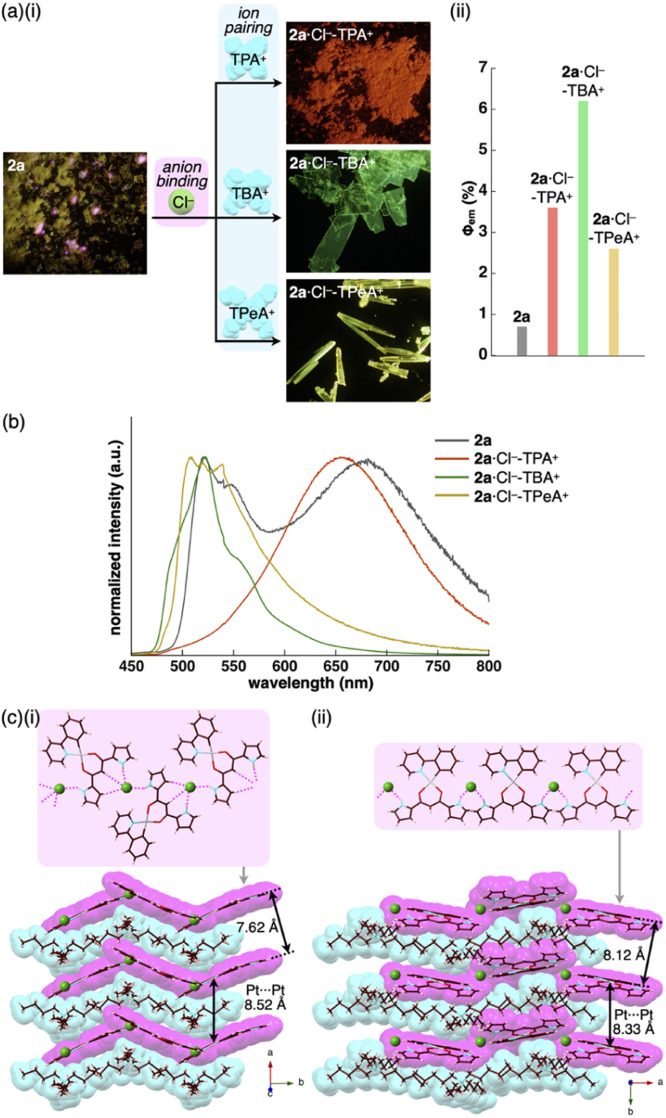
(a) (i) Photographs and (ii) quantum yields of solid-state 2a,^13^2a·Cl^−^-TPA^+^, 2a·Cl^−^-TBA^+^ and 2a·Cl^−^-TPeA^+^, (b) solid-state emission spectra of 2a (grey), 2a·Cl^−^-TPA^+^ (red), 2a·Cl^−^-TBA^+^ (green) and 2a·Cl^−^-TPeA^+^ (yellow) and (c) single-crystal X-ray structures of (i) 2a·Cl^−^-TBA^+^ and (ii) 2a·Cl^−^-TPeA^+^ as top 1D chain and side packing structures. The atom colour code in (c): green (spherical) refers to chlorine. Magenta and cyan denote the receptor–Cl^−^ complex 1D-chain and cation parts, respectively. For the crystals of 2a in (a), reflections of excitation light at UV_365 nm_ were observed in partially pink colour.

The extremely weak crystal-state emissive properties were modulated by isolating the Pt^II^ complexes without stacking by themselves. Single crystals of the ion pairs 2a·Cl^−^-TBA^+^ and 2a·Cl^−^-TPeA^+^ (TPeA^+^: tetrapentylammonium) were prepared by vapour diffusion of *n*-hexane into the THF solutions of 2a and corresponding tetraalkylammonium salts (Fig. 5). The ion pair 2a·Cl^−^-TPA^+^ (TPA^+^: tetrapropylammonium) was also prepared as precipitates by adding *n*-hexane to a mixture of 2a and TPACl in CH_2_Cl_2_.^21^ Compositions of the Cl^−^-binding Pt^II^ complex and countercations in all the solid-state samples were fully characterized by using ^1^H NMR. In contrast to the anion-free states, solid-state 2a·Cl^−^-TPA^+^, 2a·Cl^−^-TBA^+^ and 2a·Cl^−^-TPeA^+^ exhibited phosphorescence with the *λ*_em_ at 654, 522 and 509 nm with *Φ*_em_ (relative intensities to the solid-state 2a) of 3.6% (5.1), 6.2% (8.9) and 2.6% (3.7), respectively, indicating enhanced phosphorescence properties (Table 4, Fig. 6a, b and S86†). The *λ*_em_ of 2a·Cl^−^-TBA^+^ and 2a·Cl^−^-TPeA^+^ were similar to the solution-state *λ*_em_ of 2a·Cl^−^, suggesting the phosphorescence derived from the monomeric Cl^−^ complex as suggested by single-crystal packing structures (*vide infra*). Notably, 2a·Cl^−^-TPA^+^ exhibited a distinctive red-shifted emission. The emission lifetimes, such as 440 μs for 2a·Cl^−^-TPeA^+^, were nearly 200 times longer than those of the monomers in solution (Fig. S82†).

**Table 4:** Solid-state properties (emission peaks, emission lifetimes and quantum yields) of 2a and its ion pairs of Cl^−^ complexes with tetraalkylammonium cations

	*λ*_em_ [nm]	*τ*_1_ [μs]/*f*_1_ [%]	*τ*_2_ [μs]/*f*_2_ [%]	*τ*_3_ [μs]/*f*_3_ [%]	*Φ*_em_ [%]
2a	523	140/31	550/69	—	0.7
2a·Cl^−^-TPA^+^	654	110/24	440/76	—	3.6
2a·Cl^−^-TBA^+^	522	2.9/43	23/25	210/32	6.2
2a·Cl^−^-TPeA^+^	509	6.3/8	86/24	440/68	2.6

The enhanced phosphorescence intensities in the ion-pairing assemblies were investigated using solid-state packing structures revealed by single-crystal X-ray analysis. The ion pair 2a·Cl^−^-TBA^+^ exhibited an anion-binding mode with hydrogen bonding of the singly inverted pyrrole NH, bridging CH and pyrrole CH (Fig. 6c(i), S24† and Table 5). The Cl^−^ also interacted with the pyrrole NH of neighbouring 2a, resulting in a Cl^−^-bridged 1D-chain structure based on the singly pyrrole-inverted (*py1i*) conformation. Importantly, 2a·Cl^−^ and TBA^+^ are alternately arranged on the *a*-axis to form a charge-by-charge assembly with a Pt⋯Pt distance of 8.52 Å. In contrast, crystal-state 2a·Cl^−^-TPeA^+^ exhibited a packing structure with a *py0i* conformation (Fig. 6c(ii) and S25†). The pyrrole NHs of 2a formed hydrogen bonds independently, resulting in a Cl^−^-bridged chain structure. 2a·Cl^−^ and TPeA^+^ were alternately arranged to form a charge-by-charge assembly with a Pt⋯Pt distance of 8.34 Å. The assembling modes, with different numbers of inverted pyrrole rings, are determined by the alkyl chain lengths of the countercations (TBA^+^ and TPeA^+^), resulting in different nearest Cl^−^···Cl^−^ distances of 8.90 and 10.72 Å, respectively. Hirshfeld surface analysis of the crystal structures indicated no characteristic close contacts between the Pt^II^ complex and cation, suggesting that both cations exhibited similar interactions. This is also supported by the energy decomposition analysis (EDA) in the framework of the fragment molecular orbital method at the FMO2-MP2 using mixed basis sets including NOSeC-V-DZP with MCP with TZP for Pt, demonstrating that the dispersion forces were effective between the Pt^II^ complex and cations (Fig. S57 and S58†).^22–25^ Although the exact assembling structure for the precipitates of 2a·Cl^−^-TPA^+^ could not be determined by single-crystal X-ray analysis, synchrotron XRD analysis revealed no characteristic diffraction pattern, suggesting the less ordered arrangement of 2a·Cl^−^ and TPA^+^ (Fig. S83†). The speculated slipped stacking of 2a·Cl^−^ in the solid-state 2a·Cl^−^-TPA^+^ could be correlated with the red-shifted phosphorescence.

**Table 5:** Summary of the representative distances in the crystal structures of 2a·Cl^−^-TBA^+^ and 2a·Cl^−^-TPeA^+^

	2a·Cl^−^-TBA^+^	2a·Cl^−^-TPeA^+^
N(–H)⋯Cl^−^ [Å]	3.17, 3.18	3.14
C_bridging_(–H)⋯Cl^−^ [Å]	3.78	—
C_β_(–H)⋯Cl^−^ [Å]	3.63	—
Pt⋯Pt^a^ [Å]	8.52	8.33
Cl^−^···Cl^−^b [Å]	8.90	10.72

a Distances in charge-by-charge assemblies.

b Distances in Cl^−^-bridged 1D-chain structures.

In any ion pairs of 2a·Cl^−^, the isolation of the Pt^II^ complexes by aliphatic cations, required for enhancing phosphorescence intensities, was clearly indicated by the X-ray structures of the ion-pairing assemblies. Furthermore, the rigidification of packing structures by Cl^−^-bridged 1D-chain structures is vital for enhancing the phosphorescence intensities. The larger *Φ*_em_ of 2a·Cl^−^-TBA^+^ than that of 2a·Cl^−^-TPeA^+^ can be ascribed to the stabilization of the 1D-chain structure by hydrogen bonding. The electrostatic energy, revealed by EDA, originating mainly from hydrogen-bonding interactions for the 2a⋯Cl^−^···2a structure in the 1D-chain of 2a·Cl^−^-TBA^+^ possessed a larger absolute value by 10.7 kcal mol^−1^ than that of 2a·Cl^−^-TPeA^+^ (Fig. S57 and S58†).

**Fig. 7:**
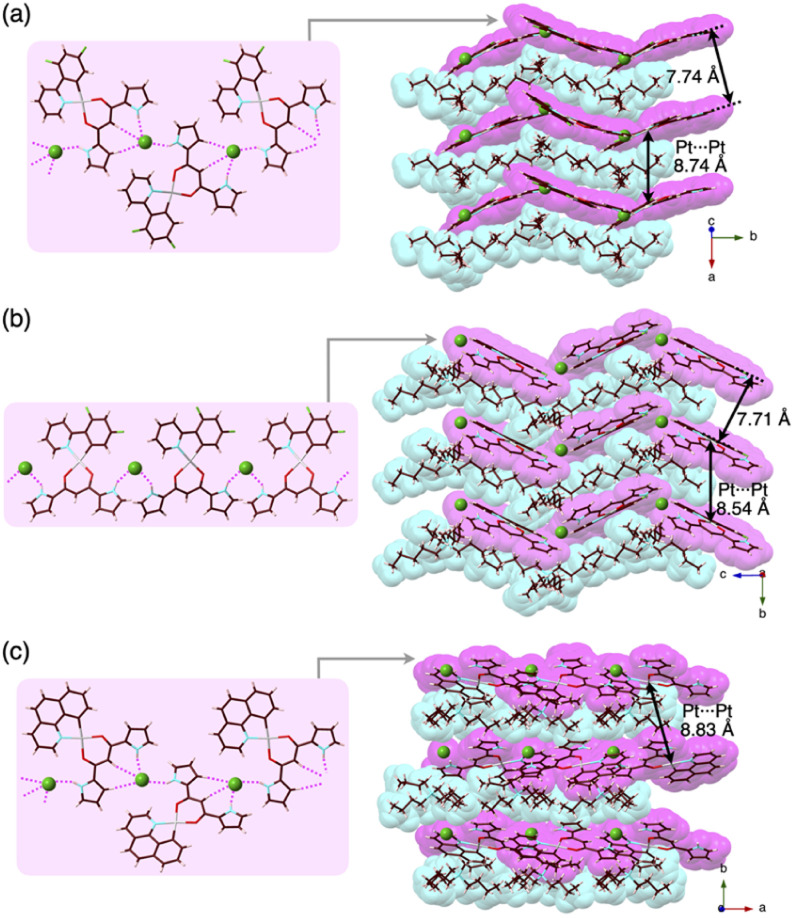
Single-crystal X-ray structures of (a) 2c·Cl^−^-TBA^+^, (b) 2c·Cl^−^-TPeA^+^ and (c) 2d·Cl^−^-TBA^+^_E_ as top 1D-chain and side packing structures.

Charge-by-charge assemblies were observed in the single-crystal packing structures of 2c,d with tetraalkylammonium cations (Fig. 7a, S28–S31† and Table 6). A crystal of 2c·Cl^−^-TBA^+^, prepared from CH_2_ClCH_2_Cl/*n*-hexane, for example, demonstrated a *py1i* conformation with Cl^−^ binding by pyrrole NH, β-CH and bridging CH. A Cl^−^-bridged chain structure similar to 2a·Cl^−^-TBA^+^ was formed by the interaction between uninverted pyrrole NH and neighbouring Cl^−^. In contrast, a single crystal of 2c·Cl^−^-TPeA^+^, prepared from CH_2_ClCH_2_Cl/*n*-hexane, exhibited a *py0i* conformation and a chain structure (Fig. 7b and S29†) as observed in 2a·Cl^−^-TPeA^+^. Furthermore, crystallization of 2d·Cl^−^-TBA^+^ from CH_2_Cl_2_/*n*-hexane (2d·Cl^−^-TBA_M_^+^) and CH_2_ClCH_2_Cl/*n*-hexane (2d·Cl^−^-TBA_E_^+^) produced two pseudo polymorphs, with 2d exhibiting *py1i* conformations that form Cl^−^-bridged chain structures in both (Fig. 7c and S31†). In all the ion-pairing assemblies revealed by X-ray analysis, Pt^II^ complexes in Cl^−^-bridged polymers were isolated by aliphatic cations in charge-by-charge arrangements.

**Table 6:** Summary of the representative distances in the crystal structures of 2c·Cl^−^-TBA^+^, 2c·Cl^−^-TPeA^+^ and 2d·Cl^−^-TBA^+^_E_

	2c·Cl^−^-TBA^+^	2c·Cl^−^-TPeA^+^	2d·Cl^−^-TBA_E_^+^
N(–H)⋯Cl^−^ [Å]	3.14, 3.18, 3.19, 3.20	3.14, 3.18	3.10, 3.16
C_bridging_(–H)⋯Cl^−^ [Å]	3.84, 3.85	—	3.82
C_β_(–H)⋯Cl^−^ [Å]	3.65, 3.72	—	3.73
Pt⋯Pt^a^ [Å]	8.74	8.33	8.83
Cl^−^···Cl^−^b [Å]	9.01	10.94	8.87

a Distances in charge-by-charge assemblies.

b Distances in Cl^−^-bridged 1D-chain structures.

**Fig. 8:**
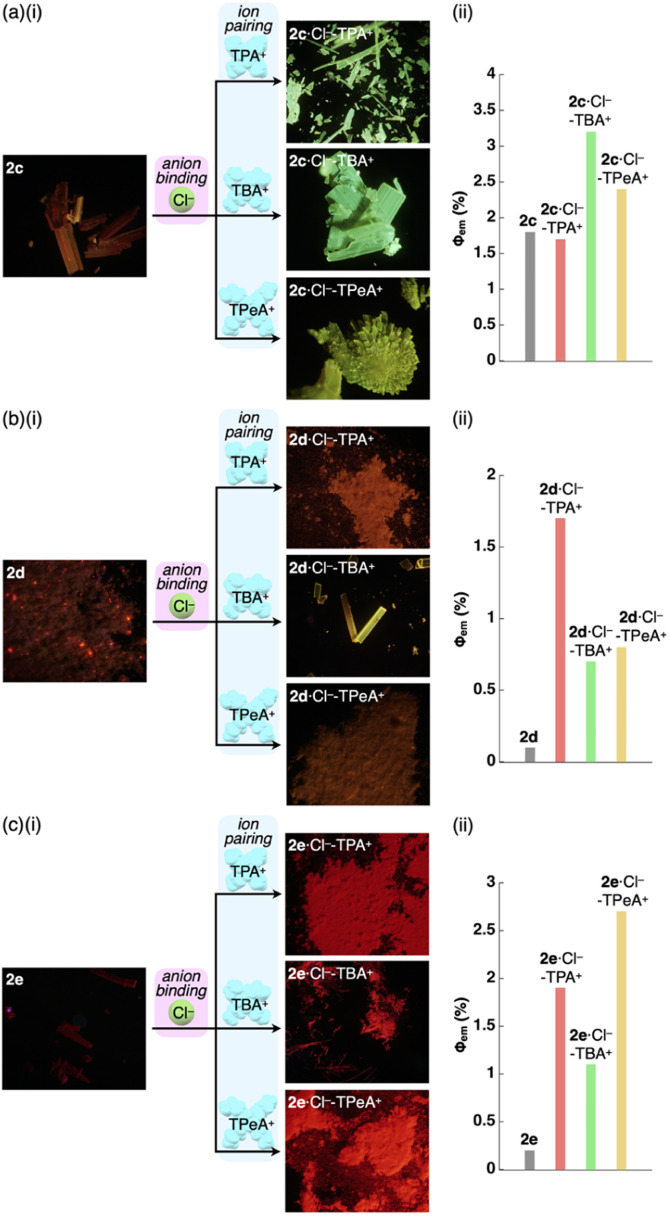
(i) Photographs and (ii) quantum yields of the Pt^II^ complexes (a) 2c, (b) 2d and (c) 2e and their Cl^−^ complexes as ion pairs with TPA^+^, TBA^+^ (2d·Cl^−^-TBA_E_^+^ for 2d) and TPeA^+^ as solid-state samples.

As expected from the crystal packing structures, emission enhancement of the solid-state ion-pairing assemblies of 2c,d was observed, as was a similar tendency for 2a (Table 7, Fig. 8a, b, S88 and S89†). For example, solid-state ion pairs 2c·Cl^−^-TPA^+^,^26^2c·Cl^−^-TBA^+^ and 2c·Cl^−^-TPeA^+^ showed the *λ*_em_ at 507, 504 and 515 nm with comparable and enhanced *Φ*_em_ of 1.7%, 3.2% and 2.4%, respectively, due to the formation of charge-by-charge assemblies.^27^ Similar to the XRD pattern of 2a·Cl^−^-TPA^+^, the synchrotron XRD pattern of 2d·Cl^−^-TPA^+^/TPeA^+^ precipitates exhibited less clear diffraction patterns with broad peaks, suggesting less ordered arrangements of 2d·Cl^−^ and countercations. The *Φ*_em_ of 2d·Cl^−^-TPA^+^ was estimated to be 1.7%, which is greater than that of the other 2d·Cl^−^ ion pairs. Substituents at π-electronic ligands controlled emissive properties, as observed in the red-shifted phosphorescence at 606–617 nm with enhanced quantum yields of 1.1–2.7% for the ion pairs of 2e·Cl^−^ with tetraalkylammonium cations as precipitates (Table 7, Fig. 8c and S90†). Furthermore, the 2b·Cl^−^-TPA^+^ precipitate obtained from CH_2_Cl_2_/*n*-hexane exhibited enhanced phosphorescence with a *Φ*_em_ of 7.5%, which is 75 times greater than that of 2b (Fig. S87†).^28^ The solid-state phosphorescence properties, *λ*_em_ and *Φ*_em_, in ion-pairing assemblies of the anion-responsive Pt^II^ complexes were modulated by the introduced π-electronic C^N ligands and coexisting cations.

**Table 7:** Solid-state properties (emission peaks, emission lifetimes and quantum yields) of 2c–e and its ion pairs of Cl^−^ complexes with tetraalkylammonium cations

	*λ*_em_ [nm]	*τ*_1_ [μs]/*f*_1_ [%]	*τ*_2_ [μs]/*f*_2_ [%]	*τ*_3_ [μs]/*f*_3_ [%]	*Φ*_em_ [%]
2c	673	61/90	450/10	—	1.8
2c·Cl^−^-TPA^+^	507	49/15	390/85	—	1.7
2c·Cl^−^-TBA^+^	504	6.3/41	80/14	410/44	3.2
2c·Cl^−^-TPeA^+^	515	7.7/10	57/25	390/65	2.4
2d	652	120/28	500/72	—	∼0.1^a^
2d·Cl^−^-TPA^+^	627	29/36	190/64	—	1.7
2d·Cl^−^-TBA^+^	563	120/26	490/74	—	0.7
2d·Cl^−^-TPeA^+^	594	30/17	330/83	—	0.8
2e	627	100/21	440/79	—	∼0.2^a^
2e·Cl^−^-TPA^+^	617	130/28	510/72	—	1.9
2e·Cl^−^-TBA^+^	606	130/27	510/73	—	1.1
2e·Cl^−^-TPeA^+^	606	96/18	420/82	—	2.7

a Accurate values could not be determined due to low *Φ*_em_.

### Mechanism for phosphorescence enhancement

Enhancing the phosphorescence in the ion-pairing assembly of Pt^II^ complexes was elucidated by DFT calculations based on an ONIOM approach^29,30^ for the packing structures of 2a and 2a·Cl^−^-TBA^+^. Computational models for solid-state 2a and 2a·Cl^−^-TBA^+^ were constructed by cutting out the 1 × 1 × 2 and 3 × 2 × 2 unit cells (Fig. S61 and S67†), where the centre parts and surroundings were treated as quantum mechanics (QM) regions at the DFT level and molecular mechanics (MM) regions at the UFF level, respectively. The electronic structures of the QM region were calculated at the CAM-B3LYP/6-31+G(d,p) and 3-21G with LanL2DZ for Pt in 2a and 2a·Cl^−^-TBA^+^, respectively. In both cases, the QM regions exhibited *C*_i_ site symmetry.

**Fig. 9:**
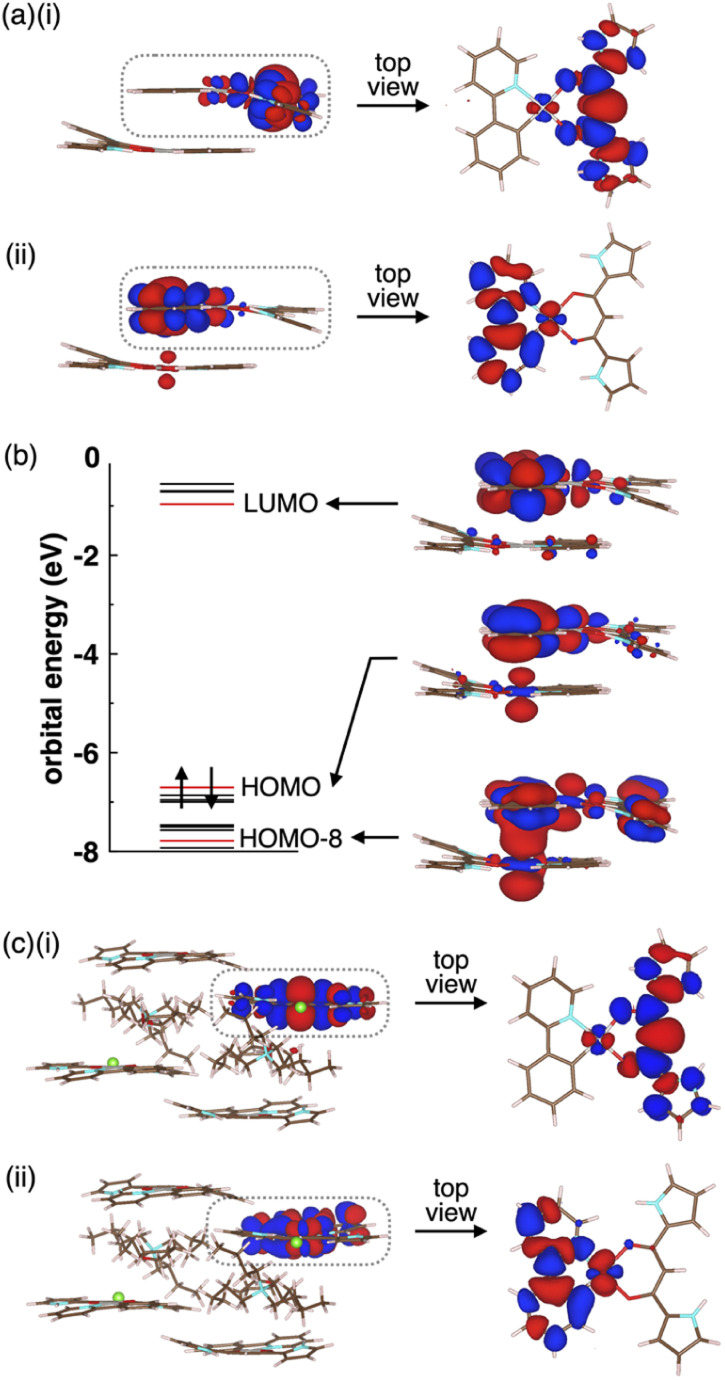
(a) Electron density differences for solid-state 2a (i) between S_0_ and T_1_ at the T_1_ optimized structure and (ii) between S_0_ and T_4_ at the T_4_ optimized structure (isosurface value: 5 × 10^−4^ a.u), (b) orbital levels and molecular orbitals at the T_4_ optimized structure (isosurface value: 2 × 10^−2^ a.u.) and (c) electron density differences for solid-state 2a·Cl^−^-TBA^+^ (i) between S_0_ and T_1_ at the T_1_ optimized structure and (ii) between S_0_ and T_6_ at the T_6_ optimized structure (isosurface value: 5 × 10^−4^ a.u.). The red and blue regions are positive and negative in electron density differences, respectively. Only the QM region is shown for simplicity.

The optimized S_0_ geometry of solid-state 2a showed pseudo-generate T_1_ (A_g_) and T_2_ (A_u_), where A_g_ and A_u_ denote the irreducible representations of the excited electronic states, as well as T_3_ (A_u_) and T_4_ (A_g_) states, whose wavefunctions were symmetrically delocalized over the 2a dimer (Fig. S62†). The geometries of T_1_ (A_g_) and T_4_ (A_g_) were further optimized owing to the distribution of their wavefunctions at the dipyrrolyldiketone and ppy ligand, respectively. The optimized T_1_ and T_4_ states with lower symmetries of *C*_1_ owing to a symmetry breaking of pseudo-Jahn–Teller distortion^31^ exhibited the lowest excited states with excitation energies of 2.25 eV/551 nm and 1.95 eV/636 nm, respectively (Fig. S63†). The S_0_–T_1_ electron density difference at the T_1_ optimized structure (Fig. 9a(i)) demonstrated that T_1_ was an excited state localized on the single molecule. In contrast, the S_0_–T_4_ electron density difference at the T_4_ optimized structure (Fig. 9a(ii)) indicated that T_4_ was an excited state with the wavefunction asymmetrically delocalized over the dimer. The calculated phosphorescence spectrum from T_1_ reproduced the sharp shape of the experimental spectrum in the short-wavelength region, whereas that from T_4_ reproduced the broad shape in the long-wavelength region (Fig. S64†). The T_4_ state mainly comprised the HOMO–LUMO transition (the CI coefficient: 0.599) and HOMO-8–LUMO transition (0.234). The HOMO and HOMO-8 originated from the intermolecular interaction between the Pt d_*z*^2^_ orbital in one molecule and the π orbital of the ppy ligand in the other (Fig. 9b).^32^ Thus, this intermolecular interaction was responsible for the energetically stable T_4_ formation.

The *Φ*_em_ values depend on nonradiative rate constants from the excited to the ground states. The rate constant significantly increases with diagonal vibronic coupling constants (VCCs) of the final electronic state because of easier acceptance of electronic excitation energy.^33^ For the solid-state 2a, the diagonal VCCs of S_0_ at the T_4_ optimized structure were larger than those at the T_1_-optimized structure (Fig. S65†), suggesting that the nonradiative transition from the T_4_ state was fast. Vibronic coupling density (VCD)^33,34^ elucidated that the large VCCs arose from the strong coupling between the electronic state and vibrational modes distributed over the ppy ligand (Fig. S66†). Thus, the low *Φ*_em_ of the solid-state 2a was attributed to T_4_ with large diagonal VCCs.

The charge-by-charge packing structure significantly affected the electronic states of 2a. In 2a·Cl^−^-TBA^+^, the pseudo-degenerate T_1_ (A_g_) and T_2_ (A_u_) as well as T_3_ (A_u_) and T_4_ (A_g_) wavefunctions at the S_0_ optimized structure were distributed over the dipyrrolyldiketone unit, whereas the T_5_ (A_u_) and T_6_ (A_g_) wavefunctions were distributed over the ppy ligand (Fig. S68†). The optimized geometries of T_1_ (A_g_) and T_6_ (A_g_) exhibited the wavefunctions localized on the single molecule by the pseudo-Jahn–Teller distortion (Fig. 9c and S69†). In contrast to the solid-state 2a, the electronic wavefunction was not delocalized over dimer for these states because of the presence of TBA^+^ between 2a·Cl^−^. The TBA^+^ in the charge-by-charge assembly clearly decoupled the electronic interaction, resulting in enhanced phosphorescence derived from the monomeric Pt^II^ complex. Although the *Φ*_em_ values of the ion-pairing assemblies are lower than those in the solution state because of the reduced interactions in the solvated monomeric forms, introduction of aliphatic cations is efficient for inhibiting the self-association and enhancing the phosphorescence properties.

## Conclusions

An ion-pairing strategy has been applied for fabricating solid-state phosphorescent materials by isolating dipyrrolyldiketone Pt^II^ complexes, as emissive anion-responsive molecules, in the form of anion complexes with aliphatic cations. Charge compensation between the anion complexes, in anion-bridged rigid chain structures, and countercations interfered with self-association, resulting in enhanced phosphorescence emission. Solid-state arrangement of the Pt^II^ complexes and their photophysical properties were influenced by the introduced arylpyridine ligands and coexisting cations. Furthermore, the facile recrystallization procedures in the ion-pairing strategy for preparing luminescent materials can be applied for large-scale production. The ion-pairing strategy used in this study does not require the solution-state anion-binding mode. To the best of our knowledge, such a room-temperature phosphorescence enhancement by anion binding and ion-pairing assembly has not been demonstrated thus far. The mechanism of the phosphorescence intensity modulated by anion binding and ion pairing was clearly revealed by theoretical studies for stacking structures. Assembly systems with multiple components, based on the combination of host systems and cationic species, would provide diverse materials with controllable emission wavelengths, quantum yields and lifetimes. Introducing more bulky and robust countercations would further improve the solid-state emission properties. Moreover, introduction of π-electronic cations^35^ would also result in the formation of materials with intriguing photophysical properties. Further studies on ion-pairing luminescent materials are currently being conducted by designing and synthesizing charged building units and precursors (ion-responsive molecules).

## Data availability

Data supporting the work in this publication are available *via* the ESI and associated crystallographic data.†

## Author contributions

H. M. designed and conducted the project. Y. H., K. K., Y. M. and H. T. carried out the synthesis, characterization and property examinations. H. S., H. I. and N. T. evaluated the solid-state absorption and emission spectra. W. O. and T. S. conducted the theoretical calculations. Y. H. and N. Y. analyzed the single-crystal X-ray structures.

## Conflicts of interest

There are no conflicts of interest to declare.

## Supplementary Material

SC-015-D3SC04564A-s001

SC-015-D3SC04564A-s002
