# Supplementary material for: Enhanced solid-state phosphorescence of organoplatinum π-systems by ion-pairing assembly
Source: Chem Sci. 2023 Dec 5;15(3):964–73. doi: 10.1039/d3sc04564a (PMC10793596; doi:10.1039/d3sc04564a)
Supplement: SC-015-D3SC04564A-s001 [file SC-015-D3SC04564A-s001.pdf]

Electronic Supplementary Material (ESI) for Chemical Science.
This journal is © The Royal Society of Chemistry 2023

Electronic Supplementary Information

Enhanced solid-state phosphorescence of organoplatinum π-systems by ion-pairing assembly

Yohei Haketa, Kaifu Komatsu, Hiroi Sei, Hiroki Imoba, Wataru Ota, Tohru Sato, Yu Murakami, Hiroki Tanaka, Nobuhiro Yasuda, Norimitsu Tohnai and Hiromitsu Maeda*

Department of Applied Chemistry, College of Life Sciences, Ritsumeikan University, Kusatsu 525–8577, Japan, Fax: +81 77 561 2659; Tel: +81 77 561 5969; E-mail: maedahir@ph.ritsumei.ac.jp, Department of Applied Chemistry, Graduate School of Engineering, Osaka University, Suita, 565–0871, Japan, MOLFEX, Inc., Kyoto 606–8103, Japan, Fukui Institute for Fundamental Chemistry, Kyoto University, Kyoto 606–8103, Japan, Department of Molecular Engineering, Graduate School of Engineering, Kyoto University, Kyoto 615–8510, Japan and Beamline Division, Japan Synchrotron Radiation Research Institute, Sayo 679–5198, Japan

Table of Contents

1. Synthetic procedures and spectroscopic data S2
Fig. S1–4 ^{1}H and ^{13}C NMR spectra. S4
Fig. S5 UV/vis absorption spectra. S8
2. X-ray crystallographic data S9
Fig. S6–32 Ortep drawings and packing diagrams. S11
Fig. S33–38 Hirshfeld surfaces. S30
3. Theoretical studies S35
Fig. S39–42 Optimized structures. S36
Fig. S43 Electrostatic potential (ESP) mapping. S40
Fig. S44–47 Molecular orbitals (HOMO and LUMO). S41
Fig. S48–55 Theoretical UV/vis absorption spectra. S45
Fig. S56 Theoretical phosphorescence spectra. S46
Fig. S57–60 EDA calculations. S50
Fig. S61–69 Theoretical studies of solid-state structures. S54
Cartesian Coordination of Optimized Structures S58
4. Anion-binding behavior S75
Fig. S70–73 UV/vis absorption spectral changes upon the addition of anions. S75
Fig. S74–77 ^{1}H NMR spectral changes upon the addition of Cl^-. S79
5. Solution-state excited-state properties S83
Fig. S78–81 Emission spectra. S83
Fig. S82 Emission decay profiles. S86
6. Solid-state assembled structures S87
Fig. S83,84 XRD patterns. S88
Fig. S85 Photographs in the solid state. S89
Fig. S86–90 Solid-state emission spectra. S90
Fig. S91–95 Solid-state excitation spectra. S93

1. Synthetic procedure and spectroscopic data

General Information. Starting materials were purchased from FUJIFILM Wako Pure Chemical Corp., Nacalai Tesque Inc., Tokyo Chemical Industry Co., Ltd., and Sigma-Aldrich Co. and were used without further purification unless otherwise stated. (1,3-Dipyrrol-2-yl-1,3-propanedionato-κ*O*[1],κ*O*[3])-[(2-pyridyl-κ*N*)phenyl-κ*C*]platinum **2a** was synthesized according to the literature procedure.[S1] NMR spectra used in the characterization of products were recorded on a JEOL ECA-600 600 MHz spectrometer. All NMR spectra were referenced to solvent. UV-visible absorption spectra were recorded on a Hitachi U-3500 spectrometer. High-resolution (HR) electrospray ionization mass spectrometry (ESI-MS) was recorded on a BRUKER microTOF using ESI-TOF method. TLC analyses were carried out on aluminum sheets coated with silica gel 60 (Merck 5554). Column chromatography was performed on Wakogel C-300.

(1,3-Dipyrrol-2-yl-1,3-propanedionato-κ*O*[1],κ*O*[3])-[4-methoxy-2-(2-pyridyl-κ*N*)phenyl-κ*C*]platinum, 2b. According to the literature procedure,[S1] in a dried Schlenk flask, $[(PtMe_2)_2(SMe_2)_2]$[S2] (60.2 mg, 0.090 mmol) was dissolved in THF (3 mL) under N_2 atmosphere. To the solution was added 2-(3-methoxyphenyl)pyridine (31 μL, 0.18 mmol). The resulting mixture was stirred for 1 h at r.t., and trifluoromethanesulfonic acid (TfOH) (20 μL, 0.18 mmol) was added dropwise. The reaction mixture was stirred for 30 min, and then a solution of 1,3-dipyrrol-2-yl-1,3-propanedione **1**[S3] (36.4 mg, 0.18 mmol) and K_2CO_3 (37.3 mg, 0.27 mmol) in MeOH (3 mL) was added. The mixture was stirred for 3 h. After the removal of the solvent under vacuum, the residue was purified with column chromatography over silica gel (Wakogel C-300; eluent: CH_2Cl_2/*n*-hexane = 3/1 (v/v)) to give **2b** (22.2 mg, 0.038 mmol, 21%) as a yellow solid. R_f = 0.43 (CH_2Cl_2). ^{1}H NMR (600 MHz, $CDCl_3$, 20 °C): δ (ppm) 9.31 (br, 1H, NH), 9.23 (br, 1H, NH), 9.03 (ddd, J = 5.4, 1.8, and 0.6 Hz, 1H, Ar-H), 7.86 (td, J = 7.8 and 1.8 Hz, 1H, Ar-H), 7.63 (d, J = 7.8 Hz, 1H, Ar-H), 7.50 (d, J = 8.4 Hz, 1H, Ar-H), 7.19 (ddd, J = 7.2, 6.0, and 1.8 Hz, 1H, Ar-H), 7.10 (m, 2H, pyrrole-H), 7.08 (d, J = 3.0 Hz, 1H, Ar-H), 6.99 (dd, J = 8.4 and 3.0 Hz, 1H, Ar-H), 6.96 (ddd, J = 3.6, 2.4, and 1.2 Hz, 1H, pyrrole-H), 6.92 (ddd, J = 3.6, 2.4, and 1.2 Hz, 1H, pyrrole-H), 6.46 (s, 1H, CH), 6.35 (ddd, J = 3.6, 3.0, and 2.4 Hz, 1H, pyrrole-H), 6.31 (ddd, J = 3.6, 3.0, and 2.4 Hz, 1H, pyrrole-H), 3.88 (s, 3H, OCH_3). $^{13}C\{^1H\}$ NMR (151 MHz, DMSO-d_6, 20 °C): δ (ppm) 169.95, 168.42, 166.94, 156.73, 148.16, 145.00, 139.15, 131.64, 131.32, 130.58, 128.95, 123.46, 123.11, 122.45, 119.19, 115.65, 112.73, 112.29, 110.13, 109.97, 108.55, 92.90, 55.18. UV/vis (CH_2Cl_2, λ_{max}[nm] (ε, 10^4 $M^{-1}cm^{-1}$)): 368 (3.1). HRMS (ESI-TOF) *m/z*: calcd for $C_{23}H_{18}N_3O_3Pt$ 579.1001 ([M – H]$^-$); found 579.1001. This compound was further characterized by single-crystal X-ray analysis.

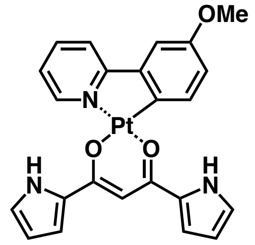

(1,3-Dipyrrol-2-yl-1,3-propanedionato-κ*O*[1],κ*O*[3])-[3,5-difluoro-2-(2-pyridyl-κ*N*)phenyl-κ*C*]platinum, 2c. According to the literature procedure,[S1] in a dried Schlenk flask, $[(PtMe_2)_2(SMe_2)_2]$[S2] (42.6 mg, 0.074 mmol) was dissolved in THF (2.5 mL) under N_2 atmosphere. To the solution was added 2-(2,4-difluorophenyl)pyridine (28.3 mg, 0.15 mmol). The resulting mixture was stirred for 1 h at r.t., and TfOH (16 μL, 0.15 mmol) was added dropwise. The reaction mixture was stirred for 30 min, and then a solution of **1**[S3] (28.3 mg, 0.15 mmol) and K_2CO_3 (30.6 mg, 0.22 mmol) in MeOH (2 mL) was added. The mixture was stirred for 1 h. After the removal of the solvent under vacuum, the residue was purified with column chromatography over silica gel (Wakogel C-300; eluent: CH_2Cl_2/*n*-hexane = 3/1 (v/v)) to give **2c** (23.3 mg, 0.040 mmol, 27%) as a yellow solid. R_f = 0.44 (CH_2Cl_2/*n*-hexane = 3/1 (v/v)). ^{1}H NMR (600 MHz, $CDCl_3$, 20 °C): δ(ppm) 9.25 (br, 1H, NH), 9.19 (br, 1H, NH), 9.03 (dd, J = 6.0 and 1.2 Hz, 1H, Ar-H), 8.01 (d, J = 8.4 Hz, 1H, Ar-H), 7.89 (td, J = 7.8 and 1.2 Hz, 1H, Ar-H), 7.21 (ddd, J = 7.2, 6.0, and 1.8 Hz, 1H, Ar-H), 7.14 (ddd, J = 3.0, 2.4, and 1.2 Hz, 1H, pyrrole-H), 7.11 (ddd, J = 2.4, 2.4, and 1.2 Hz, 1H, Ar-H), 7.05 (dd, J = 8.4 and 2.4 Hz, 1H, Ar-H), 6.96 (ddd, J = 3.6, 2.4, and 1.2 Hz, 1H, pyrrole-H), 6.92 (ddd, J = 3.6, 2.4, and 1.2 Hz, 1H, pyrrole-H), 6.63 (ddd, J = 11.4, 9.0, and 2.4 Hz, 1H, Ar-H), 6.46 (s, 1H, CH), 6.36 (dt, J = 4.2 and 2.4 Hz, 1H, pyrrole-H), 6.33 (dt, J = 3.6 and 2.4 Hz, 1H, pyrrole-H). $^{13}C\{^1H\}$ NMR (151 MHz, DMSO-d_6, 20 °C): δ (ppm) 170.10, 168.84, 163.27, 163.22, 162.63, 162.55, 160.96, 160.87, 160.03, 159.94, 158.32, 158.23, 148.51, 143.95, 143.89, 140.10, 131.11, 130.21, 128.05, 128.03, 123.92, 123.54, 122.66, 122.01, 121.90, 113.33, 112.82, 112.71, 110.31, 110.13, 99.32, 99.14, 98.96, 93.12. UV/vis (CH_2Cl_2, λ_{max}[nm] (ε, 10^4 $M^{-1}cm^{-1}$)): 411 (3.9). HRMS (ESI-TOF) *m/z*: calcd for $C_{22}H_{14}N_3F_2O_2Pt$ 585.0709 ([M – H]$^-$); found 585.0707. This compound was further characterized by single-crystal X-ray analysis.

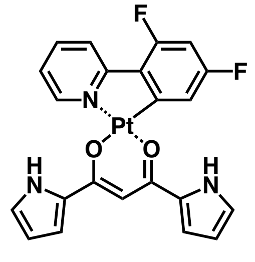

(1,3-Dipyrrol-2-yl-1,3-propanedionato-κ*O*[1],κ*O*[3])-

[benzo-κ*C*[*h*]quinoline-κ*N*]platinum, 2d.** According to the literature procedure,[S1] in a dried Schlenk flask, $[(PtMe_2)_2(SMe_2)_2]$[S2] (74.0 mg, 0.13 mmol) was dissolved in THF (2.5 mL) under N_2 atmosphere. To the solution was added benzo[*h*]quinoline (46.6 mg, 0.26 mmol). The resulting mixture was stirred for 1 h at r.t., and TfOH (28 μL, 0.25 mmol) was added dropwise. The reaction mixture was stirred for 30 min, and then a solution of **1**[S3] (52.5 mg, 0.26 mmol) and K_2CO_3 (53.9 mg, 0.39 mmol) in MeOH (3 mL) was added. The mixture was stirred for 1 h. After the removal of the solvent under vacuum, the residue was purified with column chromatography over silica gel (Wakogel C-300; eluent: CH_2Cl_2/*n*-hexane = 3/1 (v/v)) to give **2d** (17.1 mg, 0.029 mmol, 11%) as an orange solid. R_f = 0.42 (CH_2Cl_2/*n*-hexane = 3/1 (v/v)). ^{1}H NMR (600 MHz, $CDCl_3$, 20 °C): δ (ppm) 9.42 (br, 1H, NH), 9.31 (br, 1H, NH), 9.17 (dd, *J* = 5.4 and 1.2 Hz, 1H, Ar-H), 8.31 (dd, *J* = 7.8 and 1.2 Hz, 1H, Ar-H), 7.80 (d, *J* = 8.4 Hz, 1H, Ar-H), 7.78 (dd, *J* = 4.8 and 3.0 Hz, 1H, Ar-H), 7.65–7.62 (m, 2H, Ar-H), 7.58 (d, *J* = 8.4 Hz, 1H, Ar-H), 7.52 (dd, *J* = 7.8 and 5.4 Hz, 1H, Ar-H), 7.15 (ddd, *J* = 3.0, 2.4, and 1.2 Hz, 1H, pyrrole-H), 7.13 (ddd, *J* = 3.0, 3.0, and 1.2 Hz, 1H, pyrrole-H), 7.00 (ddd, *J* = 3.6, 2.4, and 1.2 Hz, 1H, pyrrole-H), 6.95 (ddd, *J* = 3.6, 3.0, and 1.2 Hz, 1H, pyrrole-H), 6.52 (s, 1H, CH), 6.37 (dt, *J* = 3.6 and 2.4 Hz, 1H, pyrrole-H), 6.34 (dt, *J* = 3.6 and 2.4 Hz, 1H, pyrrole-H). $^{13}C\{^1H\}$ NMR (151 MHz, DMSO-d_6, 20 °C): δ (ppm) 169.92, 168.45, 156.53, 147.72, 141.22, 137.90, 136.30, 132.68, 131.54, 130.48, 128.84, 128.47, 126.10, 123.63, 123.50, 123.30, 121.42, 121.06, 112,92, 112.47, 110.20, 110.02, 92.90. UV/vis (CH_2Cl_2, λ_{max}[nm] (ε, 10^4 $M^{-1}cm^{-1}$)): 368 (3.7). HRMS (ESI-TOF) *m/z*: calcd for $C_{24}H_{16}N_3O_2Pt$ 573.0896 ($[M-H]^-$); found 573.0896. This compound was further characterized by single-crystal X-ray analysis.

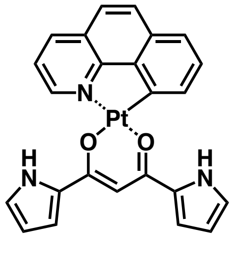

(1,3-Dipyrrol-2-yl-1,3-propanedionato-κ*O*^{1},κ*O*^{3})-[2-(2-pyridyl-κ*N*)benzo[*b*]thiophene-κ*C*]platinum, 2e. According to the literature procedure,[S1] in a dried Schlenk flask, $[(PtMe_2)_2(SMe_2)_2]$[S2] (39.1 mg, 0.068 mmol) was dissolved in THF (2 mL) under N_2 atmosphere. To the solution was added 2-(2-pyridyl)benzo[*b*]thiophene (25.4 mg, 0.12 mmol). The resulting mixture was stirred for 1.5 h at r.t., and TfOH (13 μL, 0.12 mmol) was added dropwise. The reaction mixture was stirred for 30 min, and then a solution of **1**[S3] (24.3 mg, 0.12 mmol) and K_2CO_3 (24.8 mg, 0.18 mmol) in MeOH (2 mL) was added. The mixture was stirred for 5 h. After the removal of the solvent under vacuum, the residue was purified with column chromatography over silica gel (Wakogel C-300; eluent: CH_2Cl_2/*n*-hexane = 3/1 (v/v)) to give **2e** (5.1 mg, 0.0084 mmol, 7.0%) as a yellow solid. R_f = 0.33 (CH_2Cl_2/*n*-hexane = 3/1 (v/v)). ^{1}H NMR (600 MHz, $CDCl_3$, 20 °C): δ (ppm) 9.33 (br, 1H, NH), 9.20 (br, 1H, NH), 8.94 (d, *J* = 4.8 Hz, 1H, Ar-H), 8.80 (d, *J* = 7.2 Hz, 1H, Ar-H), 7.87 (dd, *J* = 6.6 and 1.8 Hz, 1H, Ar-H), 7.78 (td, *J* = 7.8 and 1.8 Hz, 1H, Ar-H), 7.41 (td, *J* = 6.6 and 1.8 Hz, 1H, Ar-H), 7.38 (td, *J* = 6.6 and 1.8 Hz, 1H, Ar-H), 7.36 (d, *J* = 7.2 Hz, 1H, Ar-H), 7.12 (ddd, *J* = 2.4, 2.4, and 1.2 Hz, 1H, pyrrole-H), 7.04 (dd, *J* = 7.2 and 1.2 Hz, 1H, Ar-H), 6.99 (ddd, *J* = 3.6, 2.4, and 1.2 Hz, 1H, pyrrole-H), 6.97 (ddd, *J* = 3.6, 2.4, and 1.2 Hz, 1H, pyrrole-H), 6.52 (s, 1H, CH), 6.37 (dt, *J* = 3.6 and 2.4 Hz, 1H, pyrrole-H), 6.35 (dt, *J* = 3.6 and 2.4 Hz, 1H, pyrrole-H). $^{13}C\{^1H\}$ NMR (151 MHz, DMSO-d_6, 20 °C): δ (ppm) 169.75, 168.26, 162.85, 148.18, 144.80, 141.35, 140.14, 138.20, 137.67, 131.05, 130.66, 126.21, 125.52, 124.13, 123.96, 122.95, 122.81, 120.35, 118.50, 113.46, 112.60, 110.37, 110.31, 93.23. UV/vis (CH_2Cl_2, λ_{max}[nm] (ε, 10^4 $M^{-1}cm^{-1}$)): 388 (3.1). HRMS (ESI-TOF) *m/z*: calcd for $C_{24}H_{16}N_3O_2PtS$ 605.0617 ($[M-H]^-$); found 605.0618. This compound was further characterized by single-crystal X-ray analysis.

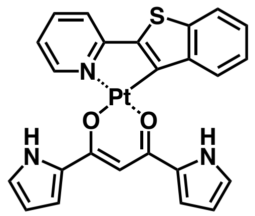

[S1] A. Kuno, G. Hirata, H. Tanaka, Y. Kobayashi, N. Yasuda and H. Maeda, *Chem. Eur. J.*, 2021, **27**, 10068–10076.

[S2] G. S. Hill, M. J. Irwin, C. J. Levy, L. M. Rendina and R. J. Puddephatt, *Inorg. Synth.*, 1998, **32**, 149–151.

[S3] H. Meada and Y. Kusunose, *Chem. Eur. J.*, 2005, **11**, 5661–5666.

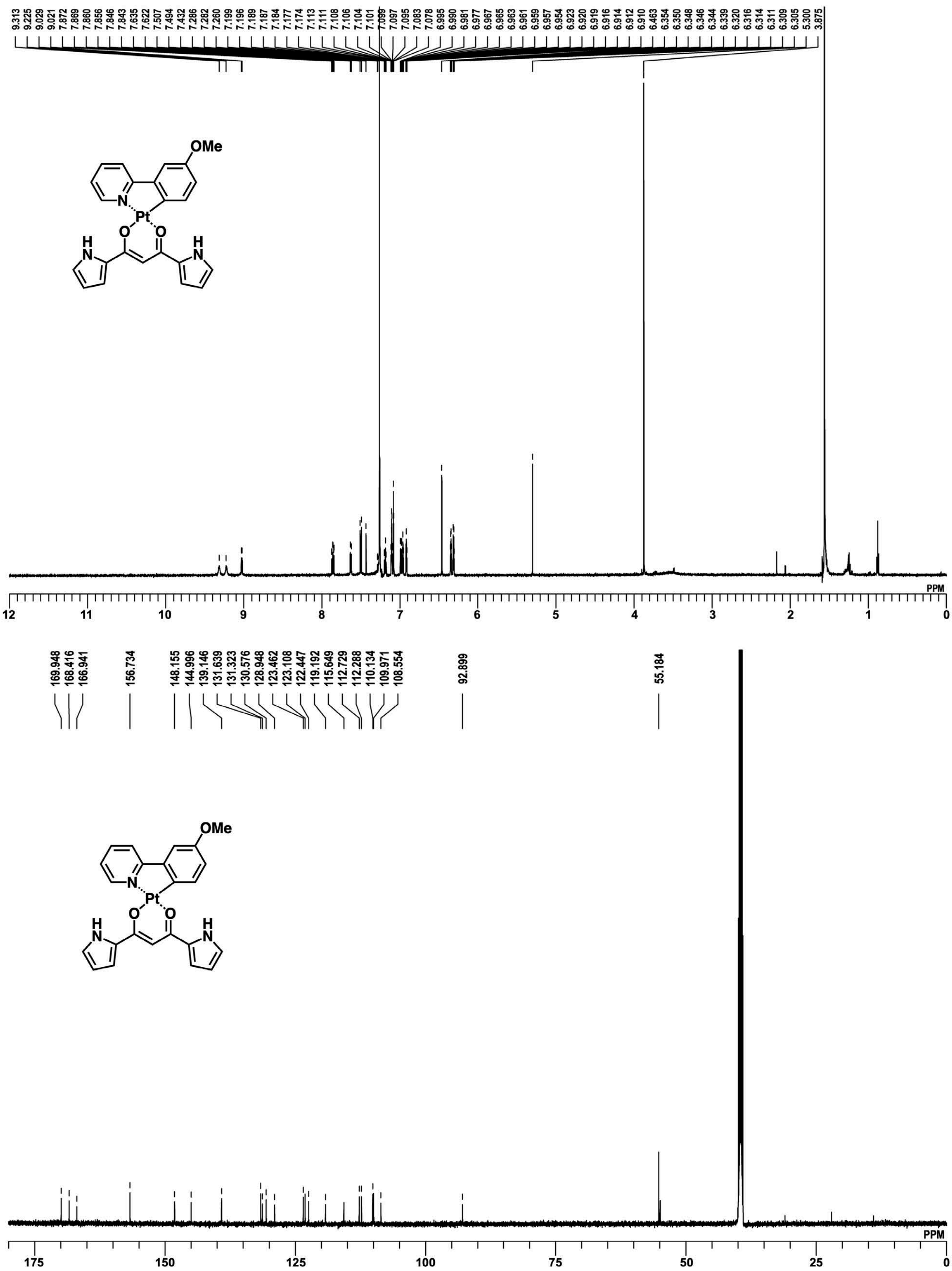

Fig. S1 ^{1}H NMR (top) and ^{13}C NMR (bottom) spectra of **2b** in $CDCl_3$ and DMSO-d_6, respectively.

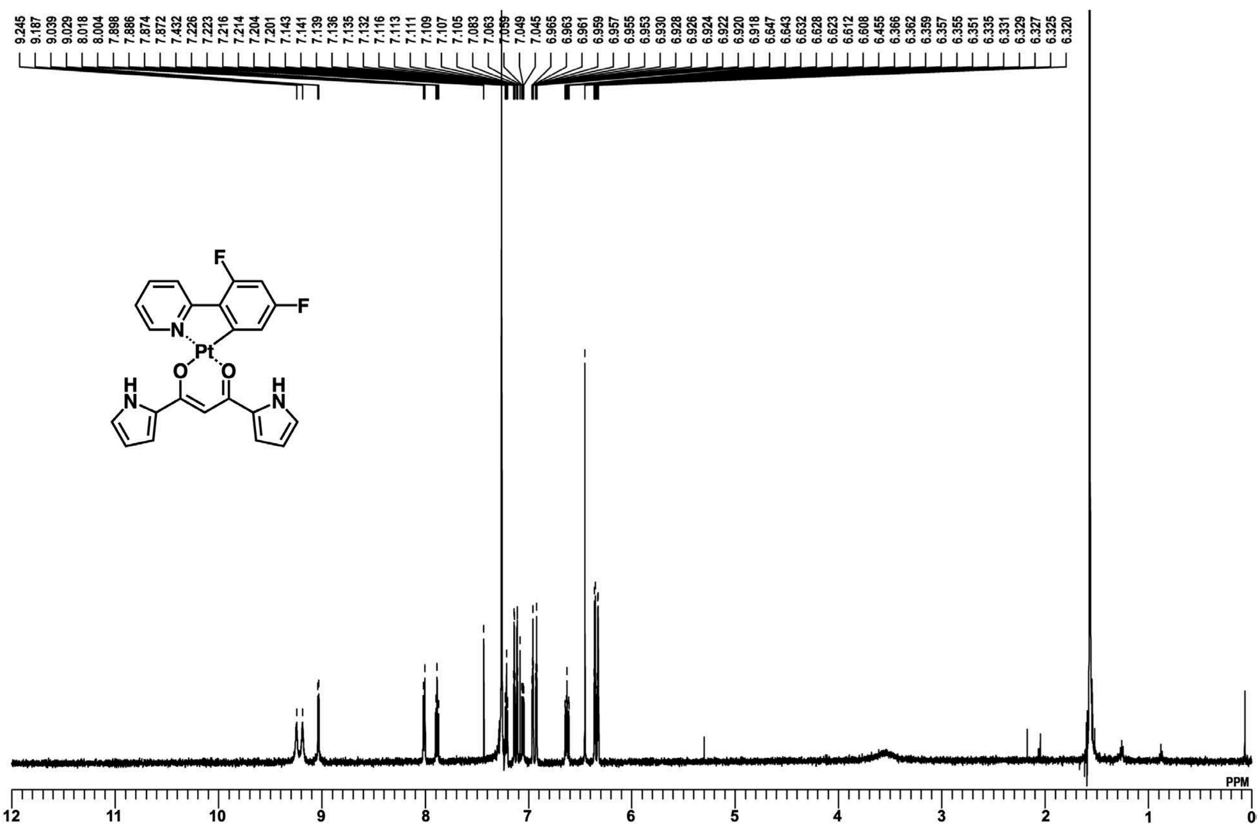

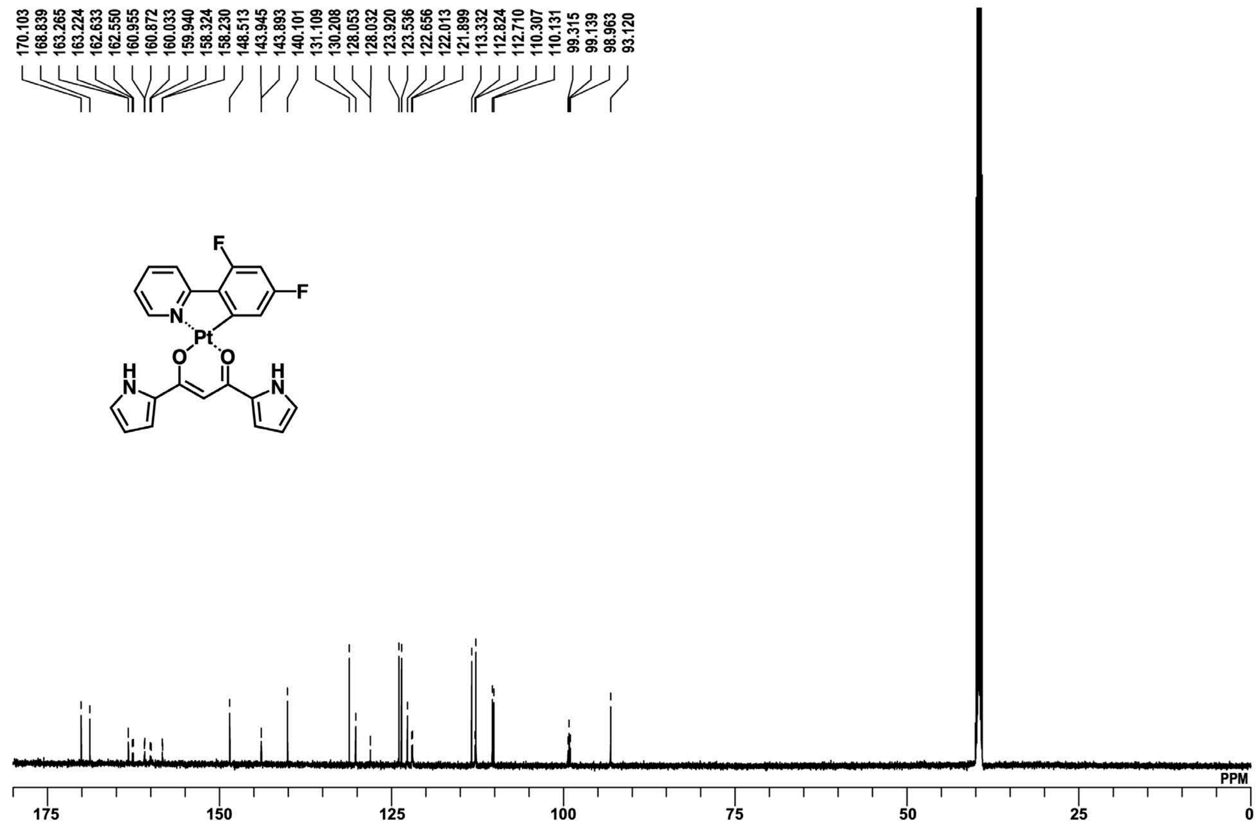

Fig. S2 ^{1}H NMR (top) and ^{13}C NMR (bottom) spectra of **2c** in $CDCl_3$ and DMSO-d_6, respectively.

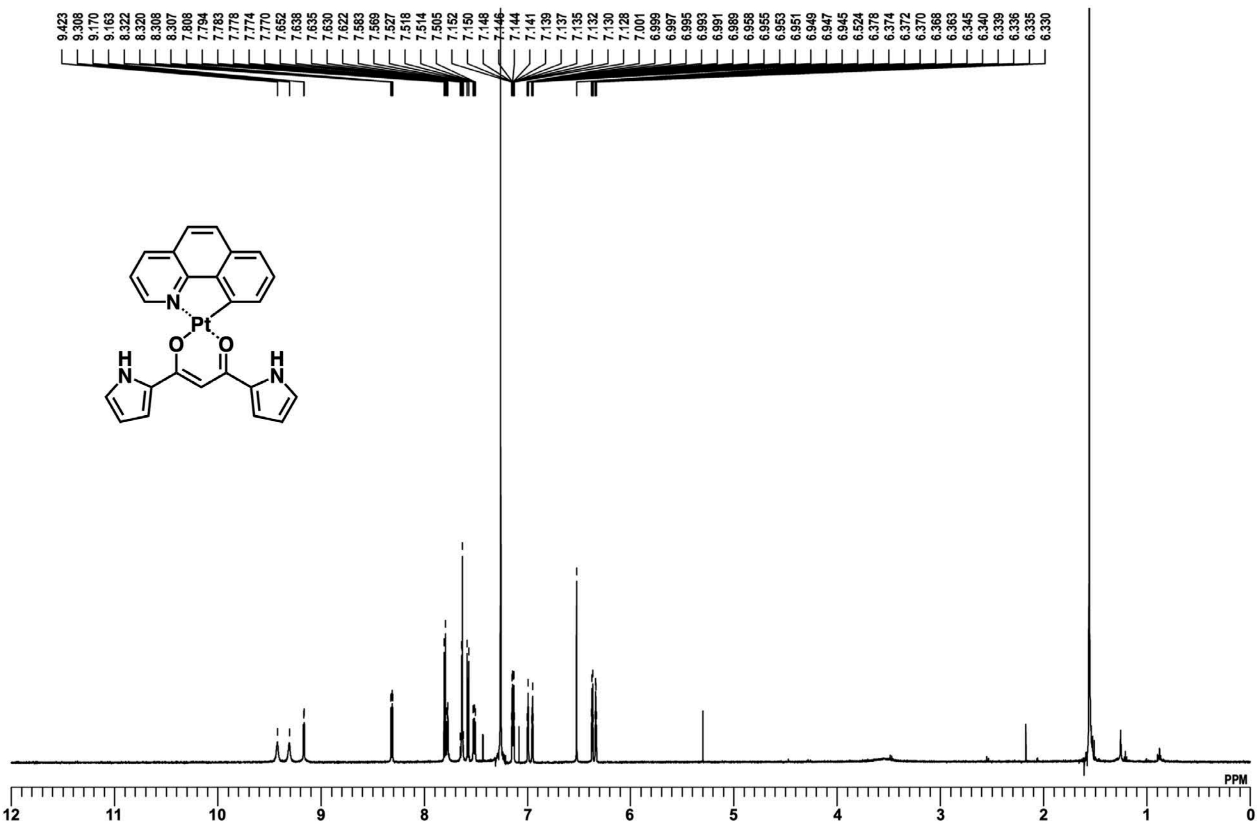

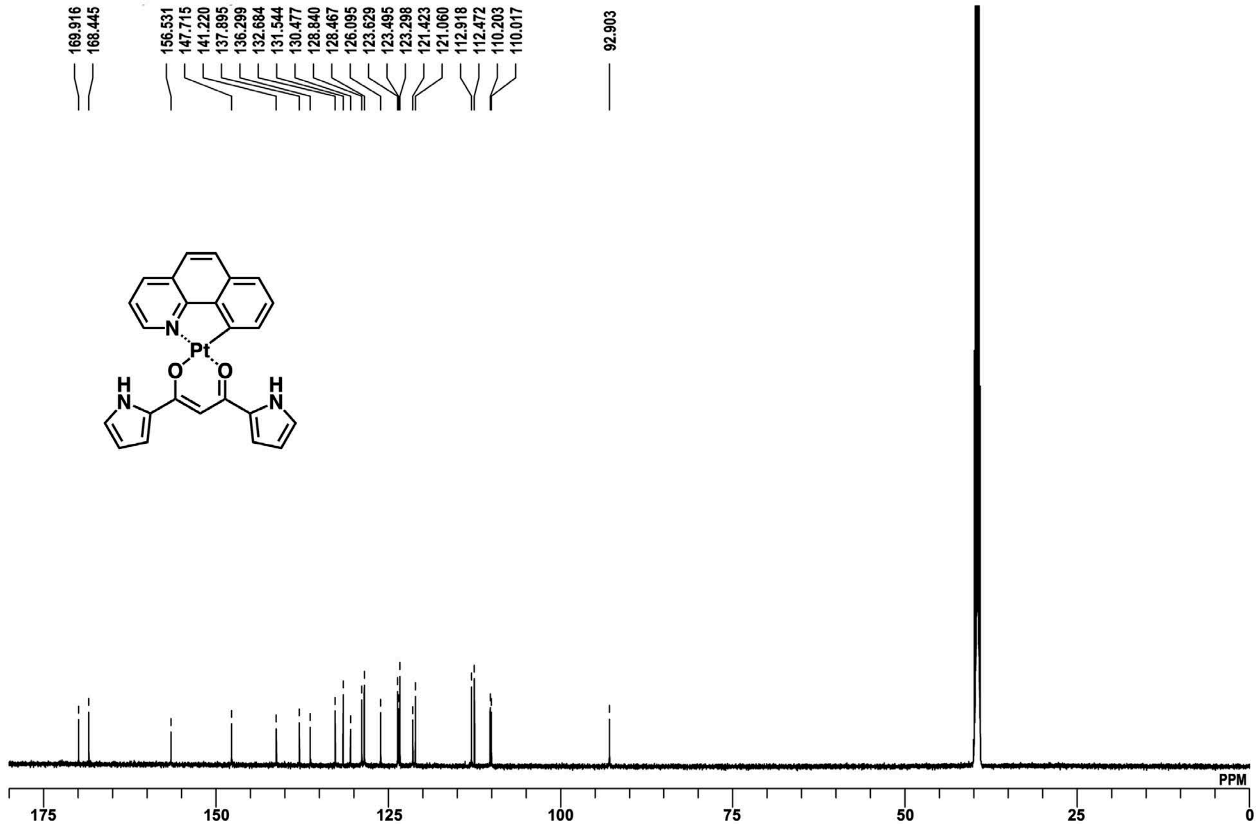

Fig. S3 ^{1}H NMR (top) and ^{13}C NMR (bottom) spectra of **2d** in $CDCl_3$ and DMSO-d_6, respectively.

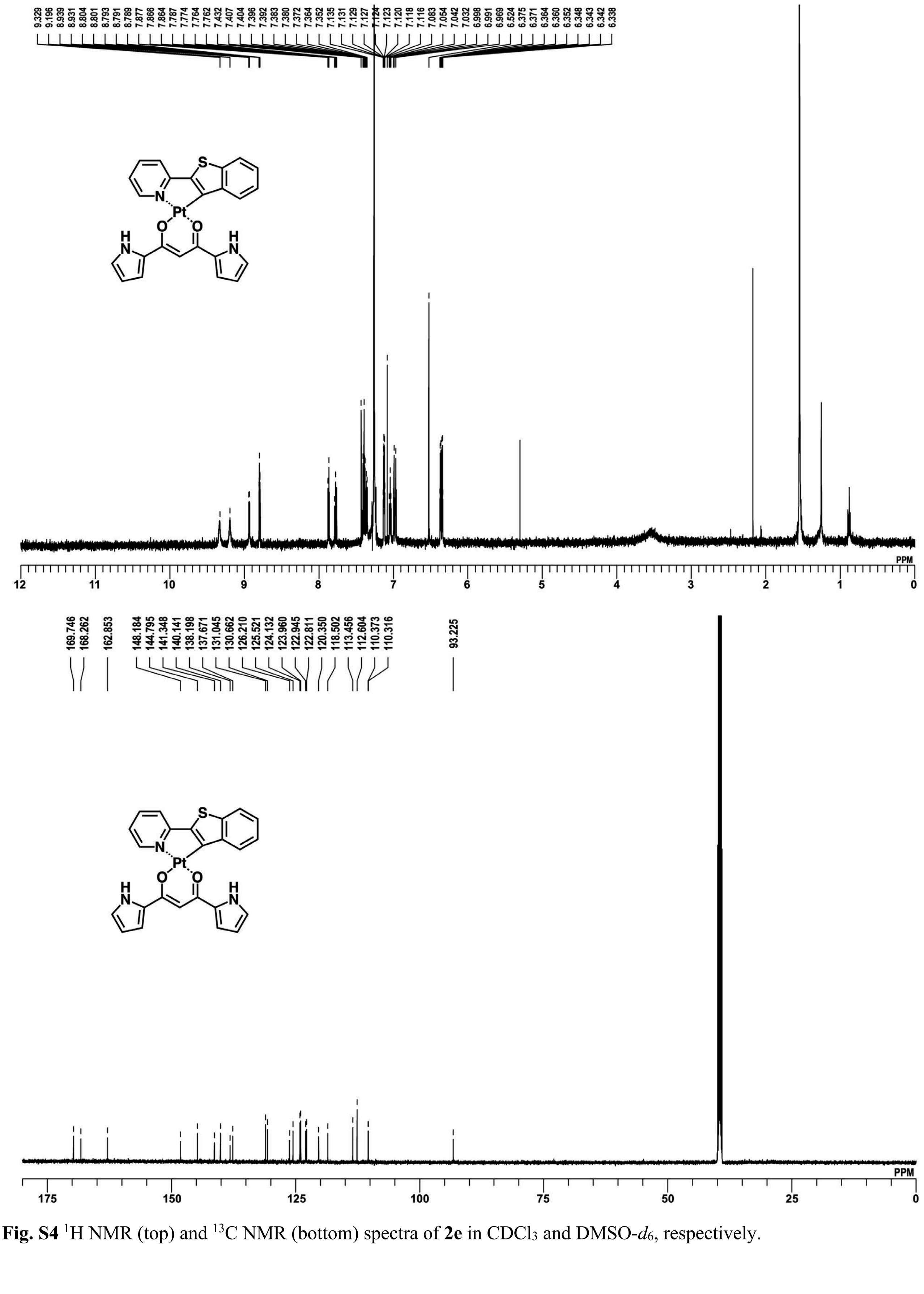

Fig. S4 ^{1}H NMR (top) and ^{13}C NMR (bottom) spectra of **2e** in $CDCl_3$ and DMSO-d_6, respectively.

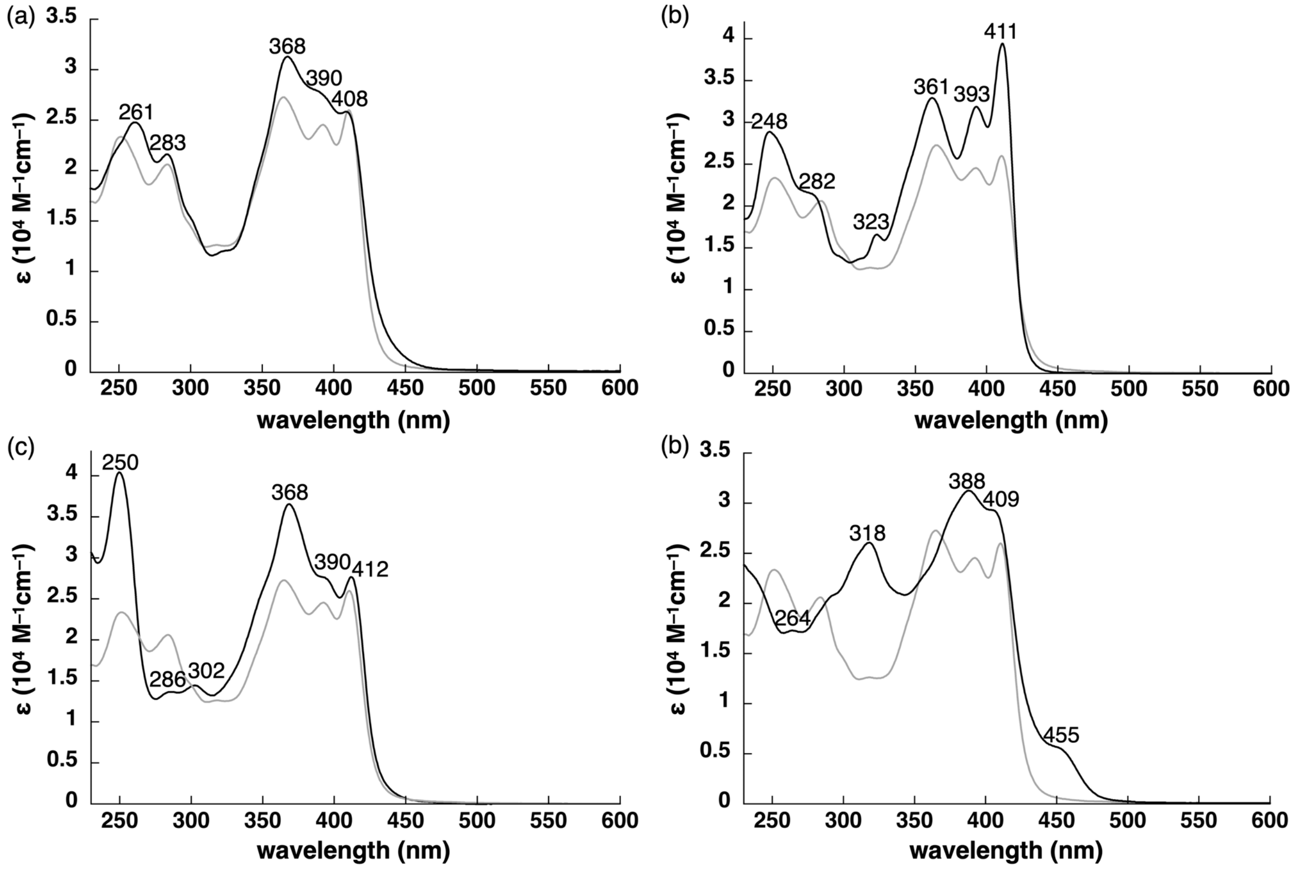

Fig. S5 UV/vis absorption spectra of (a) **2b**, (b) **2c**, (c) **2d**, and (d) **2e** in CH_2Cl_2 (0.03 mM) with that of **2a** (gray) as a reference.

2. X-ray crystallographic data

Method for single-crystal X-ray analysis. Crystallographic data are summarized in Table S1. A single crystal of **2b** was obtained by vapor diffusion of *n*-hexane into a CH_2Cl_2 solution. The data crystal was a yellow block of approximate dimensions 0.200 mm × 0.140 mm × 0.010 mm. A single crystal of **2c** was obtained by vapor diffusion of *n*-hexane into a CH_2Cl_2 solution. The data crystal was a yellow block of approximate dimensions 0.300 mm × 0.053 mm × 0.040 mm. A single crystal of **2d** was obtained by vapor diffusion of *n*-hexane into a CH_2Cl_2 solution. The data crystal was a yellow block of approximate dimensions 0.300 mm × 0.100 mm × 0.100 mm. A single crystal of **2e** was obtained by vapor diffusion of *n*-hexane into a CH_2Cl_2 solution. The data crystal was a yellow block of approximate dimensions 0.110 mm × 0.090 mm × 0.020 mm. A single crystal of **2a**·Cl^--TBA^+ was obtained by vapor diffusion of *n*-hexane into a THF solution of the 1:1 mixture of **2a**[S1] and tetrabutylammonium chloride (TBACl). The data crystal was a yellow plate of approximate dimensions 0.10 mm × 0.05 mm × 0.01 mm. A single crystal of **2a**·Cl^--$TPeA^+$ was obtained by vapor diffusion of *n*-hexane into an acetone solution of the 1:1 mixture of **2a** and tetrapentylammonium chloride (TPeACl). The data crystal was a yellow prism of approximate dimensions 0.50 mm × 0.20 mm × 0.10 mm. A single crystal of **2b**$_4$·Cl^--TBA^+ was obtained by vapor diffusion of *n*-hexane into a $CHCl_3$ solution of the 1:1 mixture of **2b** and TBACl. The data crystal was a yellow block of approximate dimensions 0.03 mm × 0.03 mm × 0.03 mm. A single crystal of **2b**$_4$·Cl^--$TPeA^+$ was obtained by vapor diffusion of *n*-hexane into a $CHCl_3$ solution of the 1:1 mixture of **2b** and TPeACl. The data crystal was a yellow plate of approximate dimensions 0.10 mm × 0.02 mm × 0.02 mm. The data crystal was a yellow plate of approximate dimensions 0.13 mm × 0.10 mm × 0.01 mm. A single crystal of **2c**·Cl^--TBA^+ was obtained by vapor diffusion of *n*-hexane into a 1,2-dichloroethane solution of the 1:1 mixture of **2c** and TBACl. The data crystal was a yellow block of approximate dimensions 0.08 mm × 0.05 mm × 0.02 mm. A single crystal of **2c**·Cl^--$TPeA^+$ was obtained by vapor diffusion of *n*-hexane into a 1,2-dichloroethane solution of the 1:1 mixture of **2c** and TPeACl. The data crystal was a yellow prism of approximate dimensions 0.05 mm × 0.02 mm × 0.02 mm. A single crystal of **2d**·Cl^--TBA^+_M was obtained by vapor diffusion of *n*-hexane into a CH_2Cl_2 solution of the 1:1 mixture of **2d** and TBACl. The data crystal was an orange block of approximate dimensions 0.03 mm × 0.03 mm × 0.01 mm. A single crystal of **2d**·Cl^--TBA^+_E was obtained by vapor diffusion of *n*-hexane into a 1,2-dichloroethane solution of the 1:1 mixture of **2d** and TBACl. The data crystal was a yellow block of approximate dimensions 0.20 mm × 0.05 mm × 0.02 mm. A single crystal of **2d**$_2$·Cl^--$TPPAu^+$ was obtained by vapor diffusion of *n*-hexane into a CH_2Cl_2 solution of the 1:1 mixture of **2d** and tetraphenylporphyrin Au^{III} complex ($TPPAu^+$) as a Cl^- ion pair.[S4] The data crystal was a red block of approximate dimensions 0.040 mm × 0.020 mm × 0.010 mm. The data of **2b** and **2d**$_2$·Cl^--$TPPAu^+$ were collected at 93 K on a Rigaku XtaLAB P200 diffractometer with graphite monochromated Cu-Kα radiation (λ = 1.54184 Å), whereas those of **2c**–**e** were collected at 100 K on a Rigaku Pliatus 3 CdTe 1M with Si (311) monochromated synchrotron radiation (λ = 0.4119 (**2c**,**d**) and 0.4118 Å (**2e**)) at BL02B1 (SPring-8).[S5] The data of **2a**·Cl^--TBA^+, **2b**$_4$·Cl^--TBA^+, **2b**$_4$·Cl^--$TPeA^+$, **2c**·Cl^--TBA^+, **2c**·Cl^--$TPeA^+$, and **2d**·Cl^--TBA^+_M were collected at 90 K on a DECTRIS EIGER X 1M diffractometer with Si(111) monochromated synchrotron radiation (λ = 0.81082 (**2a**·Cl^--TBA^+, **2b**$_4$·Cl^--TBA^+, **2b**$_4$·Cl^--$TPeA^+$, **2c**·Cl^--TBA^+, and **2c**·Cl^--$TPeA^+$) and 0.81106 Å (**2d**·Cl^--TBA^+_M)) at BL40XU (SPring-8).[S6] The data of **2a**·Cl^--$TPeA^+$ and **2d**·Cl^--TBA^+_E were collected at 90 K on a Bruker D8 Venture diffractometer with MoKα radiation (λ = 0.71073 Å) focused by multilayer confocal mirror. All the structures were solved by dual-space method. The structures were refined by a full-matrix least-squares method by using a SHELXL 2014[S7] (Yadokari-XG).[S8] In each structure, the non-hydrogen atoms were refined anisotropically. For **2b**$_4$·Cl^--TBA^+, the disordered solvents, presumably $CHCl_3$, were removed using the SQUEEZE protocol included in PLATON.[S9] CIF files (CCDC-2158662–2158674) can be obtained free of charge from the Cambridge Crystallographic Data Centre via www.ccdc.cam.ac.uk/data_request/cif.

Table S1 Crystallographic details.

	**2b**	**2c**	**2d**	**2e**	**2a**·Cl^--TBA^+
formula	$C_{23}H_{19}N_3O_3Pt·CH_2Cl_2$	$C_{22}H_{15}F_2N_3O_2Pt$	$C_{24}H_{17}N_3O_2Pt·0.5H_2O$	$C_{24}H_{17}N_3O_2PtS$	$C_{22}H_{17}N_3O_2PtCl·$ $C_{16}H_{36}N·C_4H_8O$
fw	665.43	586.46	583.50	606.55	900.48
crystal size, mm	0.200 × 0.140 × 0.010	0.300 × 0.053 × 0.040	0.300 × 0.100 × 0.100	0.110 × 0.090 × 0.020	0.10 × 0.05 × 0.01
crystal system	monoclinic	monoclinic	monoclinic	monoclinic	monoclinic
space group	$P2_1/n$ (no. 14)	$P2_1/n$ (no. 14)	$P2_1/c$ (no. 14)	$P2_1/c$ (no. 14)	$P2_1/c$ (no. 14)
a, Å	21.7245(4)	12.139(2)	29.7626(16)	12.577(13)	8.5147(2)
b, Å	8.15100(10)	6.8997(13)	5.0950(3)	8.202(5)	16.3288(4)
c, Å	27.6097(5)	22.664(4)	25.3909(13)	20.223(12)	28.9929(8)
α, °	90	90	90	90	90
β, °	108.074(2)	104.348(7)	98.831(7)	103.10(2)	97.377(2)
γ, °	90	90	90	90	90
V, Å^3	4647.79(14)	1839.1(6)	3804.6(4)	2032(3)	3997.66(18)
ρ_{calcd}, gcm^{-3}	1.902	2.118	2.037	1.983	1.496
Z	8	4	8	4	3
T, K	93(2)	100(2)	100(2)	100(2)	90(2)
μ, mm^{-1}	13.681 ^{a}	1.743 ^{b}	1.683 ^{b}	1.709 ^{b}	5.031 ^{b}
no. of reflns	56421	51901	107691	42191	41868
no. of unique reflns	8254	4203	8758	4584	7323
variables	597	271	553	280	464
λ, Å	1.54184 ^{a}	0.4119 ^{b}	0.4119 ^{b}	0.4118 ^{b}	0.81082 ^{b}
R_1 ($I > 2\sigma(I)$)	0.0659	0.0168	0.0198	0.0244	0.0805
wR_2 ($I > 2\sigma(I)$)	0.1690	0.0457	0.0517	0.0653	0.1908
GOF	1.038	1.063	1.114	1.047	1.075

	**2a**·Cl^--$TPeA^+$	**2b**$_4$·Cl^--TBA^+	**2b**$_4$·Cl^--$TPeA^+$	**2c**·Cl^--TBA^+	**2c**·Cl^--$TPeA^+$
formula	$C_{22}H_{17}N_3O_2PtCl·$ $C_{20}H_{44}N$	$4(C_{23}H_{19}N_3O_3Pt)Cl·$ $C_{16}H_{36}N·4CHCl_3$	$4(C_{23}H_{19}N_3O_3Pt)Cl·$ $C_{20}H_{44}N·2CHCl_3$	$C_{22}H_{15}F_2N_3O_2PtCl·$ $C_{16}H_{36}N·0.836C_2H_4Cl_2$	$C_{22}H_{15}F_2N_3O_2PtCl·$ $C_{20}H_{44}N·0.475C_2H_4Cl_2$
fw	884.48	3077.38	2894.75	947.05	967.41
crystal size, mm	0.50 × 0.20 × 0.10	0.03 × 0.03 × 0.03	0.10 × 0.02 × 0.02	0.08 × 0.05 × 0.02	0.05 × 0.02 × 0.02
crystal system	orthorhombic	tetragonal	monoclinic	monoclinic	monoclinic
space group	$Pca2_1$ (no. 29)	$P4ncc$ (no. 130)	$C2/c$ (no. 15)	$P2_1$ (no. 4)	$C2/c$ (no. 15)
a, Å	21.437(3)	22.024(2)	20.783(5)	16.8798(14)	47.3103(11)
b, Å	8.3346(12)	22.024(2)	23.203(6)	16.7488(14)	8.5434(2)
c, Å	22.655(4)	21.509(2)	22.652(5)	29.324(3)	21.0894(5)
α, °	90	90	90	90	90
β, °	90	90	90.778(4)	98.644(2)	96.468(2)
γ, °	90	90	90	90	90
V, Å^3	4047.7(11)	10434(2)	10922(5)	8196.2(12)	8469.9(3)
ρ_{calcd}, gcm^{-3}	1.451	1.959	1.760	1.535	1.517
Z	4	4	4	8	8
T, K	90(2)	90(2)	90(2)	90(2)	90(2)
μ, mm^{-1}	3.572 ^{c}	8.010 ^{b}	7.438 ^{b}	5.074 ^{b}	4.848 ^{b}
no. of reflns	36622	104380	58189	102897	43928
no. of unique reflns	14700	4784	9992	34372	7740
variables	456	309	676	1888	493
λ, Å	0.71073 ^{c}	0.81082 ^{b}	0.81082 ^{b}	0.81082 ^{b}	0.81082 ^{b}
R_1 ($I > 2\sigma(I)$)	0.0528	0.1029	0.0739	0.0593	0.0505
wR_2 ($I > 2\sigma(I)$)	0.1375	0.2296	0.1995	0.1523	0.1285
GOF	1.004	1.173	1.075	1.065	1.117

^{a} Cu-Kα radiation. ^{b} Synchrotron radiation. ^{c} Mo-Kα radiation.

Table S1 (Continued)

	**2d**·Cl^--TBA^+_M	**2d**·Cl^--TBA^+_E	**2d**$_2$·Cl^--$TPPAu^+$
formula	$C_{24}H_{17}N_3O_2PtCl$· $C_{16}H_{36}N$·CH_2Cl_2	$C_{24}H_{17}N_3O_2PtCl$· $C_{16}H_{36}N$·CH_2ClCH_2Cl	$2(C_{24}H_{17}N_3O_2Pt)Cl$· $C_{44}H_{28}AuN_4$·$4CH_2Cl_2$
fw	937.33	951.35	2333.81
crystal size, mm	0.03 × 0.03 × 0.01	0.20 × 0.05 × 0.02	0.040 × 0.020 × 0.010
crystal system	orthorhombic	orthorhombic	triclinic
space group	$Pca2_1$ (no. 29)	*Pbca* (no. 61)	*P*-1 (no. 2)
a, Å	28.1013(6)	17.716(8)	13.5379(2)
b, Å	8.2546(2)	17.001(7)	18.5737(3)
c, Å	17.2008(4)	27.332(12)	19.8643(3)
α, °	90	90	62.469(2)
β, °	90	90	72.9050(10)
γ, °	90	90	86.3260(10)
V, Å^3	3989.98(16)	8233(6)	4218.34(13)
ρ_{calcd}, gcm^{-3}	1.560	1.535	1.837
Z	4	8	2
T, K	90(2)	90(2)	93(2)
μ, mm^{-1}	5.235 [b]	3.644 [c]	12.397 [a]
no. of reflns	40495	48242	55062
no. of unique reflns	7272	6981	14533
variables	458	492	1186
λ, Å	0.81106 [b]	0.71073 [c]	1.54184 [a]
R_1 ($I > 2\sigma(I)$)	0.0466	0.0964	0.0385
wR_2 ($I > 2\sigma(I)$)	0.1060	0.2422	0.1099
GOF	1.144	1.028	1.006

[a] Cu-Kα radiation. [b] Synchrotron radiation. [c] Mo-Kα radiation.

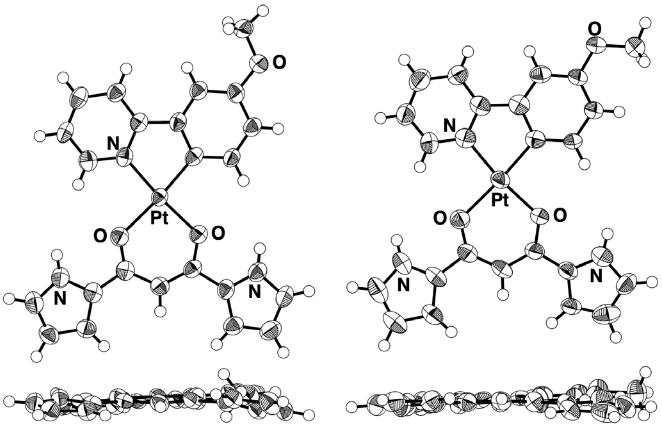

Fig. S6 Ortep drawings of single-crystal X-ray structures (top and side views) of **2b** with two independent structures. Thermal ellipsoids are scaled to the 50% probability level. Solvent molecules are omitted for clarity.

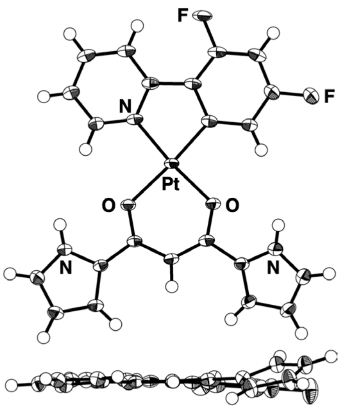

Fig. S7 Ortep drawings of single-crystal X-ray structure (top and side views) of **2c**. Thermal ellipsoids are scaled to the 50% probability level.

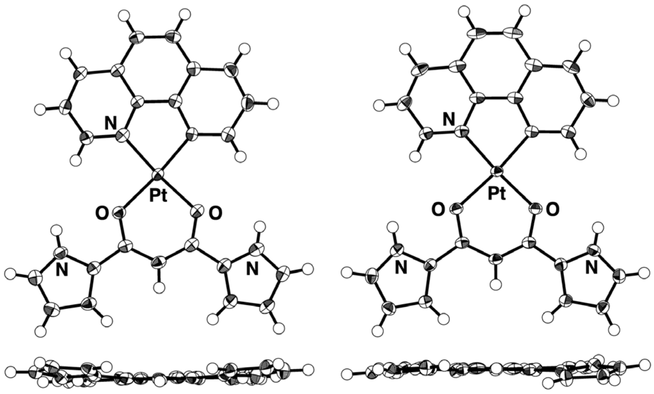

Fig. S8 Ortep drawings of single-crystal X-ray structures (top and side views) of **2d** (two independent structures). Thermal ellipsoids are scaled to the 50% probability level. Solvent molecules are omitted for clarity.

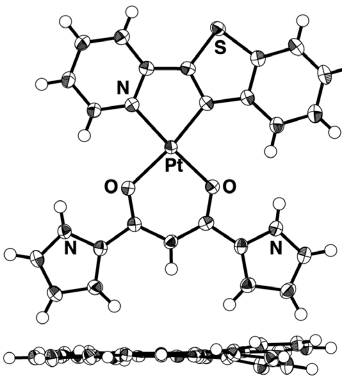

Fig. S9 Ortep drawings of single-crystal X-ray structure (top and side views) of **2e**. Thermal ellipsoids are scaled to the 50% probability level.

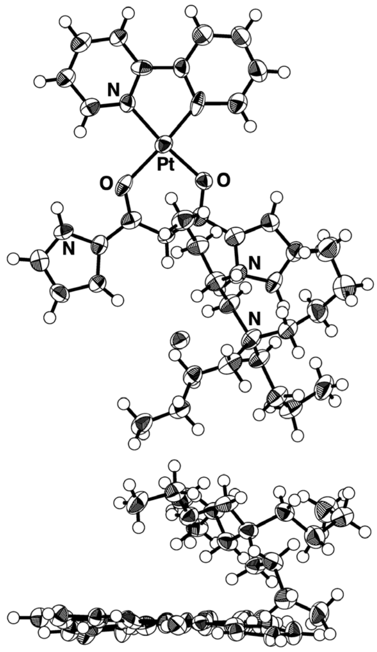

Fig. S10 Ortep drawings of single-crystal X-ray structures (top and side views) of **2a**·Cl^{-}-TBA^{+}. Thermal ellipsoids are scaled to the 50% probability level. Solvent molecules are omitted for clarity.

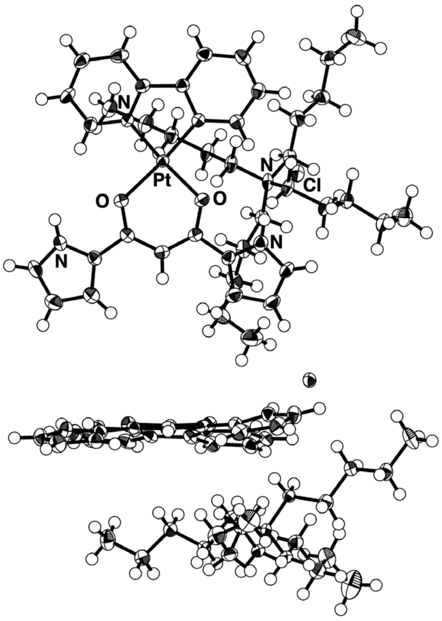

Fig. S11 Ortep drawings of single-crystal X-ray structures (top and side views) of **2a**$\cdot Cl^-$-TPeA$^+$. Thermal ellipsoids are scaled to the 50% probability level.

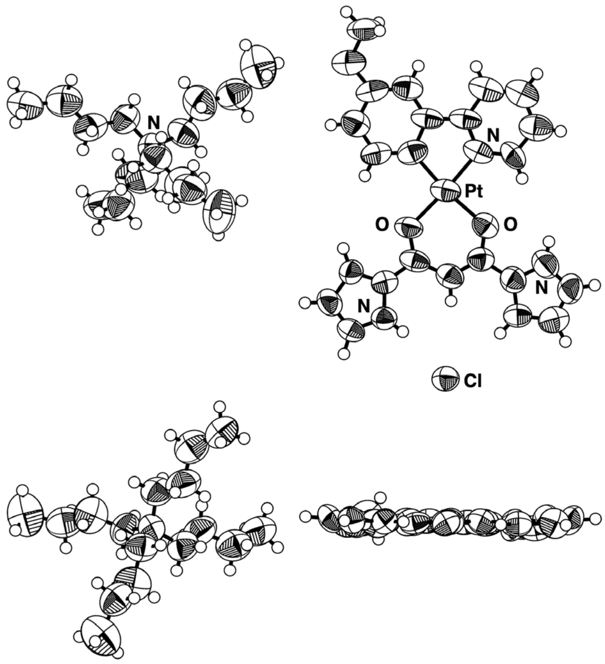

Fig. S12 Ortep drawings of single-crystal X-ray structures (top and side views) of **2b**$_4\cdot Cl^-$-TBA$^+$. Thermal ellipsoids are scaled to the 50% probability level. Four **2b** units in **2b**$_4\cdot Cl^-$ are equivalent and, thus, only one structure is shown. Solvent molecules are omitted for clarity.

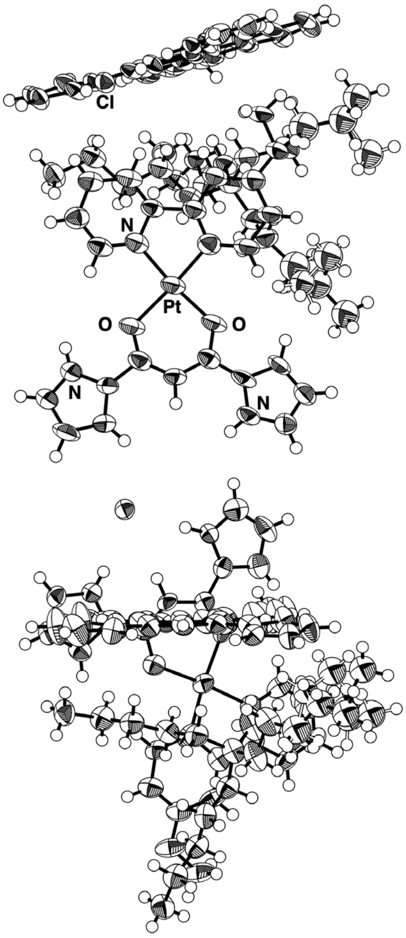

Fig. S13 Ortep drawings of single-crystal X-ray structures (top and side views) of **2b**$_4$·Cl$^-$-TPeA$^+$. Thermal ellipsoids are scaled to the 50% probability level. Four **2b** units in **2b**$_4$·Cl$^-$ are equivalent and, thus, only one structure is shown. The disordered structures in the ratio of 0.60:0.40 (a butyl unit in TBA$^+$) are represented by black and white bonds for major and minor structures, respectively. Solvent molecules are omitted for clarity.

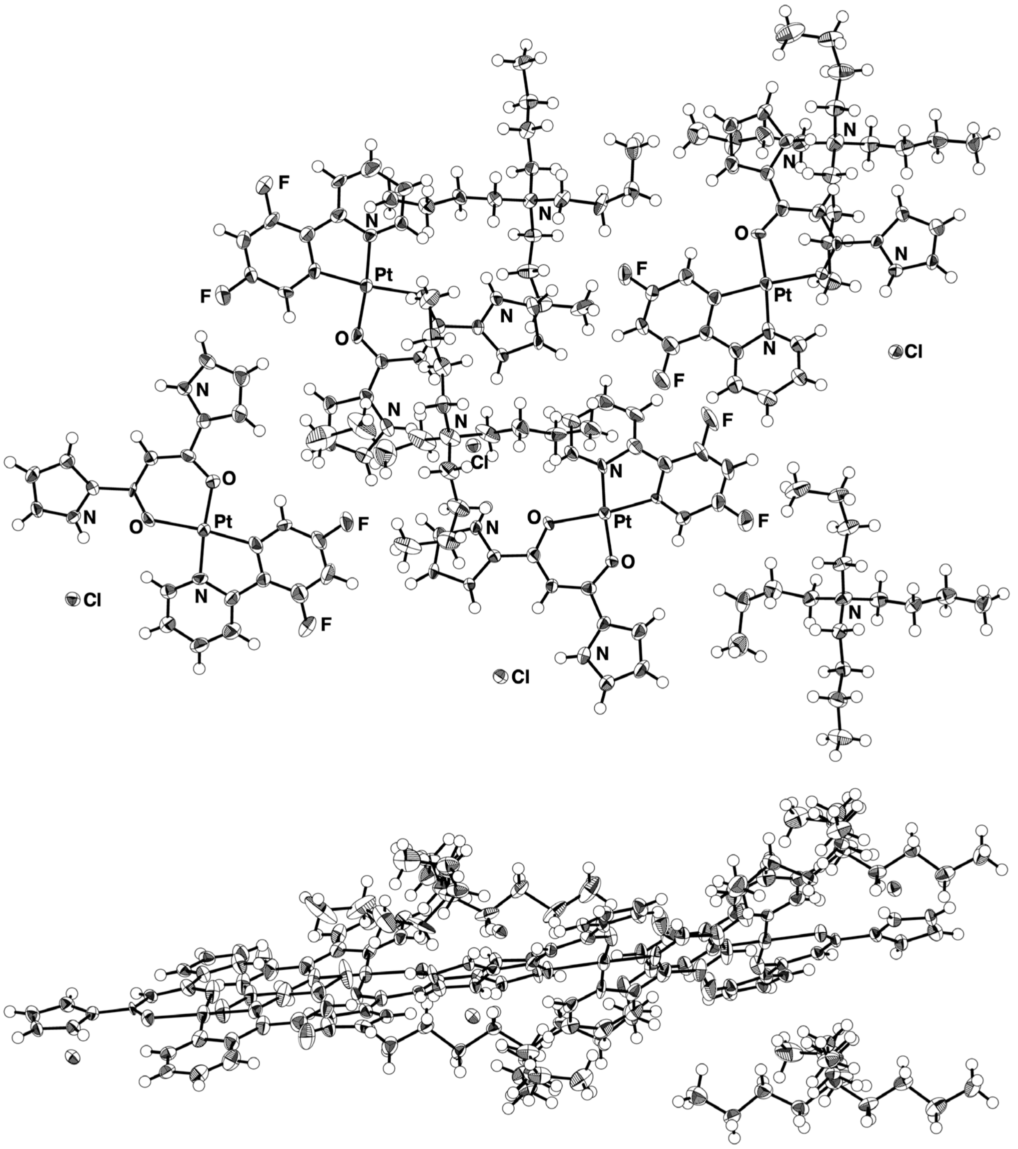

Fig. S14 Ortep drawings of single-crystal X-ray structures (top and side views) of **2c**·Cl^{-}-TBA^{+}. Thermal ellipsoids are scaled to the 50% probability level. Solvent molecules are omitted for clarity.

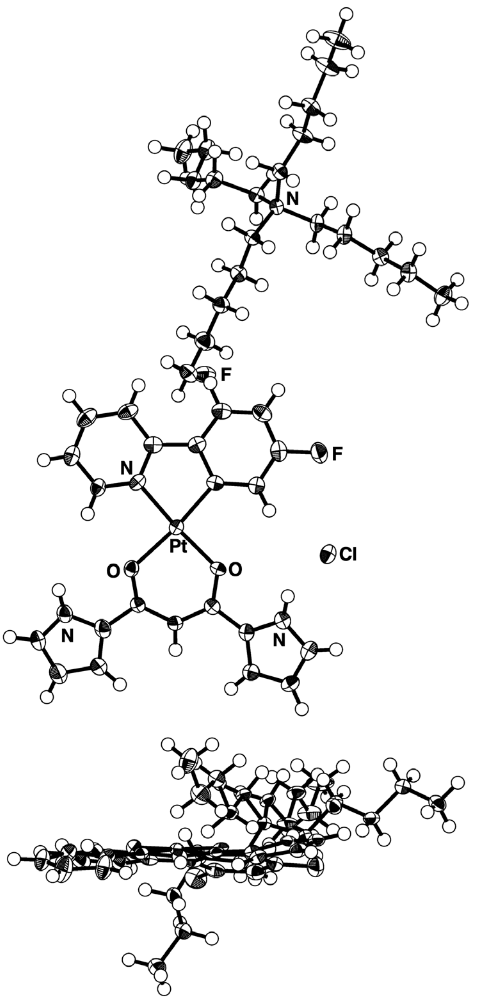

Fig. S15 Ortep drawings of single-crystal X-ray structures (top and side views) of **2c**·Cl^{-}-$TPeA^{+}$. Thermal ellipsoids are scaled to the 50% probability level. Solvent molecules are omitted for clarity.

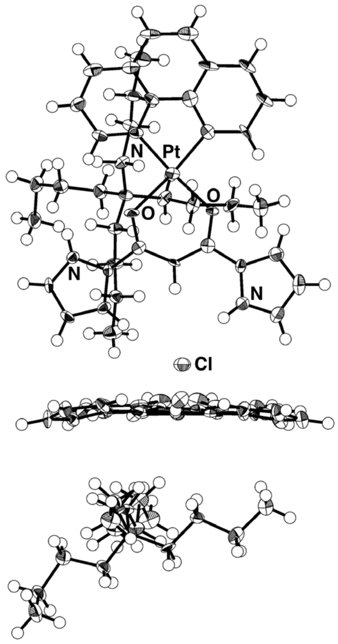

Fig. S16 Ortep drawings of single-crystal X-ray structures (top and side views) of **2d**·Cl^{-}-TBA^{+}_{M}. Thermal ellipsoids are scaled to the 50% probability level. Solvent molecules are omitted for clarity.

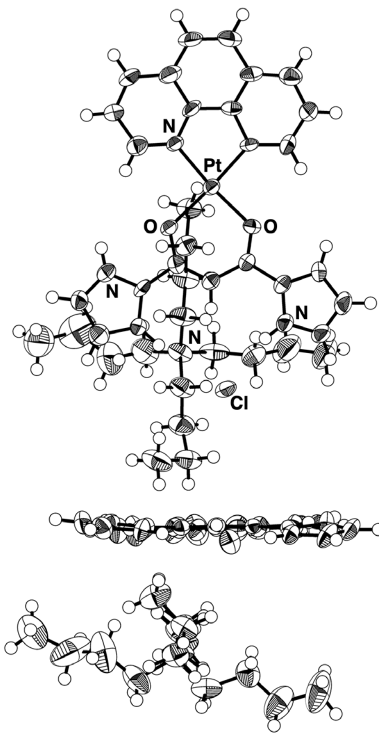

Fig. S17 Ortep drawings of single-crystal X-ray structures (top and side views) of **2d**·Cl^--TBA^+_E. Thermal ellipsoids are scaled to the 50% probability level. Solvent molecules are omitted for clarity.

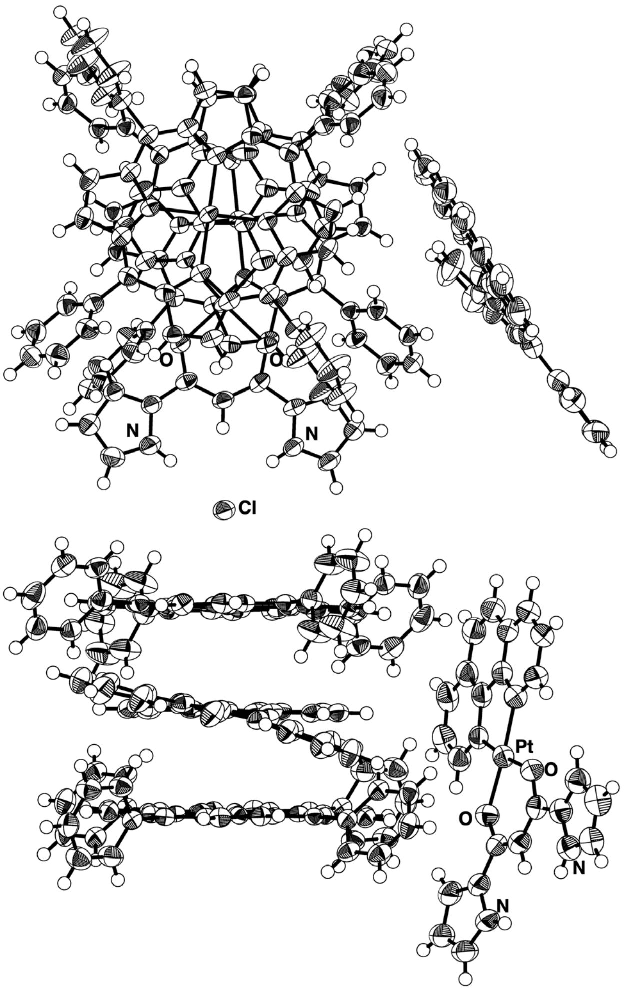

Fig. S18 Ortep drawings of single-crystal X-ray structures (top and side views) of **2d**$_2$·Cl^--$TPPAu^+$. Thermal ellipsoids are scaled to the 50% probability level. Solvent molecules are omitted for clarity.

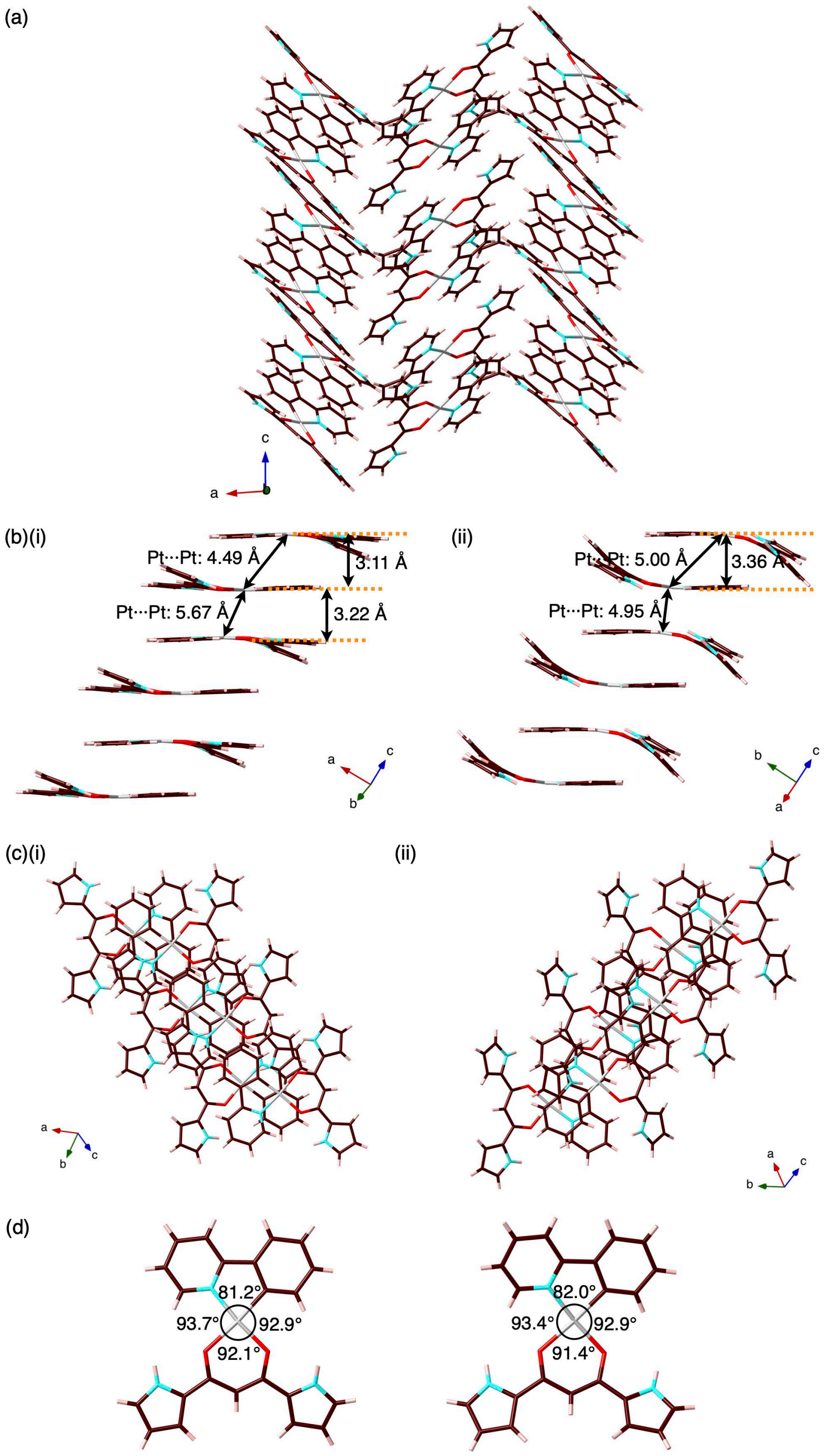

Fig. S19 Single-crystal X-ray structure of **2a**:[S1] (a) packing structure, (b) enlarged side views of the columnar structures of the two independent structures (i,ii), (c) top views of the columnar structures of the two independent structures (i,ii), and (d) constituting monomers. In (b)(i), the distances between two receptor mean planes (core 28 atoms) are 3.11/3.22 Å with the Pt···Pt distances of 4.49/5.67 Å. In (b)(ii), the distance between two planes (phenylpyridine–Pt complex (13 atoms)) is 3.36 Å with the Pt···Pt distances of 5.00/4.95 Å. The angles around Pt for two independent structures are 81.2°/92.9°/92.1°/93.7° and 82.0°/92.9°/91.4°/93.4°. Atom color code: brown, pink, blue, red, and gray refer to carbon, hydrogen, nitrogen, oxygen, and platinum, respectively.

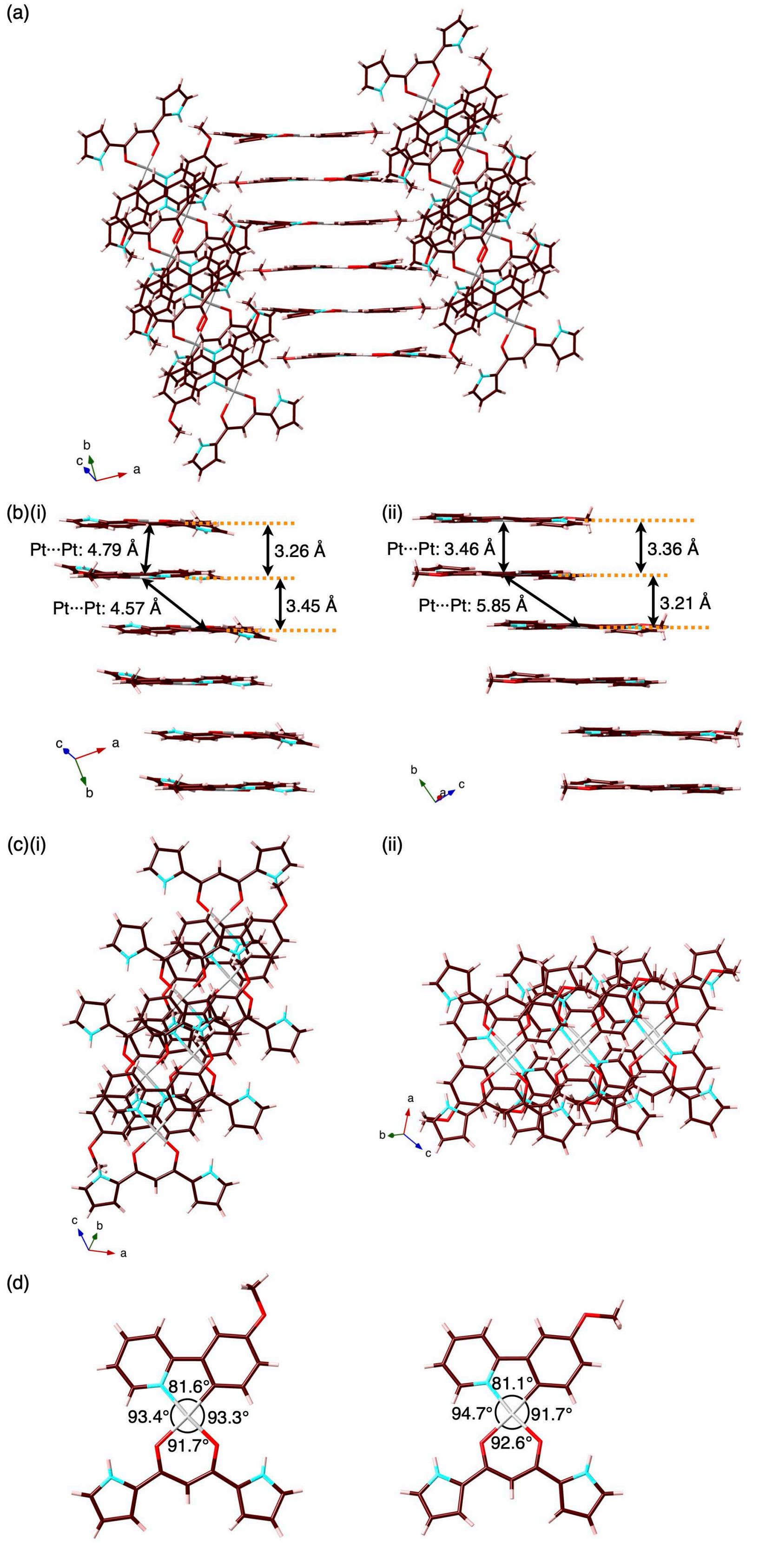

Fig. S20 Single-crystal X-ray structure of **2b**: (a) packing structure, (b) enlarged side views of the columnar structures of the two independent structures (i,ii), (c) top views of the columnar structures of the two independent structures (i,ii), and (d) constituting monomers. The distances between two receptor mean planes (core 28 atoms) are 3.26/3.45 and 3.21/3.36 Å with the Pt···Pt distances of 4.79/4.57 and 3.46/5.85 Å, respectively. The angles around Pt for two independent structures are 81.6°/93.3°/91.7°/93.4° and 81.1°/91.7°/92.6°/94.7°. Atom color code: brown, pink, blue, red, and gray refer to carbon, hydrogen, nitrogen, oxygen, and platinum, respectively.

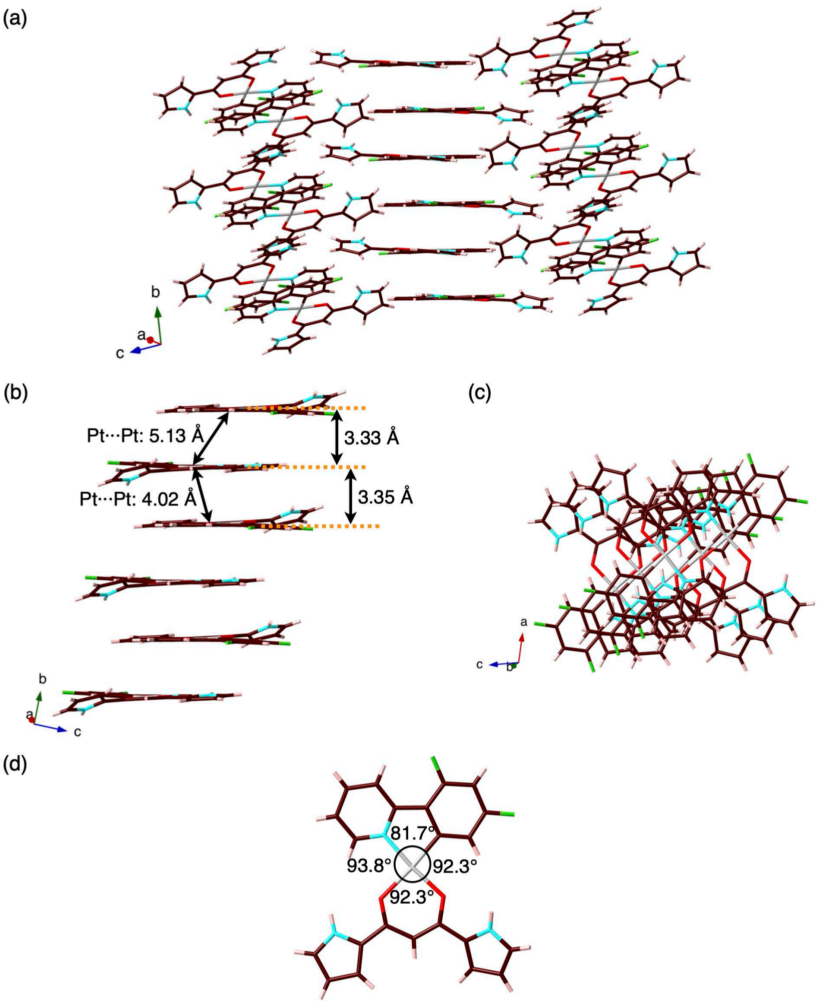

Fig. S21 Single-crystal X-ray structure of **2c**: (a) packing structure, (b) enlarged view of the columnar structure in (a), (c) top view of the columnar structure, and (d) constituting monomer. The distances between two receptor mean planes (core 28 atoms) are 3.33/3.35 Å with the Pt···Pt distances of 4.02/5.13 Å. The angles around Pt are 81.7°/92.3°/92.3°/93.8°. N–H···π interactions were formed between the pyrrole rings in the neighboring columns. Atom color code: brown, pink, blue, red, yellow green, and gray refer to carbon, hydrogen, nitrogen, oxygen, fluorine, and platinum, respectively.

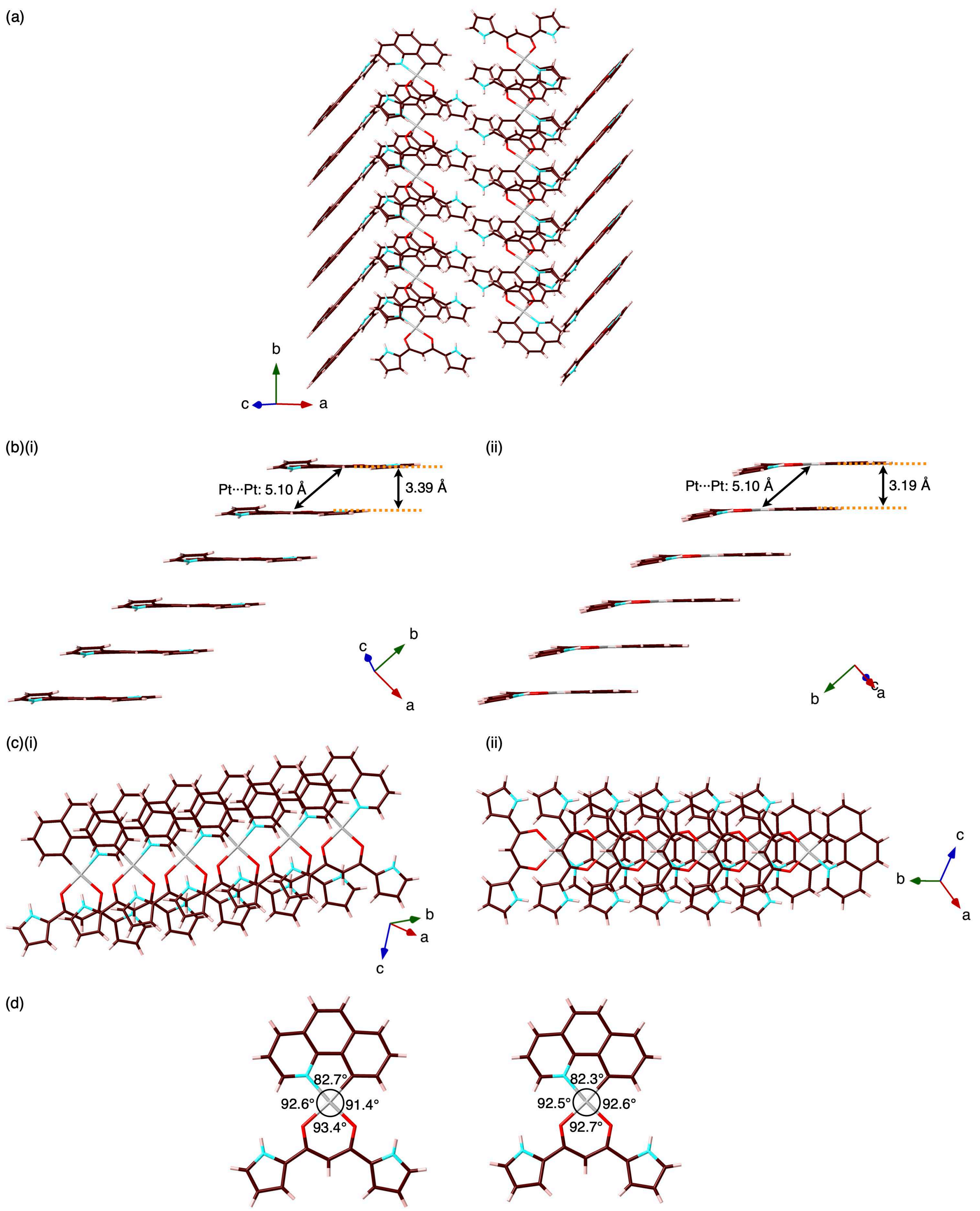

Fig. S22 Single-crystal X-ray structure of **2d**: (a) packing structure, (b) enlarged side views of the columnar structures of the two independent structures (i,ii), (c) top views of the columnar structures of the two independent structures (i,ii), and (d) constituting monomers. The distances between two receptor mean planes (core 28 atoms) are 3.39/3.19 Å with the Pt···Pt distances of 5.10/5.10 Å, respectively. The angles around Pt for two independent structures are 82.7°/91.4°/93.4°/92.6° and 82.3°/92.6°/92.7°/92.5°. Atom color code: brown, pink, blue, red, and gray refer to carbon, hydrogen, nitrogen, oxygen, and platinum, respectively.

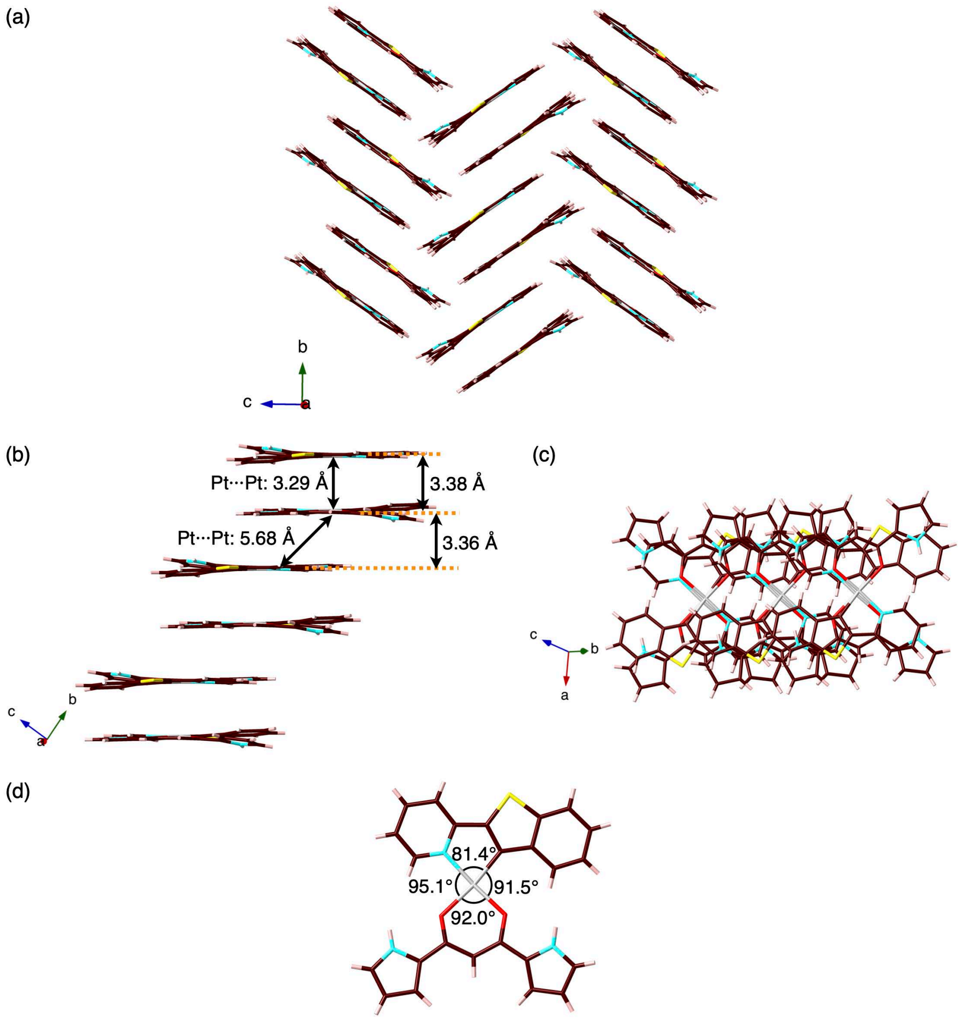

Fig. S23 Single-crystal X-ray structure of **2e**: (a) packing structure, (b) enlarged view of the columnar structure in (a), (c) top view of the columnar structure, and (d) constituting monomer. The distances between two receptor mean planes (core 28 atoms) are 3.38/3.36 Å with the Pt···Pt distances of 3.29/5.68 Å. The angles around Pt are 81.4°/91.5°/92.0°/95.1°. Atom color code: brown, pink, blue, red, yellow, and gray refer to carbon, hydrogen, nitrogen, oxygen, sulfur, and platinum, respectively.

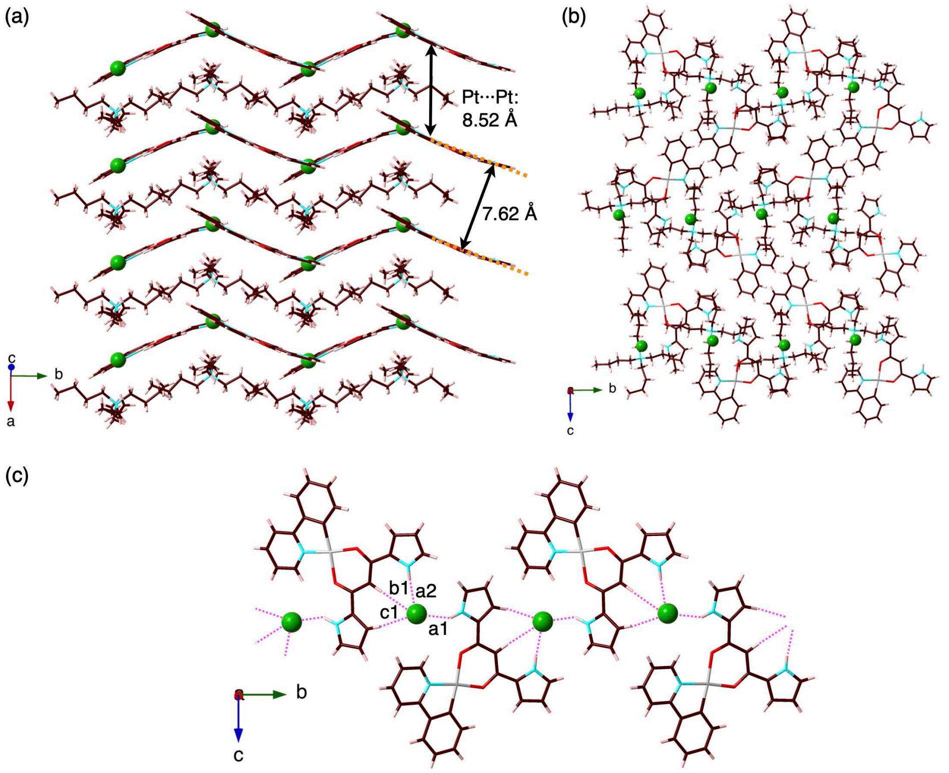

Fig. S24 Single-crystal X-ray structure of **2a**·Cl^--TBA^+: (a) packing structure as charge-by-charge assembly, (b) top view of the charge-by-charge assembly in (a), and (c) Cl^--bridged chain structure. The distance between two receptor mean planes (core 28 atoms) is 7.62 Å with the Pt···Pt distance of 8.52 Å. The N(–H)···Cl^-, $C_{bridging}$(–H)···Cl^-, and βC(–H)···Cl^- hydrogen-bonding distances are 3.18 (a1)/3.17 (a2), 3.78 (b1), and 3.63 (c1) Å, respectively, whereas the nearest Cl^-···Cl^- distance is 8.90 Å. The dihedral angles between pyrrole and diketone–Pt six-membered ring are 5.0° and 8.36°. Atom color code: brown, pink, blue, red, green (sphere), and gray refer to carbon, hydrogen, nitrogen, oxygen, chlorine, and platinum, respectively.

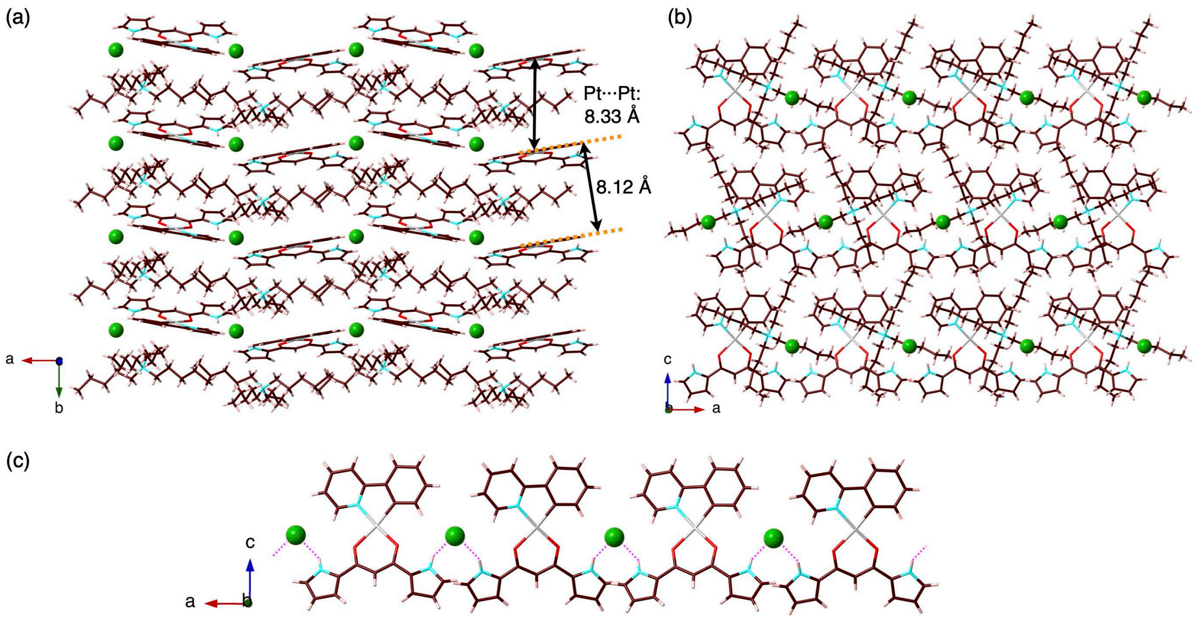

Fig. S25 Single-crystal X-ray structure of **2a**·Cl^--$TPeA^+$: (a) packing structure as charge-by-charge assembly, (b) top view of the charge-by-charge assembly in (a), and (c) Cl^--bridged chain structure. The distance between two receptor mean planes (core 28 atoms) is 8.12 Å with the Pt···Pt distance of 8.33 Å. The N(–H)···Cl^- hydrogen-bonding distance is 3.14 Å, whereas the nearest Cl^-···Cl^- distance is 10.72 Å. The dihedral angles between pyrrole and diketone–Pt six-membered rings are 8.65° and 11.1°. Atom color code: brown, pink, blue, red, green (sphere), and gray refer to carbon, hydrogen, nitrogen, oxygen, chlorine, and platinum, respectively.

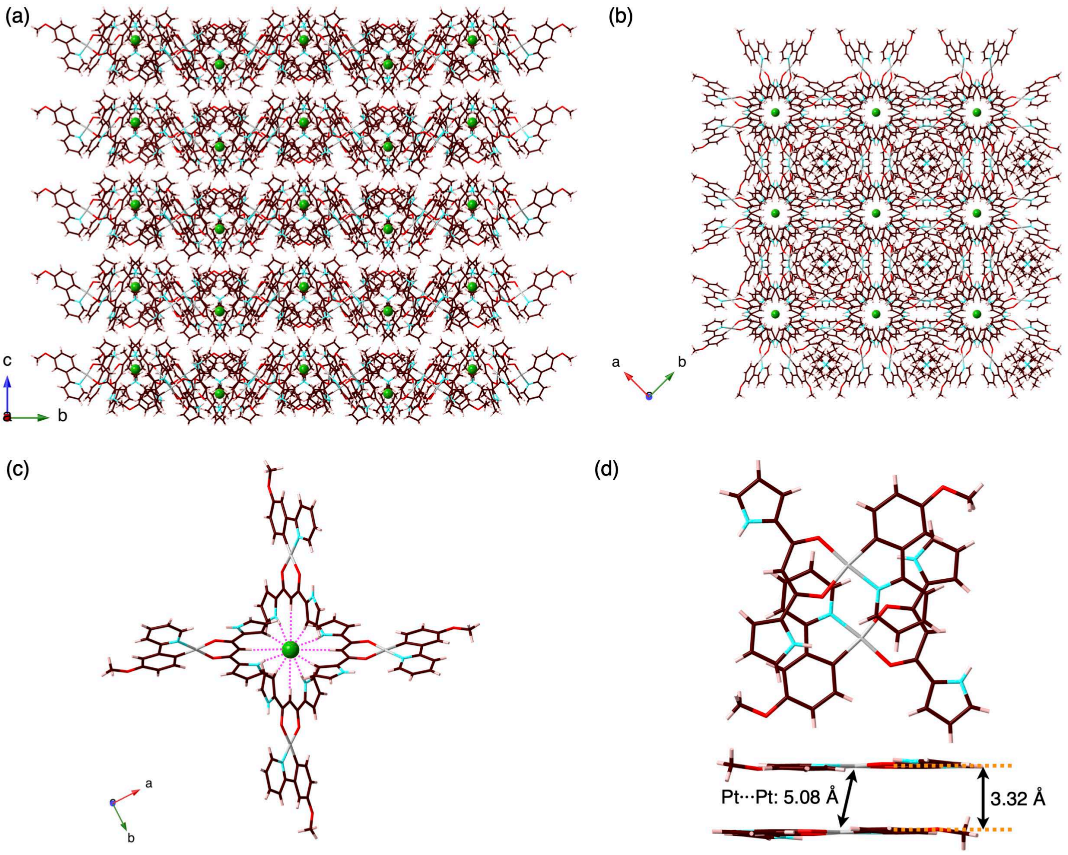

Fig. S26 Single-crystal X-ray structure of **2b**$_4$·Cl$^-$-TBA$^+$: (a) packing structure, (b) top view of the packing structure in (a), (c) [4+1]-type complex, and (d) stacking dimer of the Pt^{II} complexes. The N(–H)···Cl$^-$/C$_{bridging}$(–H)···Cl$^-$/βC(–H)···Cl$^-$ hydrogen-bonding distances in two independent binding sites are 3.30/3.93/3.67 Å. The stacking distance between two receptor mean planes (core 28 atoms) is 3.32 Å with the Pt···Pt distance of 5.08 Å. The dihedral angles between pyrrole and diketone–Pt six-membered rings are 4.17° and 4.43°. Atom color code: brown, pink, blue, red, green (sphere), and gray refer to carbon, hydrogen, nitrogen, oxygen, chlorine, and platinum, respectively.

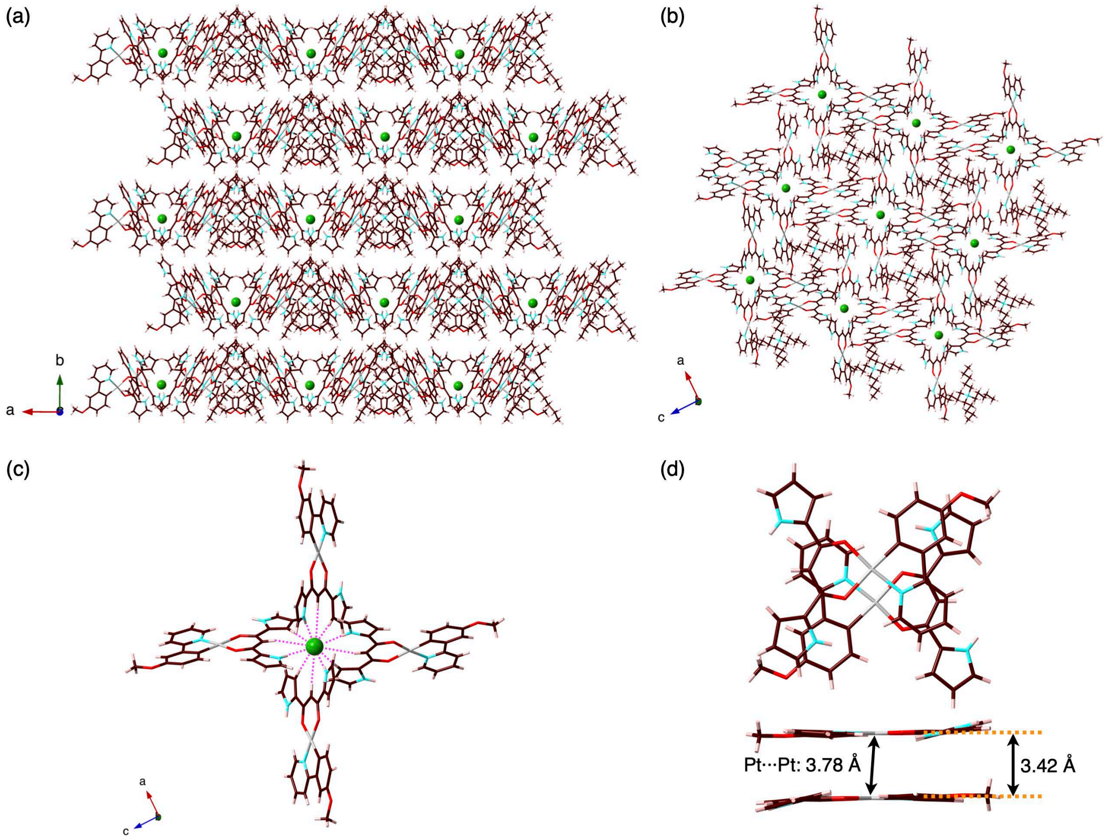

Fig. S27 Single-crystal X-ray structure of **2b**$_4$·Cl$^-$-TPeA$^+$: (a) packing structure, (b) top view of the packing structure in (a), (c) [4+1]-type complex, and (d) stacking dimer of the Pt^{II} complexes. The N(–H)···Cl$^-$/C$_{bridging}$(–H)···Cl$^-$/βC(–H)···Cl$^-$ hydrogen-bonding distances in two independent binding sites are 3.34/3.98/3.75 and 3.29/3.79/3.47 Å. The stacking distance between two receptor mean planes (core 28 atoms) is 3.42 Å with the Pt···Pt distance of 3.78 Å. The dihedral angles between pyrrole and diketone–Pt six-membered rings are 6.86°/13.3° and 3.80°/9.95° for two independent structures. Atom color code: brown, pink, blue, red, green (sphere), and gray refer to carbon, hydrogen, nitrogen, oxygen, chlorine, and platinum, respectively.

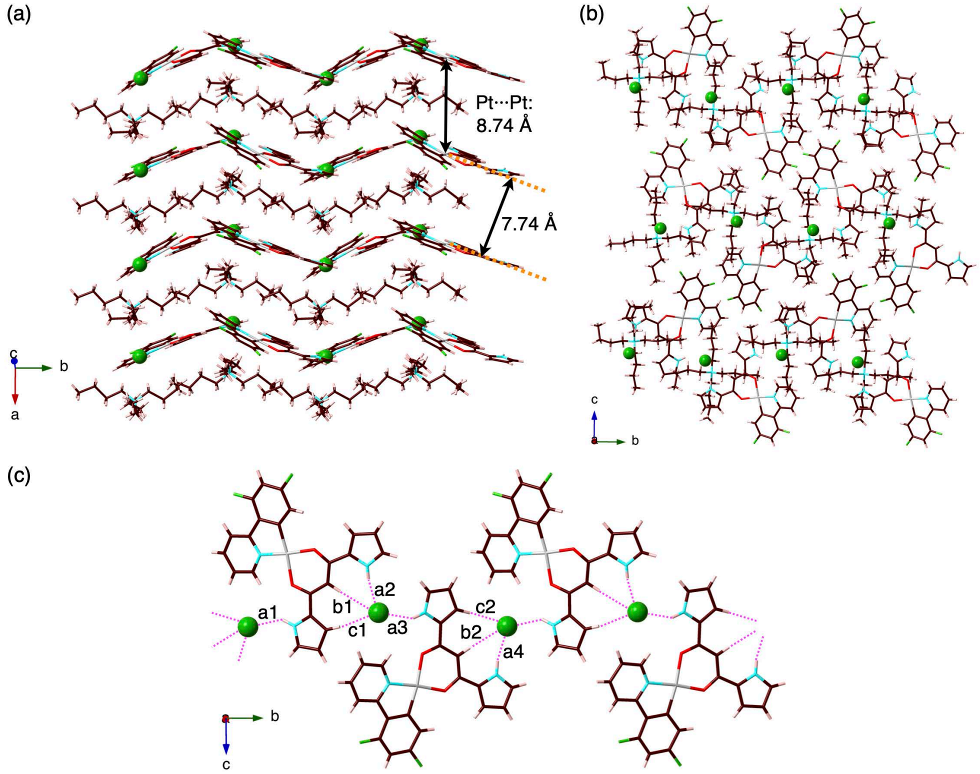

Fig. S28 Single-crystal X-ray structure of **2c**·Cl⁻-TBA⁺: (a) packing structure as charge-by-charge assembly, (b) top view of the charge-by-charge assembly in (a), and (c) Cl⁻-bridged chain structure. The distance between two receptor mean planes (core 28 atoms) is 7.74 Å with the Pt···Pt distance of 8.74 Å. The N(–H)···Cl⁻, $C_{bridging}$(–H)···Cl⁻, and βC(–H)···Cl⁻ hydrogen-bonding distances are 3.19 (a1)/3.14 (a2)/3.18 (a3)/3.20 (a4), 3.85 (b1)/3.84 (b2), and 3.65 (c1)/3.72 (c2) Å, respectively, whereas the nearest Cl⁻···Cl⁻ distance is 9.01 Å. The dihedral angles between pyrrole and diketone–Pt six-membered rings are 3.96°/7.54°, 3.70°/4.50°, 6.91°/10.8°, and 6.25°/10.0° for four independent structures. Atom color code: brown, pink, blue, red, yellow green, green (sphere), and gray refer to carbon, hydrogen, nitrogen, oxygen, fluorine, chlorine, and platinum, respectively.

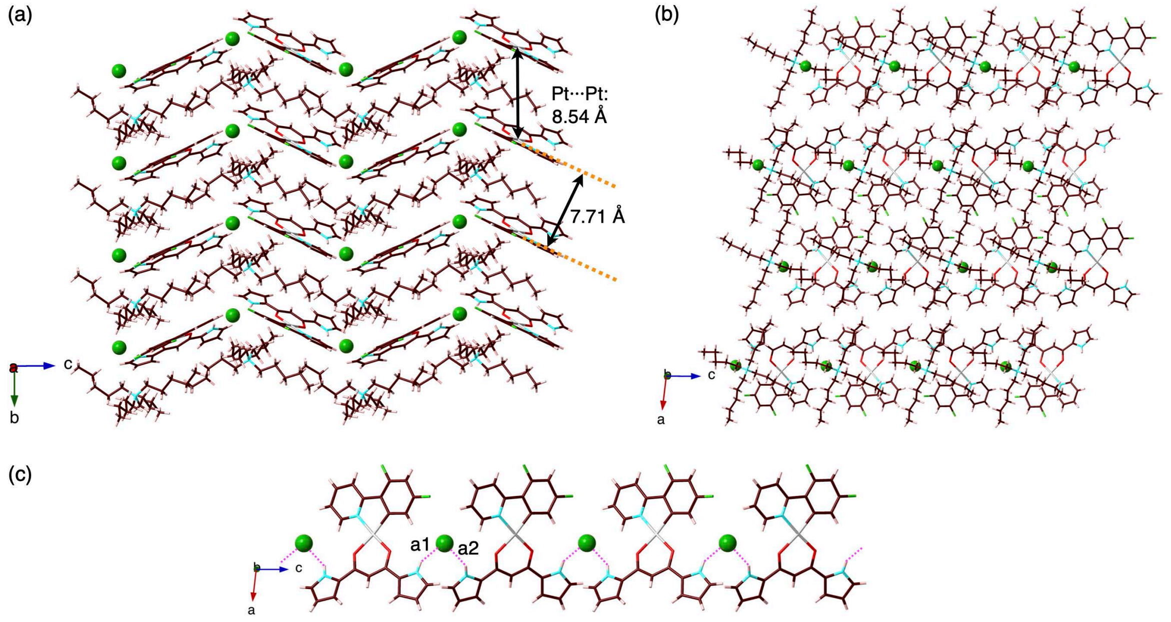

Fig. S29 Single-crystal X-ray structure of **2c**·Cl⁻-TPeA⁺: (a) packing structure as charge-by-charge assembly, (b) top view of the charge-by-charge assembly in (a), and (c) Cl⁻-bridged chain structure. The distance between two receptor mean planes (core 28 atoms) is 7.71 Å with the Pt···Pt distance of 8.33 Å. The N(–H)···Cl⁻ hydrogen-bonding distances are 3.14 (a1) and 3.18 (a2) Å, whereas the nearest Cl⁻···Cl⁻ distance is 10.94 Å. The dihedral angles between pyrrole and diketone–Pt six-membered rings are 10.8° and 12.8°. Atom color code: brown, pink, blue, red, yellow green, green (sphere), and gray refer to carbon, hydrogen, nitrogen, oxygen, fluorine, chlorine, and platinum, respectively.

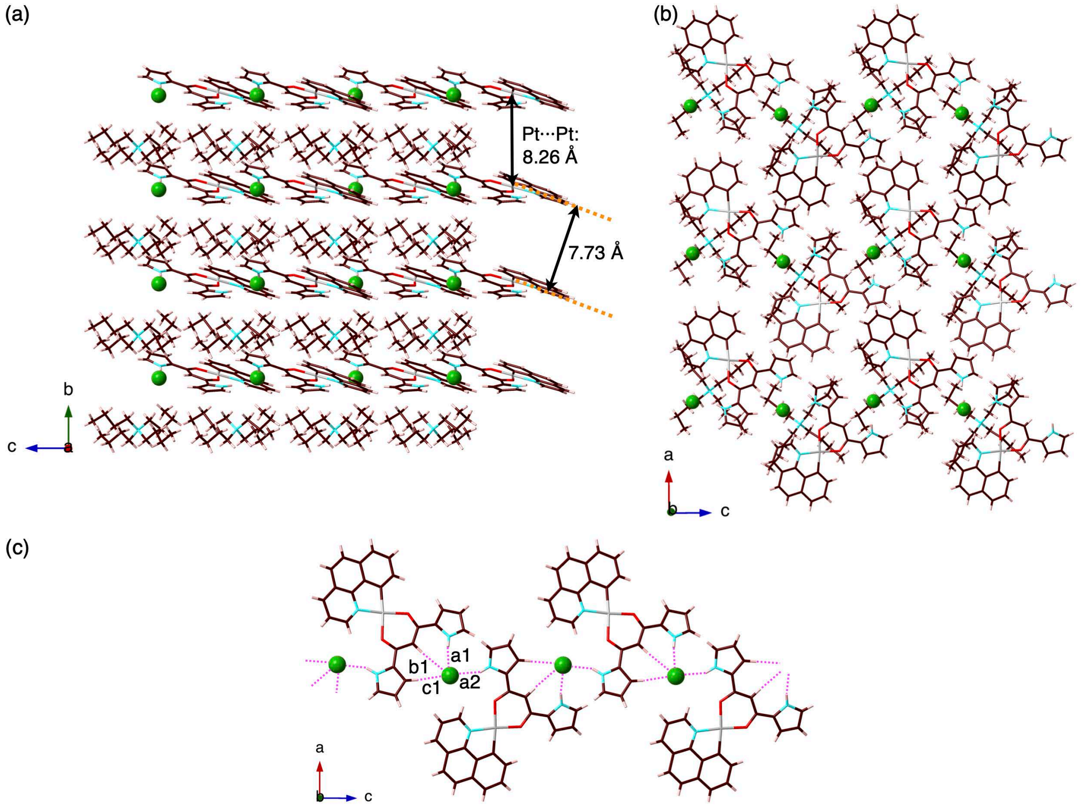

Fig. S30 Single-crystal X-ray structure of **2d**·Cl^--TBA^+_M: (a) packing structure as charge-by-charge assembly, (b) top view of the charge-by-charge assembly in (a), and (c) Cl^--bridged chain structure. The distance between two receptor mean planes (core 28 atoms) is 7.73 Å with the Pt···Pt distance of 8.26 Å. The N(–H)···Cl^-, $C_{bridging}$(–H)···Cl^-, and βC(–H)···Cl^- hydrogen-bonding distances are 3.16 (a1)/3.18 (a2), 3.76 (b1), and 3.54 (c1) Å, respectively, whereas the nearest Cl^-···Cl^- distance is 8.64 Å. The dihedral angles between pyrrole and diketone–Pt six-membered rings are 9.4° and 13.2°. Atom color code: brown, pink, blue, red, green (sphere), and gray refer to carbon, hydrogen, nitrogen, oxygen, chlorine, and platinum, respectively.

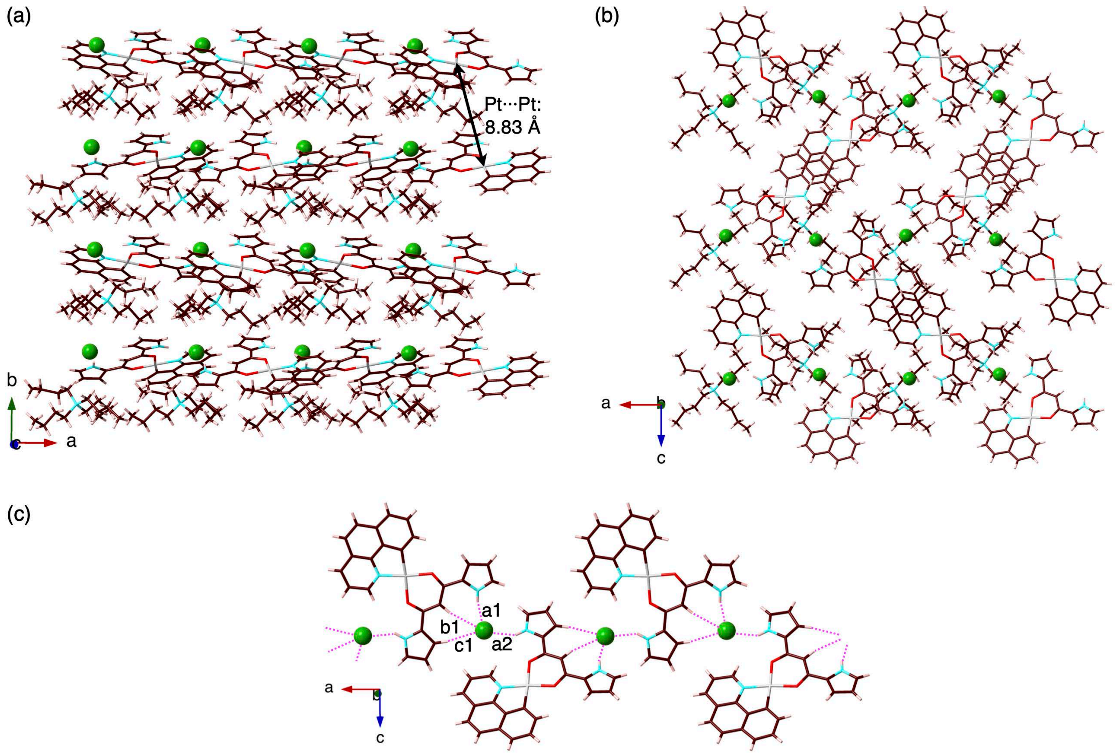

Fig. S31 Single-crystal X-ray structure of **2d**·Cl$^-$-TBA^+_E: (a) packing structure as charge-by-charge assembly, (b) top view of the charge-by-charge assembly in (a), and (c) Cl$^-$-bridged chain structure. The distance between two receptor mean planes (core 28 atoms) is 7.51 Å with the Pt···Pt distance of 8.83 Å. The N(–H)···Cl$^-$, C$_{bridging}$(–H)···Cl$^-$, and βC(–H)···Cl$^-$ hydrogen-bonding distances are 3.10 (a1)/3.16 (a2), 3.82 (b1), and 3.73 (c1) Å, respectively, whereas the nearest Cl$^-$···Cl$^-$ distance is 8.87 Å. The dihedral angles between pyrrole and diketone–Pt six-membered rings are 7.84° and 11.0°. Atom color code: brown, pink, blue, red, green (sphere), and gray refer to carbon, hydrogen, nitrogen, oxygen, chlorine, and platinum, respectively.

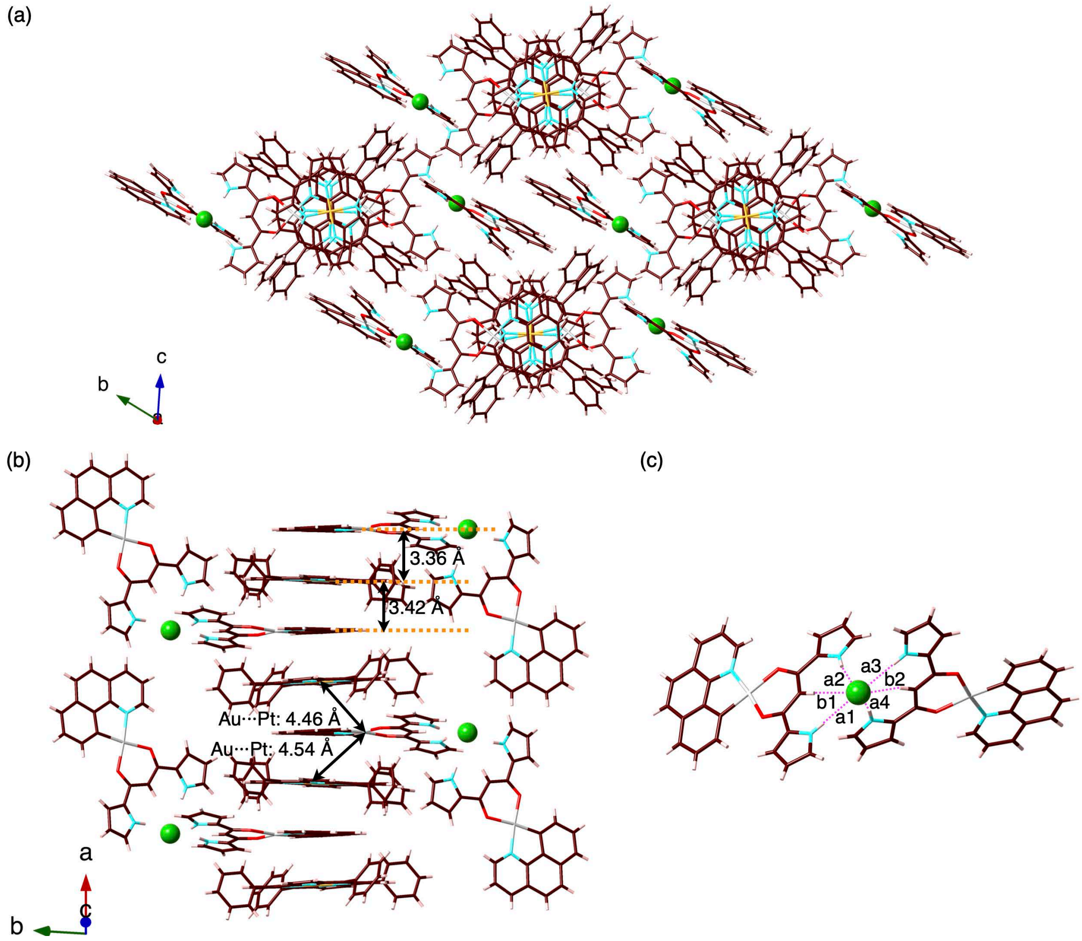

Fig. S32 Single-crystal X-ray structure of **2d**$_2$·Cl$^−$-TPPAu$^+$: (a) columnar structure along the *a* axis, (b) enlarged view of the stacking assembly, whose **2d** unit forms the [2+1]-type complex **2d**$_2$·Cl$^−$ with another **2d** that is not in the column, and (c) enlarged view of **2d**$_2$·Cl$^−$. The C(–H)···Cl$^−$ and N(–H)···Cl$^−$ distances are 3.75 (b1)/3.76 (b2) and 3.36 (a1)/3.22 (a2)/3.30 (a3)/3.30 (a4) Å, respectively. The stacking distances between TPPAu$^+$ and **2d**·Cl$^−$, estimated from the average distances between the TPPAu$^+$ (core 25-atom plane) and **2d**·Cl$^−$ (30-atom plane) units, are 3.36 and 3.42 Å. The Pt···Au distances in the columnar structure were 4.46 and 4.54 Å, showing the correlation with the stacking of the benzoquinoline unit with the core unit of TPPAu$^+$. The **2d**·Cl$^−$ unit in the charge-by-charge columns exhibited a distorted conformation, as indicated by the dihedral angle of 13.9° between benzoquinoline and dipyrrolyldiketone units. Such a [2+1]-type complex was also observed for dipyrrolyldiketone boron complexes,[S10] which formed charge-by-charge stacking assemblies with planar π-electronic countercations. Charge-by-charge stacking assembly of **2d**$_2$·Cl$^−$-TPPAu$^+$ is mainly formed by the stacking of the planar benzoquinoline unit and the TPPAu$^+$ core part, and thus the Cl$^−$-bound part of **2d**·Cl$^−$ is relatively out of the stacking core unit of the TPPAu$^+$. The Cl$^−$ is also stabilized by the hydrogen bonding of **2d** from the other side to form the [2+1]-type complex. Atom color code: brown, pink, blue, red, green (sphere), gray, and orange refer to carbon, hydrogen, nitrogen, oxygen, chlorine, platinum, and gold, respectively.

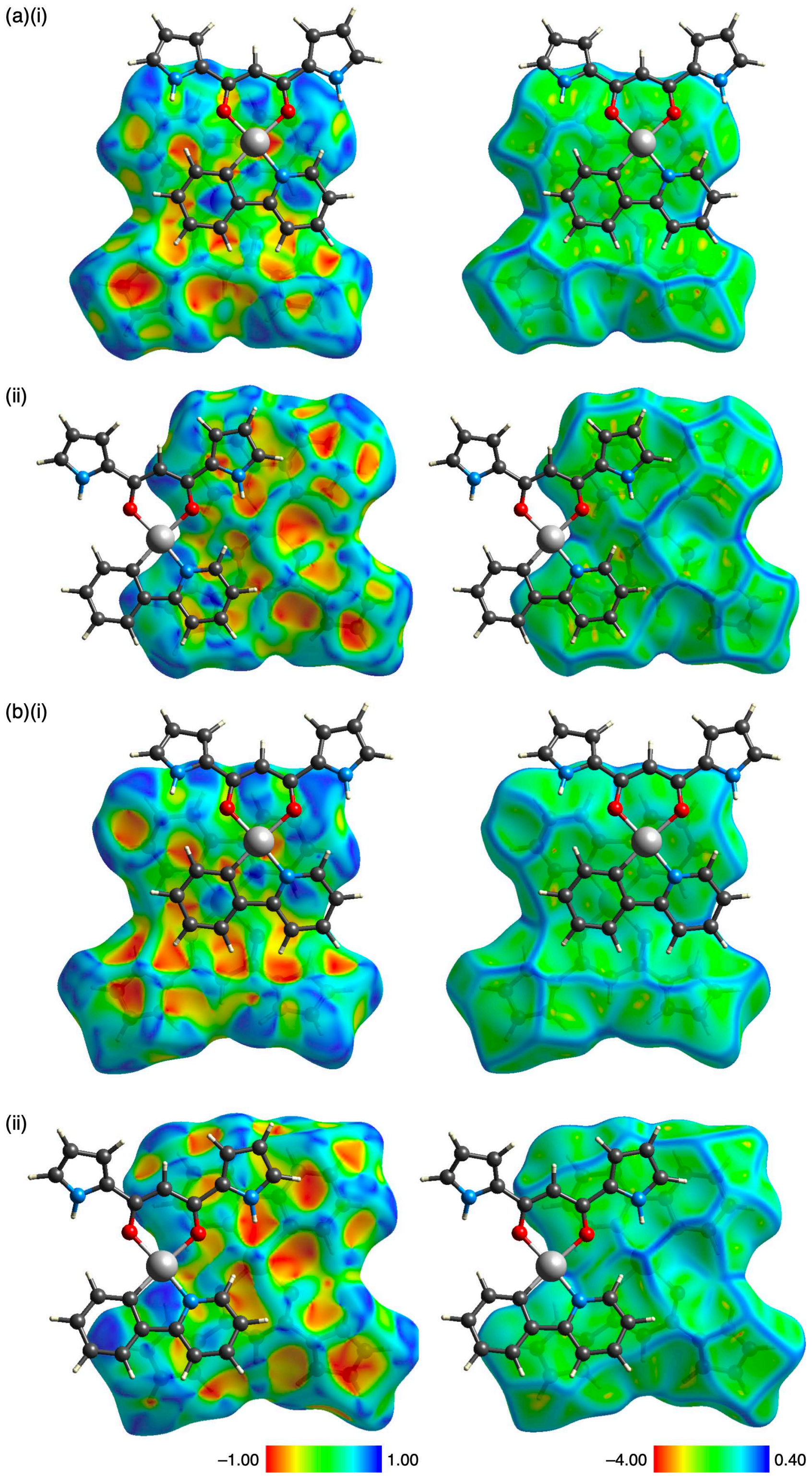

Fig. S33 Hirshfeld surfaces[S11] of **2a** for two different stacking modes (i,ii) of independent structures (a,b) mapped over shape-index (left) and curvedness (right) properties.

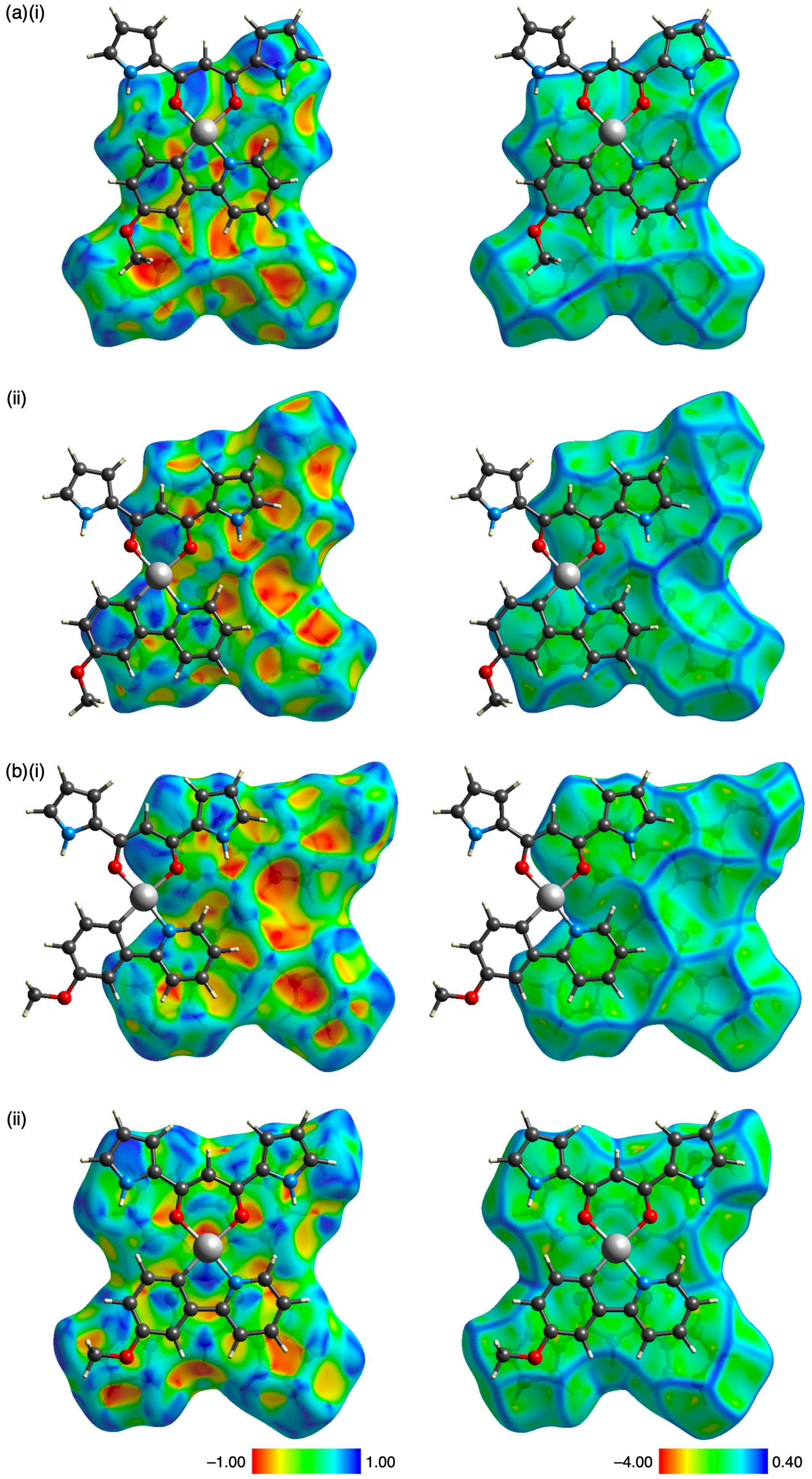

Fig. S34 Hirshfeld surfaces[S11] of **2b** for two different stacking modes (i,ii) of independent structures (a,b) mapped over shape-index (left) and curvedness (right) properties.

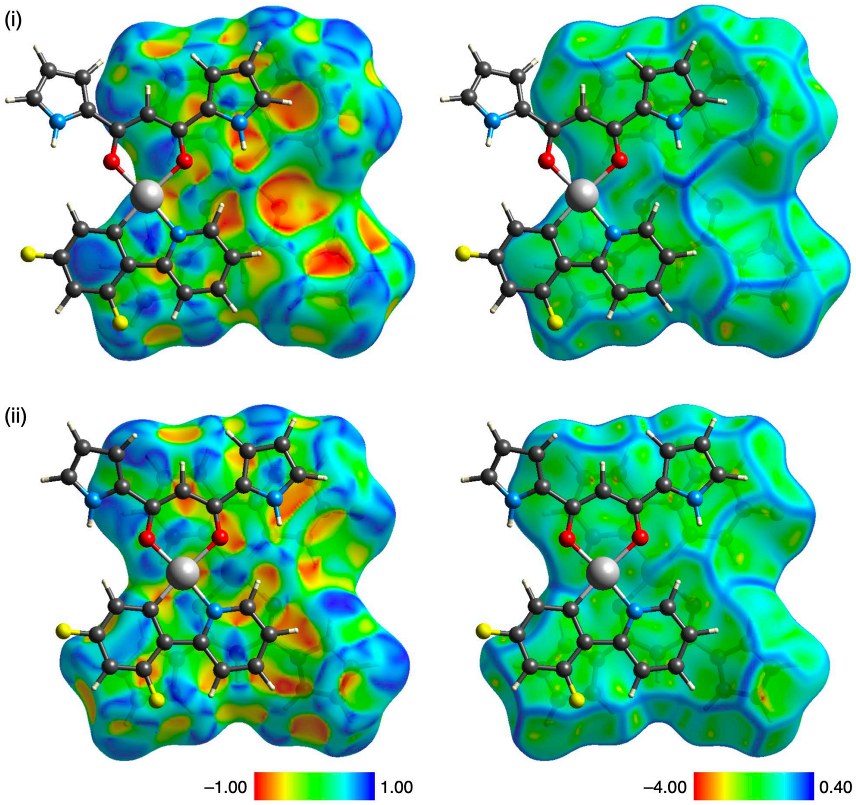

Fig. S35 Hirshfeld surfaces[S11] of **2c** for two stacking modes (i,ii) mapped over shape-index (left) and curvedness (right) properties.

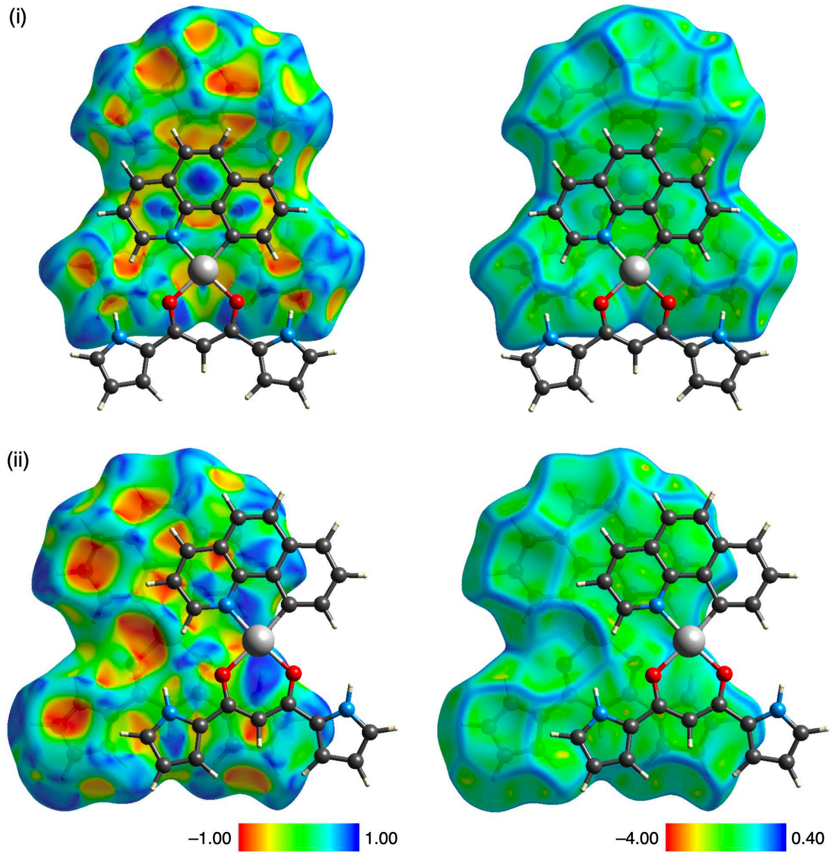

Fig. S36 Hirshfeld surfaces[S11] of **2d** for two stacking modes (i,ii) mapped over shape-index (left) and curvedness (right) properties.

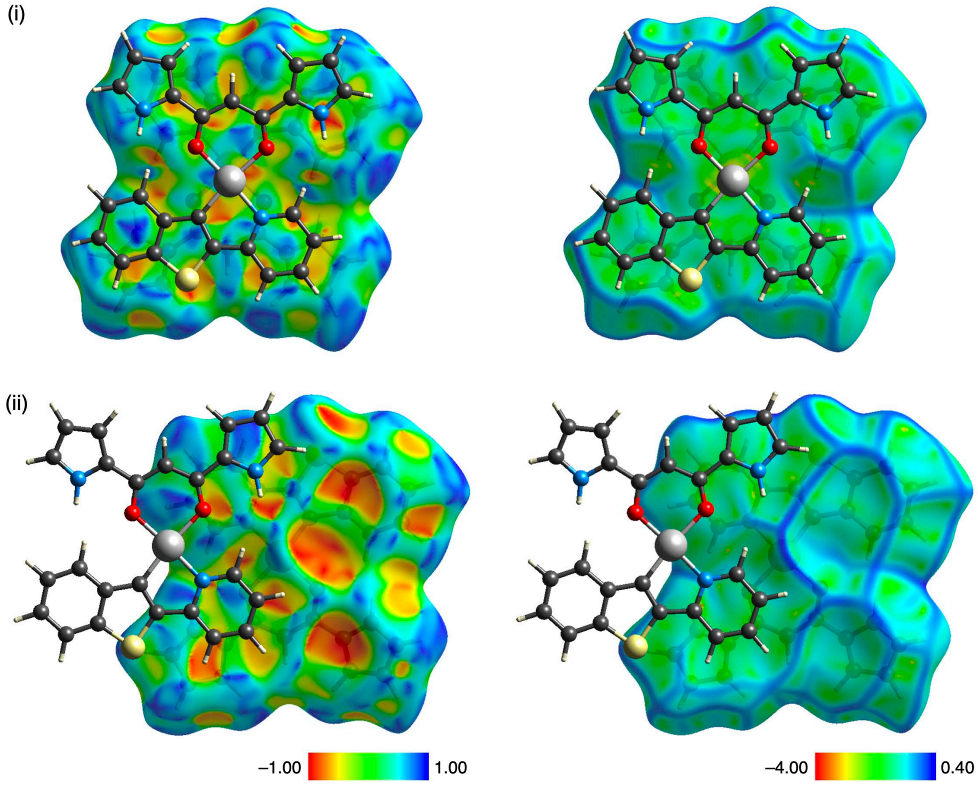

Fig. S37 Hirshfeld surfaces[S11] of **2e** for two stacking modes (i,ii) mapped over shape-index (left) and curvedness (right) properties.

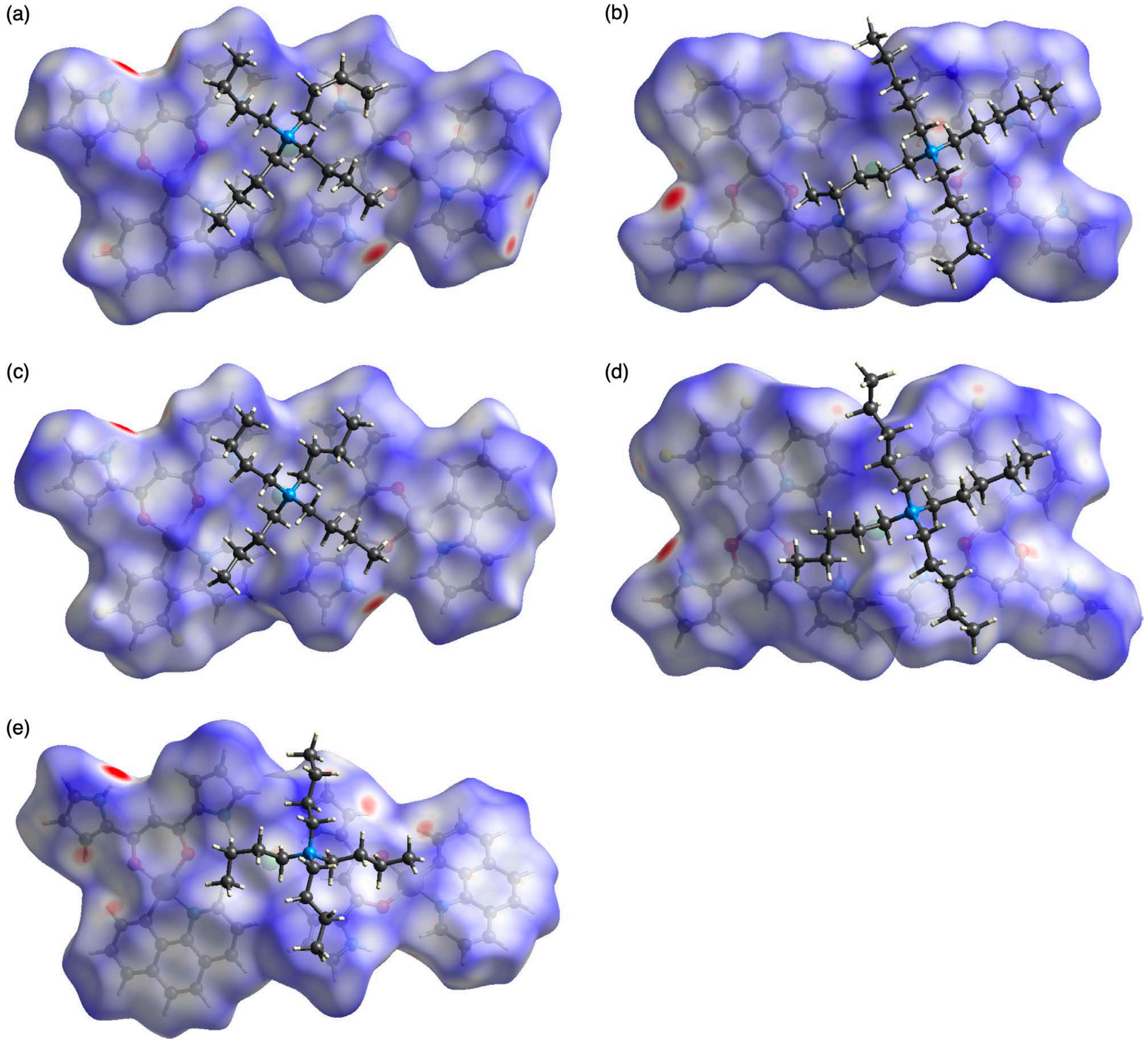

Fig. S38 Hirshfeld surfaces[S11] of (a) **2a**·Cl$^-$-TBA$^+$, (b) **2a**·Cl$^-$-TPeA$^+$, (c) **2c**·Cl$^-$-TBA$^+$, (d) **2c**·Cl$^-$-TPeA$^+$, and (e) **2d**·Cl$^-$-TBA^+_E for stacking structures mapped with d_{norm} over the range –0.4 to 1.5. No characteristic close contacts are observed between the countercations and Pt^{II} complexes.

[S4] (a) E. B. Fleischer and A. Laszlo, *Inorg. Nucl. Chem. Lett.*, 1969, **5**, 373–376; (b) R. Timkovich and A. Tulinsky, *Inorg. Chem.*, 1977, **16**, 962–963; (c) A. M. Shachter, E. B. Fleischer and R. C. Haltiwanger, *Acta Crystallogr. Sect. C*, 1987, **43**, 1876–1878; (d) C.-M. Che, R. W.-Y. Sun, W.-Y. Yu, C.-B. Ko, N. Zhu and H. Sun, *Chem. Commun.*, 2003, 1718–1719; (e) R. W.-Y. Sun, C. K.-L. Li, D.-L. Ma, J. J. Yan, C.-N. Lok, C.-H. Leung, N. Zhu and C.-M. Che, *Chem. Eur. J.*, 2010, **16**, 3097–3113; (f) Y. Haketa, Y. Bando, Y. Sasano, H. Tanaka, N. Yasuda, I. Hisaki and H. Maeda, *iScience*, 2019, **14**, 241–256.

[S5] K. Sugimoto, H. Ohsumi, S. Aoyagi, E. Nishibori, C. Moriyoshi, Y. Kuroiwa, H. Sawa and M. Takata, *AIP Conf. Proc.*, 2010, **1234**, 887–890.

[S6] (a) N. Yasuda, H. Murayama, Y. Fukuyama, J. E. Kim, S. Kimura, K. Toriumi, Y. Tanaka, Y. Moritomo, Y. Kuroiwa, K. Kato, H. Tanaka and M. Takata, *J. Synchrotron Rad.*, 2009, **16**, 352–357; (b) N. Yasuda, Y. Fukuyama, K. Toriumi, S. Kimura and M. Takata, *AIP Conf. Proc.*, 2010, **1234**, 147–150.

[S7] G. M. Sheldrick, *Acta Crystallogr. Sect. A*, 2008, **64**, 112–122.

[S8] (a) *Yadokari-XG*, Software for Crystal Structure Analyses, K. Wakita, 2001; (b) C. Kabuto, S. Akine, T. Nemoto and E. Kwon, *J. Cryst. Soc. Jpn.*, 2009, **51**, 218–224.

[S9] A. L. Spek, *Acta Crystallogr. Sect. D*, 2009, **65**, 148–155.

[S10] (a) Y. Haketa, M. Takayama and H. Maeda, *Org. Biomol. Chem.*, 2012, **10**, 2603–2606; (b) R. Yamakado, T. Sakurai, W. Matsuda, S. Seki, N. Yasuda, S. Akine and H. Maeda, *Chem. Eur. J.*, 2016, **22**, 626–638; (c) R. Yamakado, Y. Ashida, R. Sato, Y. Shigeta, N. Yasuda and H. Maeda, *Chem. Eur. J.*, 2017, **23**, 4160–4168.

[S11] P. R. Spackman, M. J. Turner, J. J. McKinnon, S. K. Wolff, D. J. Grimwood, D. Jayatilaka and M. A. Spackman, *J. Appl. Cryst.*, 2021, **54**, 1006–1011.

3. Theoretical Studies

Computational Method. Calculations for optimized structures, electrostatic potentials (ESP), molecular orbitals, and UV/vis spectra were performed using the *Gaussian 09* program.[S12] The ground states (S_0) and excited triplet states (T_1) were optimized at the CAM-B3LYP level by using 6-31+G(d,p) basis set for C, H, N, O, F, S, and Cl and the LanL2DZ basis set and associated effective core potentials for Pt, whereas the excited singlet states (S_1) were optimized using the TD-DFT calculations at the same level to the ground states. Calculations for solid-state packing structures were performed using the *Gaussian 16* program.[S13] The central dimer and surroundings in the packing structure of **2a** were treated as the QM region at CAM-B3LYP/6-31+G(d,p) with the LanL2DZ basis set for Pt and MM region at UFF level, respectively. In the case of the packing structure of **2a**·Cl^--TBA^+, the central four **2a**, two Cl^-, two TBA^+, and two THF were treated as the QM region at CAM-B3LYP/3-21G(d,p) with the LanL2DZ basis set for Pt, whereas the surroundings were treated as the MM region at UFF level. In the both cases, the QM region has C_i site symmetry. The coordinates of the MM region, which was employed to include the packing effects in the solid state, were fixed during the geometry optimization of the QM region.

Vibronic Coupling Density. Diagonal vibronic coupling density (VCD) of electronic state n and vibrational mode α, $\eta_{n,\alpha}$, is given by an integrand of diagonal vibronic coupling constant (VCC), $V_{n,\alpha}$, as follows:[S14,15]

$$V_{n,\alpha} = \int d\boldsymbol{x}\, \eta_{n,\alpha}(\boldsymbol{x}) \tag{S1}$$

where $\boldsymbol{x} = (x, y, z)$ is a three-dimensional Cartesian coordinate. The diagonal VCD is expressed as

$$\eta_{n,\alpha}(\boldsymbol{x}) = \Delta\rho_{nm}(\boldsymbol{x}) \times v_\alpha(\boldsymbol{x}) \tag{S2}$$

Here, $\Delta\rho_{nm}(\boldsymbol{x})$ is an electron density difference between electronic state n and reference electronic state m. $v_\alpha(\boldsymbol{x})$ is a potential derivative defined by

$$v_\alpha(\boldsymbol{x}) = \left(\frac{\partial u(\boldsymbol{x})}{\partial Q_\alpha}\right)_{\mathbf{R}_0} \tag{S3}$$

where $u(\boldsymbol{x})$ is an attractive electron-nucleus potential that acts on a single electron and Q_α is a mass-weighted normal coordinate of vibrational mode α. $\mathbf{R}_0$ is a reference nuclear configuration taken at the equilibrium geometry of reference electronic state m. The VCD enables us to understand the origin of vibronic coupling in terms of electronic state and vibrational mode.

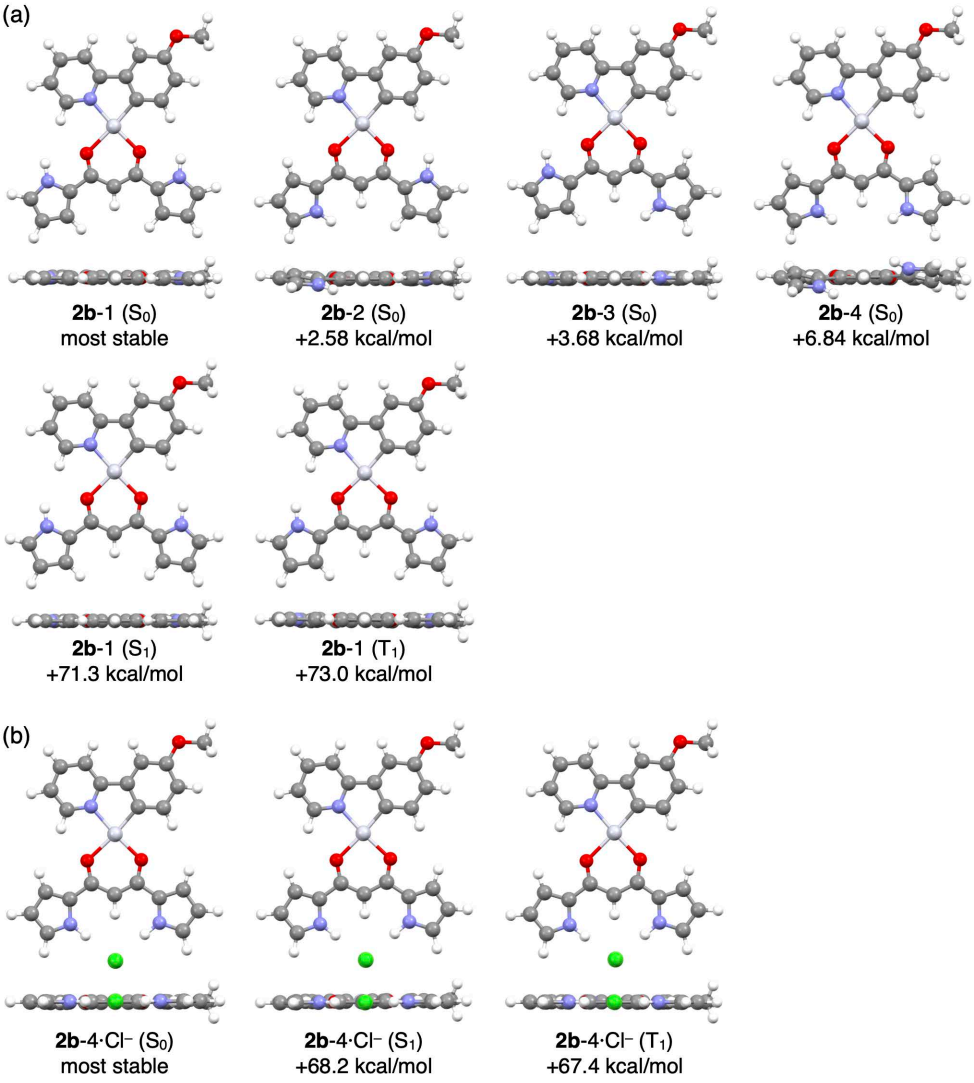

Fig. S39 Optimized structures and relative energies of (a) **2b** (four conformations) and (b) **2b**·Cl^- in the ground (S_0) state and singlet (S_1) and triplet (T_1) excited states. It is noteworthy that the energy of **2b**-1 as T_1 is slightly larger than that of S_1 state,[S16] and the reasons for such a positive ΔE_{ST} value will be discussed elsewhere.

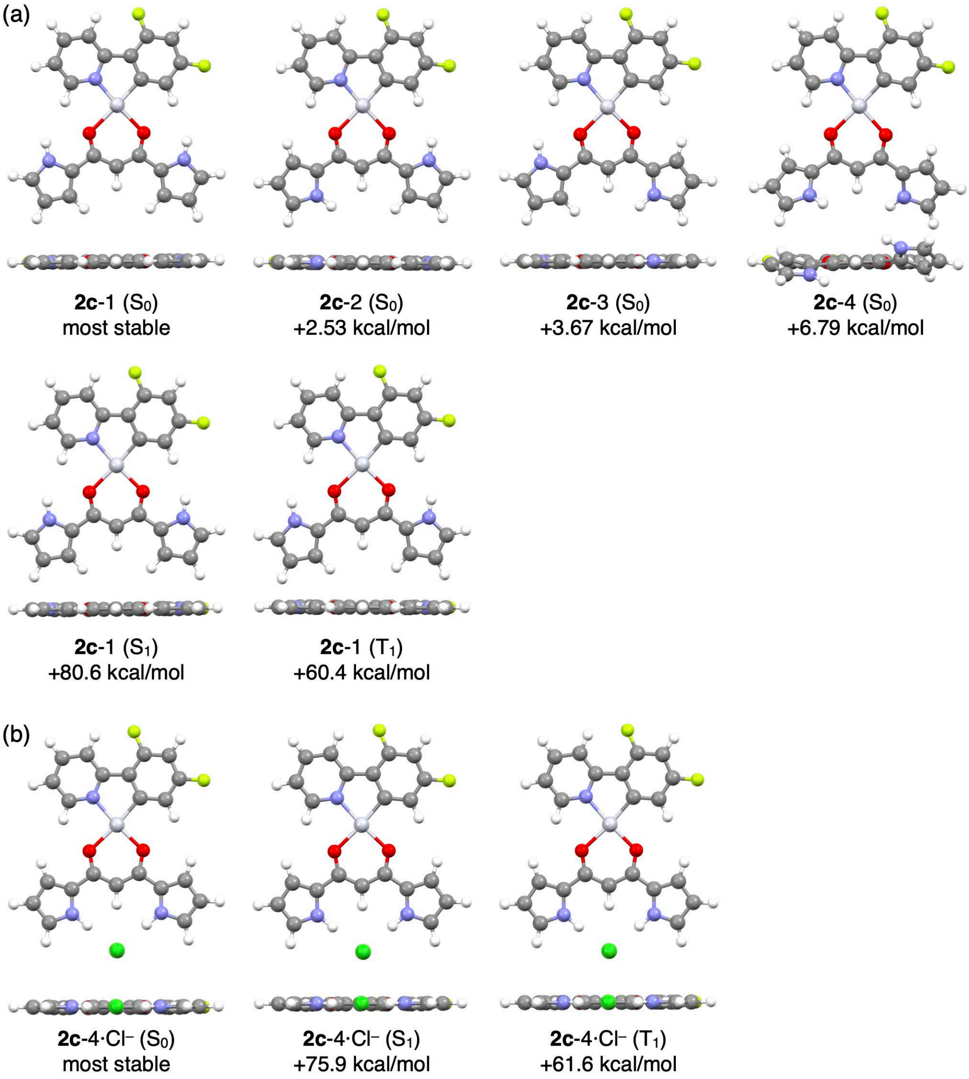

Fig. S40 Optimized structures and relative energies of (a) **2c** (four conformations) and (b) **2c**·Cl^- in the ground (S_0) state and singlet (S_1) and triplet (T_1) excited states.

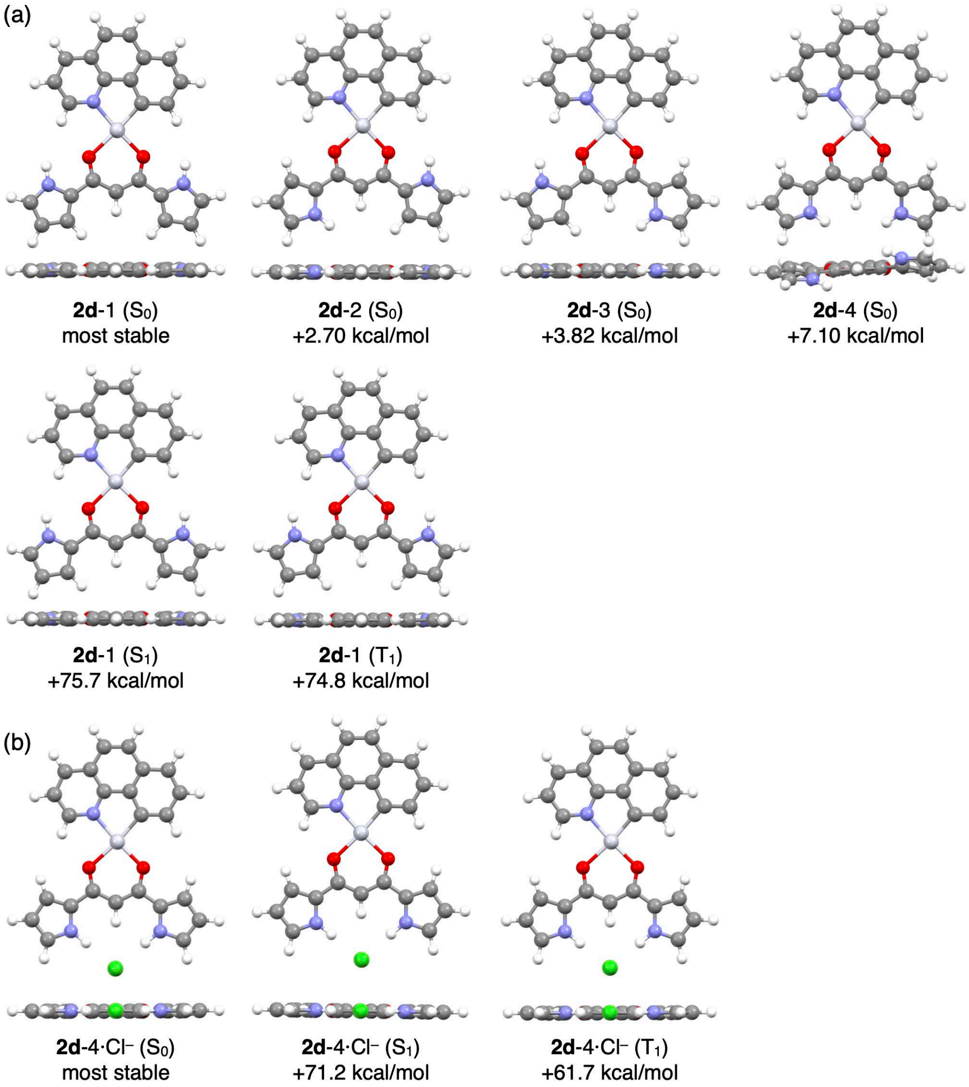

Fig. S41 Optimized structures and relative energies of (a) **2d** (four conformations) and (b) **2d**·Cl^- in the ground (S_0) state and singlet (S_1) and triplet (T_1) excited states.

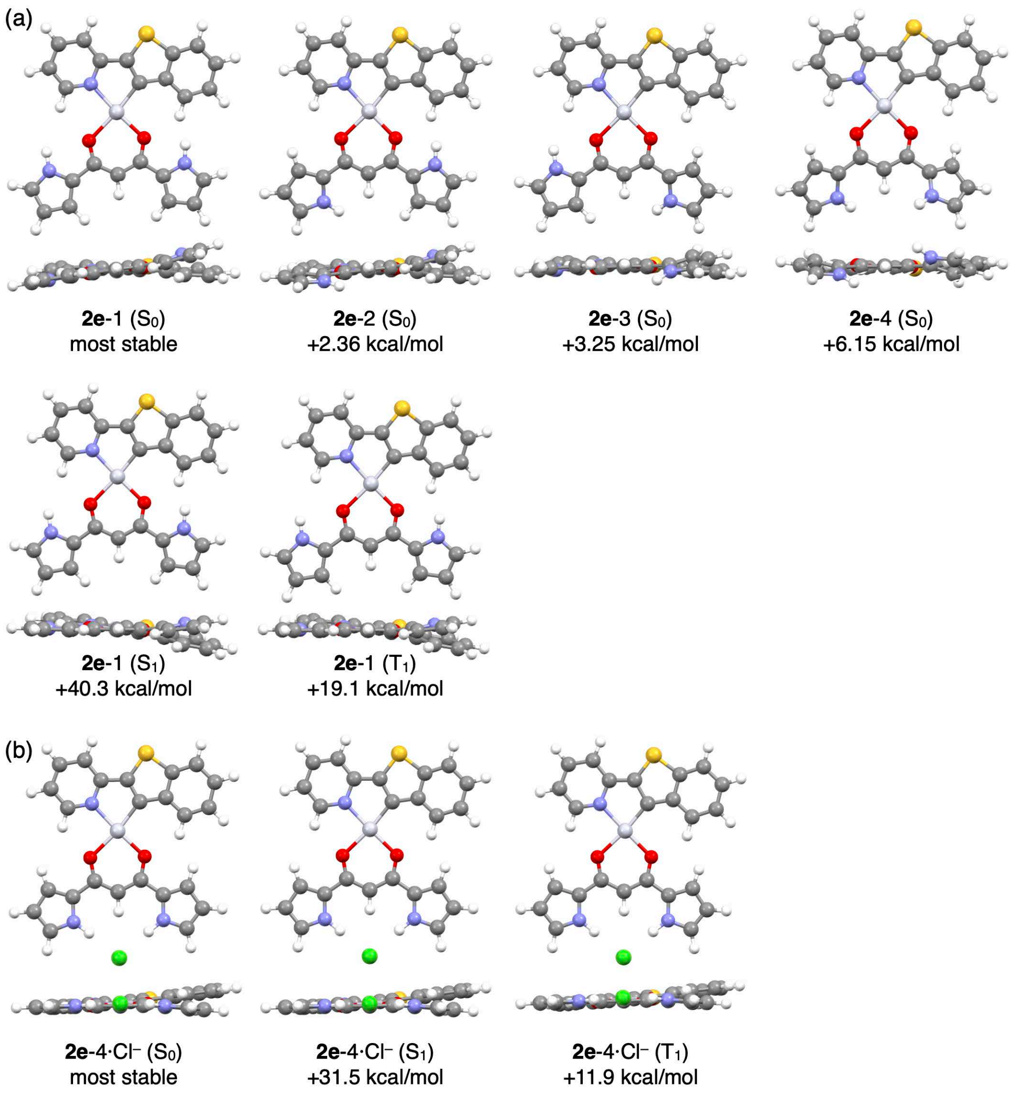

Fig. S42 Optimized structures and relative energies of (a) **2e** (four conformations) and (b) **2e**·Cl^- in the ground (S_0) state and singlet (S_1) and triplet (T_1) excited states.

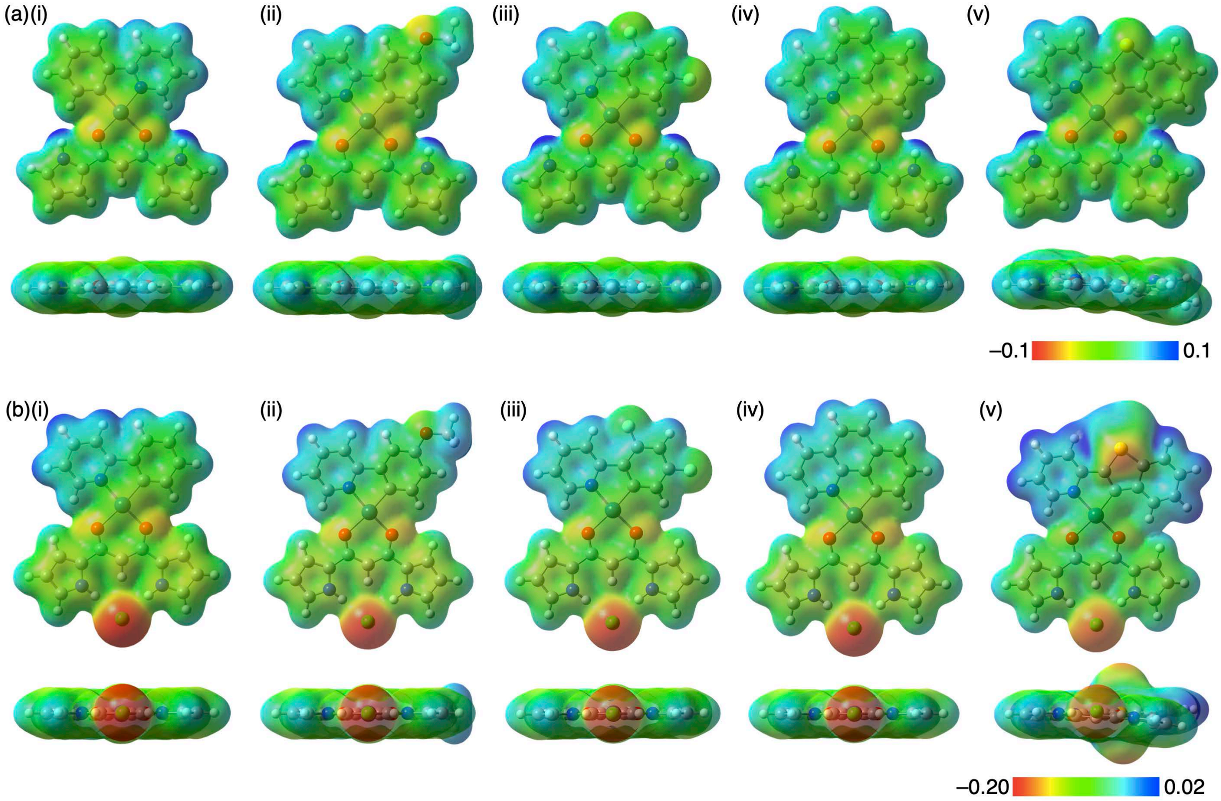

Fig. S43 ESP mapping (δ = 0.005) of (a) Pt^{II} complexes (i) **2a**, (ii) **2b**, (iii) **2c**, (iv) **2d**, and (v) **2e** and (b) their Cl^{-} complexes calculated at the CAM-B3LYP level by using 6-31+G(d,p) basis set was used for C, H, N, O, F, S, and Cl and the LanL2DZ basis set and associated effective core potentials were used for Pt in solution phase.

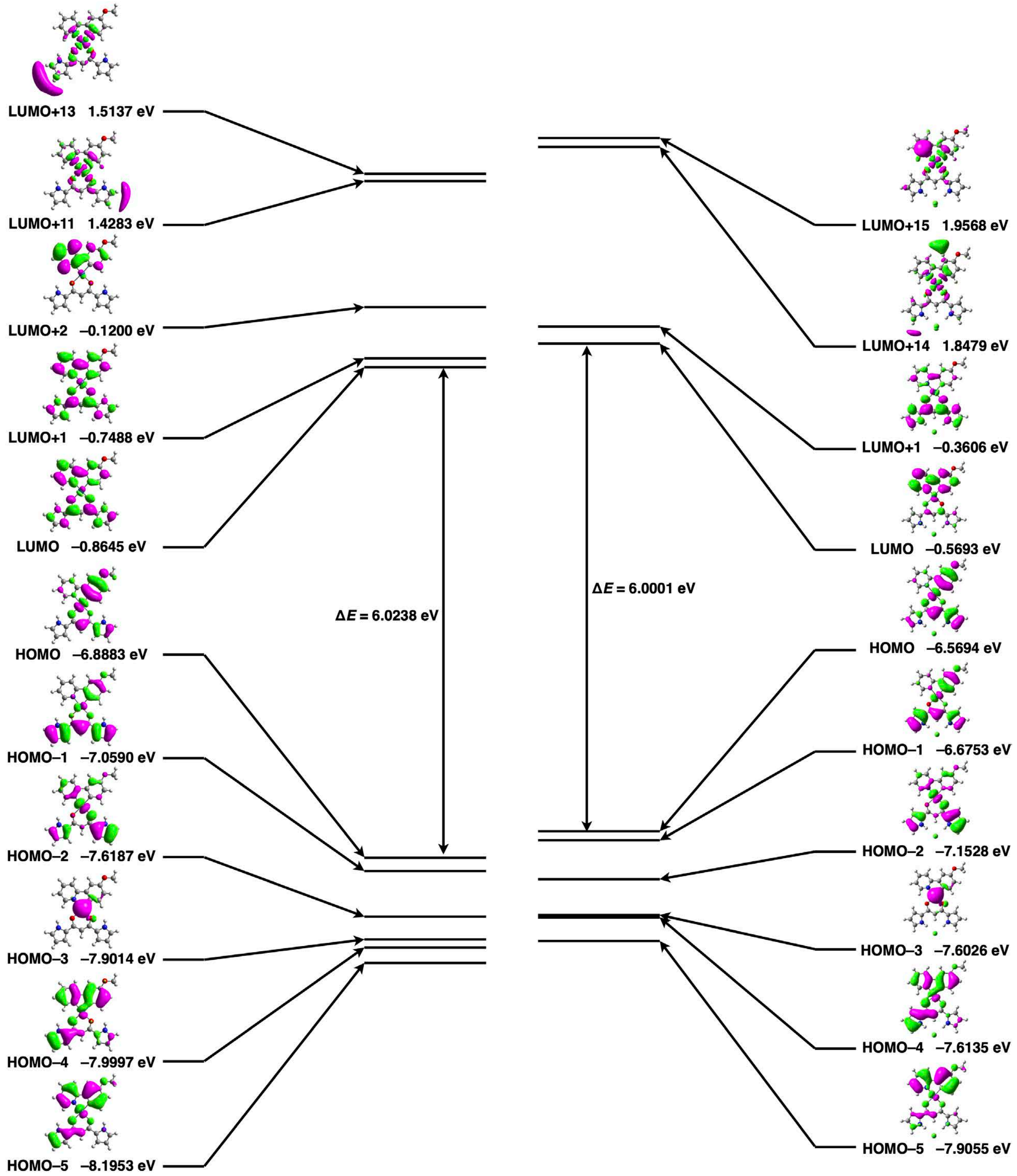

Fig. S44 Molecular orbitals (HOMO/LUMO) of **2b** (left) and **2b**·Cl^- (right) estimated at the CAM-B3LYP level by using 6-31+G(d,p) basis set for C, H, N, O, and Cl and the LanL2DZ basis set and associated effective core potentials for Pt in CH_2Cl_2.

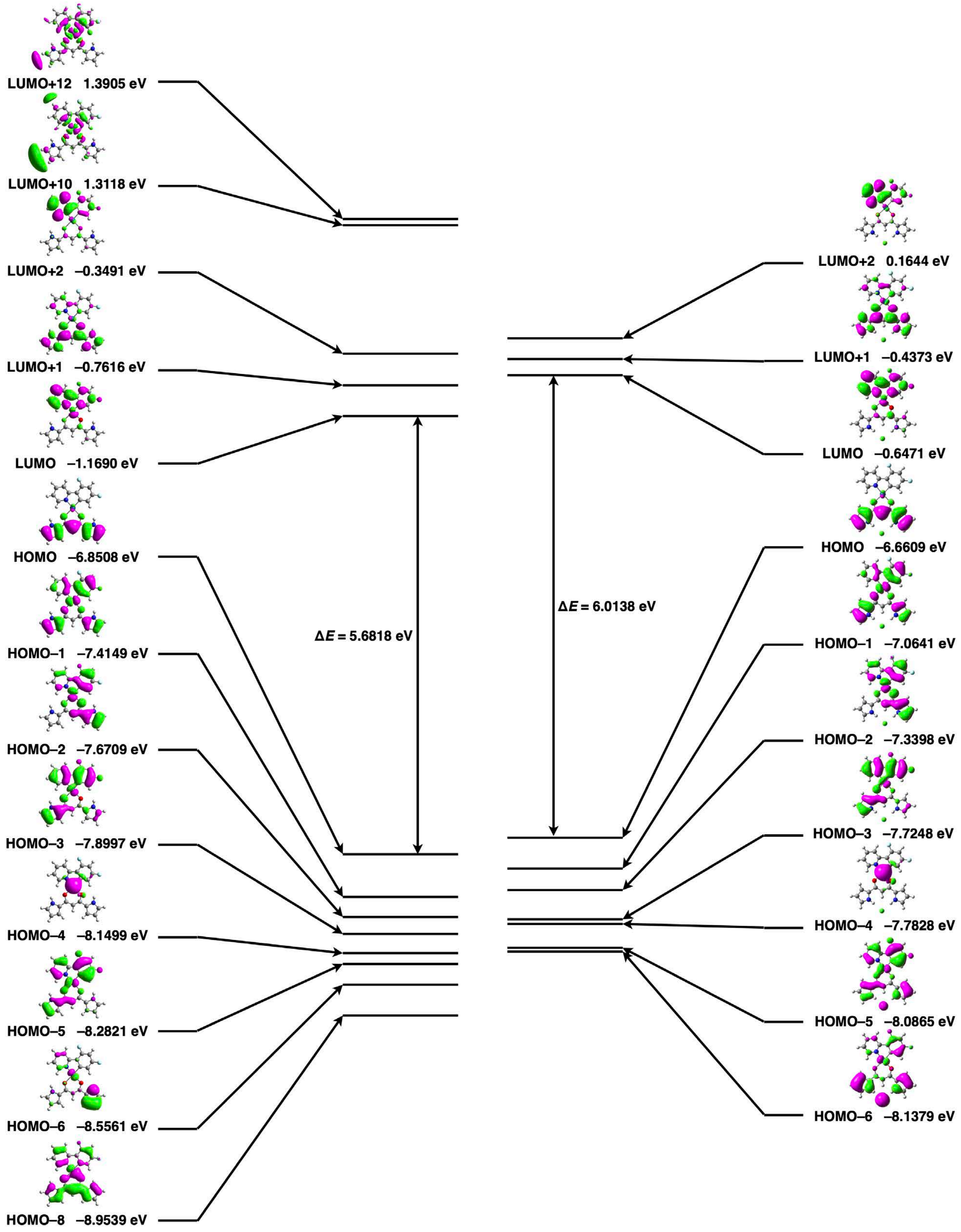

Fig. S45 Molecular orbitals (HOMO/LUMO) of **2c** (left) and **2c**·Cl^- (right) estimated at the CAM-B3LYP level by using 6-31+G(d,p) basis set for C, H, N, O, F, and Cl and the LanL2DZ basis set and associated effective core potentials for Pt in CH_2Cl_2.

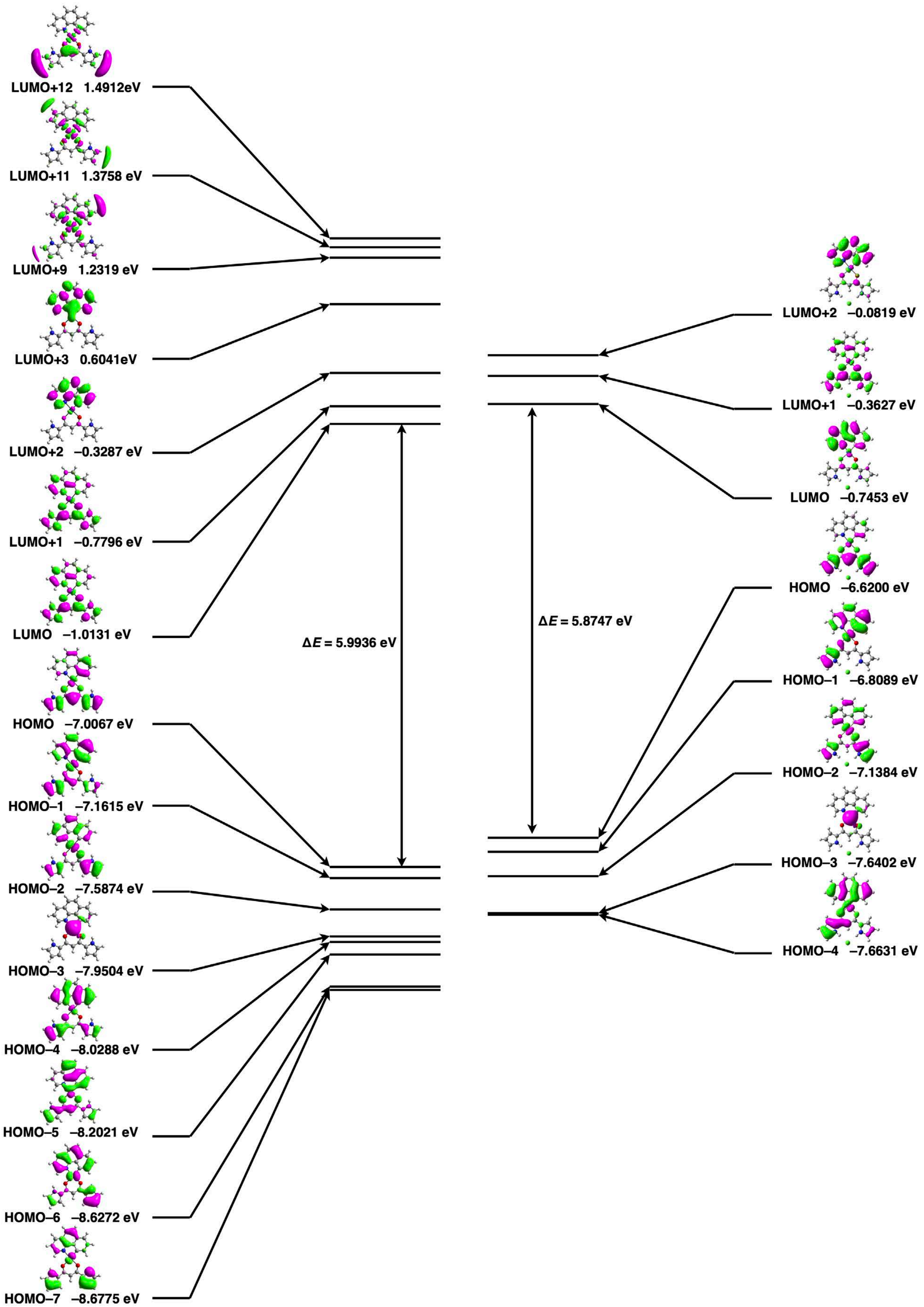

Fig. S46 Molecular orbitals (HOMO/LUMO) of **2d** (left) and **2d**·Cl^- (right) estimated at the CAM-B3LYP level by using 6-31+G(d,p) basis set for C, H, N, O, and Cl and the LanL2DZ basis set and associated effective core potentials for Pt in CH_2Cl_2.

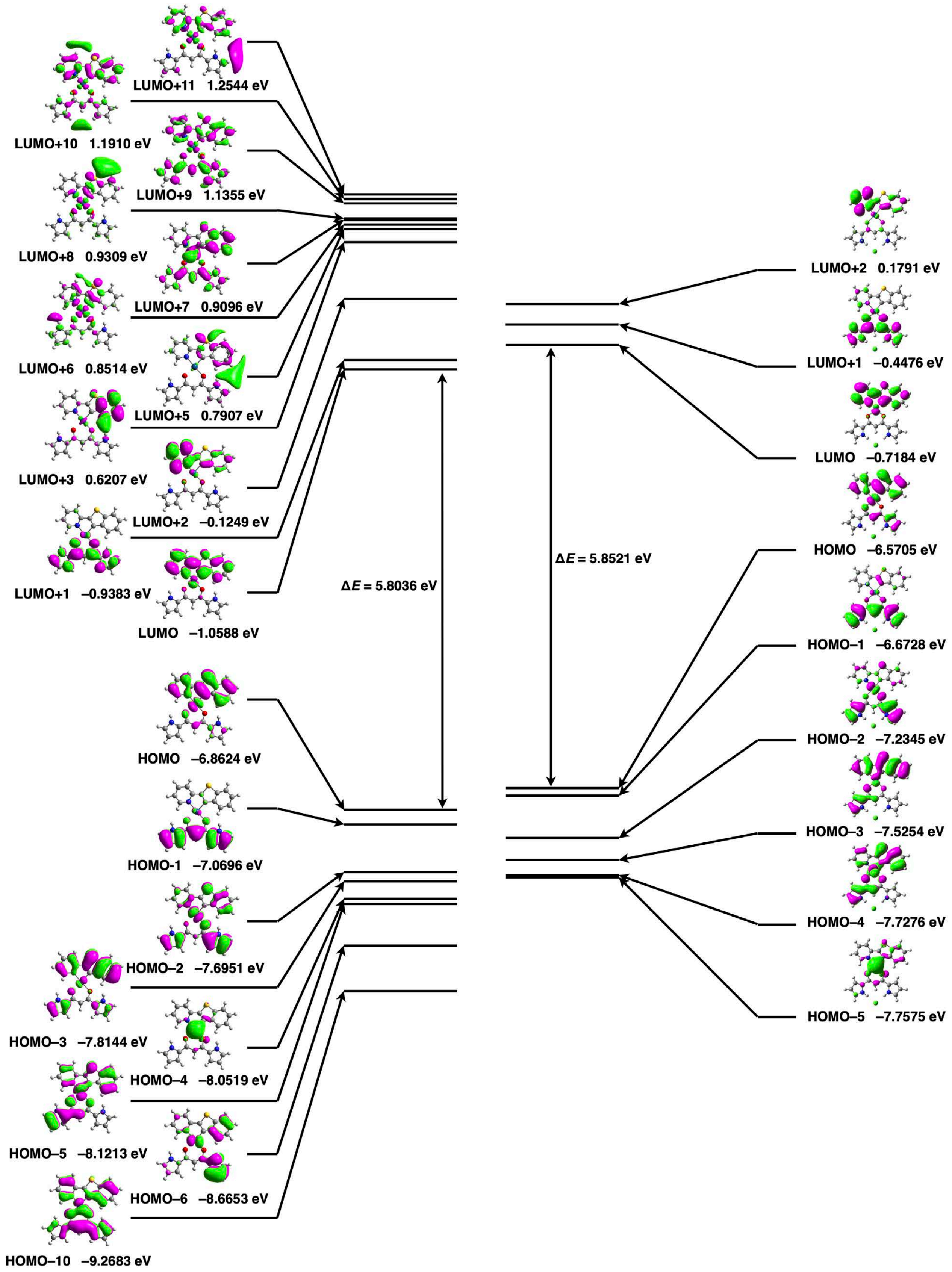

Fig. S47 Molecular orbitals (HOMO/LUMO) of **2e** (left) and **2e**·Cl^{-} (right) estimated at the CAM-B3LYP level by using 6-31+G(d,p) basis set for C, H, N, O, S, and Cl and the LanL2DZ basis set and associated effective core potentials for Pt in CH_2Cl_2.

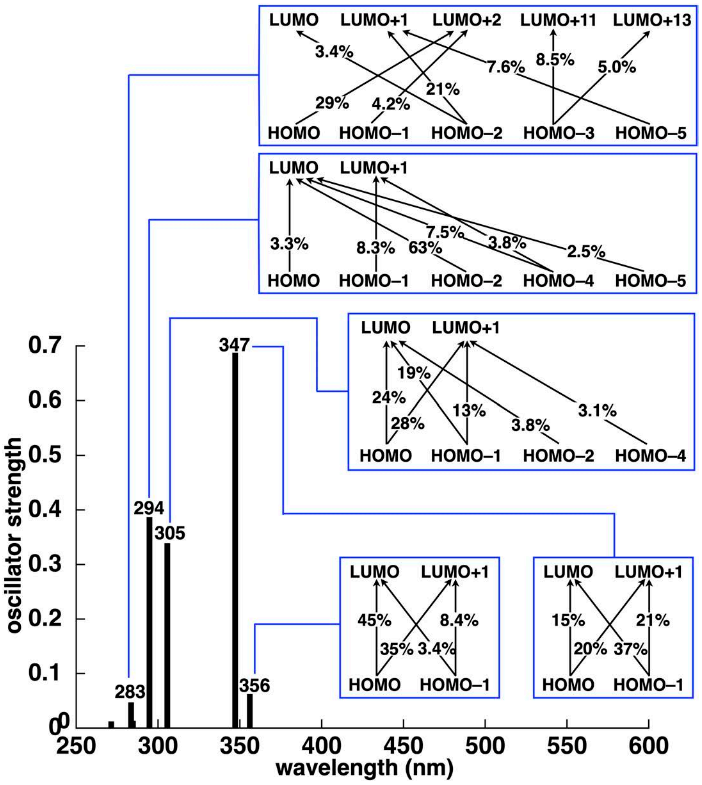

Fig. S48 TD-DFT-based UV/vis absorption stick spectrum of **2b** with the transitions correlated with molecular orbitals estimated at the CAM-B3LYP level by using 6-31+G(d,p) basis set for C, H, N, and O and the LanL2DZ basis set and associated effective core potentials for Pt in CH_2Cl_2.

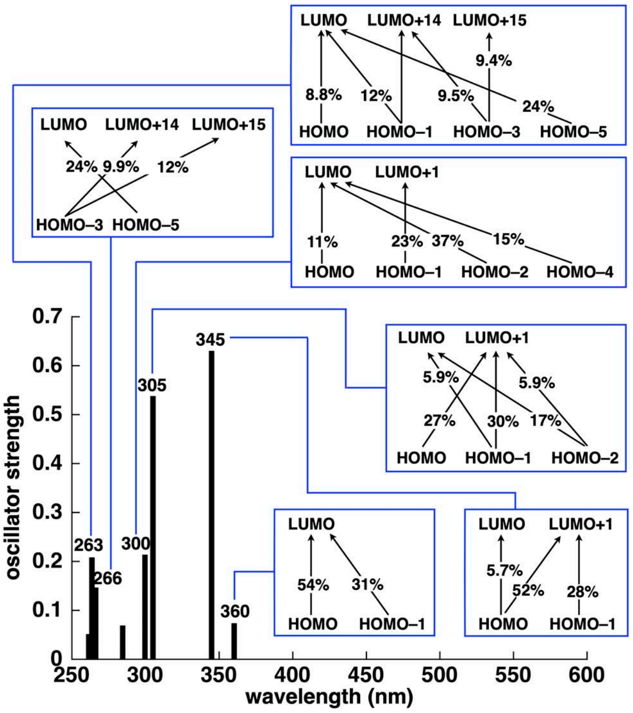

Fig. S49 TD-DFT-based UV/vis absorption stick spectrum of **2b**·Cl^- with the transitions correlated with molecular orbitals estimated at the CAM-B3LYP level by using 6-31+G(d,p) basis set for C, H, N, O, and Cl and the LanL2DZ basis set and associated effective core potentials for Pt in CH_2Cl_2.

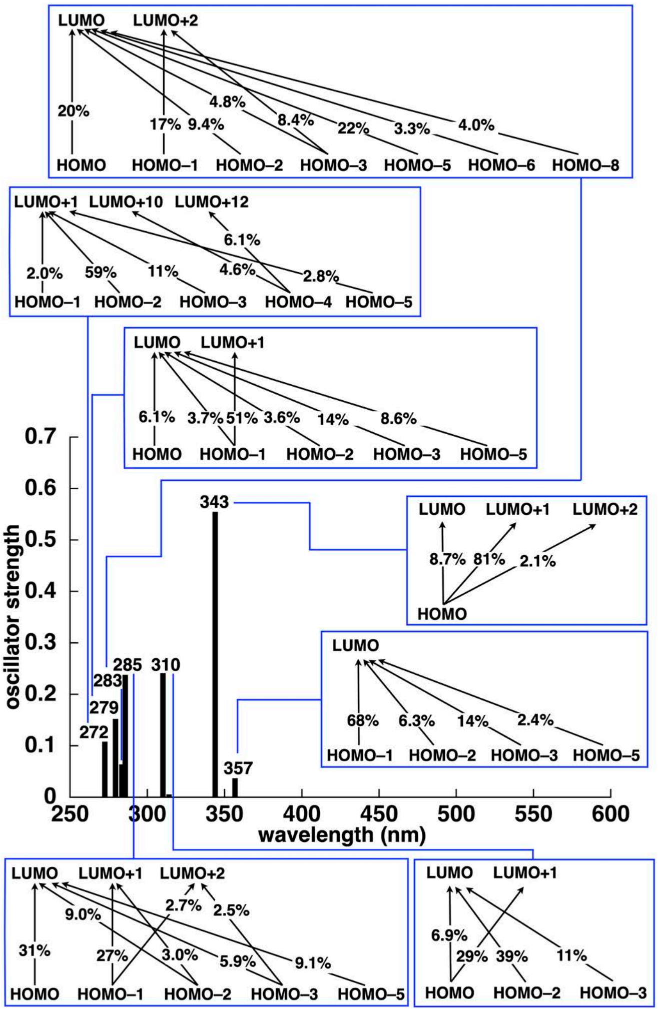

Fig. S50 TD-DFT-based UV/vis absorption stick spectrum of **2c** with the transitions correlated with molecular orbitals estimated at the CAM-B3LYP level by using 6-31+G(d,p) basis set for C, H, N, O, and F and the LanL2DZ basis set and associated effective core potentials for Pt in CH_2Cl_2.

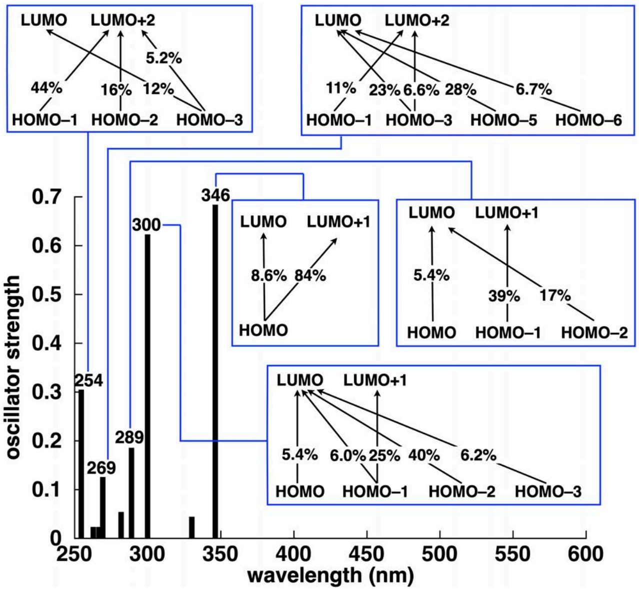

Fig. S51 TD-DFT-based UV/vis absorption stick spectrum of **2c**·Cl^- with the transitions correlated with molecular orbitals estimated at the CAM-B3LYP level by using 6-31+G(d,p) basis set for C, H, N, O, F, and Cl and the LanL2DZ basis set and associated effective core potentials for Pt in CH_2Cl_2.

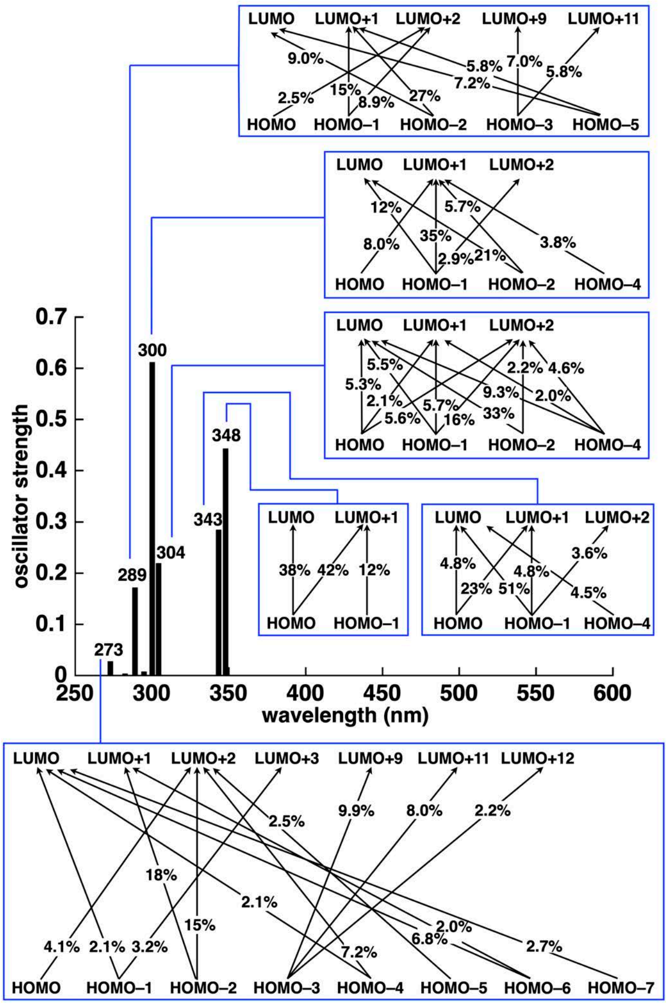

Fig. S52 TD-DFT-based UV/vis absorption stick spectrum of **2d** with the transitions correlated with molecular orbitals estimated at the CAM-B3LYP level by using 6-31+G(d,p) basis set for C, H, N, and O and the LanL2DZ basis set and associated effective core potentials for Pt in CH_2Cl_2.

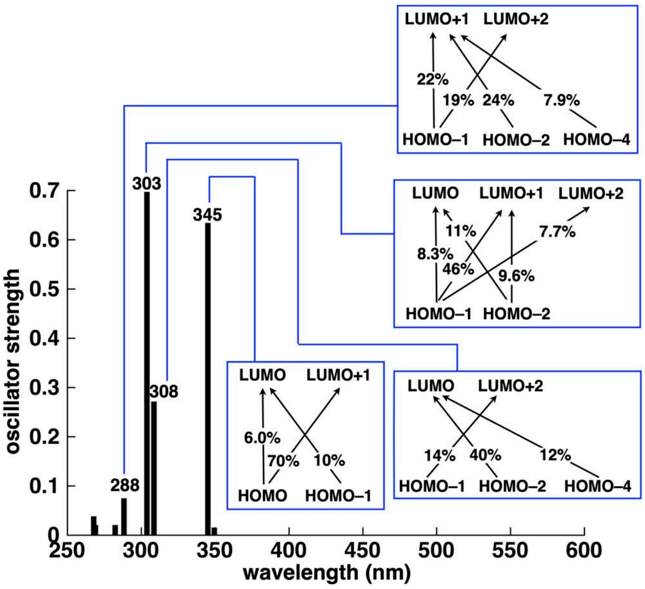

Fig. S53 TD-DFT-based UV/vis absorption stick spectrum of **2d**·Cl^- with the transitions correlated with molecular orbitals estimated at the CAM-B3LYP level by using 6-31+G(d,p) basis set for C, H, N, O, and Cl and the LanL2DZ basis set and associated effective core potentials for Pt in CH_2Cl_2.

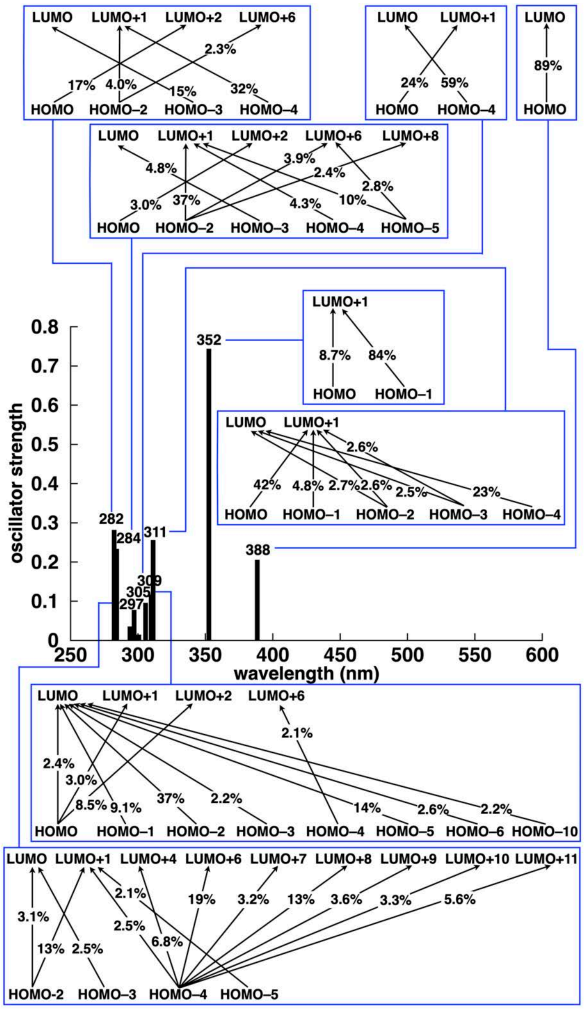

Fig. S54 TD-DFT-based UV/vis absorption stick spectrum of **2e** with the transitions correlated with molecular orbitals estimated at the CAM-B3LYP level by using 6-31+G(d,p) basis set for C, H, N, O, and S and the LanL2DZ basis set and associated effective core potentials for Pt in CH_2Cl_2.

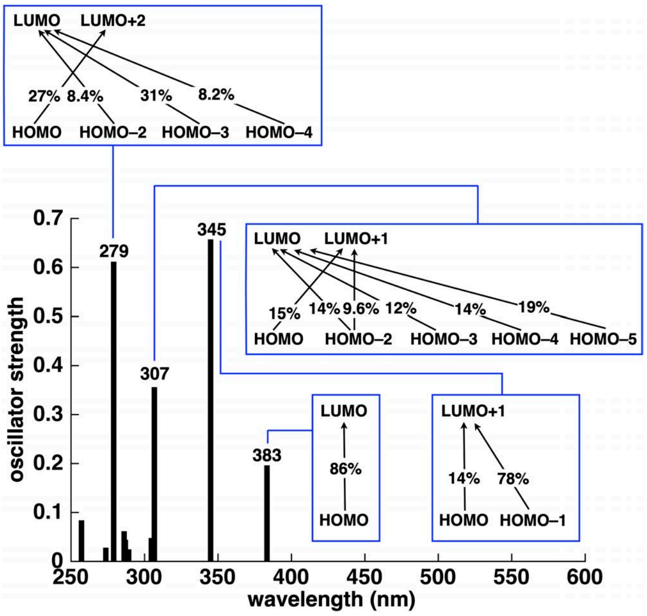

Fig. S55 TD-DFT-based UV/vis absorption stick spectrum of **2e**·Cl^- with the transitions correlated with molecular orbitals estimated at the CAM-B3LYP level by using 6-31+G(d,p) basis set for C, H, N, O, S, and Cl and the LanL2DZ basis set and associated effective core potentials for Pt in CH_2Cl_2.

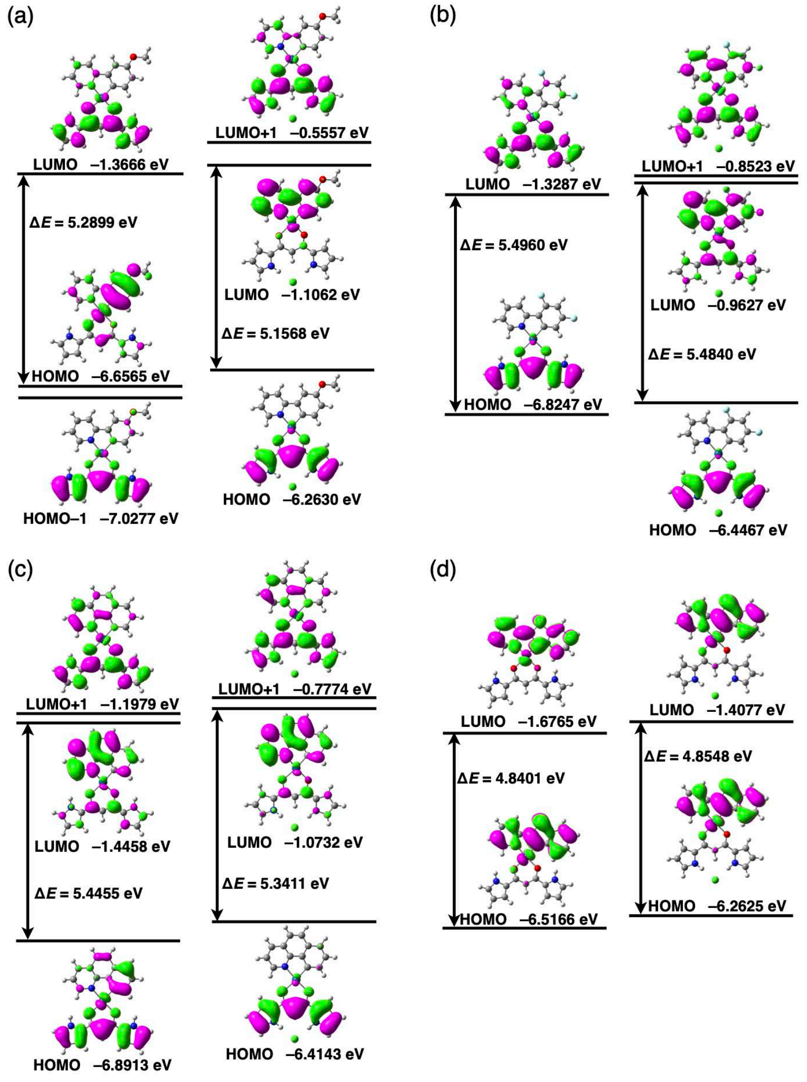

Fig. S56 Molecular orbitals (HOMO and LUMOs) of (a) **2b** and **2b**·Cl^-, (b) **2c** and **2c**·Cl^-, (c) **2d** and **2d**·Cl^-, and (d) **2e** and **2e**·Cl^- in the T_1 states. Molecular orbitals were estimated at the TD-PCM-M06-2X level by using 6-31+G(d,p) basis set for C, H, N, O, F, S, and Cl and the LanL2DZ basis set and associated effective core potentials for Pt in solution phase (CH_2Cl_2) based on the T_1 optimized structures (in vacuum). The theoretical emission maxima (λ_{max}) were estimated at 499, 524, 495, and 714 nm for **2b**–**e** and at 486, 501, 499, and 703 nm for **2b**–**e**·Cl^-. The phosphorescence emissions of **2c**,**e** were mainly derived from the LUMO-to-HOMO (84% and 96%, respectively) transitions, whereas those of **2b**,**d** were ascribed to the LUMO-to-HOMO–1 (68%) and LUMO+1-to-HOMO (60%) transitions, respectively. Furthermore, phosphorescence emissions of **2c**,**d**·Cl^- were mainly derived from the LUMO+1-to-HOMO (67% and 71%, respectively) transitions, whereas those of **2a**·Cl^- and **2e**·Cl^- were mainly derived from the LUMO+1-to-HOMO (82%) and LUMO-to-HOMO (96%), respectively.

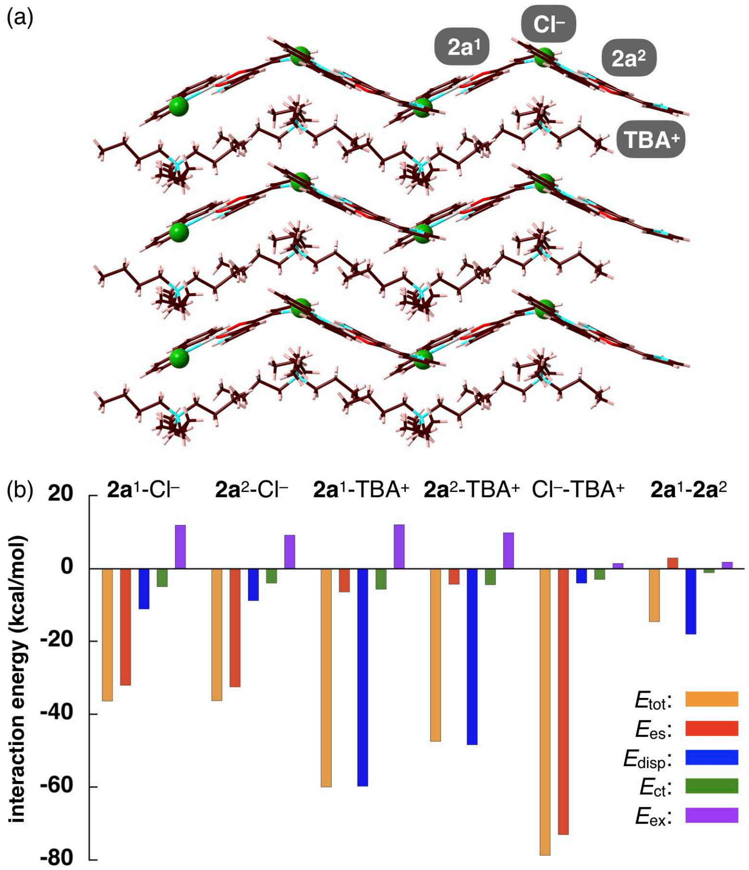

Fig. S57 Single-crystal X-ray structure of **2a**·Cl^{-}-TBA^{+} for the EDA calculations (Table S2): (a) packing structure and (b) intermolecular interaction energies (kcal/mol) between selected ions estimated at the FMO2-MP2 method using mixed basis sets including NOSeC-V-TZP with MCP for Pt and NOSeC-V-DZP with MCP for the other atoms.[S17–19] The labels (**2a**^{1}-Cl^{-}, **2a**^{2}-Cl^{-}, **2a**^{1}-TBA^{+}, **2a**^{2}-TBA^{+}, Cl^{-}-TBA^{+}, and **2a**^{1}-**2a**^{2}) correspond to the fragments shown in Table S2.

Table S2 Energies between selected fragments in **2a**·Cl^{-}-$TATA^{+}$ (Fig. S57) estimated by EDA calculations based on an FMO2-MP2 using mixed basis sets including NOSeC-V-TZP with MCP for Pt and NOSeC-V-DZP with MCP for the other atoms.[S17–19]

fragments	total interaction energy (E_{tot}) (kcal/mol)	electrostatic interaction energy (E_{es}) (kcal/mol)	dispersion interaction energy (E_{disp}) (kcal/mol)	charge-transfer interaction energy (E_{ct+mix}) (kcal/mol)	exchange repulsion interaction energy (E_{ex}) (kcal/mol)
**2a**^{1}-Cl^{-}	–36.403	–32.072	–11.152	–4.991	11.813
**2a**^{2}-Cl^{-}	–36.291	–32.498	–8.823	–4.067	9.097
**2a**^{1}-TBA^{+}	–59.976	–6.484	–59.791	–5.698	11.998
**2a**^{2}-TBA^{+}	–47.468	–4.343	–48.390	–4.496	9.761
Cl^{-}-TBA^{+}	–78.758	–73.041	–4.056	–3.023	1.362
**2a**^{1}-**2a**^{2}	–14.635	2.878	–18.052	–1.187	1.725

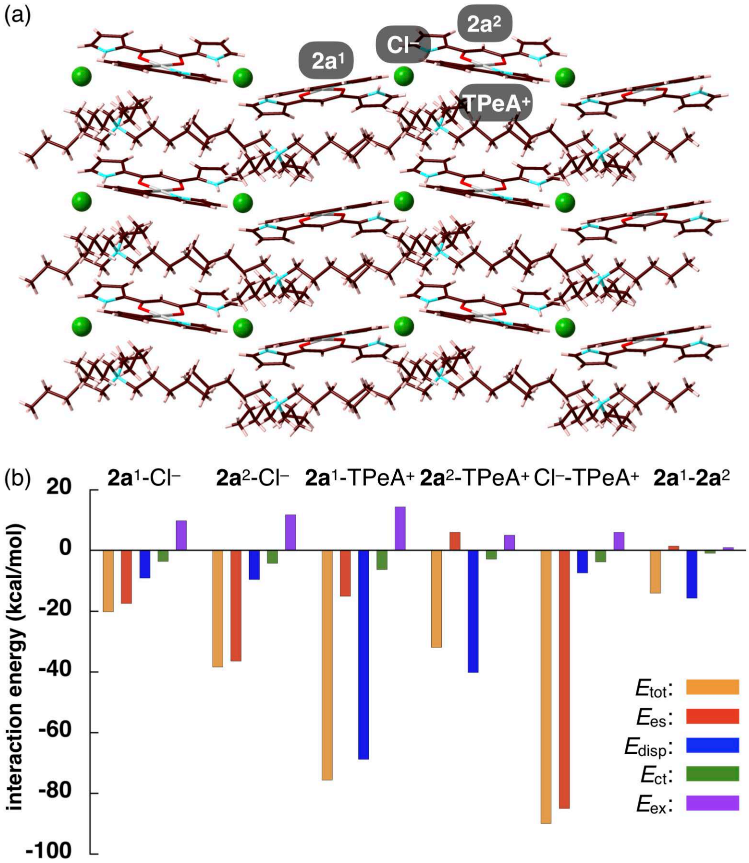

Fig. S58 Single-crystal X-ray structure of **2a**·Cl⁻-TPeA⁺ for the EDA calculations (Table S3): (a) packing structure and (b) intermolecular interaction energies (kcal/mol) between selected ions estimated at the FMO2-MP2 method using mixed basis sets including NOSeC-V-TZP with MCP for Pt and NOSeC-V-DZP with MCP for the other atoms.[S17–19] The labels (**2a**^{1}-Cl⁻, **2a**^{2}-Cl⁻, **2a**^{1}-TPeA⁺, **2a**^{2}-TPeA⁺, Cl⁻-TPeA⁺, and **2a**^{1}-**2a**^{2}) correspond to the fragments shown in Table S3.

Table S3 Energies between selected fragments in **2a**·Cl⁻-TATA⁺ (Fig. S58) estimated by EDA calculations based on an FMO2-MP2 using mixed basis sets including NOSeC-V-TZP with MCP for Pt and NOSeC-V-DZP with MCP for the other atoms.[S17–19]

fragments	total interaction energy (E_{tot}) (kcal/mol)	electrostatic interaction energy (E_{es}) (kcal/mol)	dispersion interaction energy (E_{disp}) (kcal/mol)	charge-transfer interaction energy (E_{ct+mix}) (kcal/mol)	exchange repulsion interaction energy (E_{ex}) (kcal/mol)
**2a**^{1}-Cl⁻	–20.167	–17.449	–9.009	–3.533	9.824
**2a**^{2}-Cl⁻	–38.371	–36.434	–9.528	–4.166	11.756
**2a**^{1}-TPeA⁺	–75.633	–14.994	–68.763	–6.230	14.354
**2a**^{2}-TPeA⁺	–31.903	6.035	–40.154	–2.823	5.039
Cl⁻-TPeA⁺	–89.970	–85.004	–7.315	–3.704	6.053
**2a**^{1}-**2a**^{2}	–14.038	1.475	–15.663	–0.879	1.029

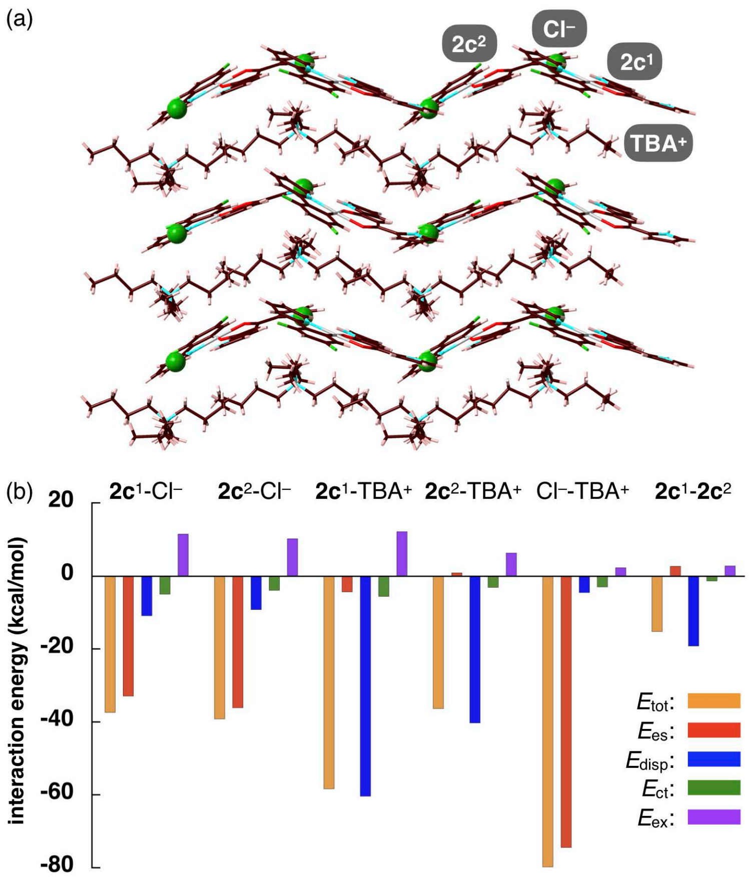

Fig. S59 Single-crystal X-ray structure of **2c**·Cl$^-$-TBA$^+$ for the EDA calculations (Table S4): (a) packing structure and (b) intermolecular interaction energies (kcal/mol) between selected ions estimated at the FMO2-MP2 method using mixed basis sets including NOSeC-V-TZP with MCP for Pt and NOSeC-V-DZP with MCP for the other atoms.[S17–19] The labels (**2c**^{1}-Cl$^-$, **2c**^{2}-Cl$^-$, **2c**^{1}-TPeA$^+$, **2c**^{2}-TBA$^+$, Cl$^-$-TBA$^+$, and **2c**^{1}-**2c**^{2}) correspond to the fragments shown in Table S4.

Table S4 Energies between selected fragments in **2c**·Cl$^-$-TATA$^+$ (Fig. S59) estimated by EDA calculations based on an FMO2-MP2 using mixed basis sets including NOSeC-V-TZP with MCP for Pt and NOSeC-V-DZP with MCP for the other atoms.[S17–19]

fragments	total interaction energy (E_{tot}) (kcal/mol)	electrostatic interaction energy (E_{es}) (kcal/mol)	dispersion interaction energy (E_{disp}) (kcal/mol)	charge-transfer interaction energy (E_{ct+mix}) (kcal/mol)	exchange repulsion interaction energy (E_{ex}) (kcal/mol)
**2c**^{1}-Cl$^-$	–37.388	–32.924	–10.888	–5.007	11.431
**2c**^{2}-Cl$^-$	–39.177	–36.173	–9.227	–3.953	10.176
**2c**^{1}-TBA$^+$	–58.368	–4.415	–60.399	–5.628	12.074
**2c**^{2}-TBA$^+$	–36.375	0.807	–40.283	–3.139	6.240
Cl$^-$-TBA$^+$	–79.803	–74.436	–4.534	–3.045	2.213
**2c**^{1}-**2c**^{2}	–15.252	2.594	–19.192	–1.365	2.711

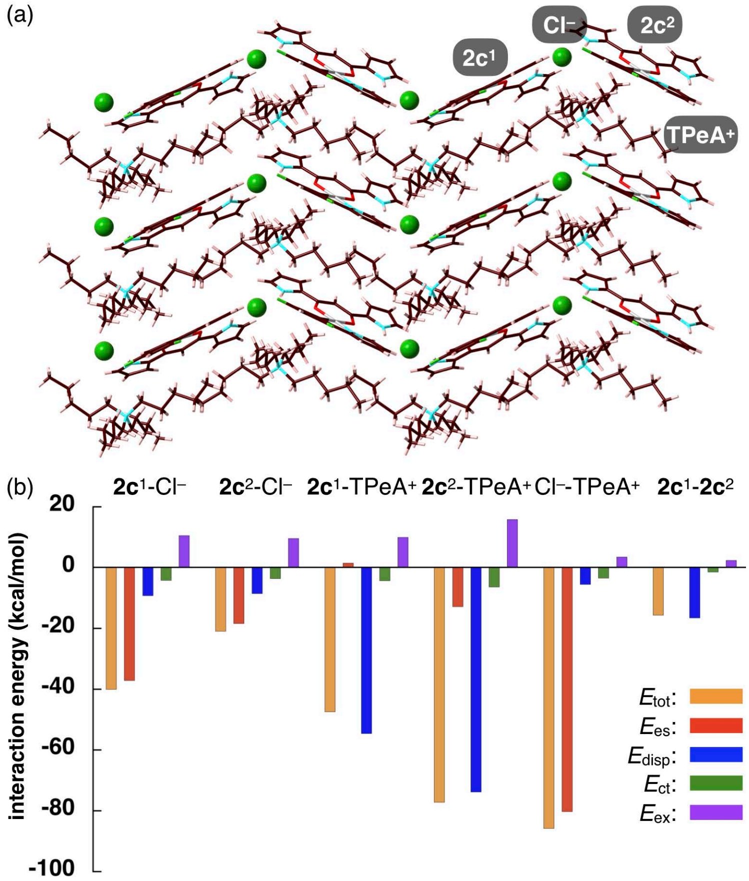

Fig. S60 Single-crystal X-ray structure of **2c**·Cl$^-$-TPeA$^+$ for the EDA calculations (Table S5): (a) packing structure and (b) intermolecular interaction energies (kcal/mol) between selected ions estimated at the FMO2-MP2 method using mixed basis sets including NOSeC-V-TZP with MCP for Pt and NOSeC-V-DZP with MCP for the other atoms.[S17–19] The labels (**2c**^{1}-Cl$^-$, **2c**^{2}-Cl$^-$, **2c**^{1}-TPeA$^+$, **2c**^{2}-TPeA$^+$, Cl$^-$-TPeA$^+$, and **2c**^{1}-**2c**^{2}) correspond to the fragments shown in Table S5.

Table S5 Energies between selected fragments in **2a**·Cl$^-$-TATA$^+$ (Fig. S60) estimated by EDA calculations based on an FMO2-MP2 using mixed basis sets including NOSeC-V-TZP with MCP for Pt and NOSeC-V-DZP with MCP for the other atoms.[S17–19]

fragments	total interaction energy (E_{tot}) (kcal/mol)	electrostatic interaction energy (E_{es}) (kcal/mol)	dispersion interaction energy (E_{disp}) (kcal/mol)	charge-transfer interaction energy (E_{ct+mix}) (kcal/mol)	exchange repulsion interaction energy (E_{ex}) (kcal/mol)
**2c**^{1}-Cl$^-$	–40.065	–37.183	–9.233	–4.171	10.522
**2c**^{2}-Cl$^-$	–20.966	–18.402	–8.530	–3.615	9.582
**2c**^{1}-TBA$^+$	–47.492	1.462	–54.616	–4.286	9.948
**2c**^{2}-TBA$^+$	–77.201	–12.859	–73.828	–6.356	15.843
Cl$^-$-TBA$^+$	–85.846	–80.270	–5.551	–3.485	3.460
**2c**^{1}-**2c**^{2}	–15.696	–0.107	–16.587	–1.395	2.394

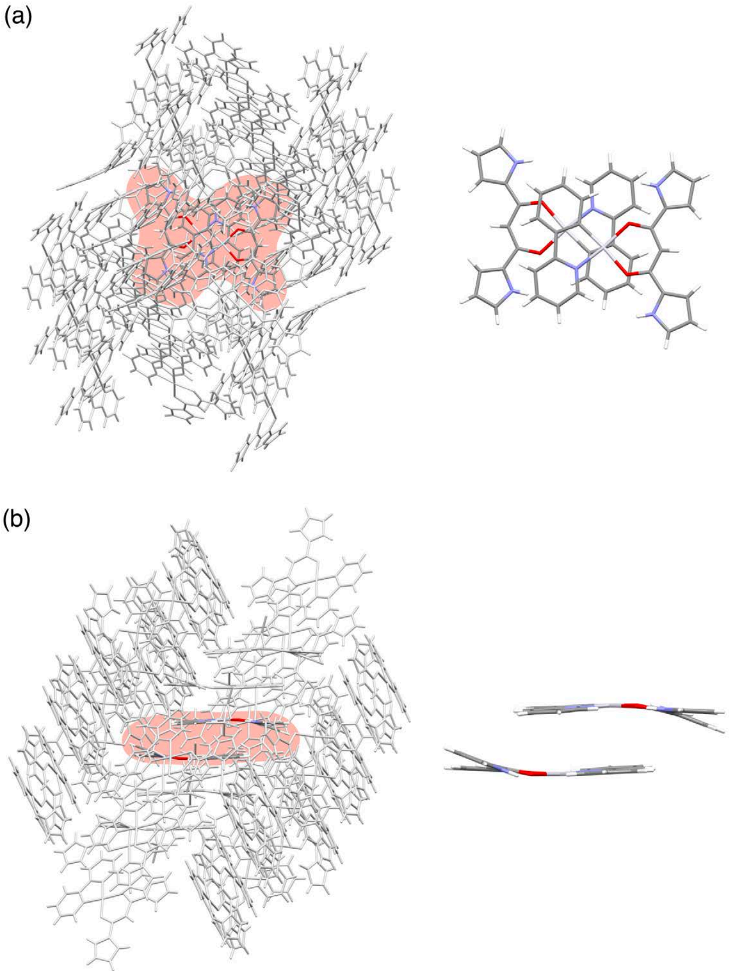

Fig. S61 (a) Top and (b) side views with enlarged central part as shown by red shade of a computational model for solid-state **2a** obtained by cutting out the 1 × 1 × 2 unit cell. The central dimer and surroundings were treated as the QM region at CAM-B3LYP/6-31+G(d,p) with the LanL2DZ basis set for Pt and MM region at UFF level, respectively. The QM region has C_i site symmetry.

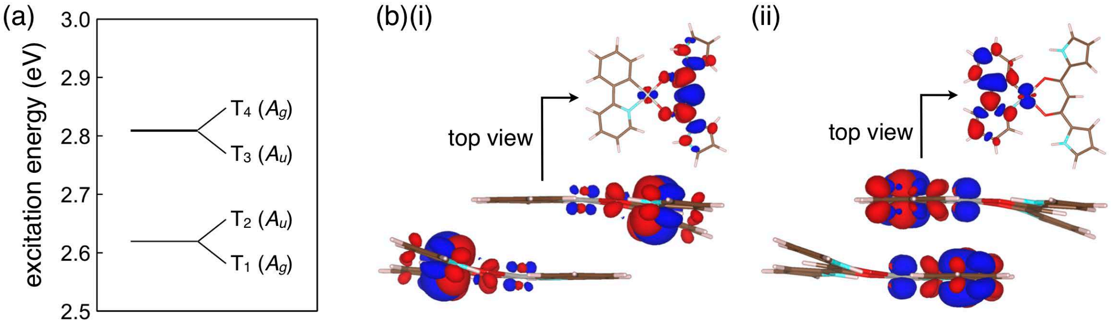

Fig. S62 (a) Calculated energy levels of excited states at the S_0 optimized structure for solid-state **2a** and (b)(i) electron density differences between S_0 (A_g) and T_1 (A_g) as well as (ii) S_0 (A_g) and T_4 (A_g) at the S_0 optimized structure (isosurface value: 5 × 10^{-4} a.u.). The electron density difference between T_2 (A_u) and S_0 (A_g) is similar to that between T_1 (A_g) and S_0 (A_g) due to the pseudo-degeneracy of T_1 and T_2. The electron density difference between T_3 (A_u) and S_0 (A_g) is similar to that between T_4 (A_g) and S_0 (A_g). Only the QM region is shown for simplicity. The red/blue region is positive/negative.

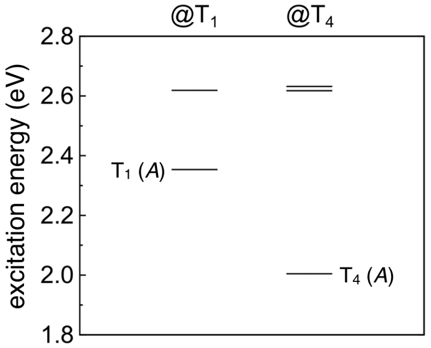

Fig. S63 Energy levels of excited triplet states at the T_1 and T_4 optimized structures for solid-state **2a**. T_4 becomes the lowest triplet after the geometry optimization. The total energy of T_4 is lower than that of T_1 by 0.06 eV at their optimized structures.

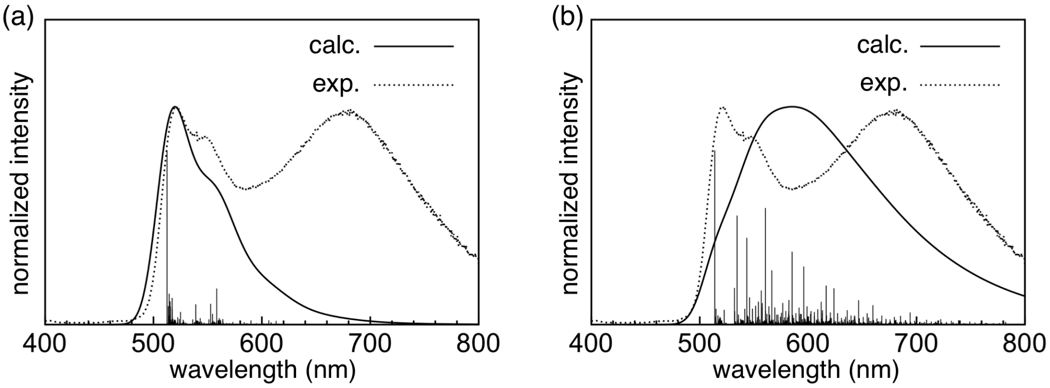

Fig. S64 Calculated phosphorescence spectra[S15] from (a) T_1 and (b) T_4 for solid-state **2a**, compared with the experimental spectrum in Fig. S86. Vertical lines represent the intensities of the Franck-Condon factors. The broadening of the density of final vibronic states was expressed using the Gaussian function with a linewidth of 500 cm^{-1}.

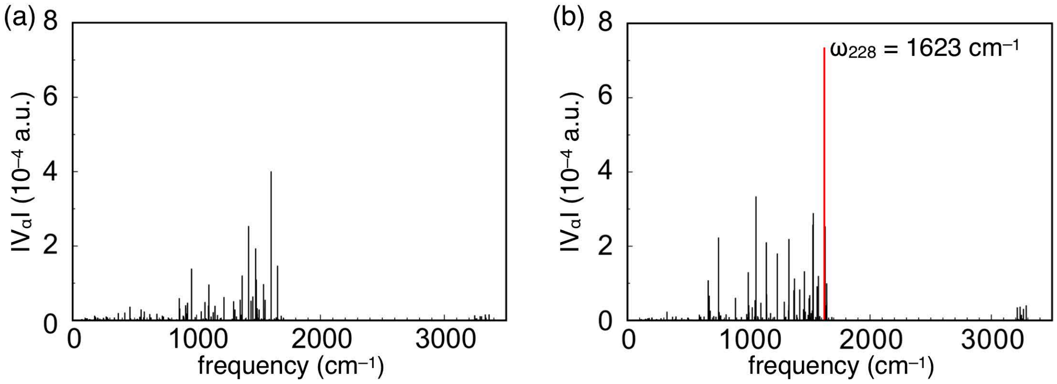

Fig. S65 Diagonal vibronic coupling constants (VCCs) of S_0 at the (a) T_1 and (b) T_4 optimized structures for solid-state **2a**. The diagonal VCC of vibrational mode 226 for S_0 at the T_4 optimized structure was the largest, which was analyzed by vibronic coupling density (VCD) in Fig. S66.

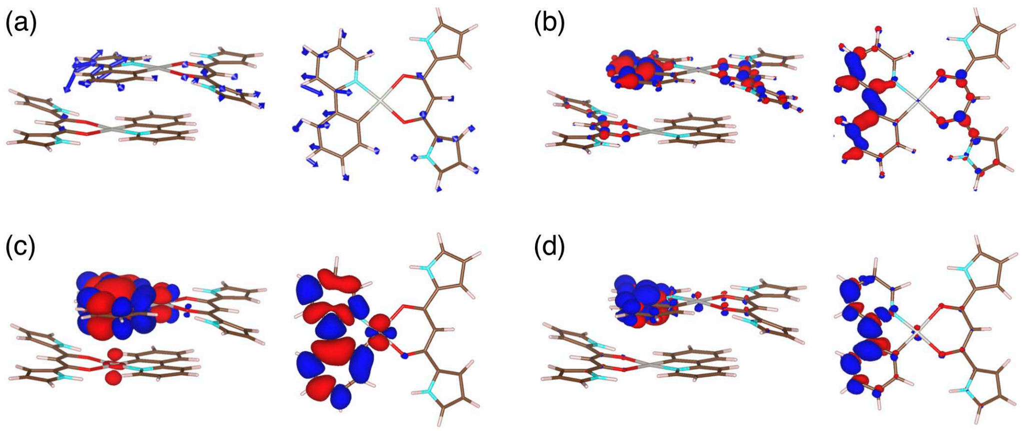

Fig. S66 Diagonal vibronic coupling density of S_0 for vibrational mode 226 at the T_4 optimized structure for solid-state **2a**: (a) vibrational mode 226, (b) potential derivative of mode 226, $v_\alpha(\boldsymbol{x})$, (c) electron density difference between S_0 and T_4, $\Delta\rho_{nm}(\boldsymbol{x})$, and (d) diagonal VCD, $\eta_{n,\alpha}(\boldsymbol{x})$. Isosurface values of $v_\alpha(\boldsymbol{x})$, $\Delta\rho_{nm}(\boldsymbol{x})$, and $\eta_{n,\alpha}(\boldsymbol{x})$ are 5×10^{-3}, 5×10^{-4}, and 3×10^{-6} a.u., respectively. The red/blue region is positive/negative. A direction of the vibrational mode was taken so that the VCC was negative.

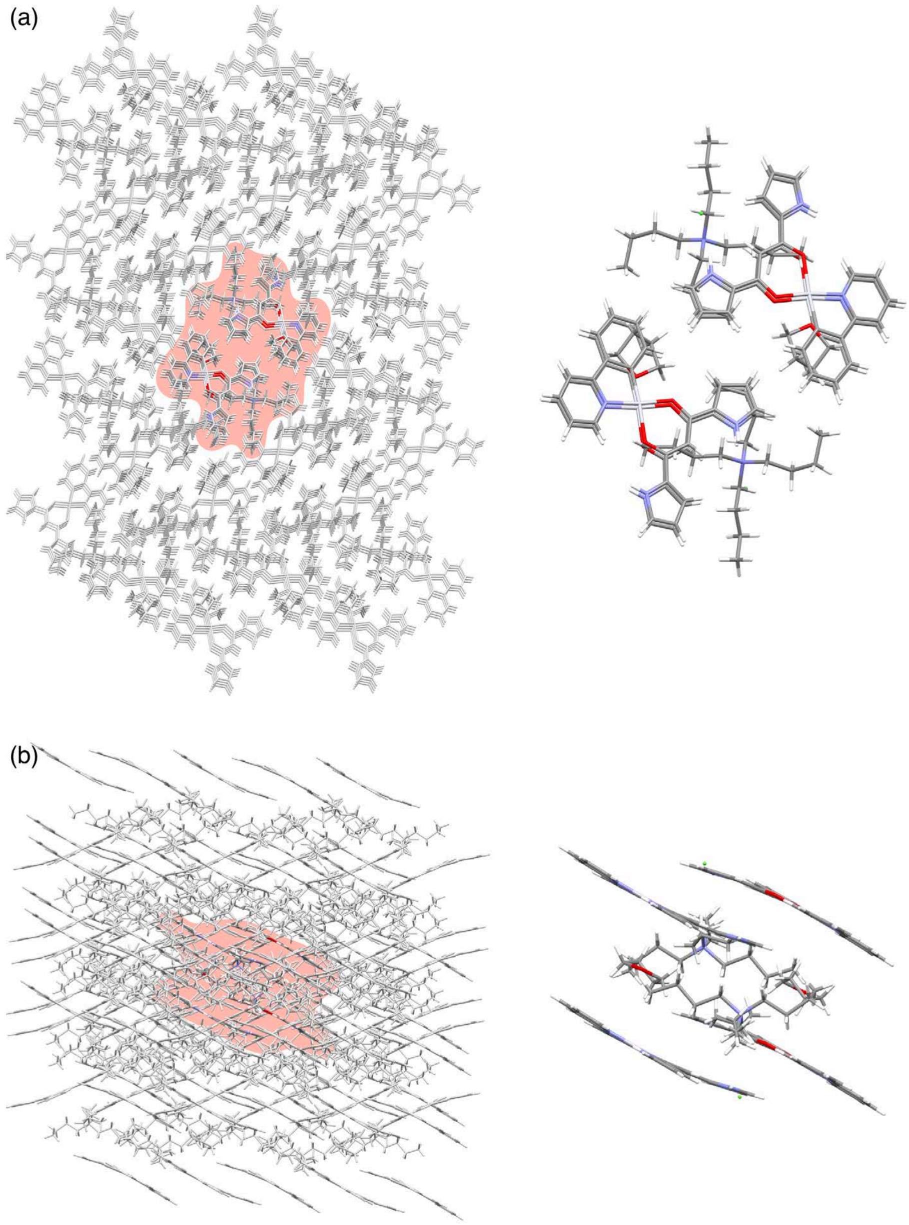

Fig. S67 (a) Top and (b) side views with enlarged center part as shown by red shade of a computational model for **2a**·Cl^--TBA^+ obtained by cutting out the 3 × 2 × 2 unit cell. The central four **2a**, two Cl^-, two TBA^+, and two THF were treated as the QM region at CAM-B3LYP/3-21G(d,p) with the LanL2DZ basis set for Pt, whereas the surroundings were treated as the MM region at UFF level. The QM region has C_i site symmetry.

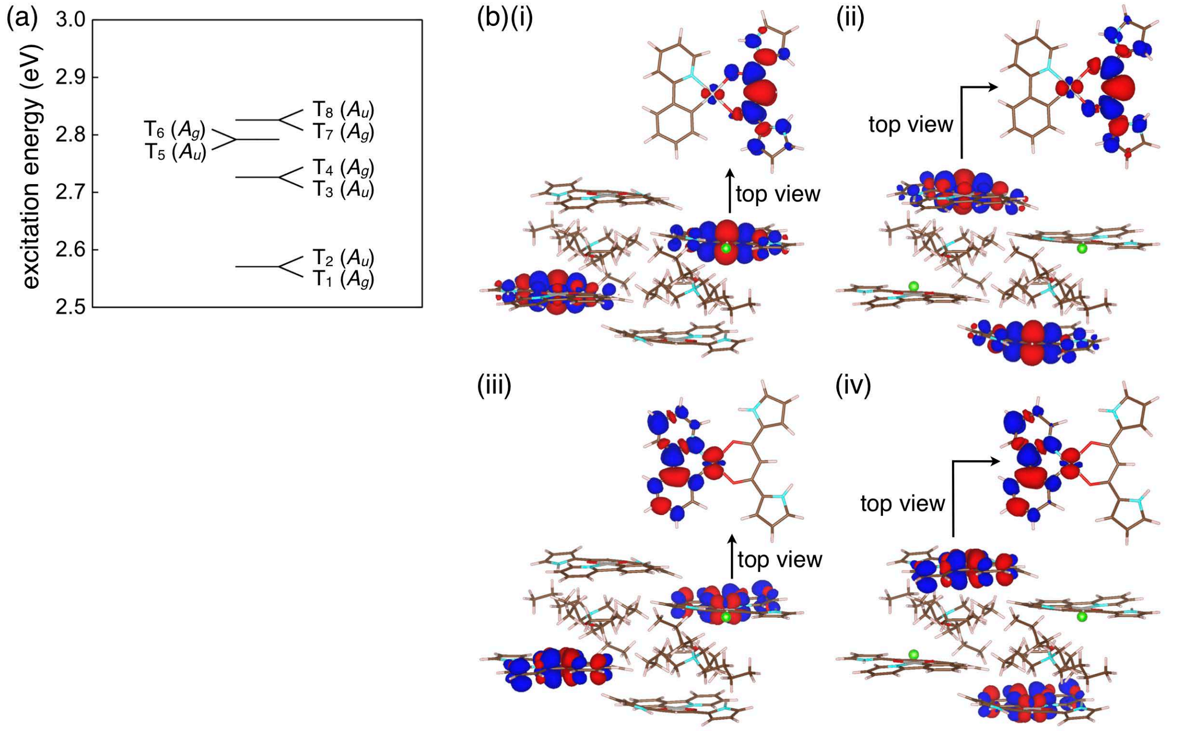

Fig. S68 (a) Calculated energy levels of excited triplet states at the S_0 optimized structure for **2a**·Cl$^-$-TBA$^+$. Electron density differences between (b) S_0 (A_g) and T_1 (A_g), (c) S_0 (A_g) and T_4 (A_g), (d) S_0 (A_g) and T_6 (A_g), and (e) S_0 (A_g) and T_8 (A_g) at the S_0 optimized structure (isosurface value: 5×10^{-4} a.u). Only the QM region is shown for simplicity. The red/blue region is positive/negative.

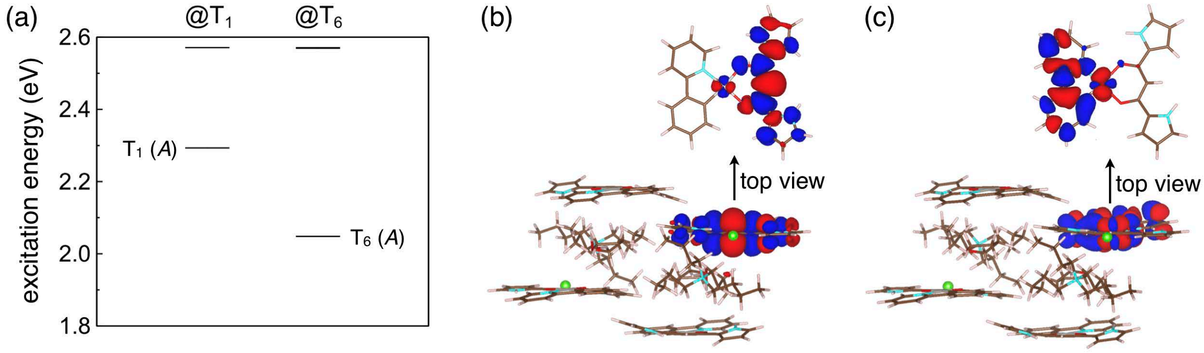

Fig. S69 (a) Calculated energy levels of excited triplet states at the T_1 and T_6 optimized structure for **2a**·Cl$^-$-TBA$^+$. The total energy of T_6 is higher than that of T_1 by 0.01 eV at their optimized structures. Electron density differences (b) between S_0 and T_1 at the T_1 optimized structure and (c) between S_0 and T_6 at the T_6 optimized structure (isosurface value: 5×10^{-4} a.u). The red/blue region is positive/negative.

Cartesian Coordination of Optimized Structures

2b-1 (S_0)

(CAM-B3LYP/6-31+G(d,p) for C, H, N, and O and CAM-B3LYP/LanL2DZ for Pt)

-1396.3558006 hartree

C,2.1220037287,3.3537133773,0.0001066222
C,3.2666084498,4.1349088023,0.000499617
C,2.8579140785,5.4879236689,0.0001506871
C,1.4765526185,5.4977152527,-0.0003479914
N,1.0491661188,4.2076315408,-0.0003464895
H,0.0977482401,3.8728062349,-0.0006413938
H,4.2844532297,3.7733534932,0.0009602312
H,3.4978778886,6.3583935829,0.0002834826
H,0.7789192543,6.3219654377,-0.000717968
C,1.8870428994,1.909628907,0.0001630797
C,2.9813397735,1.042531979,0.0003427361
C,2.9459164375,-0.358537976,0.0003294925
H,3.9589026843,1.5033336207,0.0004220528
O,0.6390811942,1.5921266026,0.000019698
O,1.8941366114,-1.0888504375,0.0001248647
C,4.2134986899,-1.0932579319,0.0005056204
C,5.5429754429,-0.7029669593,0.0009086693
N,4.1965491779,-2.4645704495,0.0002603193
C,6.3324383575,-1.8756765405,0.0008883981
H,5.9078963986,0.3135671974,0.0011980328
C,5.4664938845,-2.9512104253,0.0004904202
H,3.3374892397,-2.9910788749,-0.0000730938

H,7.4114358287,-1.9308492053,0.0011475562
H,5.6631903591,-4.0130183954,0.0003472256
Pt,-0.0964296124,-0.2990236163,0.0000340569
C,-2.3394052172,-2.1095797701,-0.0000445142
C,-0.2989068282,-3.261748113,-0.0001781791
C,-2.9280096811,-0.769668561,0.0001143186
C,-3.0290321249,-3.3222882692,-0.000142959
C,-0.9345077162,-4.4899465604,-0.0002721396
H,0.778920424,-3.155486742,-0.0001778695
C,-4.2999148063,-0.5288660081,0.0002123929
C,-1.9853061995,0.2814683549,0.0002012964
C,-2.3273582367,-4.5163571,-0.0002590422
H,-0.3488580947,-5.4014661171,-0.0003525516
C,-4.7691216809,0.7808824376,0.0004044189
C,-2.4873188496,1.5786787595,0.0004055175
H,-2.8602220208,-5.4616933585,-0.0003348148
C,-3.8587212341,1.8388666161,0.0005063026
H,-1.7947304719,2.4132974721,0.000513714
H,-4.2037611655,2.8659661194,0.0006672604
N,-0.9807760218,-2.1085368947,-0.0000747136
H,-4.1119133912,-3.3192986286,-0.0001244398
H,-5.031524204,-1.3299105661,0.0001519209
O,-6.1283042481,0.9277787426,0.0004718723
C,-6.6599814705,2.2358182442,0.000878681
H,-6.3559819087,2.7930475452,-0.8935579522
H,-7.7439764386,2.1211327189,0.0009897177
H,-6.3557421772,2.7925827724,0.8955224967

2b-2 (S_0)

(CAM-B3LYP/6-31+G(d,p) for C, H, N, and O and CAM-B3LYP/LanL2DZ for Pt)

-1396.3499211 hartree
C,2.1094981013,3.3233443903,0.0000562453
C,1.1784267749,4.3452378315,-0.0024133541
C,1.8797637435,5.5720908996,-0.0013421968
C,3.2251609529,5.2706508399,0.0019170598
N,3.3553640352,3.9139375384,0.002743794
H,4.2345220158,3.4246289149,0.0057933951
H,0.1095096653,4.1978053217,-0.0049209041
H,1.4541446546,6.5650645441,-0.0027822911
H,4.0923503768,5.914380589,0.003811561
C,1.8719033486,1.8772391441,-0.0001376972
C,2.9570239042,0.9897132822,-0.0002477232
C,2.9046078504,-0.4119306297,-0.0000831994
H,3.951190199,1.4145565624,-0.0008954665
O,0.6295348781,1.5651130614,-0.0002541909
O,1.8472296185,-1.1307407974,0.0001688498
C,4.1645411965,-1.1634664058,-0.0001998355
C,5.500520818,-0.7962627461,-0.000571354
N,4.1249664938,-2.5336815406,0.0000947232
C,6.2703637496,-1.9822037334,-0.000499331
H,5.8851067008,0.2130486844,-0.0009012445
C,5.3860718457,-3.0424418446,-0.0000750343
H,3.2559540164,-3.0440982209,0.0004099525
H,7.3482791123,-2.0557859518,-0.0007356741
H,5.5644609308,-4.1074436256,0.0001138022
Pt,-0.1344564116,-0.3105523932,-0.0003134137
C,-2.4242337924,-2.0608483308,0.0001165655
C,-0.4157609854,-3.2686155197,0.0000454448
C,-2.9762110611,-0.7060704211,0.0000997885
C,-3.146250752,-3.2549299457,0.0002750051
C,-1.0840709189,-4.4792023956,0.0002048594
H,0.66468232,-3.1925870816,-0.0000598405
C,-4.3410521301,-0.4245918994,0.0002755845
C,-2.0055676111,0.3177688425,-0.0000477671
C,-2.4773472627,-4.4674785464,0.0003170952
H,-0.5235652807,-5.4063958423,0.0002357012
C,-4.7686159072,0.8990130906,0.000314013
C,-2.4631240232,1.6316458289,0.0000103702
H,-3.0356996509,-5.3980128744,0.0004411749
C,-3.826523823,1.929784515,0.000190051
H,-1.7418203594,2.4417482895,-0.0000455083
H,-4.141052868,2.9666614235,0.0002389283
N,-1.0655624429,-2.0971576303,-0.0000009585
H,-4.2286602454,-3.2222584031,0.0003708555
H,-5.0967404943,-1.2029908151,0.0003996677
O,-6.1232836361,1.0881797557,0.0004799615
C,-6.6130780925,2.4125224,0.0006823731
H,-6.2913723813,2.9596489497,-0.8937150911
H,-7.7002096753,2.3322077034,0.0008695818
H,-6.2910481377,2.9594931017,0.8950582819

2b-3 (S_0)

(CAM-B3LYP/6-31+G(d,p) for C, H, N, and O and CAM-B3LYP/LanL2DZ for Pt)

-1396.3516932 hartree
C,2.2024664043,3.2708369395,0.0376346006
C,3.3691435532,4.0178291239,0.0738718537
C,3.0008121377,5.382523184,0.082788087
C,1.6207612805,5.4330340624,0.0517315755
N,1.156170736,4.1563234186,0.0250884998
H,0.1956637428,3.8485541271,0.0010630988
H,4.376391654,3.62819928,0.0954487591
H,3.6658354843,6.2335672219,0.1098212472
H,0.9477169002,6.2774267458,0.0469286082
C,1.9194360557,1.8341189458,0.0153196565
C,2.9851919326,0.9291422139,0.0204561681
C,2.9014799629,-0.4754367629,0.0100628935
H,3.9697522336,1.3734318438,0.0742588441
O,0.6630683428,1.5611301476,-0.008634682
O,1.823386601,-1.1531452777,0.0292258988
C,4.1348332288,-1.2698380528,-0.0152219277
C,4.2979454522,-2.6214522172,0.2228806369
N,5.3850910348,-0.7533617267,-0.2785953393
C,5.6741873735,-2.9214899138,0.0979354935
H,3.4983198333,-3.300658702,0.4765264467
C,6.3185889668,-1.74537695,-0.2218380421
H,5.5721243872,0.1957616754,-0.5576049022
H,6.1450108692,-3.8850973634,0.2283563024
H,7.3614563119,-1.5427179387,-0.4159891809
Pt,-0.1308887492,-0.3089478734,0.0075278998
C,-2.3995350568,-2.0828437994,0.0114554888
C,-0.3707783356,-3.2620070463,0.0264574175
C,-2.9681787786,-0.7331014637,-0.0051661111
C,-3.1052721854,-3.285461351,0.0182453518
C,-1.0254478232,-4.4808863799,0.032664229
H,0.7094830315,-3.1704279712,0.0260096962
C,-4.3359793912,-0.4721397821,-0.0145828819
C,-2.0099434723,0.3047302419,-0.0136700206
C,-2.4181341738,-4.4886569105,0.0293214735

H,-0.4516947761,-5.3998845733,0.0390751739
C,-4.786220544,0.8444342086,-0.0331530739
C,-2.4934077377,1.6087617581,-0.0331989479
H,-2.963405548,-5.4269535288,0.0342548613
C,-3.8611813028,1.8891708552,-0.0429245825
H,-1.7894135569,2.434189502,-0.041431353
H,-4.1913329906,2.9210715608,-0.057983166
N,-1.0412971459,-2.1014190209,0.0177396266
H,-4.1880696008,-3.2686693935,0.013862703
H,-5.0791603027,-1.2624797314,-0.0080652847
O,-6.1435681091,1.0105044849,-0.0409813651
C,-6.6560987987,2.3257389383,-0.0606474987
H,-6.3398860515,2.8665405224,-0.9609648408
H,-7.7416851803,2.2268923791,-0.0642302253
H,-6.3479984396,2.8901939995,0.8278952737

2b-4 (S_0)

(CAM-B3LYP/6-31+G(d,p) for C, H, N, and O and CAM-B3LYP/LanL2DZ for Pt)

-1396.3516932 hartree
C,2.1959275497,3.235197807,0.0427702093
C,1.3774598007,4.2986746853,-0.2835524948
C,2.1231193821,5.4880905346,-0.1069411332
C,3.3761132798,5.1216615498,0.3343252977
N,3.4175168111,3.7593573231,0.4083467172
H,4.177371144,3.2296065281,0.80323237
H,0.3591837382,4.2040502735,-0.6291945971
H,1.785497592,6.4991623501,-0.282949265
H,4.2317436512,5.7216888412,0.6067898166
C,1.8956854975,1.7998237625,0.0038044545
C,2.9526190106,0.8756551229,-0.0059433026
C,2.8509288797,-0.5295206679,0.0031909047
H,3.9525984473,1.2890930512,-0.0212048241
O,0.6445335651,1.5372378353,-0.0319053347
O,1.7700342516,-1.1950712875,0.0763586445
C,4.0776611192,-1.3348982502,-0.0635444487
C,4.265810698,-2.6582221155,0.2842187933
N,5.2962731748,-0.8506674474,-0.4882853611
C,5.62642507,-2.9762264489,0.0598662687
H,3.4961575152,-3.3046857427,0.6781608655
C,6.2338422922,-1.8407992103,-0.430744022
H,5.4419978257,0.054279896,-0.905534507
H,6.1106766271,-3.9254494915,0.238049456
H,7.2512565315,-1.6658233862,-0.7479006766
Pt,-0.1746270715,-0.3180936748,0.0173094429
C,-2.4892958886,-2.0303257735,0.0379524425
C,-0.4939063138,-3.2645970789,0.0989710596
C,-3.0204778431,-0.6666305578,-0.0106107533
C,-3.2276033133,-3.2133837022,0.0608019438
C,-1.1816330398,-4.4648796267,0.1225366528
H,0.5885616193,-3.2035665042,0.1078869142
C,-4.3803369651,-0.3648279416,-0.033304313
C,-2.0343852706,0.3430646464,-0.0307742351
C,-2.5740919283,-4.4343400599,0.1037183395
H,-0.6335964042,-5.3989551959,0.15361937
C,-4.7881189967,0.9647030128,-0.0760531884
C,-2.4722545918,1.6630024847,-0.0722551428
H,-3.1451154369,-5.3570735347,0.1211839817
C,-3.8312786643,1.9813034435,-0.0948912483
H,-1.7394574707,2.463212055,-0.0825948369
H,-4.1300999233,3.0224071821,-0.1255421462
N,-1.1316554236,-2.0863935535,0.0597638889
H,-4.309444433,-3.1667730511,0.0437273201
H,-5.147745975,-1.131616097,-0.0177562686
O,-6.1401367026,1.1732681517,-0.0955614983
C,-6.6099113027,2.5037682501,-0.1377140611
H,-6.2707256055,3.0210098331,-1.0434647152
H,-7.6981549501,2.4397399521,-0.1471408091
H,-6.2892072989,3.0712520976,0.7443710492

2b-1 (S_1)

(TD-CAM-B3LYP/6-31+G(d,p) for C, H, N, and O and TD-CAM-B3LYP/LanL2DZ for Pt)

-1396.24220079 hartree
C,2.0743821561,3.3453843911,0.0001851927
C,3.2296376283,4.1119070997,0.0003429486
C,2.8399476377,5.4696326477,0.0003613498
C,1.4587114955,5.4984878757,0.0001128069
N,1.0132649,4.2147784364,0.0000241158
H,0.0567075928,3.896858769,-0.0001458689
H,4.2425463888,3.7370159712,0.0004863289
H,3.4917533843,6.3311801523,0.000504123
H,0.772837565,6.3325688401,0.000013601
C,1.8253807138,1.9050877002,0.0001371193
C,2.9165585792,1.0308704428,0.0001505497
C,2.8728891731,-0.3676835894,0.0000523862
H,3.8969898841,1.4851947795,0.0002107064
O,0.5775909639,1.5914517451,0.0000584504
O,1.8128617613,-1.0947821281,-0.0000636763
C,4.1267505379,-1.1167955232,0.0000699206
C,5.4623012157,-0.7429426257,0.0002059782
N,4.0918863358,-2.4883565158,-0.0000637305
C,6.2353660009,-1.9245529953,0.0001549063
H,5.8401404997,0.2689276717,0.0003365125
C,5.354118789,-2.9893301192,-0.0000289198
H,3.2265262982,-3.0059010745,-0.0001812092
H,7.3134422977,-1.9944356886,0.000234565
H,5.5374460239,-4.0535226485,-0.0001256793
Pt,-0.1653725057,-0.3335963942,-0.0000440443
C,-2.3917309316,-2.1608988677,0.000002145
C,-0.3120546209,-3.3134874181,-0.0000082317
C,-2.9727996368,-0.8549568228,0.0000101912
C,-3.062748634,-3.3938094799,0.0000122895
C,-0.9408123496,-4.5289868116,0.0000027197
H,0.7663486268,-3.2196240902,-0.0000144126
C,-4.3274603438,-0.5515338154,0.0000290661
C,-2.0021269286,0.2311820948,0.000003536
C,-2.3660345239,-4.5783122063,0.0000115304
H,-0.3479632637,-5.4359074976,0.000005193
C,-4.7503676072,0.7855908364,0.0000605001
C,-2.4739281882,1.55702156,0.0000434865
H,-2.8867346243,-5.5287037981,0.0000188094
C,-3.8253476734,1.855504098,0.0000718519
H,-1.7443952378,2.3582574914,0.0000573709
H,-4.1600988441,2.8842900526,0.0001004423
N,-0.9887439305,-2.1395669902,-0.0000121167
H,-4.147627431,-3.3975593949,0.0000217842
H,-5.0932812808,-1.3187383332,0.0000285917
O,-6.0838043793,0.9623279945,0.0000791059
C,-6.6231699785,2.276100771,0.0001571287

H,-6.3188542655,2.8259419524,-0.8958868539
H,-7.7049116467,2.1502849558,0.0001988106
H,-6.3187756236,2.8258674994,0.8962196288

2b-1 (T_1)
(CAM-B3LYP/6-31+G(d,p) for C, H, N, and O and CAM-B3LYP/LanL2DZ for Pt)
-1396.2395237 hartree
C,2.0642201856,3.3507473228,0.0002396715
C,3.2226274921,4.1262419993,0.0003595372
C,2.8288951028,5.483785472,0.0002288055
C,1.4492739596,5.5159060563,0.000552222
N,1.0034159247,4.2241934817,0.0004571592
H,0.0479494424,3.9072905555,0.0005397127
H,4.2364160147,3.753718154,0.0003188596
H,3.4810442691,6.3455262545,0.000119371
H,0.7626142966,6.3490566612,0.0007085339
C,1.8260753996,1.9268827816,0.0001006602
C,2.9092464437,1.0301349649,0.0000722107
C,2.8773345931,-0.3524632166,-0.0000171394
H,3.8928378944,1.4826477948,0.0001375469
O,0.5520200661,1.5875775714,0.0000255323
O,1.7625130123,-1.0867395769,-0.0001165611
C,4.1046382086,-1.1285575851,-0.0000116402
C,5.4507026675,-0.7841466865,0.0001838946
N,4.0457364003,-2.5005962608,-0.000247675
C,6.2006159735,-1.9845125661,0.0000340089
H,5.8498945568,0.219606345,0.000398068
C,5.3045138075,-3.0324487718,-0.0001615359
H,3.1732056449,-3.0018706316,-0.0004536742
H,7.2776918121,-2.0732600289,0.0001119725
H,5.4650502656,-4.1000469635,-0.0002907709
Pt,-0.128362897,-0.3029336922,-0.0001824066
C,-2.3457282622,-2.1477989482,0.0001008445
C,-0.2839207635,-3.284886099,0.000163801
C,-2.9363124586,-0.8169099812,-0.0000051329
C,-3.0236684771,-3.3637435248,0.0002611907
C,-0.9141053452,-4.5118218836,0.0003251547
H,0.7926826022,-3.1720915758,0.0001205177
C,-4.2938201304,-0.5495562419,0.0000375146
C,-1.9809972354,0.2554646659,-0.0001404033
C,-2.3155114667,-4.5550206078,0.0003730602
H,-0.3210561068,-5.4185559873,0.0004129254
C,-4.7409445364,0.7803908386,-0.0000605935
C,-2.4698983371,1.573414727,-0.0002357961
H,-2.8394396982,-5.5044216027,0.0005005866
C,-3.8265587548,1.8502722476,-0.0001986126
H,-1.7499068458,2.3833028533,-0.0003508151
H,-4.1719201483,2.8759432205,-0.0002740338
N,-0.973898902,-2.1315217611,0.0000518393
H,-4.1074163594,-3.369592845,0.0003042272
H,-5.0451148434,-1.3313447392,0.0001492239
O,-6.0787948006,0.9350658369,-0.0000074723
C,-6.6310455908,2.2436552799,-0.0000933975
H,-6.3320406526,2.7968031935,-0.8961815161
H,-7.7115695377,2.1079810626,-0.0000212579
H,-6.331941886,2.7969634368,0.8958627815

2b-4·Cl⁻ (S_0)
(CAM-B3LYP/6-31+G(d,p) for C, H, N, O, and Cl and CAM-B3LYP/LanL2DZ for Pt)
C,-2.7139694102,2.4446942282,-0.1027337317
C,-2.2730836954,3.7590982208,-0.1731277608
C,-3.4132966224,4.5900311227,-0.1765862609
C,-4.5162777776,3.753638735,-0.1081242675
N,-4.0888865796,2.4703539734,-0.0642172586
H,-4.7341328027,1.6648597796,-0.0103507435
H,-1.2360881983,4.0567636374,-0.2161391485
H,-3.4353187978,5.6700783308,-0.2232838733
H,-5.5730204816,3.9771590841,-0.0874393184
C,-1.9103364898,1.2225393162,-0.0723696691
C,-2.5559102829,-0.0149248815,0.0005904966
C,-1.9697794613,-1.2877311384,0.0394535503
H,-3.6350114488,0.015146966,0.0305109876
O,-0.6362592873,1.4344499971,-0.1184808764
O,-0.7092377359,-1.5317265591,0.0136084341
C,-2.8324944452,-2.4685803599,0.1162363838
C,-2.4551717988,-3.8039626125,0.1638709827
N,-4.2064745273,-2.4266140633,0.1543347861
C,-3.6332431332,-4.5775341136,0.2318768248
H,-1.4344505384,-4.1563587513,0.1497307272
C,-4.694818271,-3.6865556129,0.2237019853
H,-4.8133321196,-1.5890524995,0.1352801325
H,-3.7067500017,-5.655210736,0.2812185122
H,-5.7609549208,-3.8567149379,0.2621312778
Pt,0.7585310902,-0.0197209408,-0.0951106585
C,3.5247599128,-0.8258096037,-0.1398540844
C,2.080370439,-2.6724800517,-0.0172262302
C,3.5500966322,0.6373810393,-0.2046181719
C,4.627349335,-1.6812395774,-0.1343070904
C,3.1418636165,-3.5613117074,-0.0090379829
H,1.0410267955,-2.9816398983,0.0263841194
C,4.7240130642,1.3889743636,-0.2714763135
C,2.2738014821,1.2435712436,-0.1942088826
C,4.4372050008,-3.0524500702,-0.0687898576
H,2.95056107,-4.6266541078,0.0431292149
C,4.6471686207,2.7747425823,-0.3298964055
C,2.2339230403,2.6358555686,-0.2540472969
H,5.2925117646,-3.7211167269,-0.0642678703
C,3.3982843663,3.4007694348,-0.3212111265
H,1.2680117964,3.1308075262,-0.2478105465
H,3.3190718477,4.4808233067,-0.3662579879
N,2.2711309073,-1.3478527128,-0.0807306905
Cl,-6.1934438718,0.0666827111,0.1020802574
H,5.6258230436,-1.263836244,-0.1814552154
H,5.7084643821,0.9319174742,-0.2799834896
O,5.8479628571,3.4420397535,-0.3938222055
C,5.8183505524,4.849052092,-0.4555504277
H,5.3430188541,5.2829992707,0.4332648659
H,6.8589865295,5.1735215105,-0.4997941416
H,5.2918308491,5.2035689986,-1.3507353439

2b-4·Cl⁻ (S_1)
(CAM-B3LYP/6-31+G(d,p) for C, H, N, O, and Cl and CAM-B3LYP/LanL2DZ for Pt)
C,2.1855501776,2.8297182688,0.0000828774
C,1.4559086902,4.0130548364,0.0000919586
C,2.3763006653,5.0791671601,0.0001351394
C,3.6428252618,4.5122930001,-0.0000068907
N,3.5200325772,3.1666752253,-0.0000030079

H,4.3343314821,2.5274525296,-0.0000627468
H,0.3777331908,4.0700496815,0.0001292695
H,2.1525636909,6.1368617939,0.000198704
H,4.6212393753,4.970383362,-0.0000649892
C,1.6821741528,1.460584491,0.0000967941
C,2.5962837771,0.3989636674,0.0000471661
C,2.3155466429,-0.970083652,0.0000030412
H,3.641099764,0.6724489714,0.0000393581
O,0.3950746885,1.3703425406,0.0001078937
O,1.1348104523,-1.4979143001,0.000012409
C,3.4146974962,-1.9281832706,-0.000039408
C,3.342582282,-3.3162895974,-0.0000704485
N,4.7468611379,-1.5829484331,0.0000180293
C,4.6621686722,-3.8096821784,-0.0002257146
H,2.4244203775,-3.8844384804,-0.0000814244
C,5.5010085694,-2.7048153018,-0.0000458507
H,5.1519470658,-0.6310461869,0.0001152253
H,4.9732793589,-4.8450287032,-0.0003554573
H,6.5792525654,-2.6372701598,-0.0000169511
Pt,-0.6332669332,-0.3973079103,0.000089917
C,-3.1436384582,-1.8075204431,0.0000724589
C,-1.2958643279,-3.310006016,0.0000618384
C,-3.4869817949,-0.4168343076,0.0000800059
C,-4.0208281639,-2.9033471408,0.0000644174
C,-2.1303049685,-4.3968956207,0.0000545229
H,-0.2159094066,-3.4025580365,0.000062124
C,-4.77175945,0.1182761634,0.0000812007
C,-2.3477464659,0.483743859,0.0000884449
C,-3.542811644,-4.1930357412,0.0000559544
H,-1.7052545315,-5.3937346305,0.000048
C,-4.9539659867,1.5066337707,0.0000861865
C,-2.5769949968,1.8677896208,0.0000930668
H,-4.2243440874,-5.03651584,0.0000497468
C,-3.8621998619,2.3964449964,0.0000912433
H,-1.7154777223,2.5272315045,0.000098091
H,-4.0095264306,3.4684689005,0.00009273
N,-1.7592754725,-2.0383484159,0.0000720402
Cl,6.0985961225,1.3049094137,-0.0003325569
H,-5.0899399908,-2.7161715219,0.0000648249
H,-5.6589848819,-0.5054288306,0.0000779869
O,-6.2478808391,1.9162277561,0.0000833122
C,-6.5304767079,3.3024928565,0.0001135401
H,-6.12788518,3.7906499561,-0.8941652245
H,-7.617435006,3.3853990639,0.0001281565
H,-6.1278629284,3.790615329,0.8944009955

2b-4·Cl⁻ (T_1)

(CAM-B3LYP/6-31+G(d,p) for C, H, N, O, and Cl and CAM-B3LYP/LanL2DZ for Pt)

C,2.2083818434,2.8115469399,-0.0000992822
C,1.4640769136,3.9988208943,0.0000014533
C,2.3714357414,5.0662927731,-0.0000376875
C,3.6487441314,4.5126821691,-0.0000954113
N,3.5450396673,3.1673182849,-0.0001510881
H,4.3689501738,2.5408269847,-0.0002304839
H,0.3853576411,4.041946666,0.0000684849
H,2.1387756,6.1218158498,-0.0000044429
H,4.6201055462,4.9855467998,-0.0001296687
C,1.7061281288,1.4652859146,-0.0001114679
C,2.6585577916,0.374022872,-0.0001827101
C,2.3847027178,-1.038621421,-0.0001508885
H,3.7059193408,0.6584397115,-0.0002622907
O,0.4383759385,1.3108213926,-0.0000155425
O,1.2121081138,-1.5275384827,-0.0000513367
C,3.5014113035,-1.9577380693,-0.0001889468
C,3.447630689,-3.3530090623,-0.0001481124
N,4.8348051851,-1.5972727615,-0.0002955266
C,4.7683423141,-3.8270766852,-0.0001347632
H,2.5349211103,-3.9299242882,-0.000084358
C,5.5968240541,-2.7099873313,-0.0002882905
H,5.2343508633,-0.6415717914,-0.0003943593
H,5.0932485657,-4.8579008361,-0.0000745725
H,6.6745346509,-2.6336813,-0.0003677934
Pt,-0.6475922722,-0.4078463634,0.0000435269
C,-3.1800438082,-1.782120576,0.0001924981
C,-1.3458925294,-3.2881462751,0.0001379929
C,-3.5187207776,-0.3749537073,0.0001752037
C,-4.0568737647,-2.8658205101,0.0002554732
C,-2.1823014648,-4.3767168415,0.0001989858
H,-0.2663658606,-3.3853957114,0.0000895459
C,-4.8192260715,0.141977789,0.0002241717
C,-2.3991730743,0.5000094877,0.0001000981
C,-3.5844337621,-4.1680424259,0.0002603637
H,-1.758453866,-5.3746203129,0.0001992451
C,-5.0205715182,1.5180631531,0.0001985303
C,-2.6333817308,1.867026896,0.0000776635
H,-4.2725668647,-5.0066526097,0.000308589
C,-3.93318535,2.3925254243,0.000125238
H,-1.7874095492,2.5471514514,0.00002129
H,-4.0739134234,3.4666765851,0.0001024807
N,-1.8072606671,-2.0149019683,0.0001346726
Cl,6.0777863438,1.29247081,0.0003458207
H,-5.1245471033,-2.6721184214,0.0002992202
H,-5.6931420312,-0.5016867969,0.0002822076
O,-6.3363544794,1.9275646215,0.0002435255
C,-6.5958699057,3.3098611657,0.0002964403
H,-6.1858087253,3.7996497784,-0.89253309
H,-7.6820715359,3.416870592,0.0003745041
H,-6.1856822338,3.7996035425,0.8930928879

2c-1 (S_0)

(CAM-B3LYP/6-31+G(d,p) for C, H, N, O, and F and CAM-B3LYP/LanL2DZ for Pt)

-1480.3237915 hartree
C,3.0576525753,-0.0001891392,-2.6180024583
C,4.4455075715,-0.0001042242,-2.8662300547
C,4.6295937051,-0.0000056591,-4.2659693014
C,3.3568503541,-0.0000217705,-4.8680288041
N,2.4063390669,-0.0001304952,-3.8659146298
H,1.4046464882,-0.0001725003,-3.9694289807
H,5.2195980896,-0.0001267247,-2.1230464126
H,5.5689565119,0.0000680326,-4.7826419376
H,3.107620976,0.0000358704,-5.9155478401
C,2.3076249403,-0.0003220352,-1.3625156749
C,2.969252368,-0.0003758985,-0.1337118613
C,2.448751551,-0.0003748576,1.165783612
H,4.0655826544,-0.0003900608,-0.1967766393
O,1.0293312977,-0.0002079719,-1.5494246038
O,1.204332674,-0.0002610562,1.4769610881
C,3.3509532766,-0.0002278487,2.3202397469

C,4.7577258838,-0.0001951583,2.3914594293
N,2.8633995148,-0.000079278,3.6401732066
C,5.118718804,-0.0000386799,3.7578132578
H,5.43255603,-0.000279668,1.5567124603
C,3.9343143891,0.0000376845,4.5158595201
H,1.8863444611,-0.0000604637,3.8709565648
H,6.1168783147,0.0000178657,4.1494149626
H,3.8190432349,0.0001613848,5.5857952735
Pt,-0.432648612,-0.0001261405,0.0049179926
C,-3.1424027239,0.0000215081,0.8347519727
C,-1.539163145,-0.0002847336,2.6308450817
C,-3.2141926801,0.000175051,-0.6184286115
C,-4.1865578302,0.0000257614,1.7725996356
C,-2.5684788184,-0.0002847943,3.5742295252
H,-0.4708116049,-0.000401034,2.9082031755
C,-4.3405353171,0.0003525064,-1.4566571624
C,-1.9409272827,0.0001343416,-1.2537664631
C,-3.8991437063,-0.0001284567,3.1386492
H,-2.3307613716,-0.0004048422,4.6373058037
C,-4.2512957181,0.0004847559,-2.8483817587
C,-1.7935416426,0.0002518084,-2.6393319677
H,-4.7131088069,-0.0001192438,3.8666200009
C,-2.9605257519,0.0004272683,-3.401364143
H,-5.1392573127,0.0006258337,-3.4787845074
H,-0.7959525891,0.0002021196,-3.0783883007
N,-1.8198477647,-0.0001394498,1.2906601357
H,-5.224897574,0.0001584035,1.4388142728
F,-5.560314424,0.0004010314,-0.9252170405
F,-2.856433007,0.0005475775,-4.7284632048

2c-2 (S_0)
(CAM-B3LYP/6-31+G(d,p) for C, H, N, O, and F and CAM-B3LYP/LanL2DZ for Pt)
-1480.319755 hartree
C,3.0775838335,-0.0001819568,-2.594351179
C,4.4666379727,-0.0002127019,-2.8330830585
C,4.6604555399,-0.000145007,-4.2318653248
C,3.3919694364,-0.0000696604,-4.8422884108
N,2.4350579613,-0.0000895895,-3.8459847021
H,1.4335473643,-0.0000519505,-3.9557270361
H,5.2361662643,-0.0002877307,-2.0869481683
H,5.6030890181,-0.0001523574,-4.7423545002
H,3.1492740336,-0.0000072797,-5.8913680189
C,2.3129441761,-0.0002343787,-1.3448853378
C,2.9646668557,-0.0003184218,-0.1105774981
C,2.4186336322,-0.0003346794,1.1863493287
H,4.0553343095,-0.0003698441,-0.1755275522
O,1.039107143,-0.0001222525,-1.5509803722
O,1.1740649555,-0.0002374841,1.4597690797
C,3.2854152283,-0.0003196155,2.369250546
C,2.9039887266,-0.0003293002,3.7257598748
N,4.6927479806,-0.0002819563,2.3312284563
C,4.0811674888,-0.0003065026,4.5078871162
H,1.8912895421,-0.000352062,4.0830414423
C,5.1771243228,-0.0002756815,3.6291428337
H,5.2400141519,-0.0002607758,1.4900494195
H,4.1318800595,-0.0003072763,5.5788263081
H,6.2282482569,-0.0002492363,3.8611518517
Pt,-0.4512511967,-0.0000394863,-0.018023384
C,-3.1573773814,0.0000763169,0.8231730814
C,-1.5433148819,-0.0001902204,2.612068362
C,-3.2342597898,0.0002136484,-0.6307175982
C,-4.1967040516,0.000078722,1.765521372
C,-2.5692717805,-0.000189446,3.5596020311
H,-0.4705044641,-0.0002884525,2.8871381896
C,-4.3633594835,0.0003636963,-1.4643447324
C,-1.9635154154,0.0001885658,-1.2713731777
C,-3.9016144893,-0.000055157,3.1305856221
H,-2.3244456313,-0.0002893361,4.621641057
C,-4.2793998385,0.0004874181,-2.8569433737
C,-1.820908833,0.0002997713,-2.6573415443
H,-4.7119698657,-0.0000427943,3.8625062391
C,-2.9911996928,0.0004490115,-3.414756766
H,-5.1699439125,0.0006068505,-3.4835011879
H,-0.8255539257,0.0002682887,-3.1003043816
N,-1.8334095402,-0.0000635201,1.2738669348
H,-5.236621543,0.0001981087,1.4375884715
F,-5.5814024462,0.0003914637,-0.9289076502
F,-2.8920520606,0.0005627019,-4.7424195528

2c-3 (S_0)
(CAM-B3LYP/6-31+G(d,p) for C, H, N, O, and F and CAM-B3LYP/LanL2DZ for Pt)
-1480.3179382 hartree
C,2.9978413548,-0.0000683128,-2.6615214009
C,2.4622942405,-0.0008126728,-3.9656116652
C,3.5415731795,-0.000151879,-4.8775146014
C,4.7317405925,0.0010330994,-4.1310444914
N,4.4014234523,0.001069095,-2.7858726184
H,5.0418282443,0.0018791123,-2.0138971055
H,1.412899736,-0.0017388292,-4.2026312287
H,3.4675621158,-0.0004907113,-5.9473907962
H,5.748898192,0.0018145884,-4.4832525097
C,2.2737270798,-0.0003552836,-1.3888516049
C,2.9568988155,-0.0005785966,-0.1632231042
C,2.4460375531,-0.000519796,1.1396672264
H,4.048105626,-0.0008762992,-0.2214228912
O,1.0003710733,-0.000185865,-1.5457661208
O,1.2073467836,-0.0001636908,1.4668139089
C,3.3599795945,-0.0004800054,2.2880571352
C,4.766584237,-0.0010334662,2.3526582528
N,2.8788496279,0.0001712809,3.6095158573
C,5.1342724213,-0.0007164977,3.7176263775
H,5.4382398602,-0.0016224668,1.5170098238
C,3.9535705789,0.0000434495,4.4809439161
H,1.9022696035,0.0006590089,3.8443922353
H,6.1340602604,-0.0010020483,4.1047468967
H,3.8426889988,0.0004703706,5.551347861
Pt,-0.4536421829,-0.0001253077,0.0161042489
C,-3.1614915592,0.0000968113,0.8506956355
C,-1.5530808465,-0.0002325289,2.6431451803
C,-3.2364926948,0.0002359282,-0.6016260597
C,-4.2029520205,0.0001261375,1.7919626862
C,-2.5797120434,-0.0002094277,3.5892532723
H,-0.4842197291,-0.000378396,2.9167585005
C,-4.3640384096,0.0004352382,-1.4390936512
C,-1.9652595956,0.0001602276,-1.2404857455
C,-3.9116932252,-0.0000276192,3.156952785
H,-2.3394872386,-0.000336426,4.6515819127
C,-4.2758431467,0.0005580759,-2.8300365596

C,-1.8171828534,0.0002786923,-2.6250736816
H,-4.7235465588,0.0000005623,3.8871745495
C,-2.9848847715,0.0004744883,-3.3857699136
H,-5.1639729021,0.0007123924,-3.4600441399
H,-0.8165161889,0.000226085,-3.0672137684
N,-1.8377036668,-0.000082706,1.3037374436
H,-5.2420671077,0.0002764912,1.4605315118
F,-5.5831408448,0.0005112157,-0.9053145215
F,-2.8860259752,0.0005889714,-4.7126014677

2c-4 (S_0)
(CAM-B3LYP/6-31+G(d,p) for C, H, N, O, and F and CAM-B3LYP/LanL2DZ for Pt)
-1480.3129742 hartree
C,3.0197670208,0.1021993104,-2.6290913607
C,2.6038362909,-0.2943312451,-3.9135340623
C,3.6667865552,-0.0350486053,-4.8108094332
C,4.7193157958,0.5307709592,-4.0752325169
N,4.3300131316,0.6056530615,-2.7455693489
H,4.8560148776,1.0077256394,-1.9928146
H,1.6448935707,-0.7192398557,-4.1519051385
H,3.6666857917,-0.2324942316,-5.8649167639
H,5.6807972363,0.8681198895,-4.4211758999
C,2.2691992843,0.03474704,-1.3693331158
C,2.939715373,0.01275381,-0.1375470116
C,2.4013817843,0.0106846995,1.1606306782
H,4.0278093159,0.0018699471,-0.1938200577
O,1.0028438257,-0.0209502222,-1.551321003
O,1.1672437102,0.0970630866,1.4537920457
C,3.2899820895,-0.0739974583,2.3303238642
C,3.0761988329,0.4213178221,3.6282595356
N,4.5497188926,-0.7004288306,2.3176758496
C,4.2136250512,0.0966885785,4.4068471281
H,2.203587631,0.955145333,3.9551058074
C,5.1074796371,-0.6060565511,3.5857068154
H,4.937344337,-1.1877035811,1.5315480961
H,4.3642038457,0.3430656482,5.4393694744
H,6.0677014584,-1.0239307316,3.8325887787
Pt,-0.4778832738,0.0201582627,-0.0079471607
C,-3.1811703969,0.0185331824,0.8388341984
C,-1.5626843484,0.0999784036,2.6233467489
C,-3.2610008998,-0.0368394218,-0.613373072
C,-4.2181063095,0.0401470621,1.7839216243
C,-2.5865212228,0.1213336852,3.5727396163
H,-0.4892480975,0.1191378059,2.895399012
C,-4.3913469943,-0.0812212041,-1.4450161637
C,-1.9928748039,-0.0458794882,-1.2585098383
C,-3.9198060963,0.0917998658,3.1471021507
H,-2.3394629427,0.15986614,4.6334901616
C,-4.3086380057,-0.131717555,-2.835929408
C,-1.849390692,-0.0933788199,-2.6424657365
H,-4.7283948263,0.1085227328,3.8806978222
C,-3.0206104539,-0.1356674751,-3.39719969
H,-5.1995122231,-0.1658065754,-3.4609789067
H,-0.8509353738,-0.0927623911,-3.0893717713
N,-1.8564697384,0.0513285415,1.2867938601
H,-5.2585982959,0.0164342393,1.45863197
F,-5.6085558064,-0.0760564202,-0.9069764438
F,-2.9272738979,-0.1830497628,-4.7237196145

2c-1 (S_1)
(TD-CAM-B3LYP/6-31+G(d,p) for C, H, N, O, and F and TD-CAM-B3LYP/LanL2DZ for Pt)
-1480.19529986 hartree
C,-2.5969295763,2.9914645487,-0.0000914803
C,-3.8756276807,3.5313386103,-0.0001114685
C,-3.7449607686,4.9359484809,-0.0000948024
C,-2.3919989894,5.2205515478,-0.000106222
N,-1.7154702511,4.0439410598,-0.0001050271
H,-0.7154693771,3.9168608453,-0.0001098933
H,-4.8014962245,2.975241599,-0.0001168223
H,-4.5449898934,5.6617771164,-0.000089545
H,-1.8729138778,6.1674717356,-0.0001093934
C,-2.0932686104,1.6237446531,-0.0000789298
C,-3.0063321535,0.5643736901,-0.0000776948
C,-2.7116340222,-0.8023973026,-0.0000623182
H,-4.0528026285,0.8331257882,-0.0000890432
O,-0.8063427284,1.5392433727,-0.0000609842
O,-1.5341165858,-1.3268844581,-0.0000416298
C,-3.8058945837,-1.7656876362,-0.0000555487
C,-5.1876223706,-1.6370011979,-0.0000607319
N,-3.5264763984,-3.1096462969,-0.0000494438
C,-5.7365632918,-2.9367828274,-0.0000331817
H,-5.7406438428,-0.7092483463,-0.0000686133
C,-4.6787952958,-3.8271159526,-0.0000427323
H,-2.5839096887,-3.4672883605,-0.0000531116
H,-6.7846615334,-3.1983927353,-0.000020007
H,-4.6695693946,-4.9069536198,-0.0000367694
Pt,0.2607317291,-0.2183505664,-0.0000298025
C,2.789613595,-1.6048363836,-0.0000311169
C,0.9372219111,-3.1115998641,-0.000107108
C,3.1087353939,-0.2219598193,0.0000240534
C,3.6678459199,-2.7053019552,-0.0000544648
C,1.7749752298,-4.19338714,-0.0001297449
H,-0.1400691496,-3.2121843685,-0.0001250058
C,4.3825603619,0.3736898901,0.0000685887
C,1.9648490498,0.6824215216,0.0000336726
C,3.1848853251,-3.9923737498,-0.0001046881
H,1.3520772583,-5.1912092559,-0.0001662477
C,4.5734215642,1.7327820254,0.0001197288
C,2.1629812807,2.0741809021,0.0000848804
H,3.8639305068,-4.8365510511,-0.0001231666
C,3.44054697,2.5674707565,0.0001268569
H,5.5724992396,2.1514381055,0.0001529927
H,1.3135455863,2.7435751844,0.0000913182
N,1.3935836859,-1.833415921,-0.0000632413
H,4.732561043,-2.5150610811,-0.0000329674
F,5.4847229348,-0.4129508444,0.0000602796
F,3.6529993319,3.9027003002,0.0001765765

2c-1 (T_1)
(CAM-B3LYP/6-31+G(d,p) for C, H, N, O, and F and CAM-B3LYP/LanL2DZ for Pt)
-1480.2275517 hartree
C,-2.6071169009,2.9534829445,-0.0001315708
C,-3.916430302,3.4737603381,-0.0001378771
C,-3.8152634318,4.8768871502,-0.0001734592
C,-2.469313185,5.1979502283,-0.0002027461
N,-1.7576926098,4.0312913395,-0.0001766395
H,-0.7551352131,3.9239574483,-0.0001866483

H,-4.8314947376,2.9001327664,-0.0001123401
H,-4.6323066024,5.583744387,-0.0001822153
H,-1.9754309957,6.1579775891,-0.0002372631
C,-2.0737760281,1.6298876716,-0.0000962919
C,-2.9892477972,0.5376750469,-0.0000544615
C,-2.7206044873,-0.8621500523,-0.0000177619
H,-4.0371880067,0.8130542194,-0.000050901
O,-0.7665707144,1.5230476628,-0.0001019941
O,-1.5205855781,-1.3714293066,-0.0000122326
C,-3.8346902484,-1.763119361,0.0000111053
C,-5.2273680045,-1.5833695836,0.0000186691
N,-3.6145169264,-3.1179374782,0.0000443293
C,-5.8220084883,-2.861021343,0.000046985
H,-5.7495973755,-0.6379614492,0.0000029607
C,-4.8028717198,-3.7942649591,0.0000604179
H,-2.6864886209,-3.5097689358,0.0000471467
H,-6.8797202185,-3.0813803251,0.0000574165
H,-4.8370218634,-4.8733490878,0.0000829097
Pt,0.2821985525,-0.2187803832,-0.0000614577
C,2.8146367891,-1.6023343039,-0.0000754032
C,0.9984736673,-3.0902213231,-0.0002382737
C,3.1422374131,-0.1778574142,0.0000423087
C,3.7026356467,-2.6801065094,-0.0001241108
C,1.8357473064,-4.1914448223,-0.0002894255
H,-0.0821244136,-3.1662299667,-0.0002770043
C,4.4206808332,0.3756217043,0.0001257163
C,2.0240917517,0.6938852749,0.0000722948
C,3.2104414983,-3.9763131065,-0.000232451
H,1.4155192877,-5.1900786775,-0.0003726179
C,4.6517255037,1.7369523608,0.0002337104
C,2.2364929641,2.0709120417,0.0001774638
H,3.8990403927,-4.8150272039,-0.0002702162
C,3.5313896884,2.5543719258,0.0002555679
H,5.6560862849,2.1398345553,0.0002963135
H,1.4003762764,2.7579441269,0.0001986546
N,1.473678802,-1.8383681668,-0.0001374858
H,4.7659844649,-2.4902620331,-0.0000753129
F,5.5095528899,-0.4345343403,0.0001010015
F,3.7293034563,3.8917003509,0.0003591899

2c-4·Cl⁻ (S$_0$)
(CAM-B3LYP/6-31+G(d,p) for C, H, N, O, F, and Cl and CAM-B3LYP/LanL2DZ for Pt)
C,-2.4479419767,2.6603352718,0.0260088151
C,-1.8462666249,3.9119764293,0.0251808035
C,-2.8742770142,4.8770190403,0.0290429322
C,-4.0737285167,4.1821599209,0.0319915527
N,-3.8101628657,2.8553694128,0.0300963053
H,-4.5511164573,2.1348011915,0.0316294407
H,-0.7801741976,4.0826857799,0.0221580123
H,-2.7609355904,5.9522002539,0.0295716609
H,-5.0948196994,4.5349615783,0.0352615674
C,-1.8047613773,1.3487564167,0.0230819744
C,-2.5981830402,0.1980724685,0.0244572839
C,-2.1717219043,-1.1359155019,0.0223256774
H,-3.6662055614,0.3582142336,0.0275478974
O,-0.512060215,1.3992681728,0.019569647
O,-0.9469746251,-1.530528487,0.0185392157
C,-3.1695432994,-2.2054751712,0.0243305049
C,-2.9573031195,-3.5779496494,0.0225378747
N,-4.5291997522,-1.998156703,0.0283985768
C,-4.2210811887,-4.2042821938,0.0257775433
H,-1.9867980316,-4.0515370267,0.0193238531
C,-5.1669665424,-3.1909306436,0.0293463617
H,-5.0303030714,-1.0925417721,0.0306455044
H,-4.4253429365,-5.2660412385,0.0255455698
H,-6.246495357,-3.2319007767,0.0324860859
Pt,0.688901548,-0.2177979381,0.0145336543
C,3.3366243014,-1.3699704081,0.005397398
C,1.6559182074,-3.0133785942,0.0092423064
C,3.5359694757,0.0789423852,0.0059572976
C,4.3165978398,-2.3665254563,0.0015322379
C,2.5886560815,-4.0360395171,0.0055129319
H,0.5839210481,-3.1815747343,0.012423031
C,4.7605071266,0.743254419,0.0027044549
C,2.3430023389,0.8500420358,0.0102695936
C,3.9393470454,-3.7008416483,0.0016004823
H,2.2560036984,-5.0673196234,0.0056933933
C,4.8709767534,2.1182777048,0.0034775928
C,2.433887743,2.2443005993,0.0111288758
H,4.6991912131,-4.4761893177,-0.0013976594
C,3.680599602,2.8352649311,0.0077499479
H,5.8354769919,2.6089101717,0.000887875
H,1.5355799202,2.8506295983,0.0144052667
N,2.0226937277,-1.7253025082,0.0091708594
Cl,-6.1876782339,0.7174840059,0.0354258392
H,5.3593195426,-2.0840412449,-0.0014747475
F,5.92316439,0.03214631,-0.0014793575
F,3.7728226037,4.1876688232,0.008567066

2c-4·Cl⁻ (S$_1$)
(CAM-B3LYP/6-31+G(d,p) for C, H, N, O, F, and Cl and CAM-B3LYP/LanL2DZ for Pt)
C,-2.4632462452,2.6071841554,-0.000208519
C,-1.8894836274,3.8773095281,-0.0005028357
C,-2.9360351886,4.8140388589,-0.0000045813
C,-4.1219595706,4.0883051531,0.000083037
N,-3.8309412442,2.7713775344,-0.000015513
H,-4.5584466754,2.0328995961,0.0001473131
H,-0.8277808294,4.0727757255,-0.0007859113
H,-2.8495829108,5.8913856167,0.0001197086
H,-5.1503479287,4.4195143122,0.0003232134
C,-1.7908254471,1.3196860053,-0.000237532
C,-2.560558641,0.1472016234,-0.0001269776
C,-2.106906101,-1.1724175116,-0.0000099485
H,-3.6320909965,0.2831687172,-0.0001249219
O,-0.5004756453,1.3947394587,-0.0003121366
O,-0.8630070245,-1.5420558195,-0.0001010435
C,-3.0673035402,-2.2658990023,-0.0000348195
C,-2.8138321869,-3.6335213392,-0.000136527
N,-4.4333649706,-2.0978527347,-0.0003685992
C,-4.0566217283,-4.2946079216,0.000322336
H,-1.8296299359,-4.0773237857,-0.0001434541
C,-5.0332594149,-3.3086303745,-0.0001359783
H,-4.9594233487,-1.2065680115,-0.0006687937
H,-4.229682708,-5.3615905923,0.0006520374
H,-6.1109989137,-3.3836189977,-0.0002128078
Pt,0.7355566992,-0.2300040123,-0.0003385836
C,3.4068984759,-1.3194595583,-0.0000847808
C,1.7391918345,-3.0324879704,-0.0002627065

C,3.5654253156,0.0985040179,-0.0000411521
C,4.4042969094,-2.3114922899,-0.0000054392
C,2.693916277,-4.0119469414,-0.0001892229
H,0.6782292372,-3.2507166404,-0.0003676175
C,4.7573344773,0.8362810603,0.0001012738
C,2.3356522215,0.866412905,-0.0001418969
C,4.0726884267,-3.6468364867,-0.0000573669
H,2.3868583291,-5.0513836028,-0.0002333035
C,4.7949016098,2.2125984655,0.0001382939
C,2.3655913099,2.2698941692,-0.000112843
H,4.8464914994,-4.4061731004,0.0000034835
C,3.5814048452,2.9102313652,0.0000275839
H,5.7422492837,2.7379920985,0.0002501544
H,1.4377626589,2.8295581353,-0.0002001981
N,2.0519210996,-1.7114918794,-0.0002174701
Cl,-6.13012602,0.5950287949,0.0003712642
H,5.4409155329,-2.0012549513,0.0000988721
F,5.9534792341,0.1857412595,0.0002148127
F,3.640269566,4.2683059672,0.0000650973

2c-4·Cl⁻ (T_1)
(CAM-B3LYP/6-31+G(d,p) for C, H, N, O, F, and Cl and CAM-B3LYP/LanL2DZ for Pt)
C,-2.4854994001,2.5986440072,-0.0000187655
C,-1.9098674231,3.8817277626,0.0001338145
C,-2.9554235163,4.8177572779,-0.0001096077
C,-4.1452733268,4.0992909238,-0.0000786957
N,-3.8576804165,2.7747707029,-0.0000661604
H,-4.5867657524,2.0438413949,-0.0001129452
H,-0.847597607,4.0720883801,0.0002608226
H,-2.8668506334,5.8953697794,-0.0001807707
H,-5.1720899816,4.4346602327,-0.0001465242
C,-1.8061067716,1.339184502,0.00000137
C,-2.575451967,0.131844069,-0.0000594571
C,-2.1313670454,-1.2287896984,-0.0000384145
H,-3.6530748791,0.2704213526,-0.0001165232
O,-0.4901757409,1.3906907777,0.0001134031
O,-0.8677573777,-1.5653783766,0.0000596043
C,-3.0982347736,-2.2913190246,-0.0000645205
C,-2.8529758073,-3.6716818311,0.0000232931
N,-4.4705120363,-2.1273109829,-0.0000217592
C,-4.0977512467,-4.3272084357,-0.000197612
H,-1.8700006167,-4.1178063255,0.000095353
C,-5.0745000503,-3.3415068737,-0.0000658214
H,-4.9976712148,-1.2394962673,0.0000319771
H,-4.2745455925,-5.394024518,-0.0003030183
H,-6.1521447778,-3.4151397327,-0.0000671289
Pt,0.7405796979,-0.2070669407,-0.000151066
C,3.4073394495,-1.3089014613,-0.0001238758
C,1.7537726111,-2.981143185,-0.0000855382
C,3.5794257869,0.1442736282,-0.0001383234
C,4.4041110405,-2.287443197,-0.0001171191
C,2.7054226378,-3.9872578956,-0.0000798445
H,0.6839706123,-3.1648475417,-0.0000753603
C,4.7896889844,0.8329623278,-0.0001412986
C,2.3710282732,0.8917391611,-0.0001371468
C,4.0497260914,-3.628971952,-0.0000962378
H,2.3910784359,-5.0243438368,-0.0000635515
C,4.8721357254,2.2105221859,-0.0001413451
C,2.4328410935,2.2876254786,-0.0001346631
H,4.8229433779,-4.3910410569,-0.0000914832
C,3.6676426528,2.9033629645,-0.0001364039
H,5.8265804152,2.7204869453,-0.0001431953
H,1.5202572925,2.8724216845,-0.0001322169
N,2.0991108585,-1.6870457016,-0.000107394
Cl,-6.1354703108,0.5699135032,-0.0000394246
H,5.4418401354,-1.9867995339,-0.0001276754
F,5.9671279974,0.1456419476,-0.0001390104
F,3.733269096,4.2580843797,-0.0001347395

2d-1 (S_0)
(CAM-B3LYP/6-31+G(d,p) for C, H, N, and O and CAM-B3LYP/LanL2DZ for Pt)
-1358.0653354 hartree
C,-3.1941673713,2.5273608298,0.0001239172
C,-4.5520478151,2.8046545264,0.0001192523
C,-4.698644661,4.2102267516,0.0001941657
C,-3.4285163291,4.7538958635,0.0002804867
N,-2.5351539297,3.7300380791,0.0002350406
H,-1.5281786903,3.7894843928,0.0002684327
H,-5.3508131205,2.0775843627,0.0000510188
H,-5.625555491,4.7652340395,0.0001998061
H,-3.1043253118,5.7839290064,0.0003638237
C,-2.4184101843,1.2877189286,0.0000496347
C,-3.0894989253,0.0630427347,-0.0000598153
C,-2.5159347589,-1.2158378459,-0.0001556846
H,-4.1692962218,0.1090047409,-0.0000737991
O,-1.1446248864,1.4803956358,0.0000750626
O,-1.2632869918,-1.485536756,-0.0001652717
C,-3.4007790841,-2.3829566422,-0.0002775245
C,-4.777799059,-2.5374307926,-0.0003240521
N,-2.8543251533,-3.6409441407,-0.0003396399
C,-5.0518099523,-3.9241695449,-0.0004872936
H,-5.5076989578,-1.7413692354,-0.0002796157
C,-3.8370419949,-4.5808955227,-0.000471502
H,-1.8582937095,-3.7933417851,-0.0002982082
H,-6.0253838995,-4.3925430821,-0.0005822236
H,-3.6077182588,-5.6361322015,-0.0005484352
Pt,0.2507915109,0.0127908836,-0.000023855
C,3.0018069952,-0.7765756563,0.0000292381
C,1.6446431678,-2.6610093871,0.000339651
C,3.0260842716,0.6437747131,-0.0001920675
C,4.1765913085,-1.5445215184,0.0001139232
C,2.7668598788,-3.4967312161,0.0004346049
H,0.6310879161,-3.0432808714,0.0004177128
C,4.260534579,1.3168558867,-0.0003227425
C,1.7669784695,1.2911957198,-0.0002643948
C,5.428988998,-0.8351419135,-0.0000250457
C,4.0325815779,-2.9417978488,0.000324671
H,2.6259766558,-4.5711960093,0.0005938404
C,4.2324538736,2.724304732,-0.000534571
C,5.4639744018,0.5242790353,-0.0002315457
C,1.7910503873,2.6756533248,-0.0004661643
H,6.3502666072,-1.409743756,0.0000383405
H,4.9137705191,-3.576880998,0.0003962349
C,3.013005816,3.3775125505,-0.0006009344
H,5.1615992754,3.2862833735,-0.0006413736
H,6.4209583511,1.0386234052,-0.0003328934
H,0.8598447097,3.2322153243,-0.0005238394
H,2.9956680468,4.463900277,-0.00076154

N,1.7604214404,-1.3386473936,0.0001481748

2d-2 (S_0)

(CAM-B3LYP/6-31+G(d,p) for C, H, N, and O and CAM-B3LYP/LanL2DZ for Pt)

-1358.0610329 hartree

C,-3.1894712364,2.4929036684,-0.0001074802
C,-4.5493083707,2.7602851908,-0.0000852125
C,-4.7069951359,4.1648633673,-0.0001750694
C,-3.4410078614,4.7177244561,-0.0001623464
N,-2.5404061153,3.7003911667,-0.000115433
H,-1.5335245974,3.7660308227,-0.0001011349
H,-5.3443306048,2.0290454042,-0.0000493014
H,-5.6380660086,4.7128611402,-0.0002113937
H,-3.1243682519,5.7500647734,-0.000189758
C,-2.3983229135,1.2609387279,-0.0000852881
C,-3.055630626,0.02701374,-0.0000524945
C,-2.4657487757,-1.250630012,-0.0000283077
H,-4.1353471773,0.085262807,0.0000066327
O,-1.1291348413,1.4713696188,-0.0001281656
O,-1.2132170642,-1.486448414,0.0000018438
C,-3.3169231566,-2.4451908535,-0.000024801
C,-2.9324287524,-3.7733794887,0.0002837809
N,-4.6954082219,-2.4271249606,-0.0003269713
C,-4.1025231603,-4.565944135,0.0001601052
H,-1.9088467436,-4.1150607226,0.0005846961
C,-5.1770630079,-3.701881239,-0.0002427151
H,-5.2687671852,-1.600114674,-0.0006607634
H,-4.1585918765,-5.644807102,0.0003366493
H,-6.2392282995,-3.8972343469,-0.0004699653
Pt,0.2817212483,0.0154795944,-0.0000885192
C,3.0244167473,-0.791016575,-0.0001076515
C,1.6465368113,-2.6640725261,-0.0001787122
C,3.0594823185,0.6295443584,-0.0000557663
C,4.1916823192,-1.5689285413,-0.0001322053
C,2.7631500934,-3.5085399984,-0.0002059665
H,0.6280851301,-3.0358590641,-0.0001994742
C,4.2993267232,1.292117547,-0.0000306389
C,1.8049370169,1.2872980427,-0.0000362579
C,5.4498806298,-0.8698334413,-0.000104023
C,4.0336990192,-2.9653058191,-0.0001831557
H,2.6122807063,-4.5816173033,-0.0002458561
C,4.2827036354,2.7001286411,0.000016332
C,5.4961597165,0.4894064683,-0.0000559661
C,1.840917466,2.6714835847,0.000006468
H,6.3665810915,-1.4517966579,-0.0001220972
H,4.9089176359,-3.6086905108,-0.0002035933
C,3.0689010738,3.363406496,0.000033405
H,5.2165024168,3.2544193709,0.0000376047
H,6.4574534379,0.9957052097,-0.0000357437
H,0.9147287343,3.2369131224,0.0000187614
H,3.0606240562,4.4499445742,0.0000680582
N,1.7788909063,-1.3429558177,-0.0001316787

2d-3 (S_0)

(CAM-B3LYP/6-31+G(d,p) for C, H, N, and O and CAM-B3LYP/LanL2DZ for Pt)

-1358.059242 hartree

C,3.1389251966,2.5553377933,0.0001906697
C,2.6570079053,3.8512691667,-0.001000608
C,3.7660926257,4.7268788834,-0.0003512049
C,4.9014115329,3.9442932062,0.0012184853
N,4.5153723849,2.637176703,0.0014287546
H,5.1482247991,1.8549416231,0.002793989
H,1.6103461783,4.1137747511,-0.0022389022
H,3.7424721115,5.8069226795,-0.0009625159
H,5.9463564729,4.2172315953,0.0021996089
C,2.3780596363,1.3036557102,0.0000675693
C,3.0521601064,0.0740706314,-0.0000440406
C,2.482885789,-1.2078218099,0.0000078216
H,4.1330341337,0.0977801031,-0.0003767661
O,1.1087166881,1.4797260328,0.0000645167
O,1.233753952,-1.4843490832,0.0001915397
C,3.3726333939,-2.3737014398,-0.0001528924
C,4.7493612144,-2.5304141116,-0.0005277399
N,2.8253494545,-3.63068647,0.0000913072
C,5.0219611021,-3.9176923633,-0.0005398412
H,5.4822814321,-1.7370710353,-0.000814957
C,3.8062758247,-4.5723665809,-0.000143875
H,1.8285871727,-3.7800097204,0.0003969332
H,5.9948944754,-4.3874016075,-0.0008072125
H,3.5752122067,-5.6271832245,-0.0000077743
Pt,-0.2844928154,0.0145365153,0.00010669
C,-3.0431855168,-0.7471290836,0.0001868364
C,-1.7077778993,-2.6468883451,0.0003571477
C,-3.0517400217,0.6731459672,0.00005106
C,-4.2269952446,-1.5015721128,0.0002603578
C,-2.8393217201,-3.4697496635,0.0004350384
H,-0.6990878365,-3.042021419,0.0003866982
C,-4.2777895169,1.3616031578,-0.0000027799
C,-1.7865804725,1.3060684078,-0.0000136255
C,-5.4710005818,-0.7772156766,0.0001985262
C,-4.0988372964,-2.9002054573,0.0003883594
H,-2.7108375083,-4.5457857647,0.0005303858
C,-4.2304388289,2.7683343238,-0.0001247215
C,-5.4901135775,0.5827451274,0.0000745674
C,-1.7877369243,2.6907877862,-0.0001272961
H,-6.399003273,-1.3409227026,0.000254359
H,-4.9871692251,-3.5253051485,0.0004486124
C,-3.002005151,3.4060334689,-0.0001805046
H,-5.1520904805,3.3426790396,-0.000167896
H,-6.4413181143,1.1078187881,0.0000319196
H,-0.8465536207,3.2303792186,-0.0001537453
H,-2.9712330576,4.4921343766,-0.0002649313
N,-1.807880106,-1.3231442364,0.0002400768

2d-4 (S_0)

(CAM-B3LYP/6-31+G(d,p) for C, H, N, and O and CAM-B3LYP/LanL2DZ for Pt)

-1358.0540274 hartree

C,-3.1362978563,2.5122736814,-0.034990507
C,-2.7462655092,3.7954276447,0.2951861542
C,-3.8642877844,4.6448577196,0.1221474582
C,-4.9071874335,3.8604669281,-0.3208731587
N,-4.4647633,2.5717190802,-0.3991912373
H,-4.9888293271,1.8090533177,-0.7962146635
H,-1.7597288339,4.0656852475,0.6402098076
H,-3.905674102,5.7094322897,0.3014956949
H,-5.9198487569,4.1202174733,-0.5918475627
C,-2.3478148876,1.2764397935,0.000028766

C,-3.0090326062,0.0374984449,0.0030000108
C,-2.4208700722,-1.24255578,-0.0094102403
H,-4.0905959873,0.0720097032,0.0146730531
O,-1.084318295,1.4747331733,0.0388033487
O,-1.1743655311,-1.4878956124,-0.0768815673
C,-3.2866037784,-2.4275459848,0.0456155008
C,-2.9952217039,-3.7298518954,-0.3103820206
N,-4.6003637183,-2.4057263113,0.4622793662
C,-4.1584338978,-4.5075044862,-0.0992314828
H,-2.0450149746,-4.0611459697,-0.7012644011
C,-5.1292888597,-3.6618785704,0.3919465486
H,-5.0575692951,-1.6134355757,0.8836074395
H,-4.276991555,-5.5649188362,-0.2865149578
H,-6.1451692591,-3.8582557856,0.7014035176
Pt,0.3222101454,0.0181637745,-0.0108701875
C,3.07162996,-0.7625829747,-0.0249802571
C,1.7140165998,-2.6489834558,-0.1037613189
C,3.0917808804,0.6570158036,0.0313933678
C,4.2475513795,-1.5274104025,-0.0463992394
C,2.8397848813,-3.4807809911,-0.1275281965
H,0.699998781,-3.0327658139,-0.1203503142
C,4.3236951999,1.3335630134,0.0644936163
C,1.8316884402,1.3012171249,0.0496187624
C,5.497875085,-0.8147945279,-0.0110145516
C,4.1045566002,-2.9242254536,-0.0997753611
H,2.7007912217,-4.5547645457,-0.166594569
C,4.2885419618,2.7400482141,0.1178877443
C,5.5290770574,0.5440821591,0.041342571
C,1.8451104376,2.6848933066,0.1006375345
H,6.4209982696,-1.3863219747,-0.0270323081
H,4.9865921337,-3.5580239001,-0.1178956248
C,3.0661021082,3.3886029571,0.1341922783
H,5.2152703791,3.3056145946,0.1448700395
H,6.484931268,1.0600421427,0.0670563466
H,0.9089475097,3.2336677335,0.1113096818
H,3.0452409987,4.4742714248,0.1734246366
N,1.8318560264,-1.3272098985,-0.055689518

2d-1 (S_1)
(TD-CAM-B3LYP/6-31+G(d,p) for C, H, N, and O and TD-CAM-B3LYP/LanL2DZ for Pt)
-1357.94466325 hartree
C,3.1711986965,2.5373763087,0.0001127985
C,4.5344632654,2.7928900441,0.0001382469
C,4.7045028144,4.1944470073,0.0001245763
C,3.4432225831,4.7594527062,0.0001922506
N,2.5326440186,3.7518340521,0.0001494597
H,1.5276451808,3.8323653982,0.0001540284
H,5.3212737091,2.0530202274,0.000127318
H,5.6404075727,4.7339689663,0.0001112641
H,3.1370897339,5.7950162753,0.0002313965
C,2.3812642293,1.3099856808,0.0000750077
C,3.042922163,0.0769771767,0.0000368071
C,2.458855748,-1.1935816075,-0.0000233153
H,4.1229764653,0.1123743649,0.0000613151
O,1.1092446999,1.5095814833,0.0000581839
O,1.1967244564,-1.4512617875,-0.0000339545
C,3.3177889256,-2.3729754896,-0.000009013
C,4.6932979809,-2.552916525,0.0000679124
N,2.7472955766,-3.6210037641,0.0000137481
C,4.9404419122,-3.9426338395,-0.0000849831
H,5.4379078568,-1.7704673377,0.0001142671
C,3.7117454943,-4.5763855713,-0.0000131239
H,1.7480939584,-3.7572288923,0.000026003
H,5.9045426679,-4.4300272028,-0.0001539542
H,3.463262873,-5.6272723646,-0.0000305697
Pt,-0.3044268424,0.0196219652,-0.0000116845
C,-3.0405609727,-0.785543316,0.0000163474
C,-1.6734241895,-2.702487704,0.0001159615
C,-3.0705500272,0.6037652163,-0.0000508349
C,-4.2213830671,-1.5559633603,0.0000494261
C,-2.7976278507,-3.5063546831,0.0001509199
H,-0.6704950233,-3.1071479275,0.0001404839
C,-4.2917686589,1.3118501649,-0.000088888
C,-1.784821571,1.2619214743,-0.0000767768
C,-5.4541989875,-0.8245435141,0.0000088225
C,-4.0948595851,-2.9598015818,0.0001187978
H,-2.6590609778,-4.582033515,0.00020401
C,-4.216614182,2.7199296618,-0.0001561325
C,-5.4949508814,0.5467924267,-0.000057625
C,-1.7802597332,2.665179302,-0.0001408709
H,-6.3820706044,-1.3890260921,0.0000334709
H,-4.9719028107,-3.5960568545,0.0001468253
C,-2.9826516987,3.3799404939,-0.0001808673
H,-5.1344831989,3.3014525242,-0.0001872065
H,-6.4517146267,1.0609152693,-0.0000852051
H,-0.8331397155,3.1920796558,-0.0001614299
H,-2.9637967162,4.4649103137,-0.0002319093
N,-1.7650356617,-1.3458602291,0.0000496957

2d-1 (T_1)
(CAM-B3LYP/6-31+G(d,p) for C, H, N, and O and CAM-B3LYP/LanL2DZ for Pt)
-1357.9461321 hartree
C,3.1676101522,2.5390538184,0.0000594027
C,4.5385844466,2.7972964305,0.0000977106
C,4.7073030376,4.1977682682,0.0001080319
C,3.4476336308,4.7662192456,0.0000703281
N,2.5323285602,3.7566113198,0.0000651572
H,1.5278836343,3.8406714431,0.0000491695
H,5.3265476034,2.058587598,0.0001132259
H,5.6438519254,4.7363796514,0.0001325017
H,3.1432134556,5.8020933208,0.0000612272
C,2.3878369308,1.3254635164,0.0000305616
C,3.0459844065,0.0745152095,0.0000365671
C,2.4700710438,-1.1964948536,0.0000184369
H,4.1279283755,0.1094021328,0.0000654883
O,1.097197144,1.495557843,0.000012518
O,1.1783082678,-1.4205932009,0.0000014958
C,3.3076786311,-2.3814527822,0.0000647797
C,4.6833670581,-2.5784991819,0.0001209123
N,2.7247289279,-3.6243597857,0.000102116
C,4.9141740989,-3.9730522302,0.0001652122
H,5.4386927923,-1.8061224626,0.0001413612
C,3.6824374917,-4.59518962,0.0000535895
H,1.7258043951,-3.7547963928,0.0001008483
H,5.8738290761,-4.4694888608,0.000219488
H,3.4216412512,-5.6428233026,0.0000193157
Pt,-0.2915659181,0.0307229483,-0.000010891
C,-3.0241638616,-0.784222594,-0.0000416117

C,-1.646447636,-2.6781889601,-0.0000410604
C,-3.0596814041,0.6265959889,-0.00002989
C,-4.1971772501,-1.5505556346,-0.000060478
C,-2.7682779029,-3.5029818687,-0.0000601791
H,-0.6351409536,-3.060760428,-0.0000355174
C,-4.2881968901,1.3089482319,-0.000034761
C,-1.7960608716,1.2829000119,-0.000001616
C,-5.4355333796,-0.8399386125,-0.0000684771
C,-4.0490031013,-2.9654364714,-0.0000696358
H,-2.6219290262,-4.5774706442,-0.0000686696
C,-4.2344034838,2.7279526591,-0.0000154648
C,-5.483630546,0.5327743015,-0.0000549281
C,-1.7995219693,2.6752223234,-0.0000020959
H,-6.358365895,-1.4125144964,-0.0000828326
H,-4.923969729,-3.6065468155,-0.000084169
C,-3.0183524286,3.3850180743,0.0000013649
H,-5.1604661443,3.2960391372,-0.000019289
H,-6.4437429812,1.0404231648,-0.0000582743
H,-0.8590913919,3.2146697685,0.0000034582
H,-3.0011355479,4.4706026589,0.0000078574
N,-1.7697630247,-1.3329248672,-0.0000312846

2d-4·Cl⁻ (S_0)
(CAM-B3LYP/6-31+G(d,p) for C, H, N, O, and Cl and CAM-B3LYP/LanL2DZ for Pt)

C,2.7933169828,2.479732485,-0.0408987133
C,2.35901235,3.7983460421,-0.038254892
C,3.5039574034,4.6223813414,-0.044934216
C,4.6030251691,3.777947262,-0.051421048
N,4.1689350071,2.4962849826,-0.0487909697
H,4.8100767237,1.6857773968,-0.0527280364
H,1.32312287,4.1029527679,-0.0321208549
H,3.5315651738,5.703279452,-0.0450113153
H,5.6613413534,3.994807701,-0.0576252239
C,1.9829636328,1.2624384491,-0.0361219801
C,2.6207313575,0.0185051639,-0.0391656137
C,2.0281675036,-1.2518613662,-0.0359010298
H,3.7004491839,0.0409679524,-0.044893308
O,0.7094672405,1.484900926,-0.0293549266
O,0.7654560995,-1.4896794876,-0.0291265877
C,2.8849618715,-2.4389789674,-0.0406492655
C,2.501001132,-3.7734073273,-0.0378100038
N,4.2597144634,-2.4053254748,-0.0487953469
C,3.6756888408,-4.5548066001,-0.0447567737
H,1.4782255434,-4.1200110508,-0.031510574
C,4.7420095627,-3.6693993287,-0.0511411785
H,4.8712428756,-1.5707544392,-0.0529672786
H,3.7439931141,-5.6339316044,-0.0448872022
H,5.8077428308,-3.8461523194,-0.0573722882
Pt,-0.68296738,0.031720251,-0.0212715227
C,-3.4342435674,-0.7543859524,-0.0059302917
C,-2.0698404582,-2.6382463695,-0.0131272417
C,-3.4572803614,0.6675678155,-0.0060885894
C,-4.6077884634,-1.5239759595,0.000705421
C,-3.1939533336,-3.4743586054,-0.0067539461
H,-1.0511757359,-3.0121507279,-0.0187035148
C,-4.6930957695,1.3408207672,0.0006054225
C,-2.1953347144,1.3139049021,-0.0132061341
C,-5.8602237077,-0.8139397459,0.0074917465
C,-4.4606210782,-2.9216606926,0.0001536553
H,-3.0506754875,-4.5487367381,-0.0073496919
C,-4.6644960865,2.7478235141,0.0001528122
C,-5.8952988822,0.5462644684,0.0074129438
C,-2.2198833012,2.7013979724,-0.0133694292
H,-6.7821370999,-1.3885921667,0.0126997472
H,-5.3414343408,-3.5580565898,0.0051426477
C,-3.4419950525,3.4004209361,-0.0067529445
H,-5.5939813327,3.3104849248,0.005170497
H,-6.8532922415,1.0600540314,0.0126055745
H,-1.2835767771,3.2505153897,-0.0186801292
H,-3.4249369881,4.4874311112,-0.007082893
N,-2.1927813016,-1.3167554156,-0.0126853598
Cl,6.2593492358,0.0750561133,-0.0614739029

2d-4·Cl⁻ (S_1)
(CAM-B3LYP/6-31+G(d,p) for C, H, N, O, and Cl and CAM-B3LYP/LanL2DZ for Pt)

C,-2.7618142665,2.4827408276,-0.0000954107
C,-2.3367460493,3.8084299076,-0.0000444439
C,-3.4843499202,4.6209090782,-0.0000442745
C,-4.579117131,3.7654396534,-0.0001166067
N,-4.1388765218,2.489747552,-0.0001778238
H,-4.7774456701,1.6734540421,-0.0002564882
H,-1.3034630678,4.1215383339,0.0000018257
H,-3.5214753735,5.7012188266,-0.0000005265
H,-5.6386795649,3.9764841963,-0.0001471676
C,-1.9447859822,1.2788063674,-0.0001054803
C,-2.5787312053,0.0249960536,-0.0001711138
C,-1.9801699388,-1.2352786819,-0.0002034323
H,-3.6588879074,0.039963362,-0.0001892272
O,-0.6746676915,1.4982087671,-0.0000748116
O,-0.7028813884,-1.4631518674,-0.0001719486
C,-2.8119309003,-2.4309145271,-0.0002144767
C,-2.4043483858,-3.7599651047,-0.0002284836
N,-4.1880546669,-2.4191010304,-0.0001687441
C,-3.5644371185,-4.5586651037,-0.0002620948
H,-1.3757112374,-4.0879978493,-0.0002404041
C,-4.6466742205,-3.690762782,-0.0002169987
H,-4.811383523,-1.5935773958,-0.0001184306
H,-3.6146440093,-5.6384813036,-0.000299485
H,-5.708913918,-3.8876999728,-0.0002090378
Pt,0.7323750837,0.01724736,-0.0001124836
C,3.4768853295,-0.7614026142,0.0000755347
C,2.1265134022,-2.6961080684,0.0000680547
C,3.4920074948,0.6364997225,0.0000284363
C,4.6644254581,-1.5157539596,0.0001703122
C,3.258656763,-3.4855928885,0.000159691
H,1.1239087322,-3.103096847,0.0000251346
C,4.7052726888,1.3557476229,0.0000752608
C,2.2046219983,1.283357897,-0.0000669283
C,5.8889410072,-0.7694183222,0.0002140516
C,4.5514751505,-2.9244630507,0.0002128149
H,3.1315114866,-4.5631556388,0.0001902359
C,4.6144089138,2.7676250378,0.0000248627
C,5.9157240481,0.6030806904,0.0001691693
C,2.178211269,2.6785792382,-0.0001122024
H,6.8233312359,-1.3245597015,0.0002866349
H,5.4362971659,-3.5508887124,0.0002850827
C,3.3803355029,3.4093821947,-0.0000660528
H,5.5283411921,3.3566890062,0.0000588279

H,6.8674954434,1.1277844742,0.0002065764
H,1.2213934768,3.1900077595,-0.000184943
H,3.3463476866,4.4947238542,-0.0001023097
N,2.2140333289,-1.3357255596,0.000024407
Cl,-6.1817592,0.0688451563,-0.0002050826

2d-4·Cl⁻ (T_1)
(CAM-B3LYP/6-31+G(d,p) for C, H, N, O, and Cl and CAM-B3LYP/LanL2DZ for Pt)
C,-2.7853737431,2.4664098999,-0.0000690061
C,-2.3601622869,3.80534261,0.0000284666
C,-3.5069836696,4.6166958729,-0.0001048507
C,-4.606291262,3.7676268787,-0.0003120583
N,-4.1684369699,2.484102126,-0.000321142
H,-4.8090236538,1.6748318614,-0.0004825073
H,-1.3258718554,4.1137827107,0.0001941469
H,-3.542337761,5.6974868276,-0.0000689104
H,-5.6647879263,3.9829808014,-0.0004766337
C,-1.9649408747,1.2921523565,0.0000161307
C,-2.5907981772,0.0018273336,-0.000091116
C,-1.9969037878,-1.3012811608,-0.0000355357
H,-3.6773222634,0.0176709688,-0.0002279606
O,-0.6656489371,1.4961108743,0.0002012747
O,-0.7061324703,-1.494295924,0.0001358851
C,-2.8397085303,-2.467380411,-0.0001361616
C,-2.4402931514,-3.8098943453,-0.0001208742
N,-4.2212507101,-2.4586207488,-0.0002428982
C,-3.6039790958,-4.6023490805,-0.0002272442
H,-1.4132886821,-4.142544761,-0.0000307116
C,-4.6850688354,-3.7335036103,-0.0003177415
H,-4.8449590401,-1.6360262742,-0.0002535755
H,-3.6590309359,-5.6823683188,-0.0002401599
H,-5.747627608,-3.9276688842,-0.0004091771
Pt,0.7339283358,0.0467560817,0.0001421111
C,3.484037508,-0.730456652,0.0000886335
C,2.1203848922,-2.6163995778,0.0001294361
C,3.503429388,0.6916191576,0.0000775282
C,4.6584972487,-1.4972191395,0.0000654515
C,3.2472594302,-3.4501613673,0.0001060586
H,1.1012491212,-2.9894488188,0.0001557942
C,4.7368210996,1.3685579207,0.0000450232
C,2.2389529473,1.3338088371,0.0000992236
C,5.908606079,-0.7837974204,0.0000326184
C,4.5130700857,-2.8963732288,0.000074953
H,3.1058454046,-4.5248717687,0.0001133535
C,4.7025040532,2.7762893308,0.0000345855
C,5.9406230616,0.5771185691,0.0000234508
C,2.2573415155,2.7211324614,0.0000884562
H,6.8322652872,-1.355794993,0.0000144023
H,5.3946817518,-3.5316933504,0.0000570253
C,3.4778791212,3.4243599767,0.0000562816
H,5.6299246632,3.3425015241,0.0000091664
H,6.8976403206,1.0928504936,-0.0000021517
H,1.3179298904,3.264940749,0.0001056105
H,3.4573225557,4.511388029,0.0000478637
N,2.242603635,-1.294342438,0.0001212084
Cl,-6.184010168,0.033894021,-0.0004237232

2e-1 (S_0)
(CAM-B3LYP/6-31+G(d,p) for C, H, N, O, and S and CAM-B3LYP/LanL2DZ for Pt)
-1756.9240713 hartree
C,1.7407136192,3.4417439282,0.2332222824
C,2.2206138739,5.6484119155,0.4247272491
C,0.8700384832,5.4663433869,0.6802019453
N,0.5939136237,4.1376377934,0.5537678556
C,1.6841470204,1.9916012168,0.0662025667
C,2.8769544256,1.2647952364,-0.0085023073
C,3.0147282186,-0.1319564323,-0.0923747642
H,3.7917983179,1.8405911325,0.0321521051
O,0.4728743417,1.5231664375,0.0202001083
O,2.0531888585,-0.9919871739,-0.0793634733
C,4.3576781259,-0.70138798,-0.1912347112
N,4.5099204855,-2.0653800615,-0.3137701356
C,6.5623503637,-1.2137968796,-0.3240274917
C,5.8308790569,-2.3895765746,-0.3977708157
Pt,-0.0152509136,-0.4542848667,-0.021439799
C,-1.9935847068,-2.5686774644,0.2212524838
C,-2.7105712445,-1.329174916,0.1402171317
C,-2.5180888732,-3.860229417,0.3796535026
C,-1.9865867698,-0.165999741,-0.0363128626
C,-1.6601282897,-4.9505566329,0.4206477051
H,-2.0597444143,-5.9526740857,0.5430637269
N,-0.6358258131,-2.392003253,0.1074803016
C,-2.8669544522,0.9646338634,-0.2096352736
C,-4.2407494124,0.609635713,-0.1011701926
C,-0.2803909973,-4.747673175,0.3059940855
H,0.4196072465,-5.5746056725,0.3360325435
C,2.7710669305,4.3761572336,0.1473525861
C,5.6388190616,-0.1515547885,-0.1959474124
H,-3.5912583063,-3.9885321115,0.4694292056
C,0.1894115878,-3.449993587,0.1537043831
H,6.1538656602,-3.4148618006,-0.5032718679
H,7.6397373842,-1.1359184273,-0.3586926524
H,5.8784184177,0.8985266442,-0.1117282271
H,3.7988712372,4.1630576242,-0.1089717395
H,2.7449140982,6.5934091268,0.4349568487
H,0.1003876975,6.1774219855,0.9420143906
S,-4.4613622119,-1.1144413859,0.1692372629
C,-5.259480724,1.5582873552,-0.230302998
C,-2.5470698204,2.3052275395,-0.5065244346
C,-3.5578928953,3.2460386193,-0.6452272681
C,-4.9074566708,2.8776145407,-0.4956715025
H,-1.5059608143,2.5657503211,-0.6542100802
H,-6.302548079,1.2710686587,-0.1378551614
H,-5.6866134779,3.6265610632,-0.6042994912
H,-3.3085964816,4.2761092898,-0.883820145
H,-0.2916384914,3.6803492291,0.7126578833
H,1.2426176333,-3.2108083782,0.0704376573
H,3.7201810304,-2.6897805894,-0.352658694

2e-2 (S_0)
(CAM-B3LYP/6-31+G(d,p) for C, H, N, O, and S and CAM-B3LYP/LanL2DZ for Pt)
-1756.9188848 hartree
C,1.8552514174,3.378663299,0.1380937422
C,1.5525049865,5.5987546235,0.4872215311
C,2.8242471264,5.4065714931,-0.0225049807
N,3.0053770315,4.0640798808,-0.2126822773
C,1.7275455648,1.921514011,0.0662010868

C,2.889469751,1.1348720137,0.0575424082
C,2.9663932993,-0.2683299265,-0.014338042
H,3.8323979417,1.6550704612,0.1676835451
O,0.5036327156,1.5069377149,0.0283105212
O,1.9673260785,-1.0786092215,-0.0665991629
C,4.2853113901,-0.9015694395,-0.0334840508
N,4.3776834914,-2.2700942991,-0.1614158473
C,6.4661767161,-1.5232792212,-0.0296304672
C,5.6833276789,-2.6598912061,-0.16377075
Pt,-0.0798503813,-0.4456140462,-0.0122126763
C,-2.1511842374,-2.481838066,0.0877204089
C,-2.8121789433,-1.2110906066,0.0591602257
C,-2.734323254,-3.7567770448,0.1561137581
C,-2.0376794654,-0.0687736734,-0.0135475197
C,-1.9246080315,-4.8835125833,0.1688493877
H,-2.3696902355,-5.872482127,0.2222292633
N,-0.7842458457,-2.3587424231,0.0322004166
C,-2.8668879246,1.1103023161,-0.0942064077
C,-4.2561881778,0.8034835406,-0.0527314948
C,-0.5339384461,-4.7350267065,0.1150055966
H,0.1296303321,-5.5919377382,0.1254997451
C,0.9403711972,4.3262154288,0.582284204
C,5.5898083549,-0.4171022332,0.0505518624
H,-3.8145259343,-3.8427232698,0.1999062356
C,-0.006058279,-3.4527210959,0.0492334626
H,5.9610156984,-3.699272705,-0.2595183465
H,7.546036388,-1.4995985806,0.0071477362
H,5.8766201816,0.618215051,0.1656956732
H,-0.0428638197,4.0988644472,0.9661603225
H,1.1203025757,6.5496565337,0.7646396882
H,3.6023911874,6.1147141054,-0.2669444363
S,-4.5527126167,-0.9266939049,0.0659615104
C,-5.2342062298,1.7999223846,-0.1159311087
C,-2.4848652884,2.4608180021,-0.227241385
C,-3.4558854587,3.4498200128,-0.2956779153
C,-4.8228457447,3.1234377054,-0.234644614
H,-1.430034313,2.6982129274,-0.2850105383
H,-6.2897502544,1.5477071201,-0.0795427051
H,-5.568961719,3.9112416351,-0.2878064882
H,-3.1587926493,4.4891827147,-0.4021331209
H,3.7969232174,3.6469566236,-0.675758989
H,1.0584303603,-3.2560425038,0.0125827631
H,3.5616493672,-2.8537631437,-0.2549599604

2e-3 (S_0)

(CAM-B3LYP/6-31+G(d,p) for C, H, N, O, and S and CAM-B3LYP/LanL2DZ for Pt)

-1756.9203184 hartree
C,1.8611511207,3.3477797904,0.256146169
C,2.4288188013,5.531602877,0.4728546434
C,1.0681941984,5.4032022234,0.7068164
N,0.7400731126,4.0881161378,0.5661095934
C,1.742263134,1.9020334897,0.0748981001
C,2.9035220417,1.1206785639,0.0162880376
C,2.9773080504,-0.28610771,-0.0682637062
H,3.8347719605,1.6602474651,0.1354919602
O,0.5136702476,1.4910743999,0.0037434883
O,1.9760836849,-1.0882945773,-0.0401708832
C,4.2881692245,-0.9299954255,-0.1680104907
N,5.454894027,-0.2616504958,-0.4972692626
C,6.0278594692,-2.3859428353,-0.1582994783
C,6.5063486062,-1.1348491187,-0.5078969997
Pt,-0.0584940414,-0.4680899856,-0.0253708378
C,-2.0934073646,-2.5275387428,0.1830501252
C,-2.7765754632,-1.2664389366,0.115875005
C,-2.6506800289,-3.8072051372,0.3175452658
C,-2.0211494916,-0.1222631567,-0.0516451431
C,-1.8194765227,-4.9196097524,0.3328450206
H,-2.2444074941,-5.9135482968,0.4358552251
N,-0.7317678659,-2.3851823185,0.0745586498
C,-2.8695846376,1.0339136103,-0.2120308576
C,-4.2528228245,0.7171006069,-0.1067173182
C,-0.4360525747,-4.7505872984,0.2134650559
H,0.241967346,-5.5961218273,0.217923087
C,2.9299752667,4.239659743,0.1922796842
C,4.6345580775,-2.2587937533,0.0484509898
H,-3.7266405695,-3.9105902251,0.4063976142
C,0.0698464667,-3.4628910772,0.0881014493
H,7.5027210943,-0.8062043427,-0.7648992747
H,6.6211646057,-3.2839833825,-0.0607758966
H,3.9441390112,-3.0318860451,0.3504875359
H,3.9520619291,3.987707308,-0.0514813085
H,2.9913100292,6.4540541226,0.4988723607
H,0.3245961383,6.1434697806,0.9628464172
S,-4.5208811047,-1.0028676074,0.1473312332
C,-5.2436402955,1.696304418,-0.2247624514
C,-2.5107595649,2.3682075974,-0.491644003
C,-3.4939190016,3.3395111692,-0.6191281993
C,-4.8536075142,3.0080835245,-0.4746012081
H,-1.4621179111,2.6002943303,-0.6355082474
H,-6.2946439954,1.4385122344,-0.1351170128
H,-5.6108441181,3.7804231984,-0.5742089063
H,-3.2152934484,4.3651166982,-0.8443493486
H,-0.1659117402,3.6655646367,0.7059953885
H,1.1287545907,-3.2490723836,-0.0034461531
H,5.4958063086,0.6958874861,-0.8071998024

2e-4 (S_0)

(CAM-B3LYP/6-31+G(d,p) for C, H, N, O, and S and CAM-B3LYP/LanL2DZ for Pt)

-1756.9142772 hartree
C,-1.9130214798,3.3139685552,0.0099087237
C,-1.6985797539,5.5524188165,0.3005437955
C,-2.9244217355,5.3055776849,-0.2900114766
N,-3.0563585357,3.951215493,-0.4413936937
C,-1.7403074812,1.8584350991,-0.0006393599
C,-2.8819328807,1.0383922253,-0.0139914323
C,-2.9179613875,-0.3724914397,-0.041855917
H,-3.8360534052,1.5508572555,0.0178851762
O,-0.5072144067,1.4787621693,0.0238583974
O,-1.8952682757,-1.1402893803,-0.1160128583
C,-4.2116528811,-1.0591082873,0.0075998941
N,-5.3909381456,-0.4630216066,0.4220245268
C,-5.9129757505,-2.551819959,-0.145614164
C,-6.4184001502,-1.3635027374,0.3510807746
Pt,0.1228545549,-0.463489243,-0.0422970921
C,2.2072081534,-2.4837114428,0.019445214
C,2.8592915533,-1.2064252286,0.0407942309
C,2.7969483484,-3.7562410127,0.0498998997
C,2.0774015972,-0.0678629537,-0.0013078911

C,1.9918630483,-4.8867726946,0.0185056876
H,2.4418754162,-5.8747564606,0.0421734471
N,0.8404843934,-2.3678159388,-0.0434735151
C,2.8980415365,1.1193612762,-0.0208297669
C,4.2890537694,0.8223189881,0.030789845
C,0.6013243484,-4.744010645,-0.0410763171
H,-0.0576907946,-5.604157185,-0.0639089314
C,-1.0587669711,4.302334031,0.482250511
C,-4.5260951244,-2.3634106729,-0.353761178
H,3.8772330566,-3.8385737563,0.0990496899
C,0.0636553022,-3.4636393652,-0.0687416211
H,-7.4177298214,-1.0957509536,0.6617134741
H,-6.4838575641,-3.4488911479,-0.3389331199
H,-3.8208716852,-3.0763368879,-0.7540956399
H,-0.0994130406,4.1176595049,0.9422136084
H,-1.3136797962,6.5244302302,0.574493544
H,-3.7006001842,5.981744092,-0.6172610725
S,4.597352531,-0.9090511974,0.0859607878
C,5.2587891234,1.8287858449,0.0238584676
C,2.5055478951,2.4707866769,-0.1023008555
C,3.4684735016,3.4700304303,-0.1145163065
C,4.8372696545,3.1526425392,-0.0465351443
H,1.4492157546,2.7012138031,-0.1659396241
H,6.3159634812,1.5844491767,0.0667616447
H,5.5769917696,3.948210726,-0.0554627036
H,3.1641063498,4.5106440848,-0.1813598989
H,-3.7861978073,3.4982034411,-0.9682405903
H,-1.0025348828,-3.2713102432,-0.1067218732
H,-5.4473484576,0.4393395052,0.8664122433

2e-1 (S_1)
(TD-CAM-B3LYP/6-31+G(d,p) for C, H, N, O, and S and TD-CAM-B3LYP/LanL2DZ for Pt)
-1756.12148413 hartree
C,-1.7043328086,3.4202075891,-0.2296242168
C,-2.2043394657,5.6155398243,-0.3942801951
C,-0.8592085093,5.4479788932,-0.6610224458
N,-0.5702220234,4.1246805895,-0.550719762
C,-1.6330844238,1.9690314394,-0.0761404901
C,-2.8181277766,1.2352633852,0.0145039598
C,-2.9382008391,-0.1586772465,0.0701722071
H,-3.7382776685,1.8016764944,-0.0034743935
O,-0.4300708604,1.5089722775,-0.0568527176
O,-1.9726991709,-1.0062158967,0.0333672792
C,-4.2693867758,-0.7521519039,0.1585248676
N,-4.3992735649,-2.1172403449,0.2114974023
C,-6.4566671616,-1.2968857997,0.2938441389
C,-5.7108302038,-2.4603758536,0.2939110887
Pt,0.0751452334,-0.4788754212,-0.0133945269
C,2.0440237763,-2.5875118299,-0.2056514461
C,2.7453880955,-1.3638977663,-0.1412082659
C,2.5578847804,-3.8857955932,-0.3272925213
C,1.9828249823,-0.1645574844,0.0391403029
C,1.7114652139,-4.9720986223,-0.3483661273
H,2.101161154,-5.9790430012,-0.4377507688
N,0.6558087687,-2.3769213201,-0.1081263083
C,2.8517213807,0.9631618196,0.2221079582
C,4.2210452012,0.6290041323,0.086561036
C,0.3127407353,-4.7393730008,-0.2476401579
H,-0.3917501924,-5.5628369605,-0.2628036119
C,-2.7396078956,4.3360512548,-0.1261253575
C,-5.5487090211,-0.2189325494,0.2089908782
H,3.6329939986,-4.0171195658,-0.3994411782
C,-0.1606340376,-3.4563506738,-0.1326508989
H,-6.0202398553,-3.4936381112,0.3467110427
H,-7.5337974386,-1.2349391415,0.3488720813
H,-5.8019319719,0.830802618,0.1867673686
H,-3.7642003336,4.1105053947,0.1310116172
H,-2.7378880986,6.5549127032,-0.3924098607
H,-0.0992498887,6.1691583142,-0.9227687866
S,4.4468659076,-1.1027022352,-0.2208578094
C,5.2312944541,1.5642487897,0.2129236563
C,2.531218289,2.2913887784,0.5473440387
C,3.5411834601,3.2365636787,0.6843383793
C,4.8806494044,2.8852510377,0.5075733388
H,1.4928495901,2.5452941824,0.7203173342
H,6.2731390078,1.2831171351,0.0972285508
H,5.6585194819,3.6338937635,0.6153995256
H,3.286355024,4.2587314201,0.9467061642
H,0.3195678439,3.6796195691,-0.7212464637
H,-1.2171114163,-3.2324083417,-0.060887166
H,-3.6043193813,-2.7368874202,0.1948392596

2e-1 (T_1)
(CAM-B3LYP/6-31+G(d,p) for C, H, N, O, and S and CAM-B3LYP/LanL2DZ for Pt)
-1756.1552279 hartree
C,-1.719213562,3.4171970514,-0.2260553629
C,-2.215495279,5.6130688314,-0.3929263336
C,-0.8667091599,5.4455824259,-0.6394067567
N,-0.5804452575,4.121516374,-0.5286235464
C,-1.6489762365,1.9645040855,-0.075555954
C,-2.8336350782,1.2296882927,0.0044347466
C,-2.9531424614,-0.1644970733,0.0670975576
H,-3.7538730096,1.7956738206,-0.0224449257
O,-0.4453470911,1.507434858,-0.0483979469
O,-1.9879956444,-1.0096410233,0.0447458889
C,-4.2888969948,-0.7549606014,0.1484843276
N,-4.4226202794,-2.119068527,0.2076388462
C,-6.4786154044,-1.2946209487,0.2693680479
C,-5.7364936873,-2.4594756586,0.2816591633
Pt,0.0650941685,-0.4635168029,-0.0013469238
C,2.0552325061,-2.5737937168,-0.2039311942
C,2.7560807199,-1.3762306252,-0.1661437804
C,2.5735112491,-3.8903043145,-0.329867776
C,1.9799775025,-0.1289591895,0.0293615055
C,1.7209305227,-4.9618137522,-0.3338280744
H,2.1083745737,-5.9707804372,-0.4258772238
N,0.6763291446,-2.3833824299,-0.0900774951
C,2.855726053,0.9661968486,0.2158509136
C,4.2316856623,0.6287347639,0.0659779021
C,0.3293778674,-4.7410367855,-0.215223033
H,-0.3738943447,-5.5646181565,-0.2171706188
C,-2.7554533159,4.3327270334,-0.1356640127
C,-5.566512772,-0.2187533233,0.1860207959
H,3.6461665179,-4.0259485797,-0.4160640525
C,-0.1365104433,-3.4430806495,-0.0982335238
H,-6.0486380795,-3.4917468638,0.3374896313
H,-7.5560021131,-1.2292740202,0.3154958327
H,-5.8170190787,0.8314291544,0.156425673

H,-3.7840605746,4.1069061376,0.1047215243
H,-2.7487299754,6.5526542165,-0.3969429914
H,-0.1020567501,6.1669344691,-0.8865493939
S,4.4573945304,-1.1133596969,-0.2737935957
C,5.2441101712,1.5488179981,0.1930184567
C,2.5531426668,2.3028477821,0.5536764981
C,3.5729870686,3.2377502019,0.690254992
C,4.9079424866,2.8789751392,0.5021384657
H,1.5186362119,2.5646745149,0.7380114355
H,6.2832076038,1.2612690073,0.0671493545
H,5.693931093,3.6188188062,0.6091682696
H,3.3278168372,4.2601651976,0.9615038748
H,0.312217513,3.6757994757,-0.6822950242
H,-1.1909286019,-3.2109986847,-0.011669285
H,-3.6289224758,-2.7398856253,0.2003131217

2e-4·Cl⁻ (S_0)

(CAM-B3LYP/6-31+G(d,p) for C, H, N, O, S, and Cl and CAM-B3LYP/LanL2DZ for Pt)

C,-2.2373533025,2.7504094863,0.2060997309
C,-2.4441845341,4.9821595448,0.4582493927
C,-3.6912249984,4.4339345593,0.2175981685
N,-3.56120643,3.0965721225,0.0712822077
C,-1.7451915899,1.3772445686,0.1276069195
C,-2.6695600751,0.3334953681,0.1216245804
C,-2.403727288,-1.0378898774,0.0520200666
H,-3.7097062715,0.6175895015,0.1816290324
O,-0.4548921125,1.278226658,0.074672462
O,-1.2338243019,-1.5706134554,0.044437362
C,-3.5177638206,-1.9793111059,-0.0074531045
N,-4.836149795,-1.6086877596,-0.1248867764
C,-4.8012935156,-3.8332449981,-0.0464982982
C,-5.6107292636,-2.7153647072,-0.1512538284
Pt,0.5392140632,-0.4771347772,0.0402741666
C,2.98857865,-2.0185968408,0.0843550217
C,3.3650920055,-0.633668475,0.050193023
C,3.8272234375,-3.1351869337,0.1255441919
C,2.3666322347,0.30565293,-0.0020728764
C,3.272525435,-4.4021196419,0.1390586613
H,3.9163399282,-5.2756625429,0.1706756149
N,1.6362281891,-2.18621763,0.0602451741
C,2.9322912112,1.6325480959,-0.0929603354
C,4.3441042459,1.6329251126,-0.0811474832
C,1.8873135623,-4.5496129193,0.1112469749
H,1.4194861285,-5.5264597958,0.1193320646
C,-1.5243046261,3.9159591302,0.4495844184
C,-3.4764911087,-3.3655397425,0.0418406534
H,4.9013894185,-2.9909222651,0.1454932526
C,1.1011687917,-3.4115457766,0.0728293938
H,-6.683070503,-2.6274843249,-0.2460911625
H,-5.134248495,-4.861643603,-0.0356515784
H,-2.5759317295,-3.9531458624,0.14080621
H,-0.4613088618,3.9649481891,0.6300439916
H,-2.2292609904,6.0284555501,0.6240568208
H,-4.6632292092,4.8981121747,0.1382249148
S,5.0008954148,0.0124275834,0.0230846657
C,5.0747151124,2.8175482553,-0.1592837601
C,2.2571260495,2.8558535738,-0.2101743297
C,2.980343805,4.0312567955,-0.2934073116
C,4.3816215323,4.0124301854,-0.2624468741
H,1.1742614245,2.8411220647,-0.2412223777
H,6.1600092107,2.8043424057,-0.1455707731
H,4.9340794918,4.9452495823,-0.3267813767
H,2.4588954151,4.978751639,-0.3866392524
H,-4.3622892604,2.4682467412,-0.1174564188
H,0.0167791211,-3.4319907308,0.0507568038
H,-5.2167727188,-0.6479850647,-0.2117059268
Cl,-6.1263293169,1.2715749021,-0.3434824462

2e-4·Cl⁻ (S_1)

(CAM-B3LYP/6-31+G(d,p) for C, H, N, O, S, and Cl and CAM-B3LYP/LanL2DZ for Pt)

C,-2.2015867209,2.7338878931,0.2049729528
C,-2.4225535959,4.9673866839,0.4607889469
C,-3.6757149058,4.4024107196,0.2766714235
N,-3.5352753994,3.0669931847,0.1273350432
C,-1.6964425637,1.3676769879,0.10708893
C,-2.6147763933,0.3142562325,0.0563327625
C,-2.341314999,-1.0558857778,0.0135911995
H,-3.6583125915,0.5912246836,0.0665399653
O,-0.4057421653,1.273904073,0.0818674444
O,-1.164485099,-1.5873301451,0.0396596801
C,-3.4428913425,-2.0078059795,-0.0529748361
N,-4.7709874147,-1.6542723724,-0.1218630246
C,-4.6997134492,-3.8814469921,-0.1365971396
C,-5.5304841719,-2.7707232299,-0.1730576898
Pt,0.6072172753,-0.5015104432,0.0447860034
C,3.0582614553,-2.0318055984,0.0857227147
C,3.416594709,-0.666732067,0.0436843148
C,3.894434534,-3.1571913903,0.1286196679
C,2.3728789067,0.3063019645,-0.0180800457
C,3.3567802113,-4.424751873,0.1524284305
H,3.9970318966,-5.2990112805,0.1837456323
N,1.6601109374,-2.1879234782,0.0692313277
C,2.9235578103,1.6286834869,-0.1267546187
C,4.3391715188,1.6454269585,-0.1031178872
C,1.9397558558,-4.5595718987,0.1334819349
H,1.4710077639,-5.5367700517,0.1511378725
C,-1.4917939481,3.9111587217,0.415774227
C,-3.3794874956,-3.3967762133,-0.0616701821
H,4.9698719126,-3.0076741155,0.1405023027
C,1.1460843667,-3.4402784472,0.093047103
H,-6.6065636955,-2.6959769156,-0.2332264012
H,-5.0167862359,-4.9146601345,-0.1616174708
H,-2.4671861499,-3.9725406907,-0.016206252
H,-0.4227034074,3.9763536913,0.5463620105
H,-2.2128071943,6.0166434823,0.6147262127
H,-4.656381915,4.8542653725,0.2420733327
S,5.0093820134,0.0116034757,0.0348600138
C,5.0706663703,2.8169785502,-0.1918091774
C,2.2537014393,2.8545377386,-0.2681137551
C,2.9815241836,4.0340607844,-0.3618090942
C,4.3782389325,4.0238993144,-0.319667243
H,1.1717976735,2.8457992476,-0.3070118936
H,6.1560579791,2.8021143339,-0.1675982834
H,4.9323668713,4.9545133168,-0.3925478363
H,2.4550686756,4.977191054,-0.4729322968
H,-4.340289984,2.4321893303,-0.0135262
H,0.063745895,-3.4895189534,0.079070795
H,-5.170149449,-0.6993163315,-0.1461426529

Cl,-6.1094069004,1.2274160978,-0.1933512636

2e-4·Cl⁻ (T₁)
(CAM-B3LYP/6-31+G(d,p) for C, H, N, O, S, and Cl and CAM-B3LYP/LanL2DZ for Pt)

C,-2.2234215592,2.7343021664,0.181335229
C,-2.4534429342,4.9707020759,0.4031651074
C,-3.7049835092,4.3979626238,0.2417457098
N,-3.5590460042,3.0599083494,0.1120428928
C,-1.7121714864,1.3662876694,0.0971031805
C,-2.6272551597,0.309861566,0.0606314402
C,-2.3490943202,-1.0605265786,0.0198543113
H,-3.671484008,0.5837301637,0.0777393021
O,-0.4220163272,1.2796556088,0.0691410352
O,-1.1731753066,-1.5845390327,0.0325560851
C,-3.4527384568,-2.0168220825,-0.0318369176
N,-4.781423636,-1.6659154443,-0.08927607
C,-4.709033301,-3.8923755444,-0.0945544235
C,-5.5411448048,-2.783864523,-0.127895149
Pt,0.5997084496,-0.4777182712,0.032432748
C,3.0652498516,-2.0205255867,0.0636487767
C,3.4353711045,-0.6820850729,0.0396113515
C,3.9050505082,-3.1659181649,0.0968402108
C,2.3652798506,0.3409008393,-0.0144762486
C,3.3484657509,-4.4178532806,0.1103934417
H,3.9820688403,-5.2986639791,0.1344134505
N,1.678487368,-2.1840897279,0.0473309411
C,2.9376476905,1.6326372199,-0.1087376207
C,4.3610544401,1.6410134543,-0.0861853615
C,1.9409681397,-4.552855732,0.0918876936
H,1.4668630691,-5.5266561697,0.1021887194
C,-1.5175322941,3.9168238259,0.3648829408
C,-3.387534371,-3.404478586,-0.0350035066
H,4.9811624109,-3.0285851015,0.1088112435
C,1.1544585958,-3.4110118329,0.0608419517
H,-6.6177799149,-2.7099053587,-0.1776388446
H,-5.024602286,-4.9263401032,-0.1116126528
H,-2.4743843438,-3.9795098204,0.0043805922
H,-0.4471928044,3.9892332207,0.4796508249
H,-2.2463037309,6.023328017,0.5369641177
H,-4.688343823,4.8441060673,0.2102419299
S,5.0299080297,-0.0122703302,0.0422223595
C,5.1031753735,2.7962611087,-0.1639332142
C,2.284951097,2.8772142919,-0.2332370044
C,3.0287564971,4.0478722774,-0.314512472
C,4.4245286562,4.0220526911,-0.2772768926
H,1.2028628752,2.8821767031,-0.2685465132
H,6.1885438916,2.7685627005,-0.1418734447
H,4.9917210012,4.9451379027,-0.340461343
H,2.5133339942,4.9987546525,-0.4119534419
H,-4.3618418116,2.4199937346,-0.0108304038
H,0.0702377932,-3.4444060769,0.047017267
H,-5.1825667763,-0.7120294299,-0.1135572378
Cl,-6.1398703088,1.2078698999,-0.1612490914

[S12] *Gaussian 09*, revision D.01, M. J. Frisch, G. W. Trucks, H. B. Schlegel, G. E. Scuseria, M. A. Robb, J. R. Cheeseman, G. Scalmani, V. Barone, B. Mennucci, G. A. Petersson, H. Nakatsuji, M. Caricato, X. Li, H. P. Hratchian, A. F. Izmaylov, J. Bloino, G. Zheng, J. L. Sonnenberg, M. Hada, M. Ehara, K. Toyota, R. Fukuda, J. Hasegawa, M. Ishida, T. Nakajima, Y. Honda, O. Kitao, H. Nakai, T. Vreven, J. A. Montgomery, Jr., J. E. Peralta, F. Ogliaro, M. Bearpark, J. J. Heyd, E. Brothers, K. N. Kudin, V. N. Staroverov, T. Keith, R. Kobayashi, J. Normand, K. Raghavachari, A. Rendell, J. C. Burant, S. S. Iyengar, J. Tomasi, M. Cossi, N. Rega, J. M. Millam, M. Klene, J. E. Knox, J. B. Cross, V. Bakken, C. Adamo, J. Jaramillo, R. Gomperts, R. E. Stratmann, O. Yazyev, A. J. Austin, R. Cammi, C. Pomelli, J. W. Ochterski, R. L. Martin, K. Morokuma, V. G. Zakrzewski, G. A. Voth, P. Salvador, J. J. Dannenberg, S. Dapprich, A. D. Daniels, Ö. Farkas, J. B. Foresman, J. V. Ortiz, J. Cioslowski and D. J. Fox, Gaussian, Inc., Wallingford CT, 2013.

[13] *Gaussian 16, Revision C.01*, M. J. Frisch, G. W. Trucks, H. B. Schlegel, G. E. Scuseria, M. A. Robb, J. R. Cheeseman, G. Scalmani, V. Barone, G. A. Petersson, H. Nakatsuji, X. Li, M. Caricato, A. V. Marenich, J. Bloino, B. G. Janesko, R. Gomperts, B. Mennucci, H. P. Hratchian, J. V. Ortiz, A. F. Izmaylov, J. L. Sonnenberg, D. Williams-Young, F. Ding, F. Lipparini, F. Egidi, J. Goings, B. Peng, A. Petrone, T. Henderson, D. Ranasinghe, V. G. Zakrzewski, J. Gao, N. Rega, G. Zheng, W. Liang, M. Hada, M. Ehara, K. Toyota, R. Fukuda, J. Hasegawa, M. Ishida, T. Nakajima, Y. Honda, O. Kitao, H. Nakai, T. Vreven, K. Throssell, J. A. Montgomery, Jr., J. E. Peralta, F. Ogliaro, M. J. Bearpark, J. J. Heyd, E. N. Brothers, K. N. Kudin, V. N. Staroverov, T. A. Keith, R. Kobayashi, J. Normand, K. Raghavachari, A. P. Rendell, J. C. Burant, S. S. Iyengar, J. Tomasi, M. Cossi, J. M. Millam, M. Klene, C. Adamo, R. Cammi, J. W. Ochterski, R. L. Martin, K. Morokuma, O. Farkas, J. B. Foresman and D. J. Fox, Gaussian, Inc., Wallingford CT, 2016.

[S14] M. Uejima, T. Sato, D. Yokoyama, K. Tanaka and J.-W. Park, *Phys. Chem. Chem. Phys.*, 2014, **16**, 14244–14256.

[S15] (a) T. Sato, K. Tokunaga and K. Tanaka, *J. Phys. Chem. A*, 2008, **112**, 758–767; (b) T. Kato, N. Haruta and T. Sato, *Vibronic Coupling Density: Understanding Molecular Deformation*, Springer, 2021.

[S16] Examples of positive ΔE_{ST} gaps: (a) V. Bonačić-Koutecký and J. Michl, *J. Am. Chem. Soc.*, 1985, **107**, 1765–1766; (b) T. Sato, M. Uejima, K. Tanaka, H. Kaji and C. Adachi, *J. Mater. Chem. C*, 2015, **3**, 870–878; (c) G. Ricci, E. San-Fabián, Y. Olivier and J. C. Sancho-García, *ChemPhysChem*, 2021, **22**, 553–560; (d) N. Aizawa, Y.-J. Pu, Y. Harabuchi, A. Nihonyanagi, R. Ibuka, H. Inuzuka, B. Dhara, Y. Koyama, K. Nakayama, S. Maeda, F. Araoka and D. Miyajima, *Nature*, 2022, **609**, 502–506.

[S17] Articles for *GAMESS*: (a) M. W. Schmidt, K. K., Baldridge, J. A. Boatz, S. T. Elbert, M. S. Gordon, J. H. Jensen, S. Koseki, N. Matsunaga, K. A. Nguyen, S. J. Su, T. L. Windus, M. Dupuis and J. A. Montgomery, *J. Comput. Chem.*, 1993, **14**, 1347−1363; (b) M. S. Gordon and M. W. Schmidt, in *Theory and Applications of Computational Chemistry: the first forty years*, eds. C. E. Dykstra, G. Frenking, K. S. Kim and G. E. Scuseria, Elsevier, 2005, pp.1167–1189; (c) G. M. J. Barca, C. Bertoni, L. Carrington, D. Datta, N. De Silva, J. E. Deustua, D. G. Fedorov, J. R. Gour, A. O. Gunina, E. Guidez, T. Harville, S. Irle, J. Ivanic, K. Kowalski, S. S. Leang, H. Li, W. Li, J. J. Lutz, I. Magoulas, J. Mato, V. Mironov, H. Nakata, B. Q. Pham, P. Piecuch, D. Poole, S. R. Pruitt, A. P. Rendell, L. B. Roskop, K. Ruedenberg, T. Sattasathuchana, M. W. Schmidt, J. Shen, L. Slipchenko, M. Sosonkina, V. Sundriyal, A. Tiwari, J. L. Galvez Vallejo, B. Westheimer, M. Włoch, P. Xu, F. Zahariev and M. S. Gordon, *J. Chem. Phys.*, 2020, **152**, 154102.

[S18] Report for FMO: K. Kitaura, E. Ikeo, T. Asada, T. Nakano and M. Uebayasi, *Chem. Phys. Lett.*, 1999, **313**, 701–706.

[S19] Report for pair interaction energy decomposition analysis (PIEDA): D. G. Fedorov and K. Kitaura, *J. Comput. Chem.*, 2007, **28**, 222–237.

4. Anion-binding behaviors

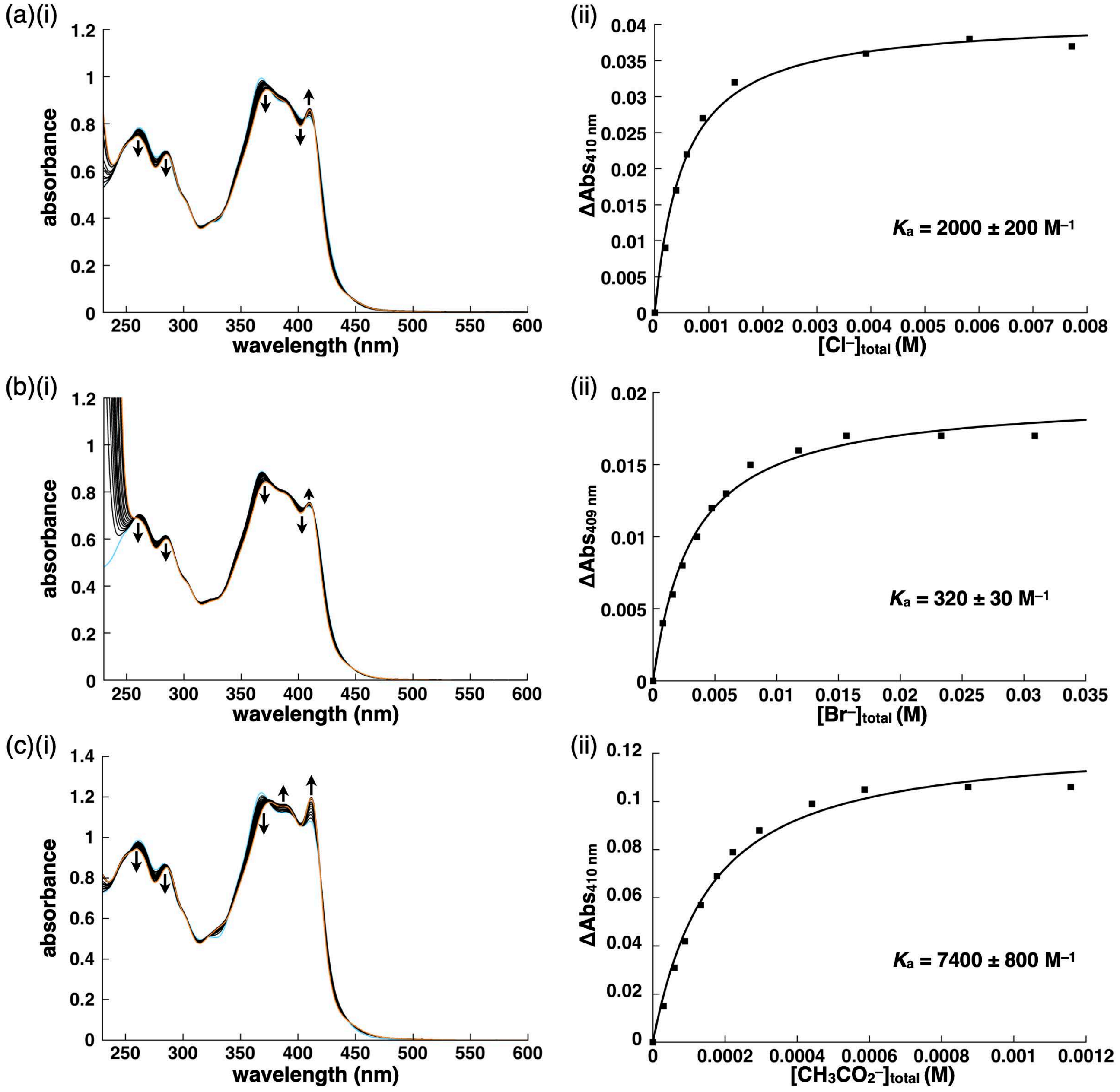

Fig. S70 (i) UV/vis absorption spectral changes and (ii) titration plots and 1:1 fitting curves of **2b** (0.03 mM) upon the addition of (a) Cl^-, (b) Br^-, and (c) $CH_3CO_2^-$ as tetrabutylammonium (TBA) salts in CH_2Cl_2. Cyan and orange lines in (i) represent the absorptions at the initial and final states, respectively.

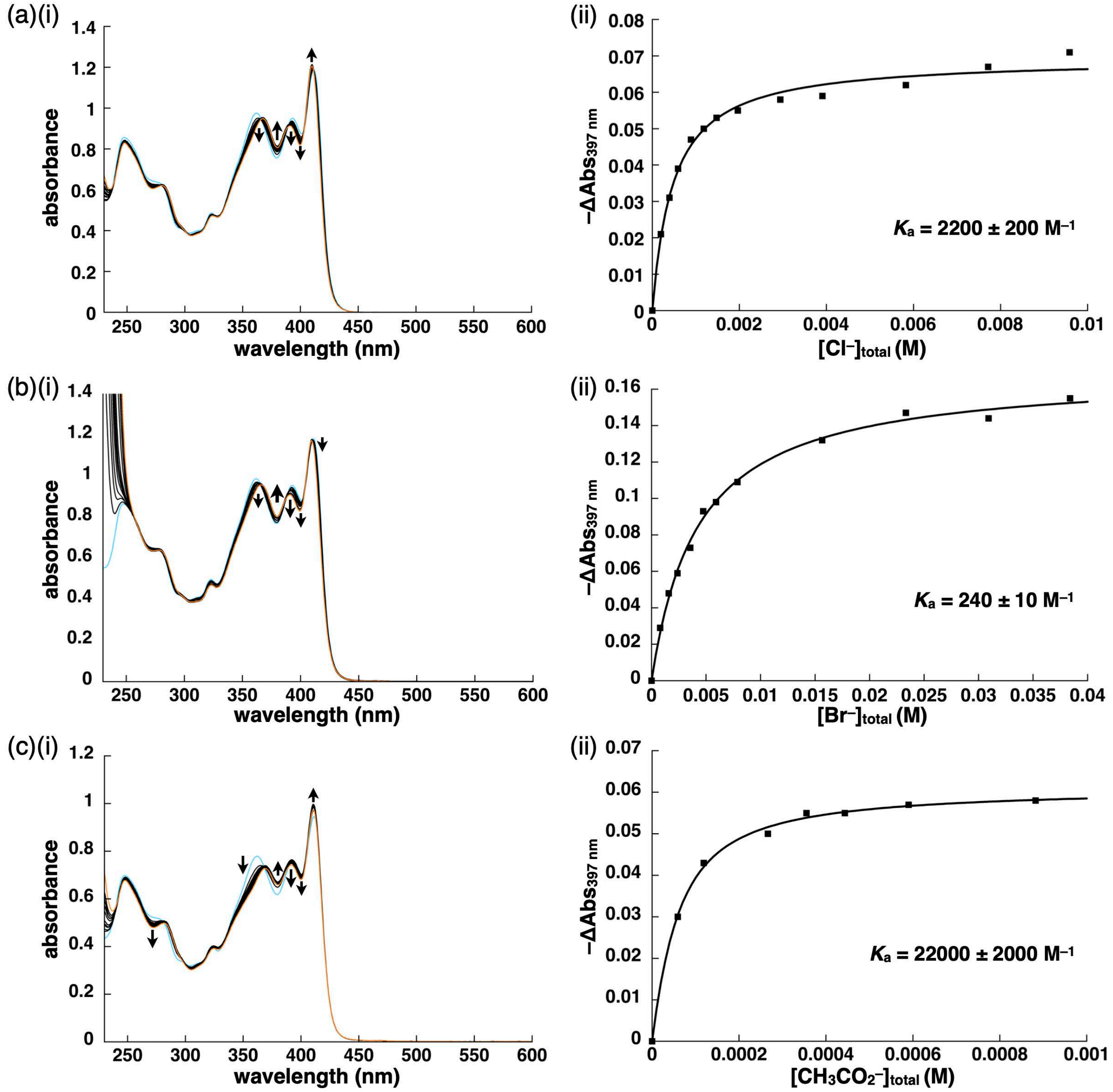

Fig. S71 (i) UV/vis absorption spectral changes and (ii) titration plots and 1:1 fitting curves of **2c** (0.03 mM) upon the addition of (a) Cl^-, (b) Br^-, and (c) $CH_3CO_2^-$ as TBA salts in CH_2Cl_2. Cyan and orange lines in (i) represent the absorptions at the initial and final states, respectively.

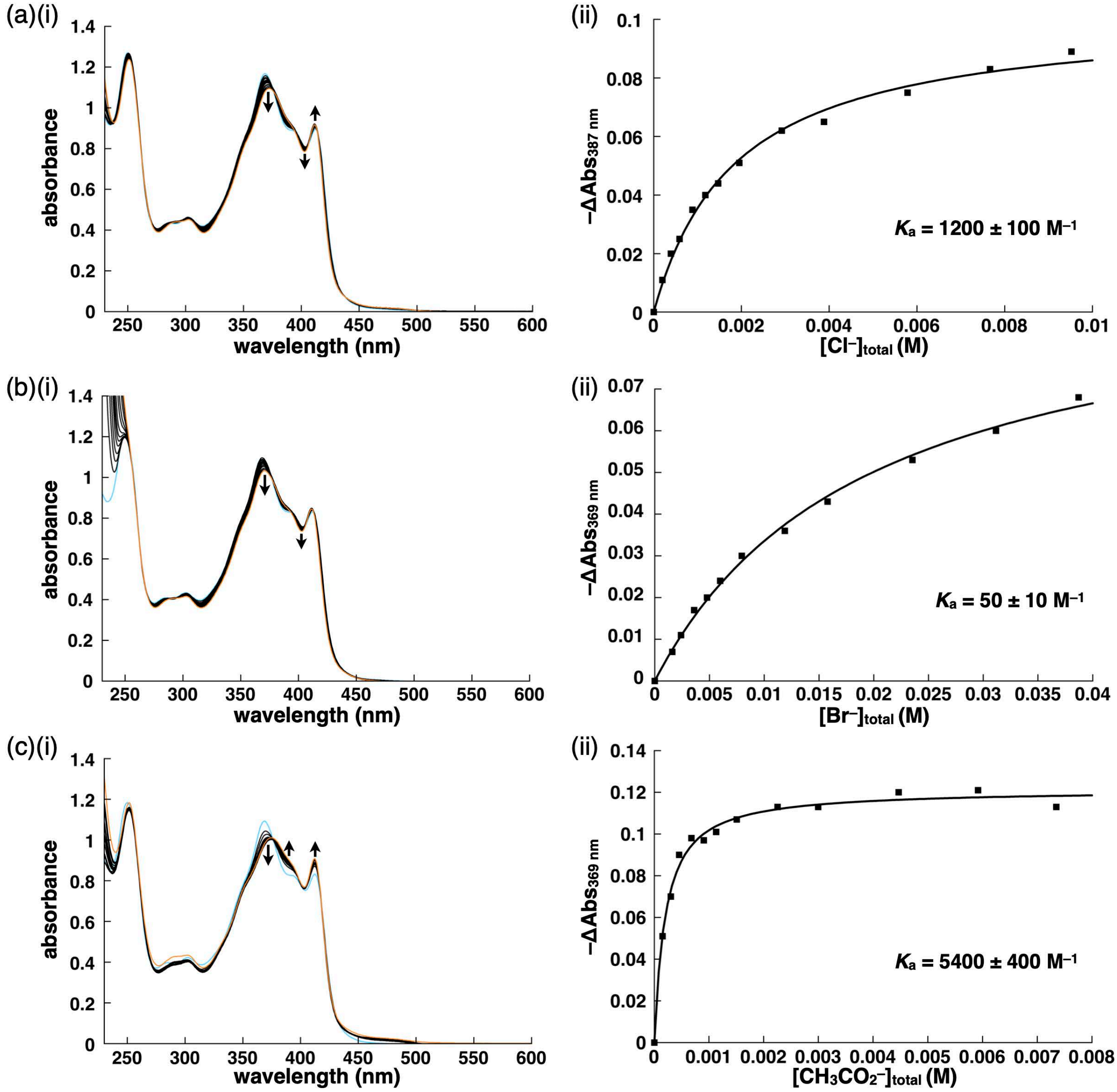

Fig. S72 (i) UV/vis absorption spectral changes and (ii) titration plots and 1:1 fitting curves of **2d** (0.03 mM) upon the addition of (a) Cl^-, (b) Br^-, and (c) $CH_3CO_2^-$ as TBA salts in CH_2Cl_2. Cyan and orange lines in (i) represent the absorptions at the initial and final states, respectively.

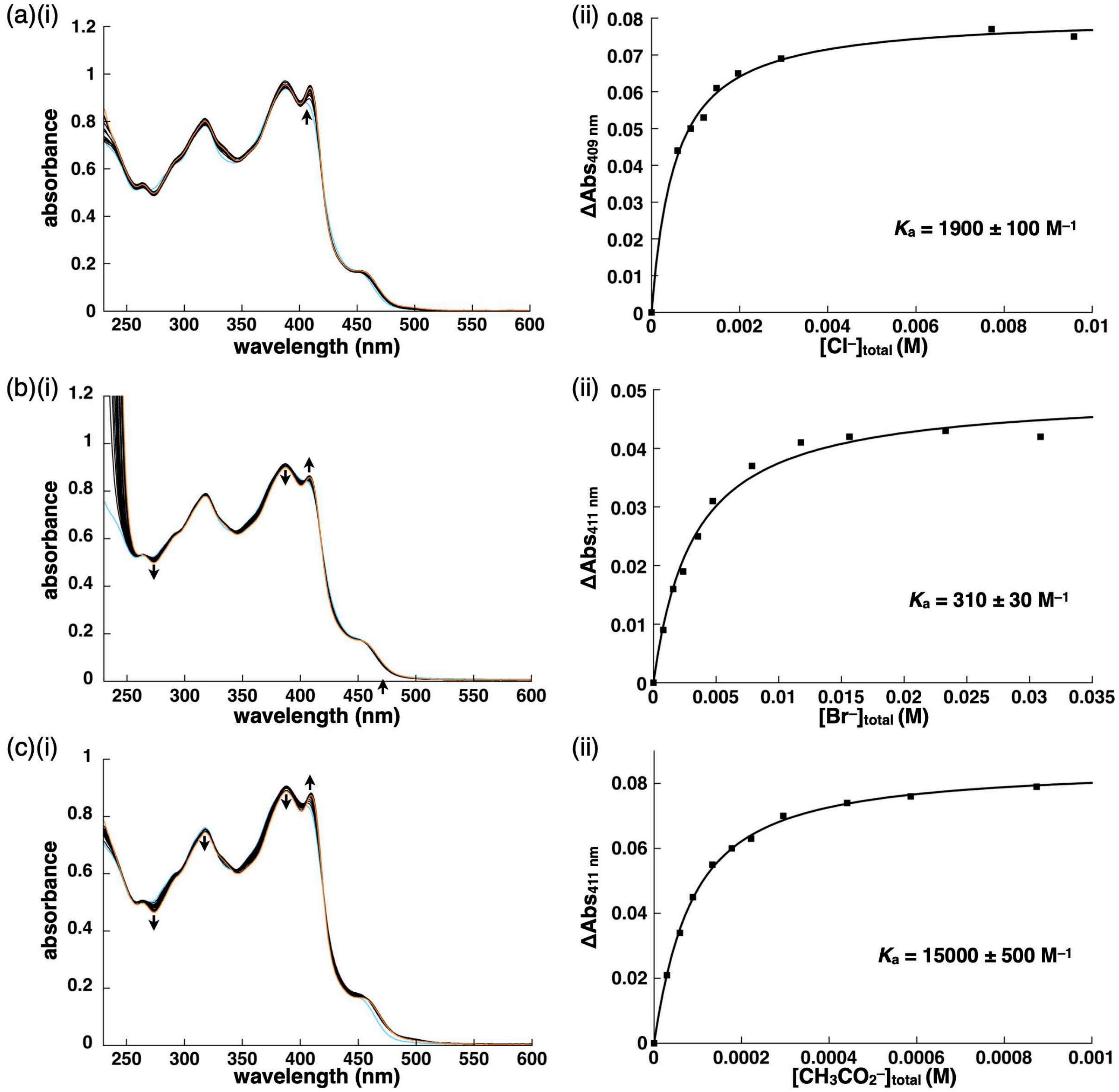

Fig. S73 (i) UV/vis absorption spectral changes and (ii) titration plots and 1:1 fitting curves of **2e** (0.03 mM) upon the addition of (a) Cl^-, (b) Br^-, and (c) $CH_3CO_2^-$ as TBA salts in CH_2Cl_2. Cyan and orange lines in (i) represent the absorptions at the initial and final states, respectively.

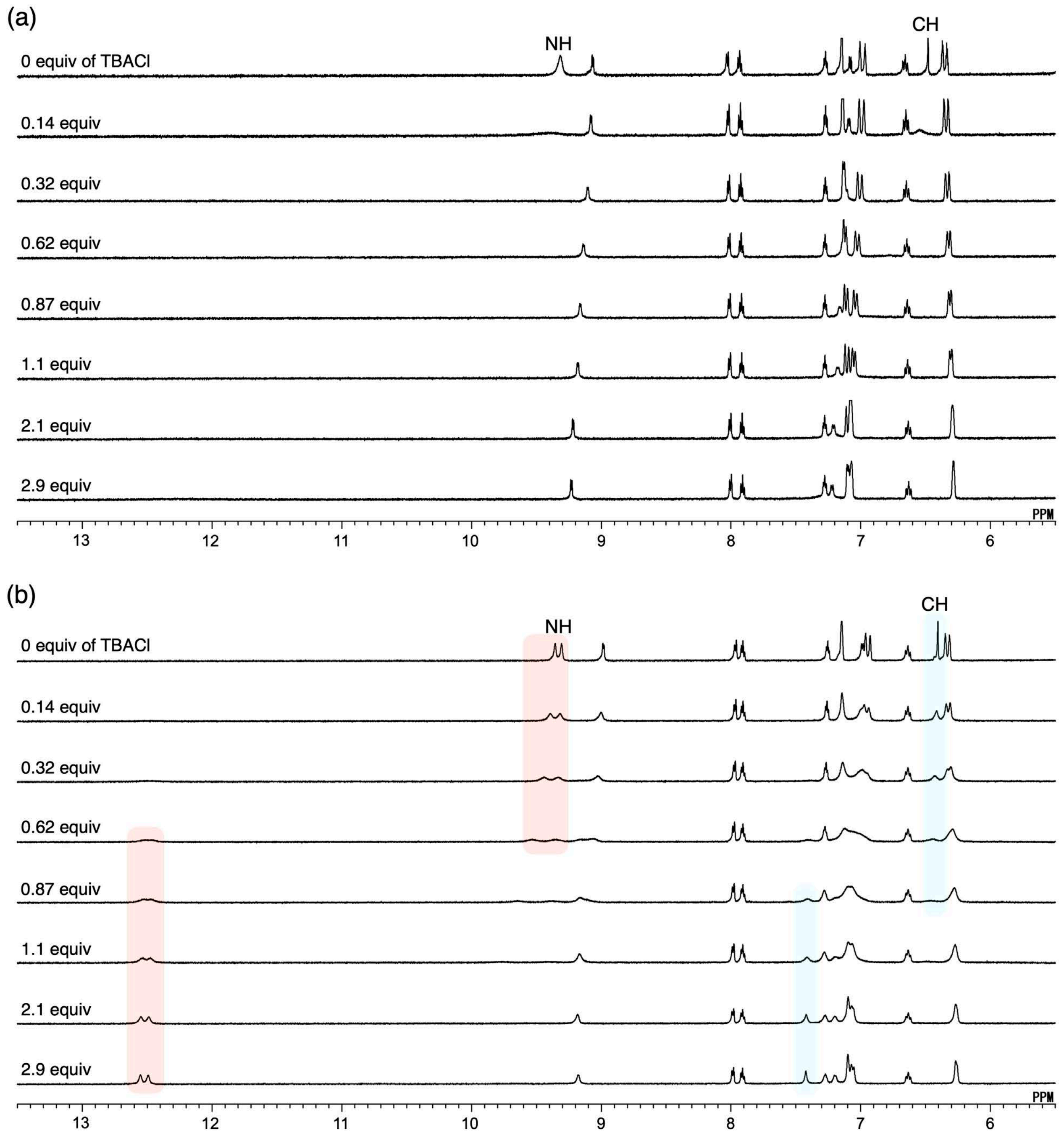

Fig. S74 ^{1}H NMR spectral changes of **2b** (1.0×10^{-3} M) upon the addition of Cl^- (0–3.0 equiv) added as a TBA salt in CD_2Cl_2 at (a) 20 °C and (b) –50 °C. As addition of anions, the signals of pyrrole NH (9.39 and 9.42 ppm) and bridging CH (6.45 ppm) were decreased and new signals were emerged in the downfield regions, whose signals were identified as anion-binding pyrrole NH (12.47 and 12.53 ppm) and bridging CH (7.37 ppm) upon the addition of 3.0 equiv of TBACl at –50 °C.

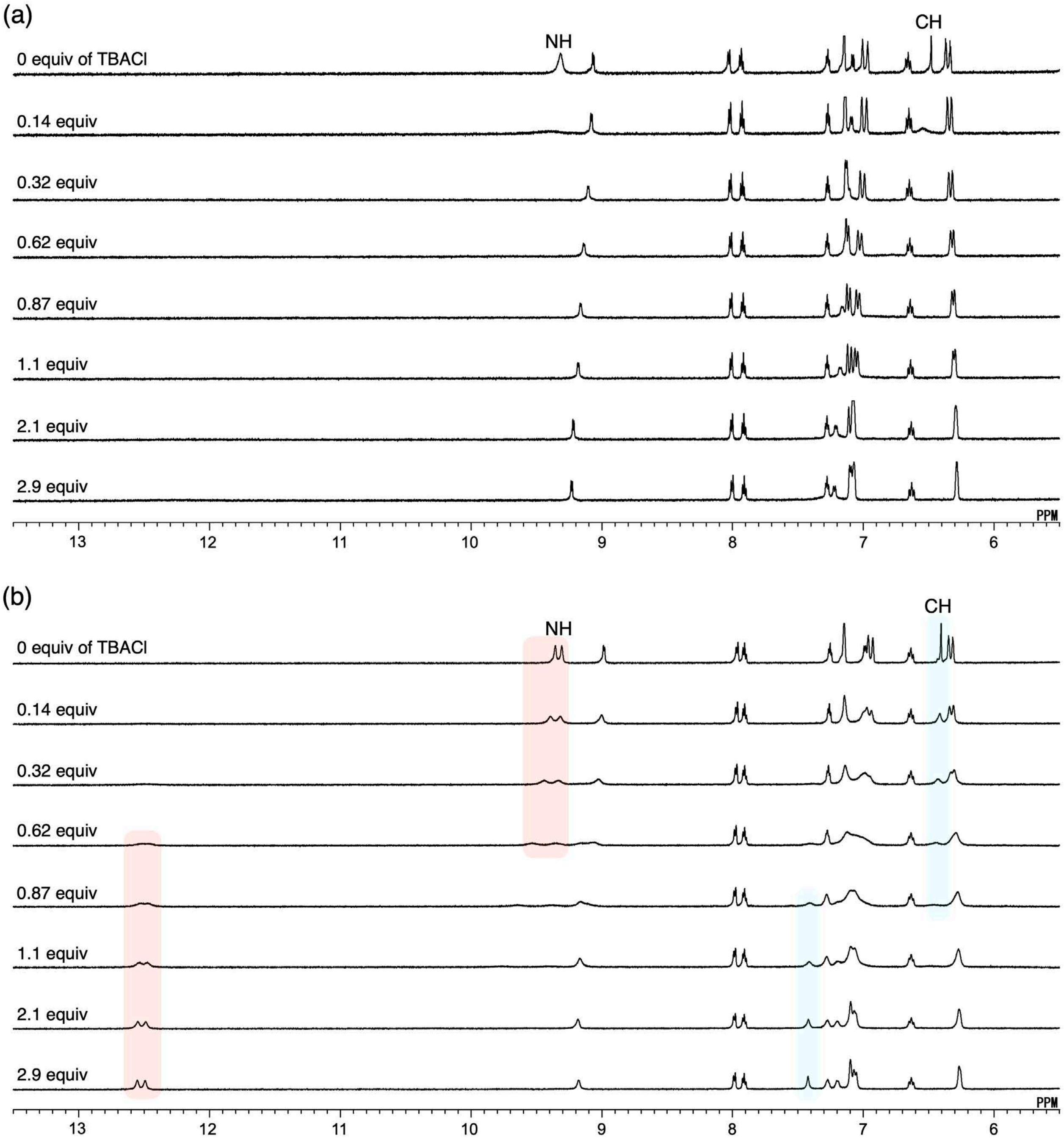

Fig. S75 ^{1}H NMR spectral changes of **2c** (1.0×10^{-3} M) upon the addition of Cl^- (0–2.9 equiv) added as a TBA salt in CD_2Cl_2 at (a) 20 °C and (b) –50 °C. As addition of anions, the signals of pyrrole NH (9.31 and 9.36 ppm) and bridging CH (6.41 ppm) were decreased and new signals were emerged in the downfield regions, whose signals were identified as anion-binding pyrrole NH (12.49 and 12.55 ppm) and bridging CH (7.42 ppm) upon the addition of 2.9 equiv of TBACl at –50 °C.

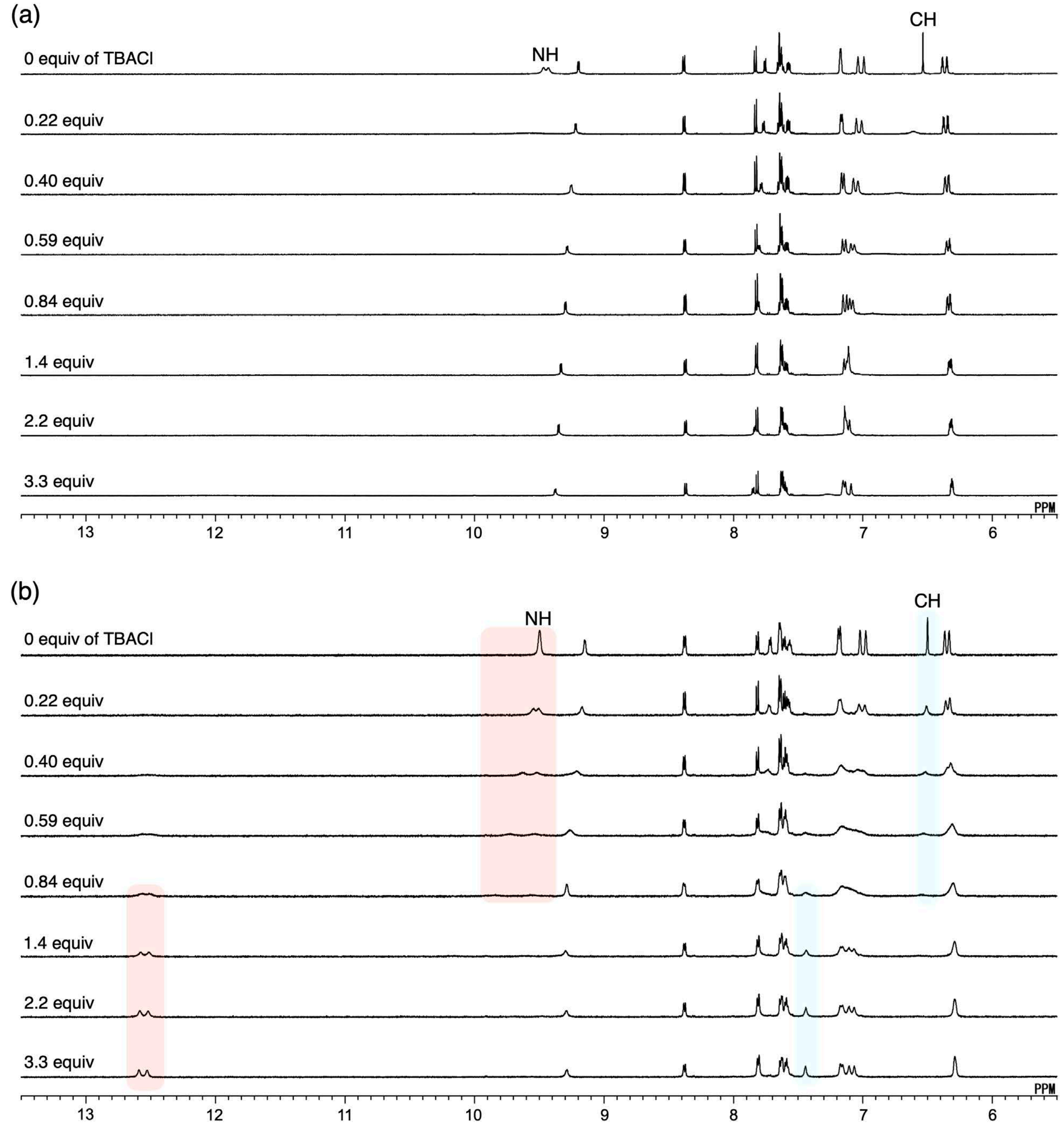

Fig. S76 ^{1}H NMR spectral changes of **2d** (1.0×10^{-3} M) upon the addition of Cl^- (0–3.3 equiv) added as a TBA salt in CD_2Cl_2 at (a) 20 °C and (b) –50 °C. By the addition of anions, the signals of pyrrole NH (9.49 ppm) and bridging CH (6.49 ppm) were decreased and new signals were emerged in the downfield regions, whose signals were identified as anion-binding pyrrole NH (12.53 and 12.59 ppm) and bridging CH (7.45 ppm) upon the addition of 3.3 equiv of TBACl at –50 °C.

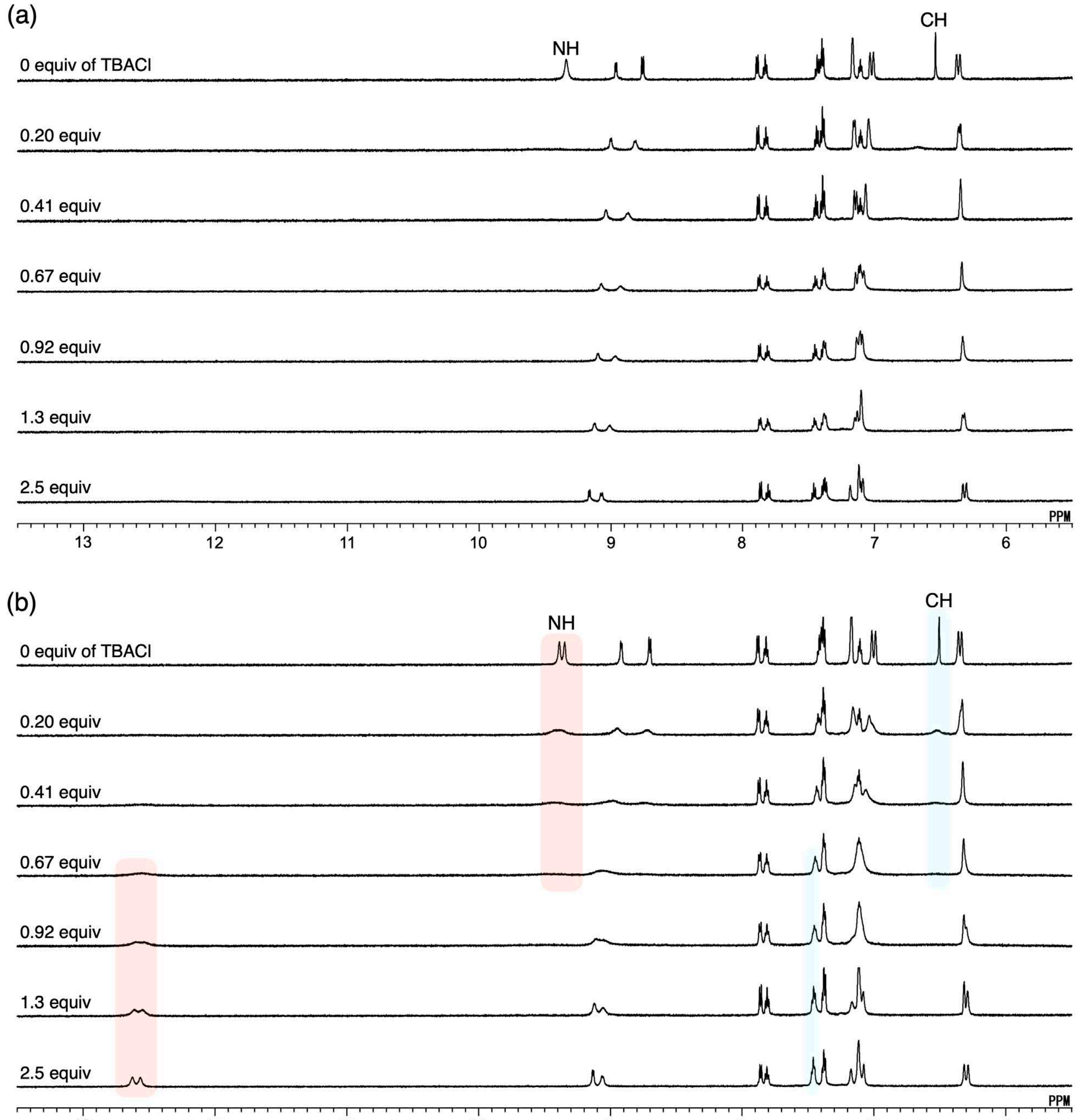

Fig. S77 ^{1}H NMR spectral changes of **2e** (1.0×10^{-3} M) upon the addition of Cl^- (0–2.5 equiv) added as a TBA salt in CD_2Cl_2 at (a) 20 °C and (b) –50 °C. As addition of anions, the signals of pyrrole NH (9.35 and 9.39 ppm) and bridging CH (6.51 ppm) were decreased and new signals were emerged in the downfield regions, whose signals were identified as anion-binding pyrrole NH (12.57 and 12.63 ppm) and bridging CH (7.45 ppm) upon the addition of 2.5 equiv of TBACl at –50 °C.

5. Solution-state excited-state properties

Method for Emission Spectra, Quantum Yields, and Emission Lifetimes. Emission spectra and quantum yields were recorded on a Hitachi F-4500 fluorescence spectrometer and a Hamamatsu Quantum Yields Measurements System for Organic LED Materials C9920-02, respectively. Emission lifetimes were measured using a C7990S system (Hamamatsu Photonics) equipped with a 403-nm excitation laser, producing 62-ps pulses with a repetition rate of 100 kHz.

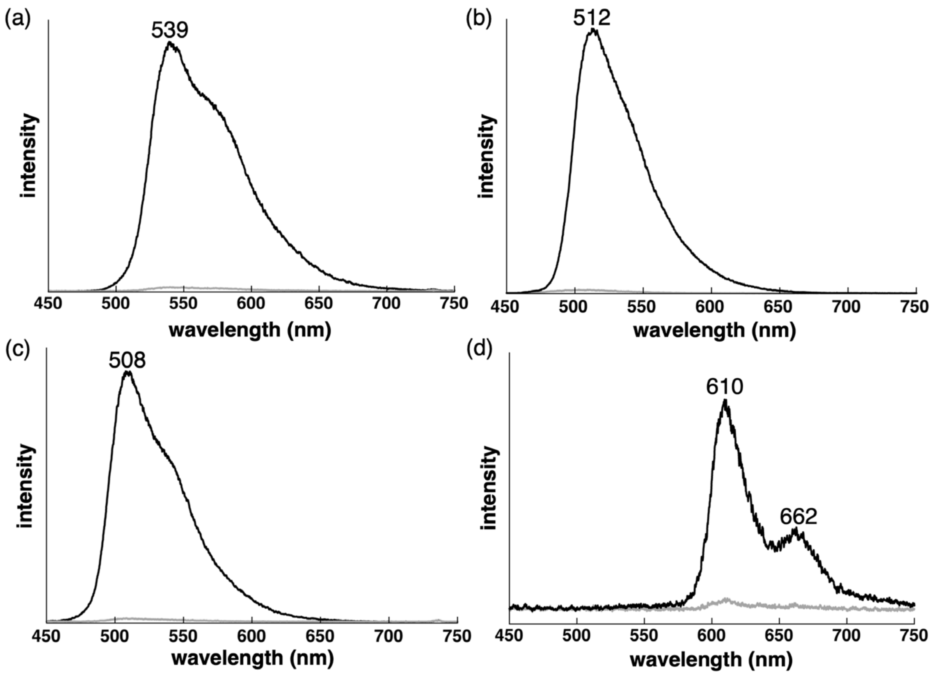

Fig. S78 Emission spectra of (a) **2b** (λ_{ex} = 368 nm), (b) **2c** (λ_{ex} = 411 nm), (c) **2d** (λ_{ex} = 368 nm), and (d) **2e** (λ_{ex} = 388 nm) in deoxygenated CH_2Cl_2 (black) and non-deoxygenated CH_2Cl_2 (gray) (3 μM for each) at r.t. Solution-state emission quantum yields for the Pt^{II} complexes are summarized in Table S6.

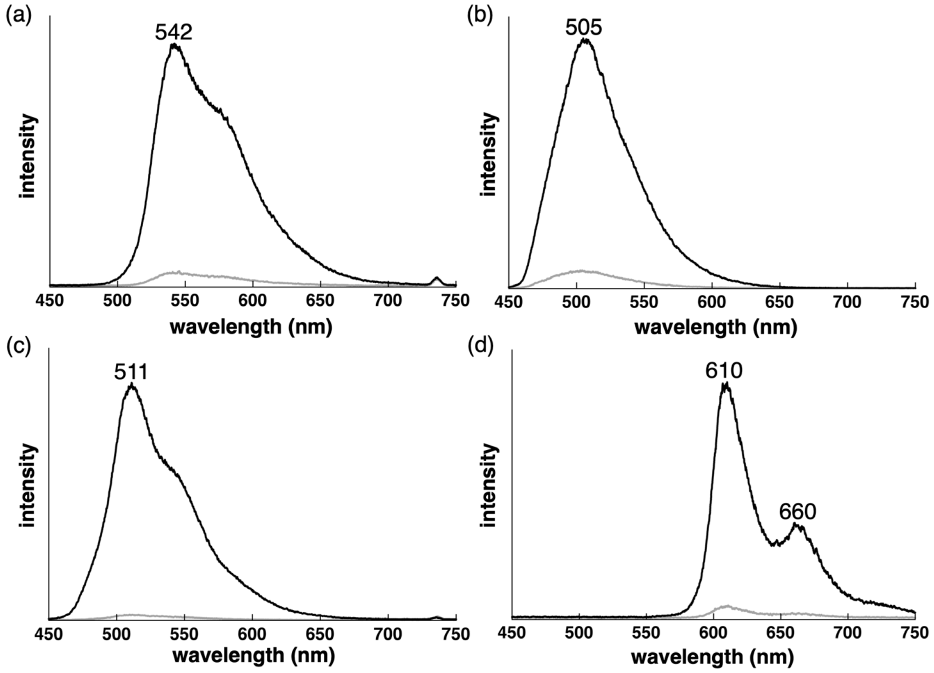

Fig. S79 Emission spectra of (a) **2b** (λ_{ex} = 368 nm), (b) **2c** (λ_{ex} = 411 nm), (c) **2d** (λ_{ex} = 368 nm), and (d) **2e** (λ_{ex} = 388 nm) upon the addition of 2000 (**2b**,**c**,**e**) and 3000 (**2d**) equiv of Cl^- as a TBA salt in deoxygenated CH_2Cl_2 (black) and non-deoxygenated CH_2Cl_2 (gray) (3 μM for each) at r.t. Solution-state emission quantum yields for the Pt^{II} complexes are summarized in Table S6.

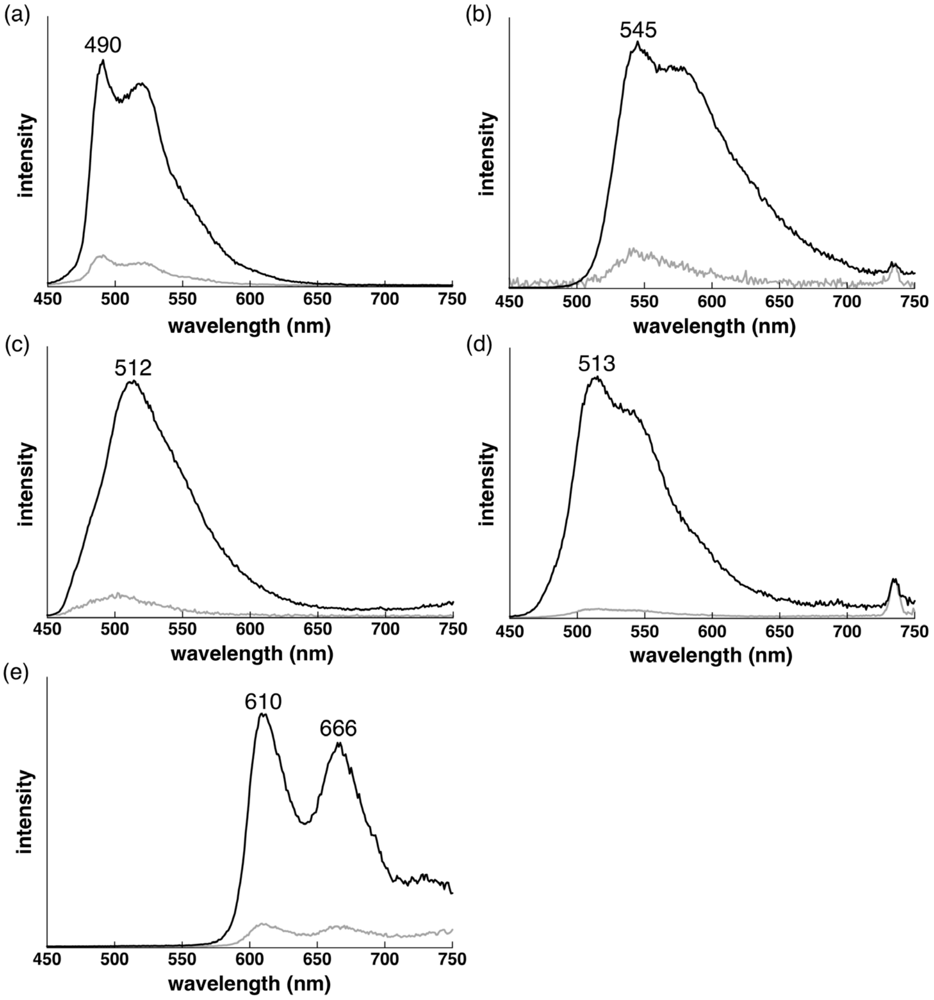

Fig. S80 Emission spectra of (a) **2a** (λ_{ex} = 410 nm), (b) **2b** (λ_{ex} = 368 nm), (c) **2c** (λ_{ex} = 411 nm), (d) **2d** (λ_{ex} = 368 nm), and (e) **2e** (λ_{ex} = 388 nm) upon the addition of 10000 (**2a**–**c**,**e**) and 12000 (**2d**) equiv of Br^{-} as a TBA salt in deoxygenated CH_2Cl_2 (black) and non-deoxygenated CH_2Cl_2 (gray) (3 μM for each) at r.t. Solution-state emission quantum yields for the Pt^{II} complexes are summarized in Table S6.

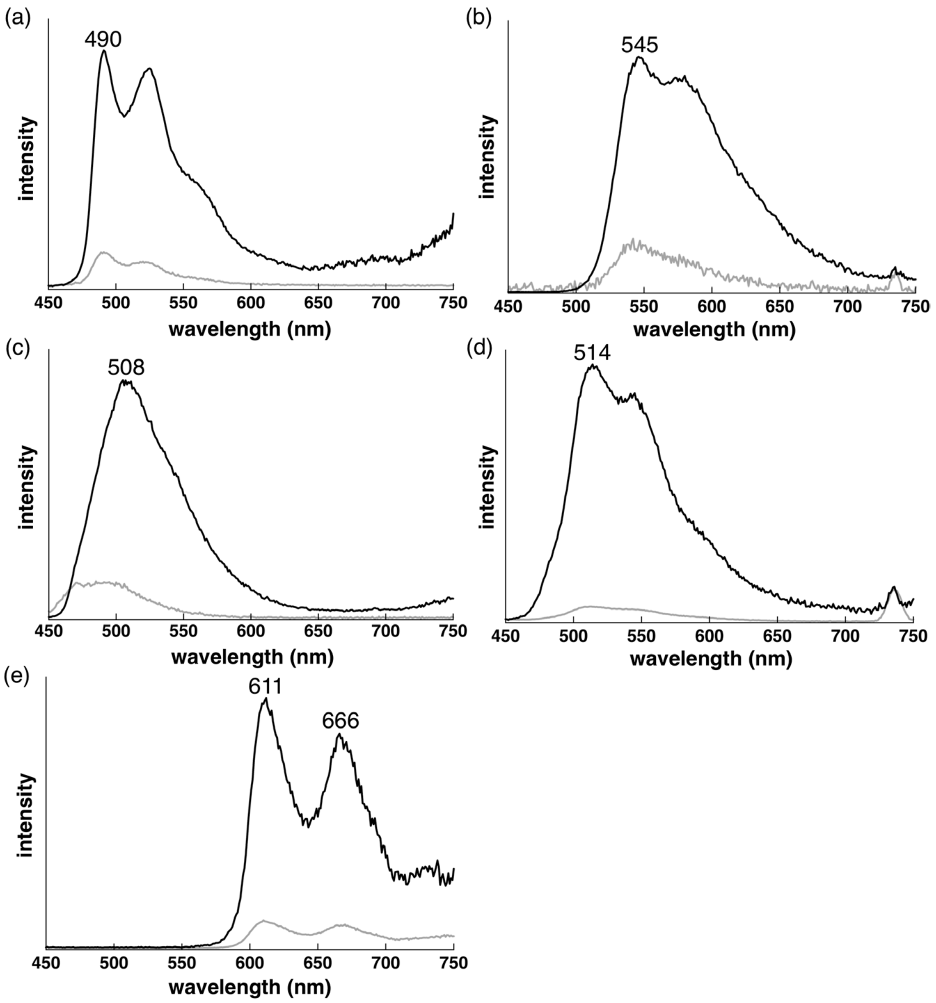

Fig. S81 Emission spectra of (a) **2a** (λ_{ex} = 410 nm), (b) **2b** (λ_{ex} = 368 nm), (c) **2c** (λ_{ex} = 411 nm), (d) **2d** (λ_{ex} = 368 nm), and (e) **2e** (λ_{ex} = 388 nm) upon the addition of 10000 (**2a**–**c**,**e**) and 12000 (**2d**) equiv of $CH_3CO_2^-$ as a TBA salt in deoxygenated CH_2Cl_2 (black) and non-deoxygenated CH_2Cl_2 (gray) (3 μM for each) at r.t. Solution-state emission quantum yields for the Pt^{II} complexes are summarized in Table S6.

Table S6 Emission quantum yields of **2a**–**e** with Cl^-, Br^-, and $CH_3CO_2^-$ complexes in deoxygenated and non-deoxygenated CH_2Cl_2 at 20 °C.

solution conditions	**2a**	**2b**	**2c**	**2d**	**2e**
deoxygenated CH_2Cl_2	0.420[a]	0.675	0.495	0.503	0.161
non-deoxygenated CH_2Cl_2	0.017[a]	0.013	0.007	0.004	0.007
solution conditions	**2a**·Cl^-	**2b**·Cl^-	**2c**·Cl^-	**2d**·Cl^-	**2e**·Cl^-
deoxygenated CH_2Cl_2	0.475[a]	0.537	0.320	0.412	0.142
non-deoxygenated CH_2Cl_2	0.071[a]	0.015	0.011	0.033	0.009
solution conditions	**2a**·Br^-	**2b**·Br^-	**2c**·Br^-	**2d**·Br^-	**2e**·Br^-
deoxygenated CH_2Cl_2	0.349	0.629	0.325	0.197	0.144
non-deoxygenated CH_2Cl_2	0.045	0.024	0.018	0.008	0.014
solution conditions	**2a**·$CH_3CO_2^-$	**2b**·$CH_3CO_2^-$	**2c**·$CH_3CO_2^-$	**2d**·$CH_3CO_2^-$	**2e**·$CH_3CO_2^-$
deoxygenated CH_2Cl_2	0.441	0.650	0.308	0.260	0.146
non-deoxygenated CH_2Cl_2	0.037	0.027	0.017	0.010	0.012

[a] Ref [S1].

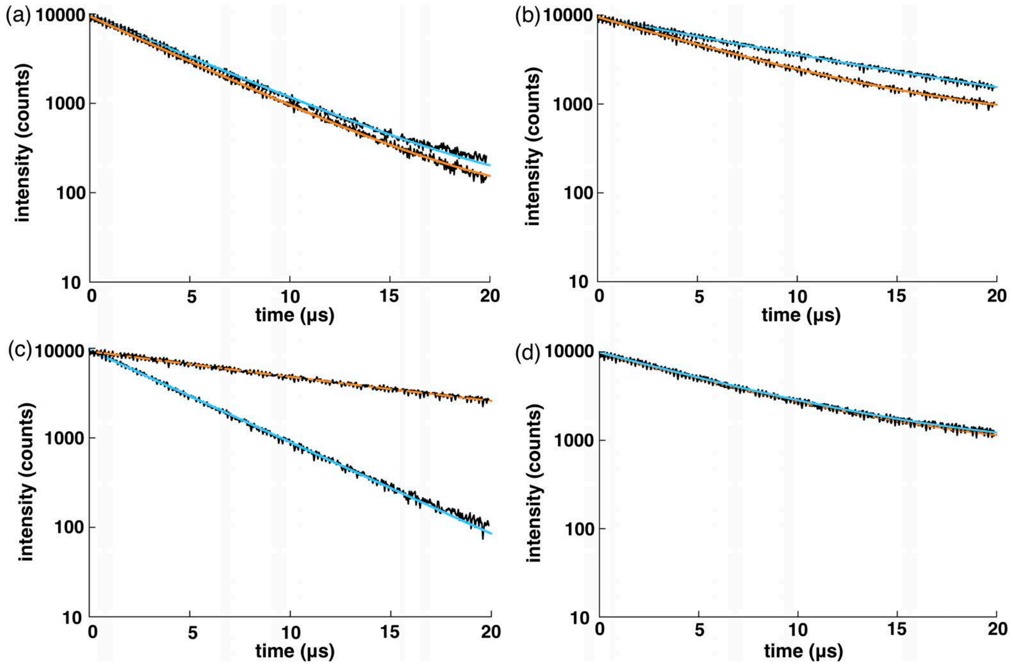

Fig. S82 Emission decay profiles of (a) **2b**, (b) **2c**, (c) **2d**, and (d) **2e** (3 μM for each) monitored at 539, 512, 508, and 662 nm, respectively, in the absence (cyan) of Cl^- and 542, 505, 511, and 660 nm, respectively, in the presence (orange) of Cl^- as a TBA salt (2000 equiv for **2b**,**c**,**e** and 3000 equiv for **2d**) in the ranges from 0 ns to 20 μs in deoxygenated CH_2Cl_2 excited at 403 nm at 20 °C. The emission decay profiles for **2b** and **2b**·Cl^- were fitted with the single exponential decay function, providing the emission lifetimes (τ), which were 4.6 and 4.3 μs, respectively. Similarly, those for **2c**/**2c**·Cl^-, **2d**/**2d**·Cl^-, and **2e**/**2e**·Cl^- provided the τ values of 9.6/6.4, 4.2/14.2, and 6.8/6.8 μs, respectively.

6. Solid-state properties

Elemental Analysis. Composition for ion-pairing assemblies prepared by precipitation were analyzed using a Series II CHNS/O Analyzer 2400 (PerkinElmer).

Synchrotron X-ray Diffraction Analysis (XRD). High-resolution XRD analysis was carried out using a synchrotron radiation X-ray beam with the wavelengths of 1.00 Å on BL40B2 at SPring-8 (Hyogo, Japan). A DECTRIS PILATUS3 S 2M with camera with camera lengths of 429.5 mm for powder samples of ion-pairing assemblies. Powder samples were enclosed in quartz capillary for the analysis. The broad peak derived from the capillary was not subtracted from the data.

Method for Solid-State Emission Spectra. Measurements of steady-state emission and excitation spectra were performed using a FP-8500 spectrofluorometer (JASCO). Phosphorescence quantum efficiencies were performed using a FP-8500 spectrofluorometer (JASCO) with an ISF-834 fluorescence integrate sphere unit (JASCO). The excitation wavelength was 365 nm. Phosphorescence lifetime measurements for the crystals were performed using a TemPro Fluorescence Lifetime System (Horiba Jobin Yvon) equipped with an LED excitation source of 352 nm with a pulse-duration full width at half maximum (FWHM) of approximately 1 ns. For measurements, single crystals were lightly crushed with a spatula on a quartz plate, and the crystals showed no significant changes in the XRD patterns.

Table S7 Summary of elemental analyses (C, H, and N) for the precipitates of **2a**·Cl^--TPA^+, **2b**·Cl^--TPA^+, **2d**·Cl^--TPA^+, **2d**·Cl^--$TPeA^+$, **2e**·Cl^--TPA^+, **2e**·Cl^--TBA^+, and **2e**·Cl^--$TPeA^+$.

samples	formula	C (% found/calculated)	H (% found/calculated)	N (% found/calculated)
**2a**·Cl^--TPA^+	$C_{22}H_{17}N_3O_2Pt\cdot C_{12}H_{28}N\cdot Cl\cdot 0.1C_6H_{14}\cdot 1.3H_2O$	51.54/51.67	6.40/6.14	7.11/6.97
**2b**·Cl^--TPA^+	$C_{23}H_{19}N_3O_3Pt\cdot C_{12}H_{28}N\cdot Cl\cdot 0.9H_2O$	51.63/51.36	6.31/6.01	6.73/6.84
**2d**·Cl^--TPA^+	$C_{24}H_{17}N_3O_2Pt\cdot C_{12}H_{28}N\cdot Cl\cdot 0.9H_2O$	53.56/53.22	6.04/5.81	7.08/6.90
**2d**·Cl^--$TPeA^+$	$C_{24}H_{17}N_3O_2Pt\cdot C_{20}H_{44}N\cdot Cl\cdot 0.1H_2O$	58.22/58.05	7.05/6.78	6.09/6.15
**2e**·Cl^--TPA^+	$C_{24}H_{17}N_3O_2Pt\cdot C_{12}H_{28}N\cdot Cl\cdot 1.0H_2O$	51.07/51.09	5.74/5.60	6.47/6.62
**2e**·Cl^--TBA^+	$C_{24}H_{17}N_3O_2PtS\cdot C_{16}H_{36}N\cdot Cl\cdot 0.6H_2O$	53.72/53.66	6.39/6.10	6.35/6.26
**2e**·Cl^--$TPeA^+$	$C_{24}H_{17}N_3O_2PtS\cdot C_{20}H_{44}N\cdot Cl\cdot 0.1C_6H_{14}\cdot 0.5H_2O$	55.93/55.9	6.94/6.67	5.82/5.85

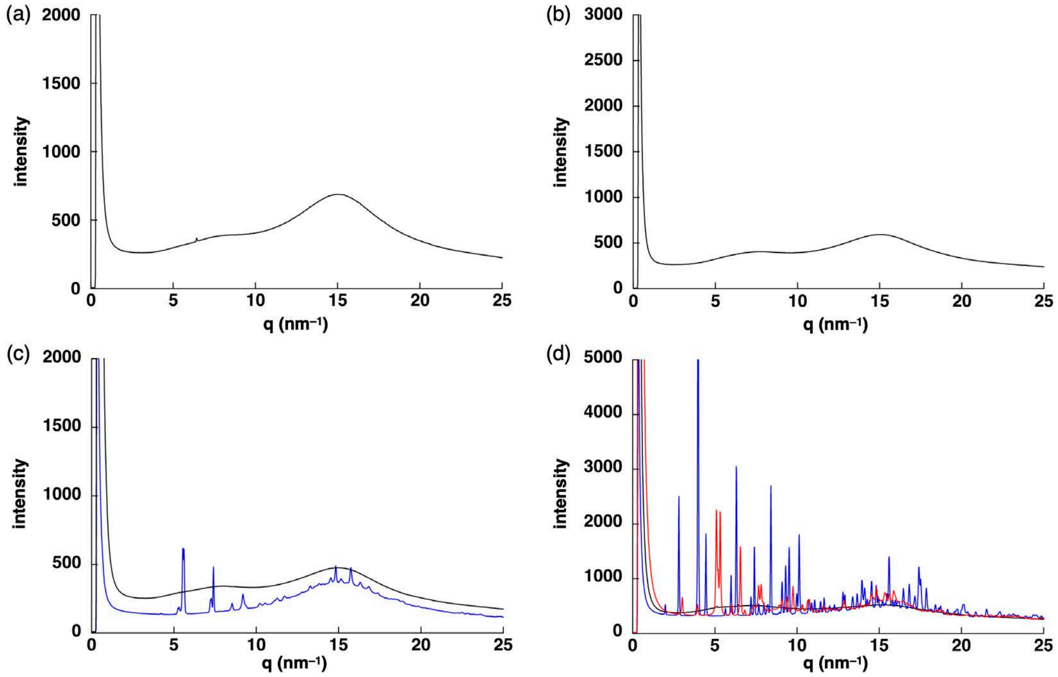

Fig. S83 Synchrotron XRD patterns of (a) **2a**·Cl⁻-TPA⁺, (b) **2b**·Cl⁻-TPA⁺, (c) **2d**·Cl⁻-TPA⁺ (black) and **2d**·Cl⁻-TPeA⁺ (blue), and (d) **2e**·Cl⁻-TPA⁺ (black), **2e**·Cl⁻-TBA⁺ (blue), and **2e**·Cl⁻-TPeA⁺ (red) as precipitates. Less clear diffraction patterns with broad peaks indicate the less ordered arrangement of anion complex and countercations.

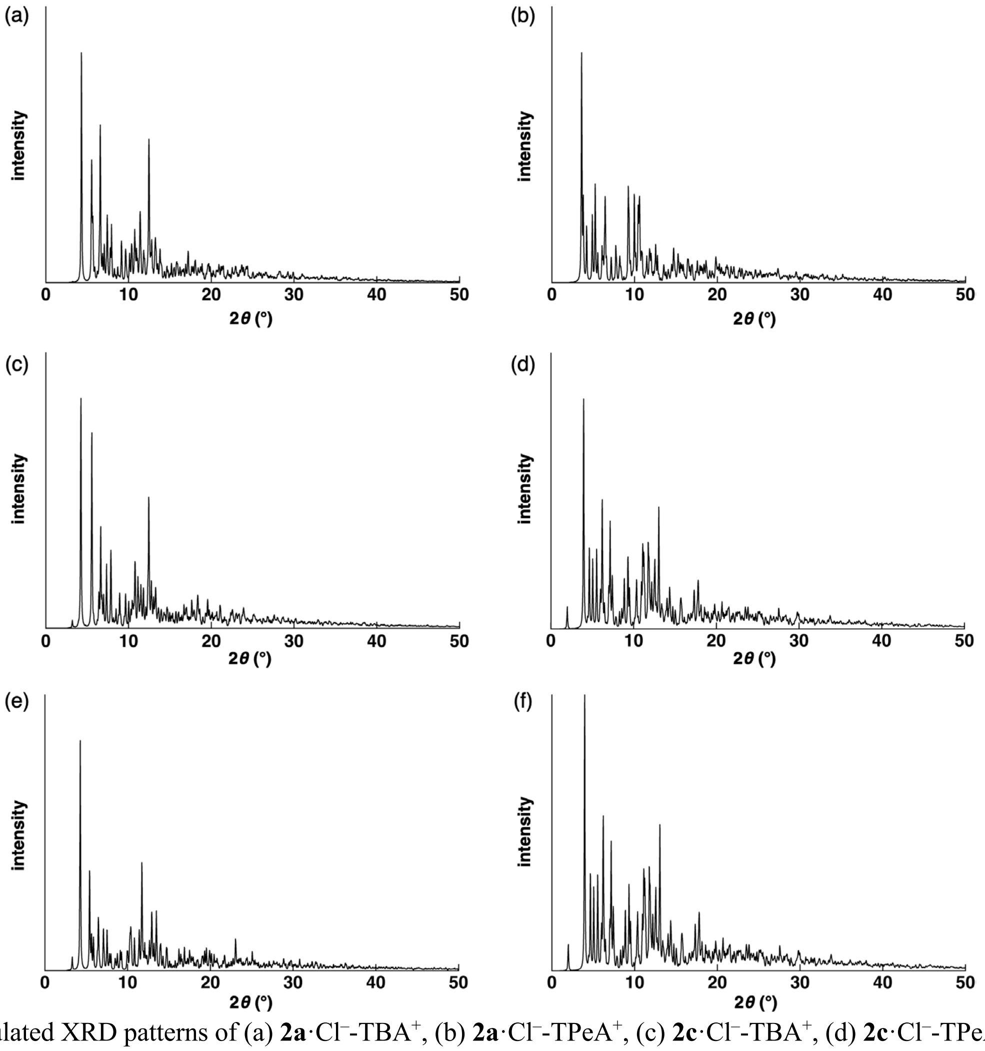

Fig. S84 Simulated XRD patterns of (a) **2a**·Cl⁻-TBA⁺, (b) **2a**·Cl⁻-TPeA⁺, (c) **2c**·Cl⁻-TBA⁺, (d) **2c**·Cl⁻-TPeA⁺, (e) **2d**·Cl⁻-TBA$^{+}_{M}$, and (f) **2d**·Cl⁻-TBA$^{+}_{E}$ as single crystals.

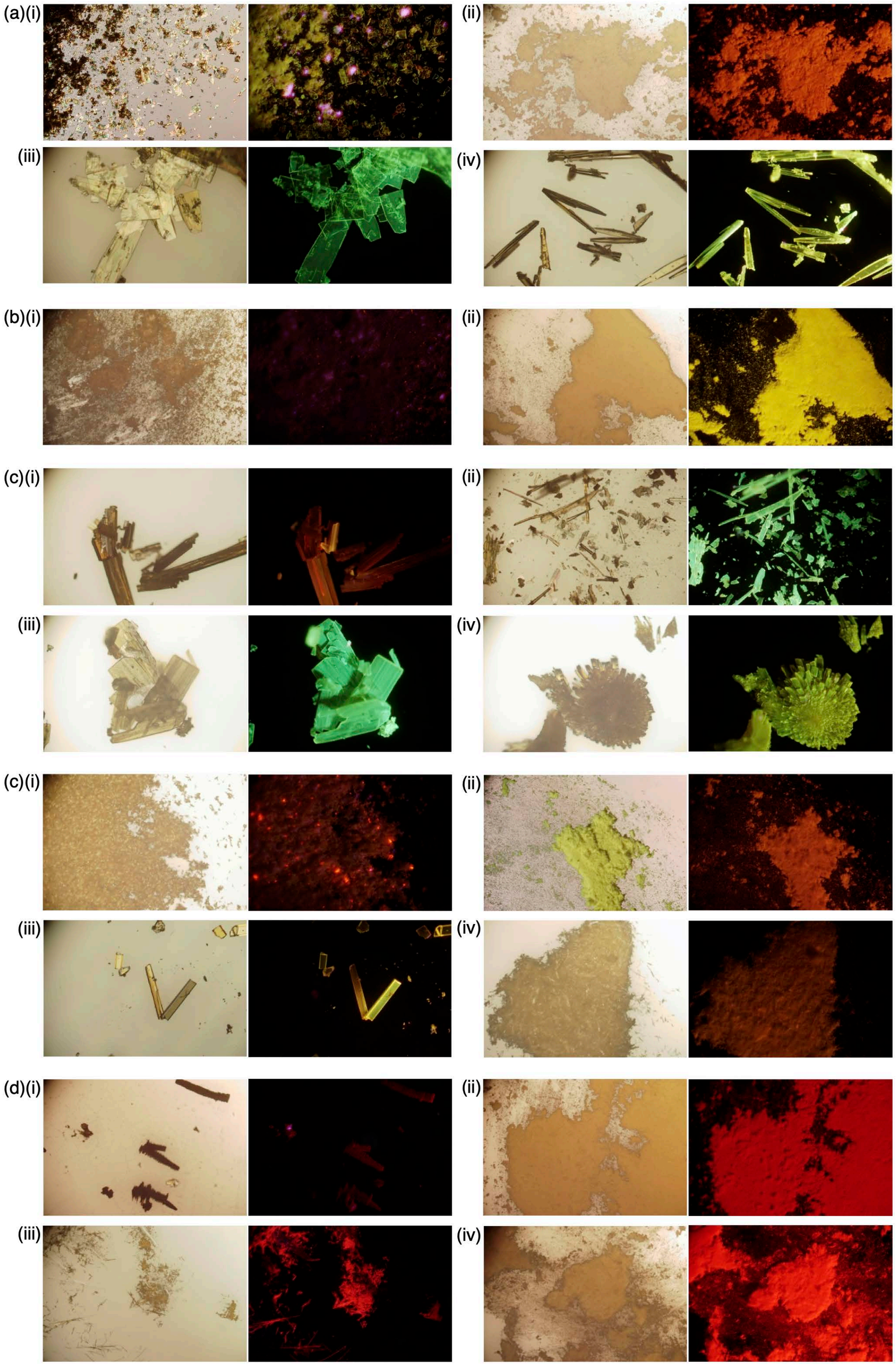

Fig. S85 Photographs of Pt^{II} complexes and their ion-pairing assemblies (anion complexes) in the solid state under visible (left) and $UV_{365\ nm}$ (right) light: (a)(i) **2a**, (ii) **2a**·Cl^--TPA^+, (iii) **2a**·Cl^--TBA^+, and (iv) **2a**·Cl^--$TPeA^+$, (b)(i) **2b** and (ii) **2b**·Cl^--TPA^+, (c)(i) **2c**, (ii) **2c**·Cl^--TPA^+, (iii) **2c**·Cl^--TBA^+, and (iv) **2c**·Cl^--$TPeA^+$, (d)(i) **2d**, (ii) **2d**·Cl^--TPA^+, (iii) **2d**·Cl^--TBA^+_E, and (iv) **2d**·Cl^--$TPeA^+$, and (e)(i) **2e**, (ii) **2e**·Cl^--TPA^+, (iii) **2e**·Cl^--TBA^+, and (iv) **2e**·Cl^--$TPeA^+$. Photographs of single crystals are shown for **2a**, **2a**·Cl^--TBA^+, **2a**·Cl^--$TPeA^+$, **2b**, **2c**, **2c**·Cl^--TPA^+, **2c**·Cl^--TBA^+, **2c**·Cl^--$TPeA^+$, **2d**, **2d**·Cl^--TBA^+_E, and **2e**, whereas the solid-state **2d**·Cl^--TPA^+, **2d**·Cl^--$TPeA^+$, **2e**·Cl^--TPA^+, **2e**·Cl^--TBA^+, and **2e**·Cl^--$TPeA^+$ were obtained as precipitates from CH_2Cl_2/*n*-hexane. In (a)(i), reflections of excitation light at $UV_{365\ nm}$ were observed in partially pink color.

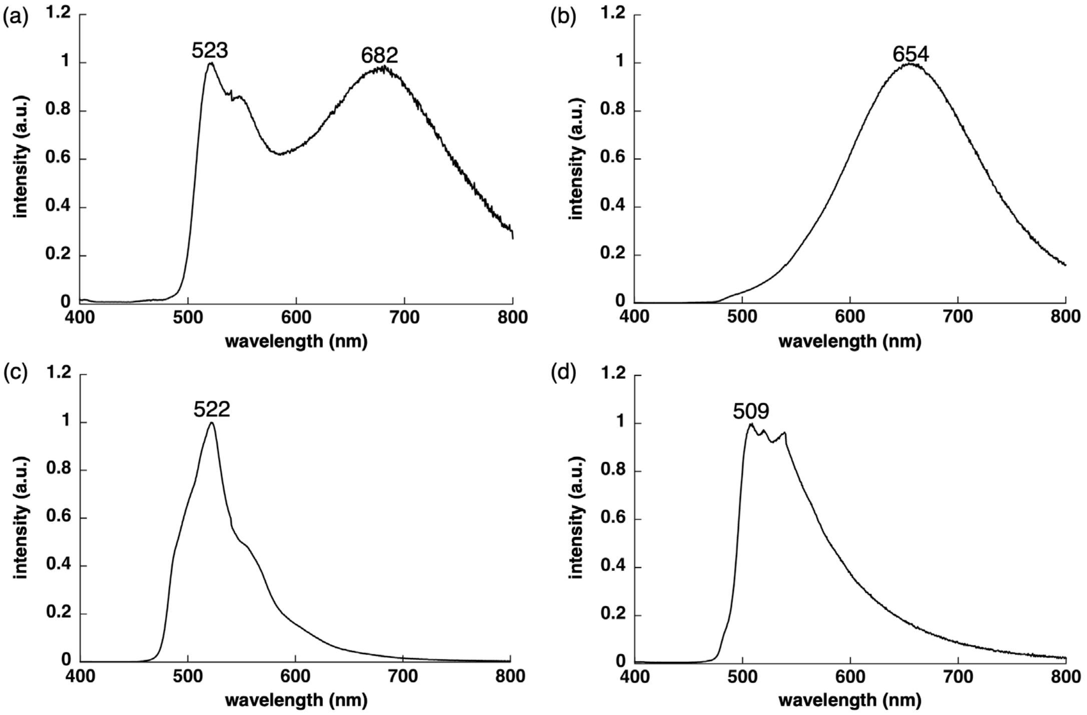

Fig. S86 Solid-state emission spectra of (a) **2a**, (b) **2a**·Cl^{-}-TPA^{+}, (c) **2a**·Cl^{-}-TBA^{+}, and (d) **2a**·Cl^{-}-$TPeA^{+}$, as single crystals for **2a**, **2a**·Cl^{-}-TBA^{+}, and **2a**·Cl^{-}-$TPeA^{+}$ and precipitates from CH_2Cl_2/*n*-hexane for **2a**·Cl^{-}-TPA^{+}, excited at 365 nm.

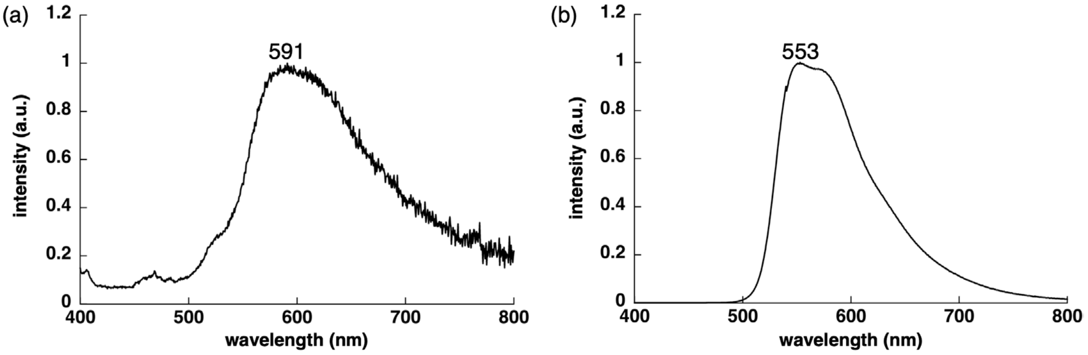

Fig. S87 Solid-state emission spectra of (a) **2b** and (b) **2b**·Cl^{-}-TPA^{+}, as single crystals for **2b** and precipitates from CH_2Cl_2/*n*-hexane for **2b**·Cl^{-}-TPA^{+}, excited at 365 nm. The data for **2b**·Cl^{-}-TBA^{+} and **2b**·Cl^{-}-$TPeA^{+}$ were not evaluated due to the formation of [4+1]-type receptor–anion complexes in the single crystals.

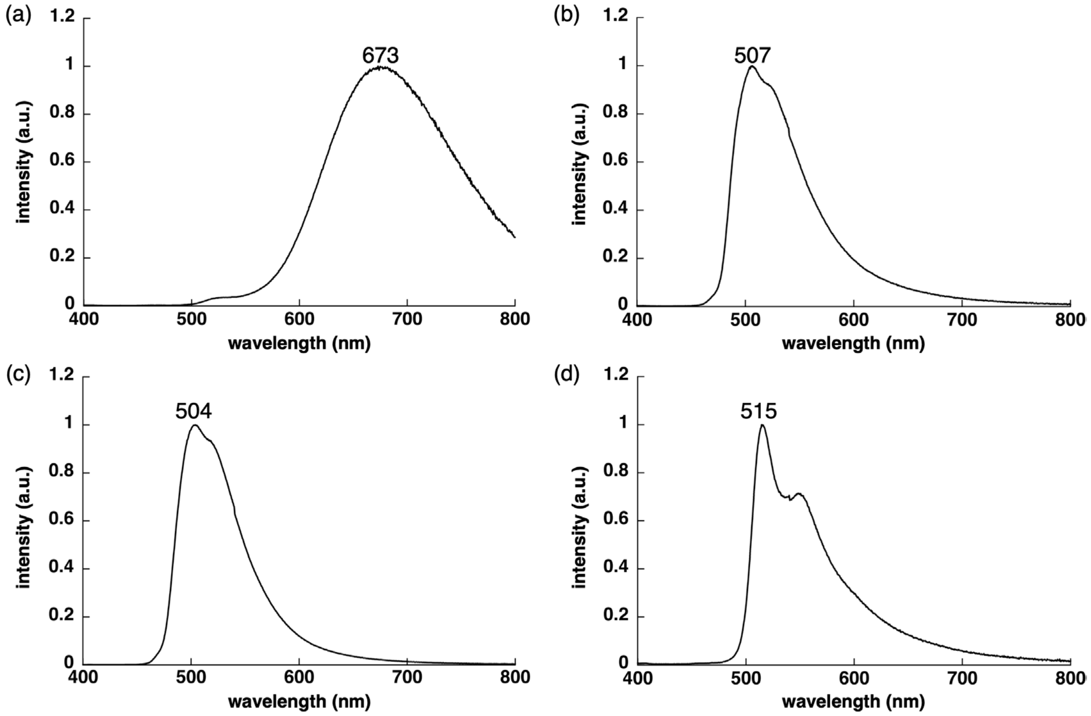

Fig. S88 Solid-state emission spectra of (a) **2c**, (b) **2c**·Cl^{-}-TPA^{+}, (c) **2c**·Cl^{-}-TBA^{+}, and (d) **2c**·Cl^{-}-$TPeA^{+}$, as single crystals, excited at 388 nm.

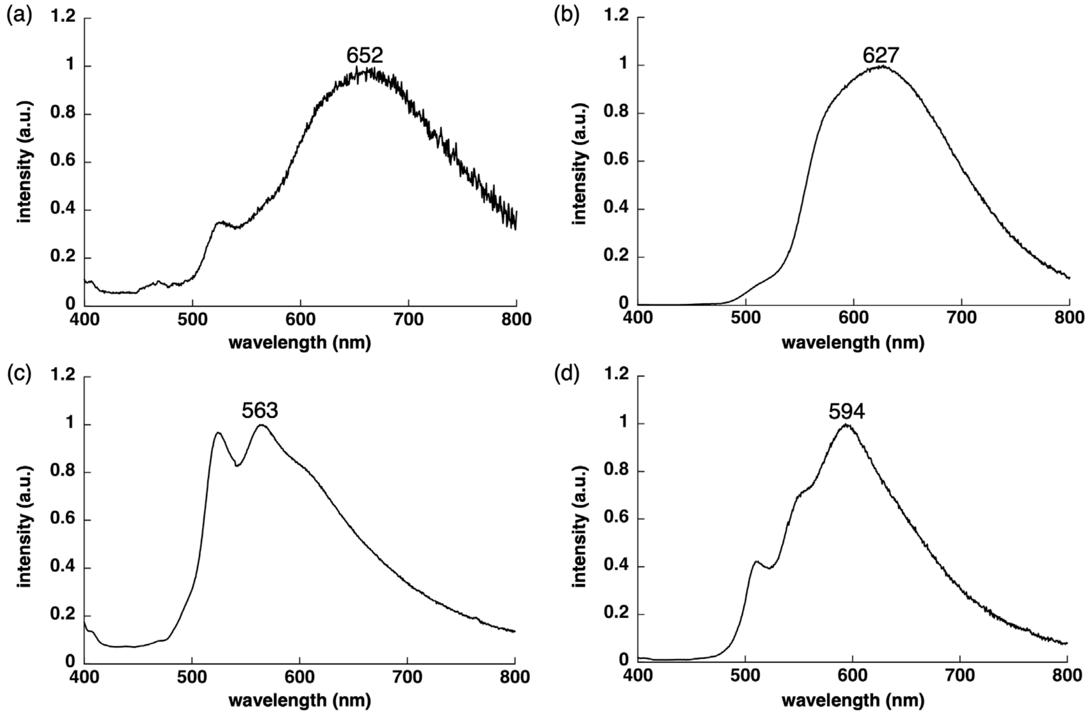

Fig. S89 Solid-state emission spectra of (a) **2d**, (b) **2d**·Cl^{-}-TPA^{+}, (c) **2d**·Cl^{-}-$TBA^{+}{}_{E}$, and (d) **2d**·Cl^{-}-$TPeA^{+}$, as single crystals for **2d** and **2d**·Cl^{-}-$TBA^{+}{}_{E}$ and precipitates from CH_2Cl_2/*n*-hexane for **2d**·Cl^{-}-TPA^{+} and **2d**·Cl^{-}-$TPeA^{+}$, excited at 365 nm.

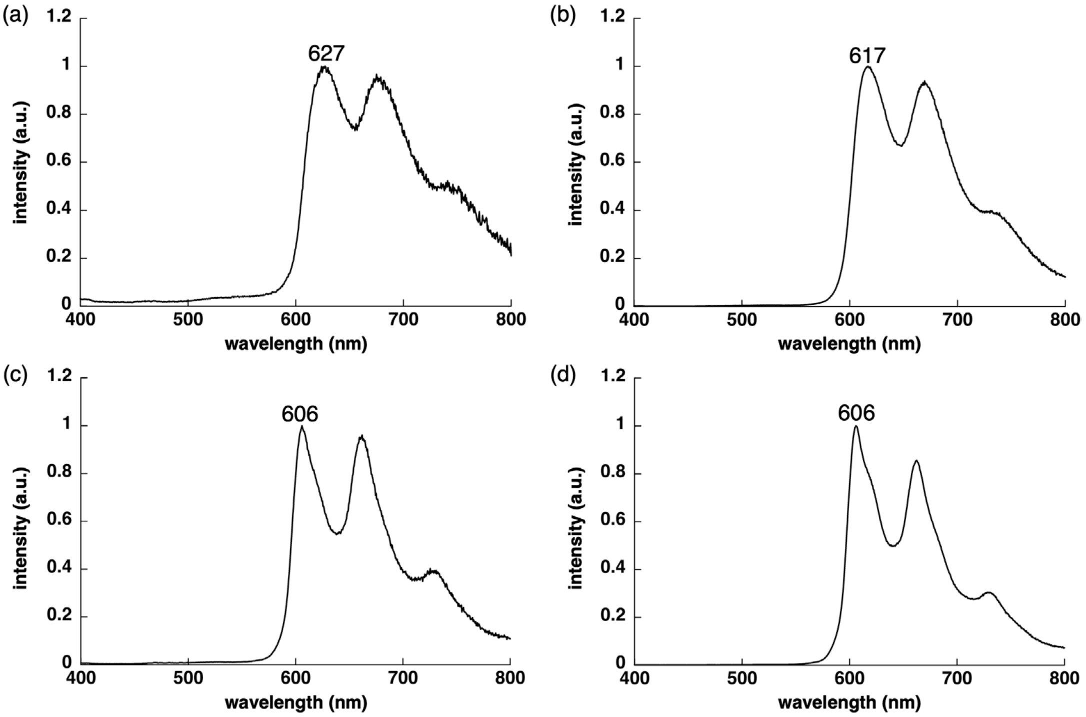

Fig. S90 Solid-state emission spectra of (a) **2e**, (b) **2e**·Cl^--TPA^+, (c) **2e**·Cl^--TBA^+, and (d) **2e**·Cl^--$TPeA^+$, as single crystals for **2e** and precipitates from CH_2Cl_2/*n*-hexane for **2e**·Cl^--TPA^+, **2e**·Cl^--TBA^+, and **2e**·Cl^--$TPeA^+$, excited at 365 nm.

Table S8 Summarized solid-state properties (emission peaks, emission lifetimes, and quantum yields) of **2a**–**e** and their ion pairs of Cl^- complexes with tetraalkylammonium cations.

samples	emission peak (nm)	τ_1 (μs) / f_1 (%)	τ_2 (μs) / f_2 (%)	τ_3 (μs) / f_3 (%)	Φ_{em} (%)
**2a**	523	140 / 31	550 / 69		0.7
**2a**·Cl^--TPA^+	654	110 / 24	440 / 76		3.6
**2a**·Cl^--TBA^+	522	2.9 / 43	23 / 25	210 / 32	6.2
**2a**·Cl^--$TPeA^+$	509	6.3 / 8	86 / 24	440 / 68	2.6
**2b**	591	120 / 27	490 / 73		~0.1[a]
**2b**·Cl^--TPA^+	533	18 / 15	300 / 85		7.5
**2c**	673	61 / 90	450 / 10		1.8
**2c**·Cl^--TPA^+	507	49 / 15	390 / 85		1.7
**2c**·Cl^--TBA^+	504	6.3 / 41	80 / 14	410 / 44	3.2
**2c**·Cl^--$TPeA^+$	515	7.7 / 10	57 / 25	390 / 65	2.4
**2d**	652	120 / 28	500 / 72		~0.1[a]
**2d**·Cl^--TPA^+	627	29 / 36	190 / 64		1.7
**2d**·Cl^--TBA^+_E	563	120 / 26	490 / 74		0.7
**2d**·Cl^--$TPeA^+$	594	30 / 17	330 / 83		0.8
**2e**	627	100 / 21	440 / 79		~0.2[a]
**2e**·Cl^--TPA^+	617	130 / 28	510 / 72		1.9
**2e**·Cl^--TBA^+	606	130 / 27	510 / 73		1.1
**2e**·Cl^--$TPeA^+$	606	96 / 18	420 / 82		2.7

[a] Accurate values could not be determined due to low Φ_{em}.

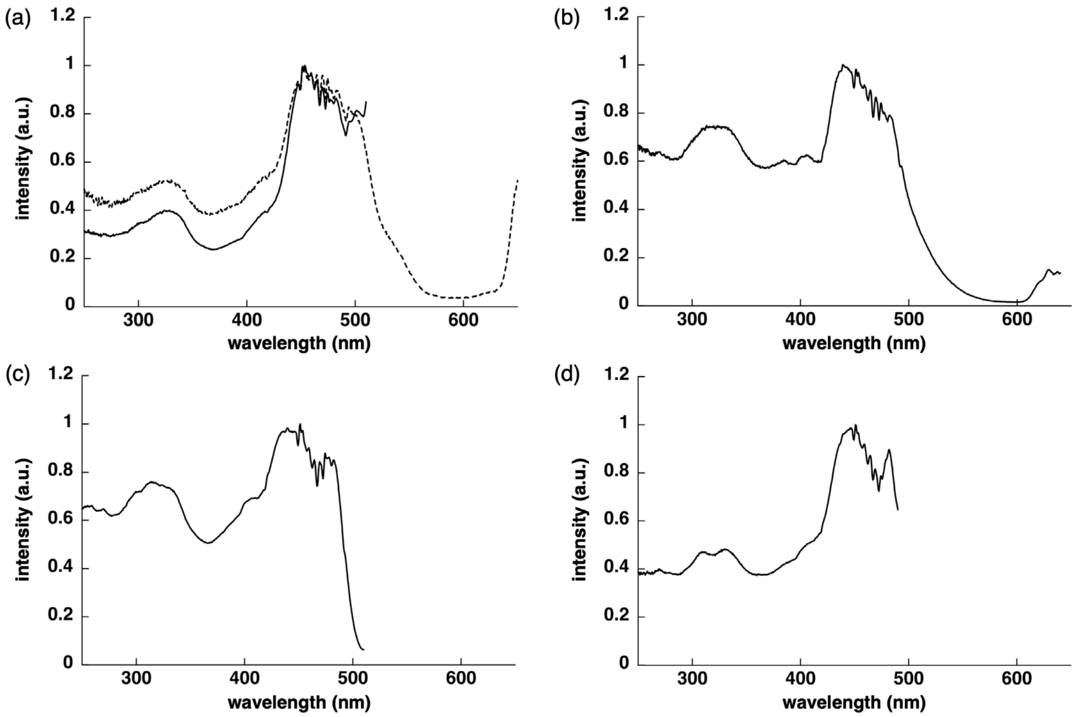

Fig. S91 Solid-state excitation spectra of (a) **2a** (λ_{em} = 523 (solid line) and 682 (broken line) nm), (b) **2a**·Cl^--TPA^+ (λ_{em} = 655 nm), (c) **2a**·Cl^--TBA^+ (λ_{em} = 522 nm), and (d) **2a**·Cl^--$TPeA^+$ (λ_{em} = 509 nm) as single crystals for **2a**, **2a**·Cl^--TBA^+, and **2a**·Cl^--$TPeA^+$ and precipitates from CH_2Cl_2/*n*-hexane for **2a**·Cl^--TPA^+.

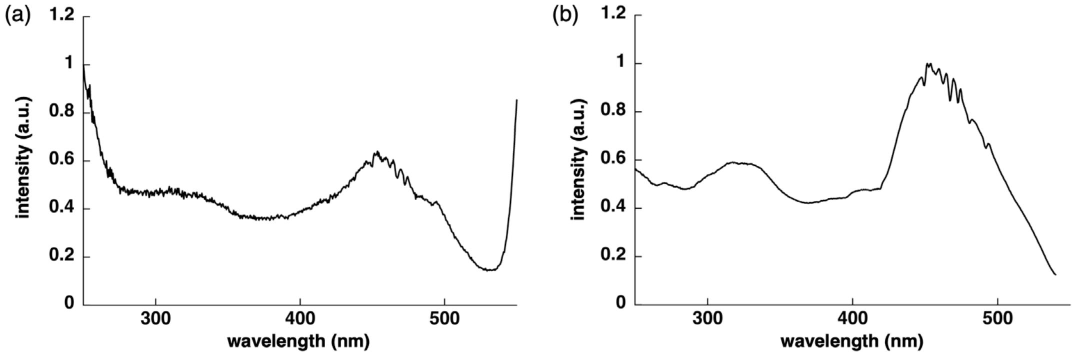

Fig. S92 Solid-state excitation spectra of (a) **2b** (λ_{em} = 591 nm) and (b) **2b**·Cl^--TPA^+ (λ_{em} = 553 nm) as single crystals for **2b** and precipitates from CH_2Cl_2/*n*-hexane for **2b**·Cl^--TPA^+. The data for **2b**·Cl^--TBA^+ and **2b**·Cl^--$TPeA^+$ were not evaluated due to the formation of [4+1]-type receptor–anion complexes in the single crystals.

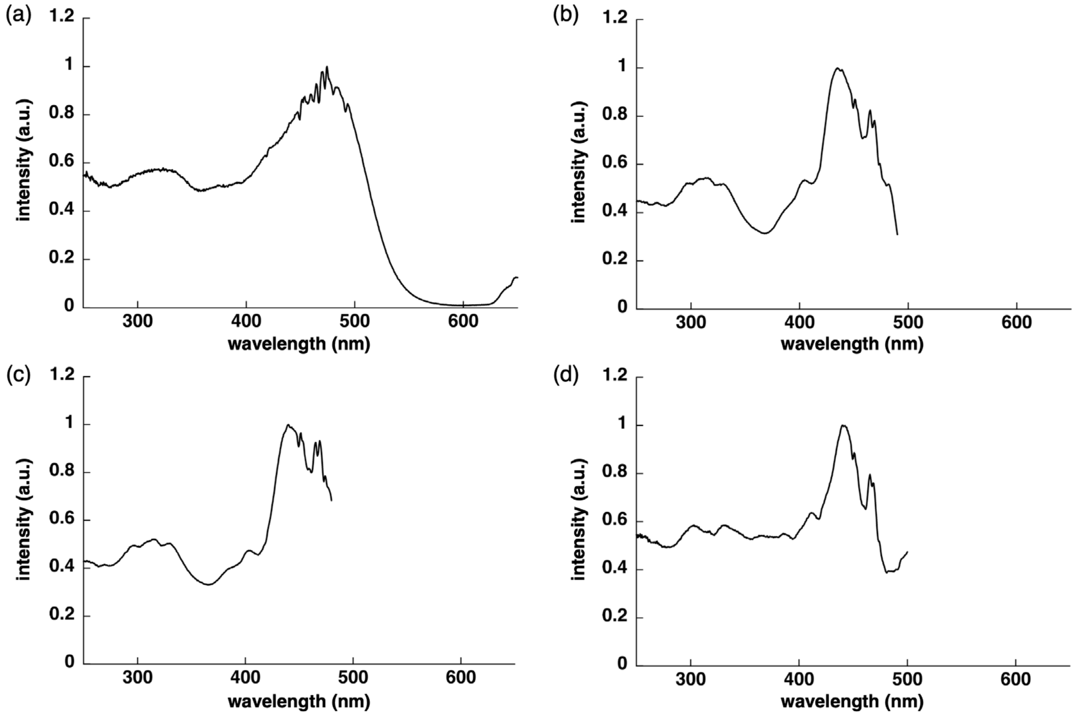

Fig. S93 Solid-state excitation spectra of (a) **2c** (λ_{em} = 673 nm), (b) **2c**·Cl⁻-TPA⁺ (λ_{em} = 507 nm), (c) **2c**·Cl⁻-TBA⁺ (λ_{em} = 504 nm), and (d) **2c**·Cl⁻-TPeA⁺ (λ_{em} = 515 nm) as single crystals.

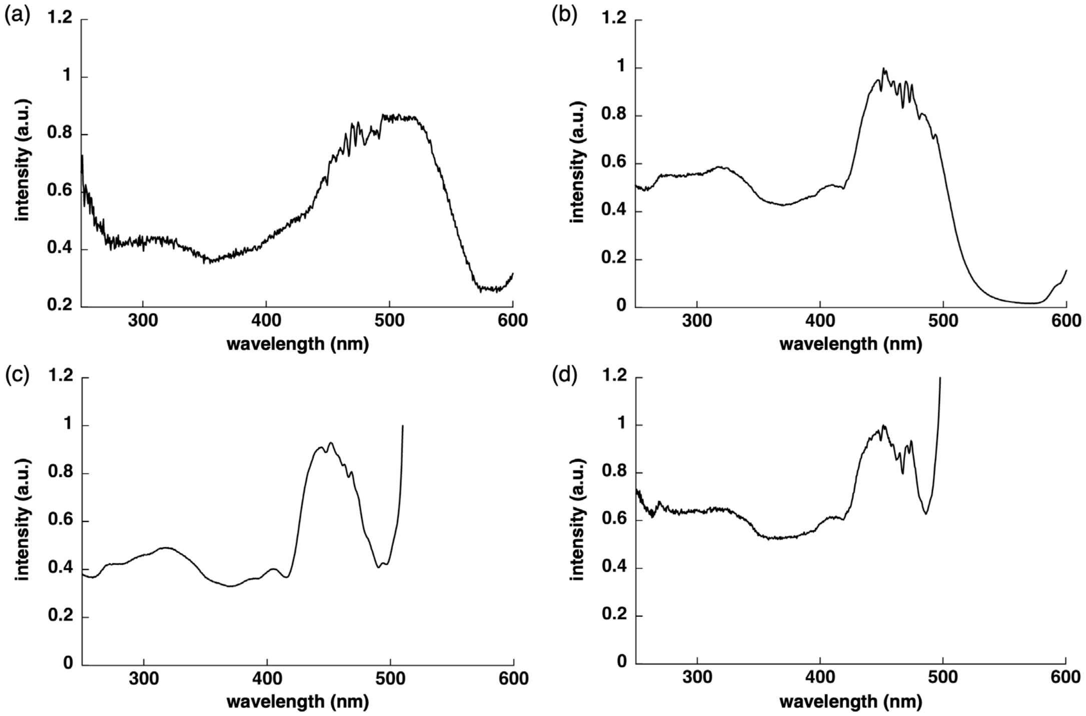

Fig. S94 Solid-state excitation spectra of (a) **2d** (λ_{em} = 652 nm), (b) **2d**·Cl⁻-TPA⁺ (λ_{em} = 628 nm), (c) **2d**·Cl⁻-TBA$^{+}_{E}$ (λ_{em} = 524 nm), and (d) **2d**·Cl⁻-TPeA⁺ (λ_{em} = 512 nm) as precipitates from CH_2Cl_2/*n*-hexane.

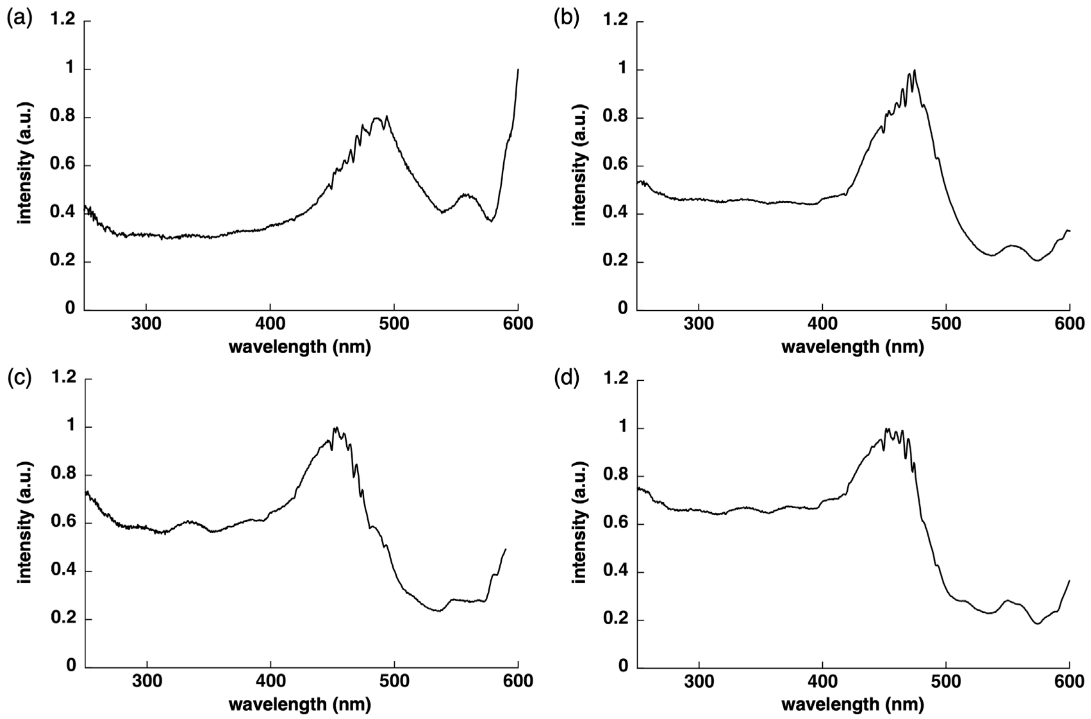

Fig. S95 Solid-state excitation spectra of (a) **2e** (λ_{em} = 627 nm), (b) **2e**·Cl^--TPA^+ (λ_{em} = 617 nm), (c) **2e**·Cl^--TBA^+ (λ_{em} = 606 nm), and (d) **2e**·Cl^--$TPeA^+$ (λ_{em} = 606 nm) as precipitates from CH_2Cl_2/*n*-hexane.
